# Total Synthesis of the Alleged Structure of Crenarchaeol Enables Structure Revision**

**DOI:** 10.1002/anie.202105384

**Published:** 2021-06-30

**Authors:** Mira Holzheimer, Jaap S. Sinninghe Damsté, Stefan Schouten, Remco W. A. Havenith, Ana V. Cunha, Adriaan J. Minnaard

**Affiliations:** ^1^ Stratingh Institute for Chemistry University of Groningen Nijenborgh 7 9747 AG Groningen The Netherlands; ^2^ Zernike Institute for Advanced Materials University of Groningen Nijenborgh 4 9747 AG Groningen The Netherlands; ^3^ Ghent Quantum Chemistry Group Department of Chemistry Ghent University Krijgslaan 281 (S3) 9000 Gent Belgium; ^4^ Eenheid Algemene Chemie (ALGC) Vrije Universiteit Brussel (VUB) Pleinlaan 2 1050 Brussels Belgium; ^5^ NIOZ Royal Netherlands Institute for Sea Research Department of Marine Microbiology and Biogeochemistry PO Box 59 1790 AB Den Burg The Netherlands; ^6^ Faculty of Geosciences Department of Earth Sciences Utrecht University PO Box 80.021 3508 TA Utrecht The Netherlands

**Keywords:** archaea, crenarchaeol, structure revision, tetraether lipid, total synthesis

## Abstract

Crenarchaeol is a glycerol dialkyl glycerol tetraether lipid produced exclusively in Archaea of the phylum Thaumarchaeota. This membrane‐spanning lipid is undoubtedly the structurally most sophisticated of all known archaeal lipids and an iconic molecule in organic geochemistry. The 66‐membered macrocycle possesses a unique chemical structure featuring 22 mostly remote stereocenters, and a cyclohexane ring connected by a single bond to a cyclopentane ring. Herein we report the first total synthesis of the proposed structure of crenarchaeol. Comparison with natural crenarchaeol allowed us to propose a revised structure of crenarchaeol, wherein one of the 22 stereocenters is inverted.

## Graphical abstract

Total synthesis of the proposed structure of crenarchaeol and comparison with the natural isolate has led to a revised structure of crenarchaeol, wherein one of the 22 stereocenters is inverted. The synthesis featured an asymmetric intermolecular Tsuji–Trost‐type alkylation to access the unusual 5‐6‐ring motif as well as a late stage ring‐closing metathesis to access the 66‐membered macrocycle of crenarchaeol.

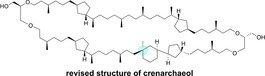

## Introduction

**Figure 1:**
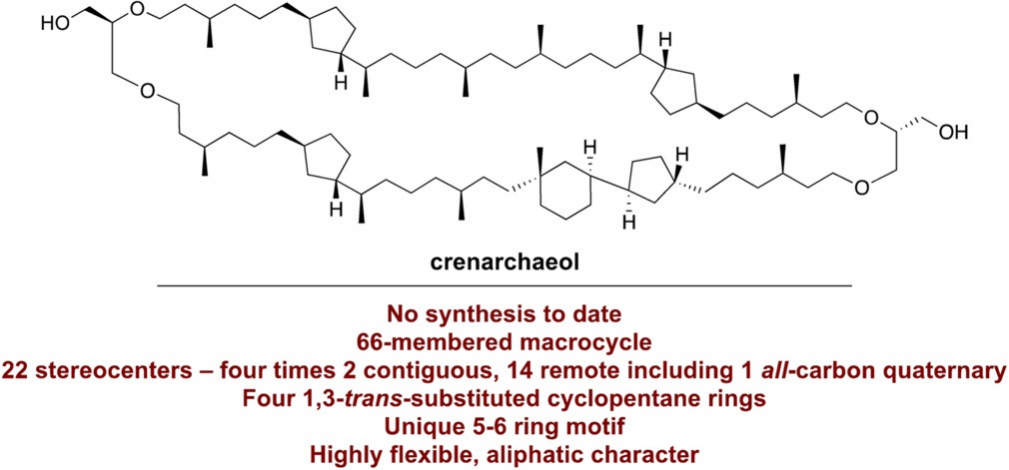
The alleged structure of crenarchaeol.

In 1990, Woese proposed to classify all living organisms in three domains of life: Archaea, Bacteria and Eukarya.^[1]^ Before that, “archaeabacteria” were considered to belong to the Bacteria. Based on differences in their genome and lipidome, Archaea were ultimately recognized as separate, third domain.^[2]^ For a long time, Archaea were primarily associated with extreme habitats such as high temperature, extreme pH, and hypersaline environments.^[3]^ Growing interest over the years, however, led to the discovery of meso‐ and extremophilic Archaea in virtually any habitat on Earth.^[4]^ The cell membrane of Archaea is built up of diether or membrane‐spanning tetraether lipids containing isoprenoid chains, contrary to the straight chain fatty acid glycerol ester lipids found in Bacteria and Eukarya.^[5]^ Apart from the difference in lipid linkage, the stereochemistry of the glycerol backbone in archaeal isoprenoidal glycerol dialkyl glycerol tetraether lipids (GDGTs) is opposite to bacterial or eukaryotic glycerolipids, raising questions on the evolution of archaeal and bacterial/eukaryotic lipid membranes.^[6]^ The lipid composition of Archaea varies, depending on the species and environmental factors, and this is considered an adaptation to their habitat.^[7]^ The ether‐linkages provide chemical stability against hydrolysis, and the presence of methyl‐branches and cyclopentane moieties, which are formed by internal cyclization of the biphytanol chain,^[8]^ leads to decreased membrane permeability, allowing growth at extreme pH, salinity, and temperature.^[9]^ One archaeal GDGT—named crenarchaeol—stands out from all other archaeal membrane lipids due to its unique chemical structure (Figure 1). Crenarchaeol is produced by a specific lineage of Archaea, the Thaumarchaeota,^[10]^ and was first isolated from surface sediments of the Arabian Sea. After extensive GC–MS and NMR analysis, the structure and stereochemistry of this unique GDGT was proposed, a considerable achievement given the fact that the molecular complexity originates merely from its unusual hydrocarbon framework.^[11]^ It contains four 1,3‐*trans*‐substituted cyclopentane moieties. One of these is connected by a single bond to a cyclohexane ring, a structural feature rarely found in natural products.^[12]^ This feature of crenarchaeol is likely formed by further internal cyclization of the bicyclic biphytanyl moiety.^[5b]^ Crenarchaeol contains a total of 22 stereocenters, most of which are remote, including an *all*‐carbon quaternary stereocenter. Recently, a parallel glycerol configuration of sedimentary crenarchaeol was inferred from chemical derivatization experiments.^[13]^ Montenegro et al. confirmed the structure of the bicyclic biphytanyl moiety in archaeal GDGTs by total synthesis,^[14]^ yet to date, there is no proof of structure of the tricyclic biphytanyl moiety of crenarchaeol and no total synthesis. The 5–6 ring motif of crenarchaeol is particularly interesting due to its complexity and uniqueness in nature. In order to ultimately confirm the structure and stereochemistry of crenarchaeol, we embarked on its total synthesis.

## Results and Discussion

**Scheme 1:**
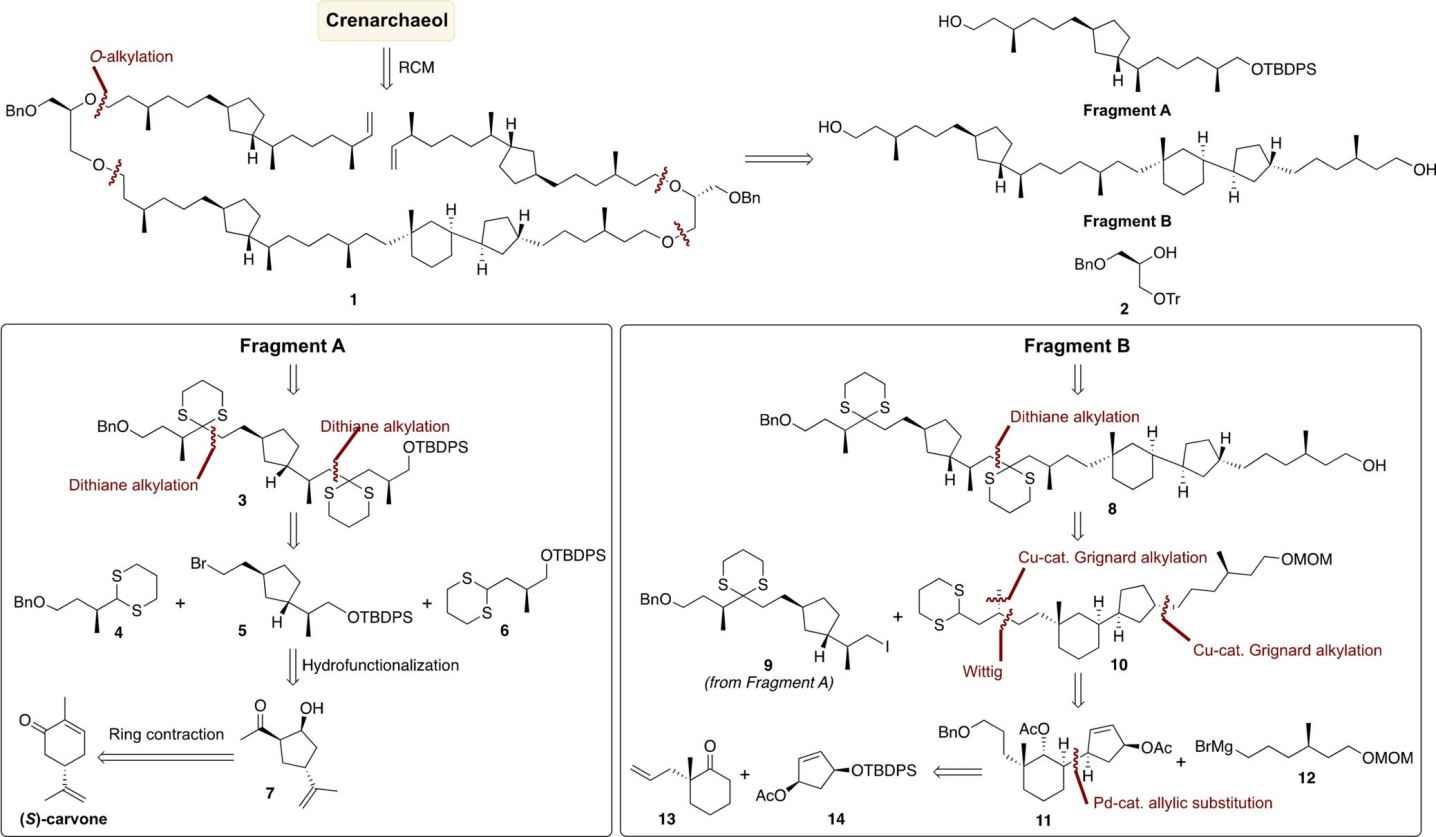
Retrosynthetic analysis of crenarchaeol.

Our retrosynthetic analysis of crenarchaeol made use of the inherent symmetry of the bicyclic biphytanyl chain of the molecule (Scheme 1). It started with the disconnection of the central C−C‐bond of the bicyclic biphytanyl moiety by intramolecular alkene metathesis and ether bond disconnection of **1**. This led to two key intermediates, termed Fragment A and B, and protected glycerol building block **2**. Fragment A can be further simplified via dithiane disconnections to arrive at building blocks **4** and **6**, both carrying a methyl‐branched stereocenter, and cyclopentane building block **5**. In turn, **5** can be traced back to hydroxyketone **7**, which is accessed from commercially available (*S*)‐carvone via ring contraction. Syntheses of archaeal *cis*‐^[15]^ and *trans*‐substituted^[14]^ cyclopentane containing lipids have been previously reported. As we planned to build the macrocycle by alkylation of a suitably functionalized glycerol building block and ring‐closing metathesis, we required differentially protected lipid chains containing the *trans*‐substituted cyclopentane and the methyl‐branches. Based on the stereochemical assessment of the bicyclic biphytane moiety in crenarchaeol^[17]^ and its subsequent confirmation provided by Helmchen et al.,^[22]^ we planned the synthesis of the desired stereoisomer.

Retrosynthesis of Fragment B commenced with the C−C‐bond disconnection of **8** arriving at dithiane **10** and iodide **9**, the latter originating from Fragment A. Further simplification of **10** by asymmetric Cu‐catalyzed Grignard alkylations and a Wittig olefination delivered diacetate **11**. The 5–6 ring motif of **11** was disconnected at the C−C‐bond joining the two carbocycles.^[16]^

We realized that for this challenging transformation an advanced intermolecular Pd‐catalyzed asymmetric allylic alkylation could be instrumental, inspired by the work of Trost.^[17]^ By this, we arrived at building blocks **13** and **14**, readily accessible from pimelic acid and cyclopentadiene, respectively.

### Synthesis of Fragment A

**Scheme 2:**
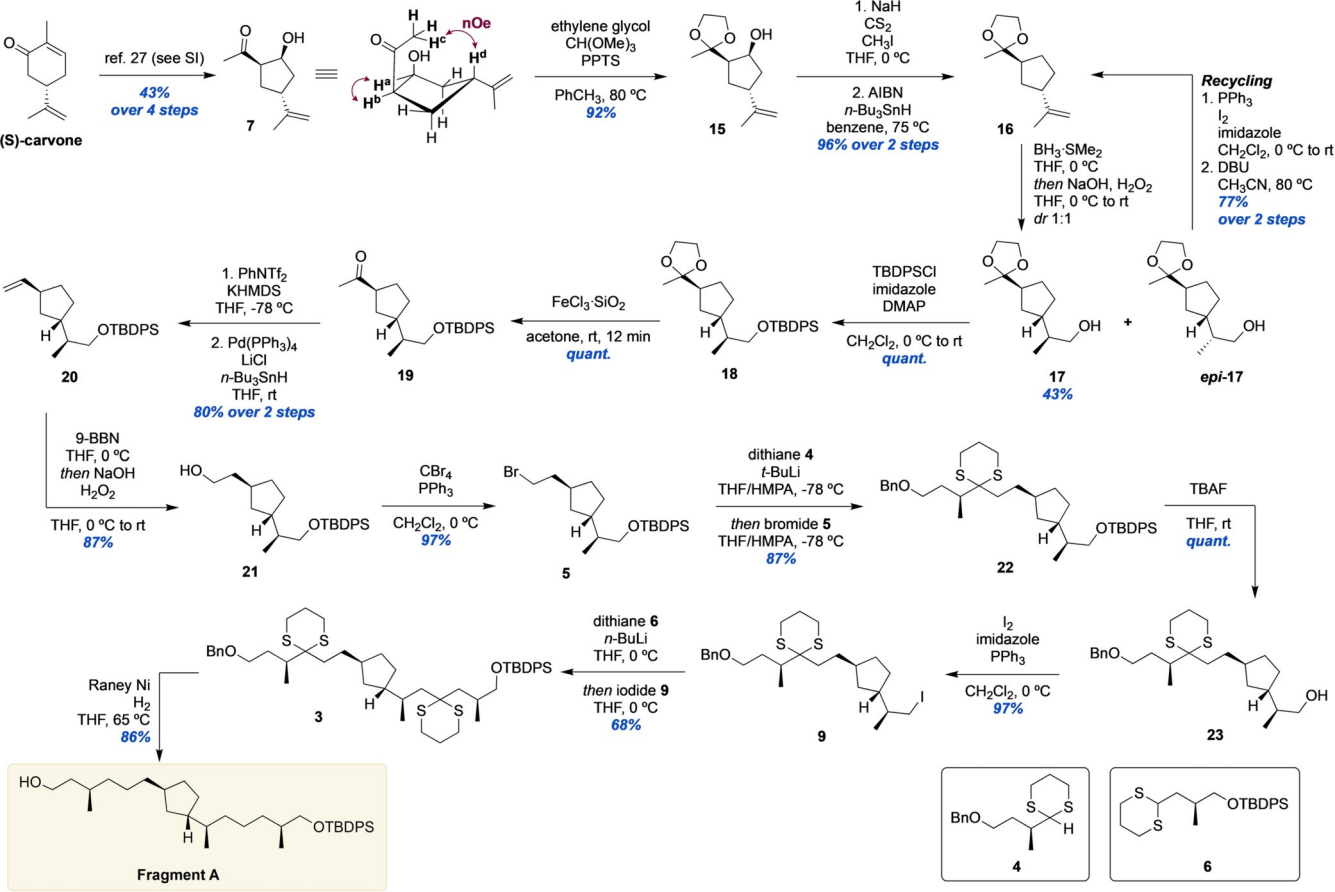
Synthesis of Fragment A.

The synthesis of Fragment A was initiated by the preparation of known β‐hydroxyketone **7** from (*S*)‐carvone (Scheme 2). Via a four‐step sequence involving a hydrolytic ring contraction,^[18]^ **7** was obtained as single diastereomer, as confirmed by NOESY. Notably, this sequence proved robust and scalable and allowed multigram synthesis of **7** (see Supporting Information). After acetal protection of **7**, the hydroxyl group of **15** was removed by Barton‐McCombie deoxygenation, providing **16** in excellent yield. Notably, acetal protection was necessary to avoid elimination of the β‐hydroxyl group in the synthesis of the xanthate intermediate. Initially, we envisioned to stereoselectively install the methyl stereocenter adjacent to the 5‐membered ring by means of Cu‐ or Co‐catalyzed asymmetric hydroboration.^[19]^ No published method to perform the asymmetric hydroboration of the 1,1‐disubstituted terminal alkene of **16** delivered **17** in acceptable yield and stereoselectivity, however. Thus, we resorted to non‐stereoselective hydroboration‐oxidation of **16** followed by diastereomer separation, giving **17** in 43 % yield as single stereoisomer. The stereochemistry of the methyl‐branched center in **17** was determined by amidation of its corresponding acid with phenylglycine methyl ester, followed by ^1^H NMR analysis (See Supporting Information).^[20]^

In addition, the efficiency of the synthesis was further increased by “recycling” of the undesired ***epi*** **‐17** by iodination and elimination, giving alkene **16** in 77 % yield over two steps. After silyl protection of **17**, the acetal moiety of **18** was removed. Optimization of the reaction conditions, to minimize epimerization, resulted in treatment of **17** in acetone with FeCl_3_ adsorbed to silica,^[21]^ giving **19** in quantitative yield with 3 % epimerization. Ketone **19** was converted to the corresponding terminal alkene by enol‐triflation and Pd‐catalyzed triflate reduction, delivering **20** in 80 % yield over two steps. Hydroboration‐oxidation of **20** gave alcohol **21** in 87 % yield, which was converted to the corresponding bromide **5** in excellent yield. With **5** in hand, the stage was set for the first dithiane alkylation.^[22]^ After optimization of the lithiation conditions of **4** (prepared using known methods, see Supporting Information), the alkylation proceeded in high yield (87 %) giving **22**. Desilylation followed by Appel iodination delivered iodide **9**, which serves as intermediate in the synthesis of both Fragment A and B. In turn, after identification of the optimal lithiation conditions, deprotonation of dithiane **6** with *n*‐BuLi at 0 °C followed by addition of **9** produced bis‐dithiane **3** in 68 % yield. With the carbon skeleton of Fragment A constructed, the dithiane moieties and the benzyl ether of **3** were removed by Raney–nickel reduction in good yield, thus concluding the synthesis of Fragment A.

### Synthesis of Fragment B

**Scheme 3:**
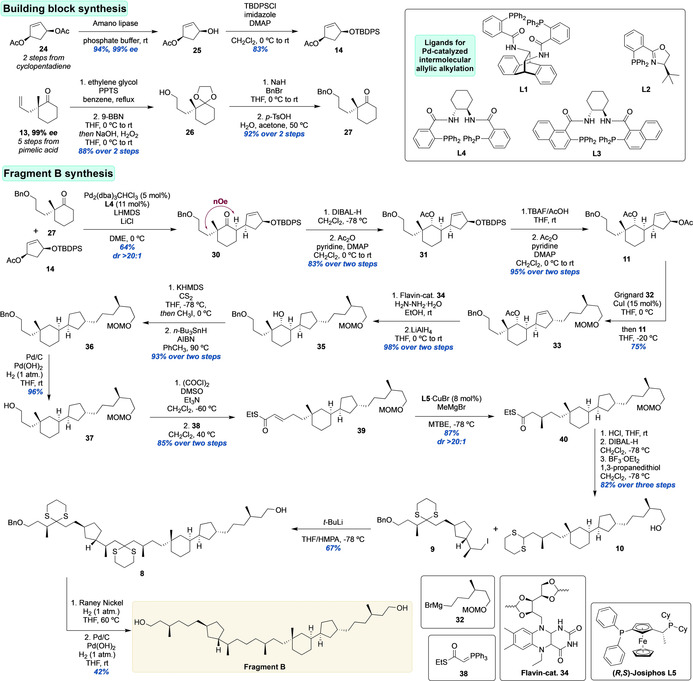
Synthesis of Fragment B.

Next, the considerably more complex Fragment B was to be constructed. The synthesis started with the preparation of two building blocks **14** and **27** (Scheme 3). The synthesis of cyclopentene **14** started from *meso*‐diacetate **24**, accessible in two steps from cyclopentadiene.^[23]^ Diacetate **24** was subjected to enzymatic desymmetrization^[24]^ in excellent yield and *ee*, followed by silyl protection giving **14**. Cyclohexanone **27** was prepared according to the method developed by the Stoltz laboratory from allyl cyclohexanone **13**,^[25]^ which was protected and subjected to hydroboration/oxidation to deliver **26**. Omission of the protection of the ketone in **13** led to the formation of the corresponding hemiacetal. Benzylation and acetal hydrolysis provided the desired cyclohexanone **27** in 92 % yield over two steps.

With acetate **14** in hand, we chose to investigate the key step—the intermolecular Pd‐catalyzed Tsuji–Trost alkylation—with 2,2‐dimethylcyclohexanone **28** as model substrate (Table 1).

**Table 1:** Optimization of the Pd‐catalyzed allylic alkylation. 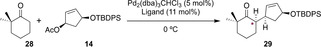

Entry^[a]^	Ligand	Base	Solvent^[c]^	Conversion^[b]^ (yield)^[c]^	*dr* ^[d]^
1	L1	LHMDS	THF	80 %	25:75
2	L2	LHMDS	THF	40 %	51:49
3	L3	LHMDS	THF	40 %	81:19
4	L4	LHMDS	THF	40 % (27 %)	86:14
5	L4	LHMDS	PhCH_3_	40 %	85:15
6	L4	LHMDS	DME	42 %	94:6
7	L4	NaHMDS	DME	10–15 %	92:8
8	L4	LDA	DME	41 %	92:8
9^[e]^	L4	LHMDS	DME	**full (53 %)**	93:7

[a] See Supporting Information for details. [b] Determined by ^1^H NMR. [c] Isolated yield. [d] Determined by ^13^C NMR of the crude product. [e] 1.6 equiv. of LHMDS and 3 equiv. LiCl were used.

We started by screening ligands **L1**–**L4** (Scheme 3) in combination with Pd_2_(dba)_3_CHCl_3_ in order to achieve good chiral induction. In presence of LHMDS as base and THF as solvent at 0 °C, (*R*,*R*)‐ANDEN‐Phenyl Trost **L1** gave good conversion to the alkylation product **29**, albeit with a *dr* of 75:25 favoring the undesired diastereomer (Table 1, entry 1). Under the same conditions, (*R*)‐t‐ButylPHOX **L2** failed to give chiral induction (Table 1, entry 2). When using DACH ligands **L3** and **L4**, good diastereoselectivities of 81:19 and 86:14 were achieved (entries 3 and 4), yet with a low conversion of around 40 % and in the case of **L4** only 27 % isolated yield. Since acceptable stereo‐induction was achieved, we continued the optimization with **L4**. Changing the solvent to toluene or DME (Table 1, entry 5 and 6) did not result in higher conversion, but the latter gave the product with improved *dr* of 94:6. When using NaHMDS the conversion dropped significantly to around 10–15 % (Table 1, entry 7), while LDA performed comparable to LHMDS (entry 8). Ultimately, increasing the equivalents of LHMDS to 1.6 and using LiCl as additive resulted in full conversion (Table 1, entry 9). The product was isolated in 53 % yield with an excellent *dr* of 93:7.

We decided to apply these conditions to acetate **14** and cyclohexanone **27**, and found this system to be superior to the model reaction. Product **30** was obtained in 67 % yield with a *dr* >20:1, and no undesired diastereomer detected (Scheme 3). This variant of the intermolecular Pd‐catalyzed asymmetric allylic alkylation further expands the toolbox of this type of reaction and we expect it to open up new avenues for future asymmetric construction of joint ring systems in a convergent manner.

Progressing the synthesis of Fragment B, the ketone moiety was reduced and acetylated, giving **31** as single diastereomer. Subsequent desilylation and acetylation delivered diacetate **11** in excellent yield. Notably, attempts to shorten this sequence by performing reduction, desilylation, and double acetylation led to significantly lower yields. This was due to the formation of a tricyclic product arising from S_N_2′ addition of the non‐allylic hydroxy group to the double bond (see Supporting Information). With diacetate **11** in hand, a regioselective copper‐catalyzed Grignard alkylation with **32** (prepared from (*R*)‐citronellol, see Supporting Information) was performed providing a crude *dr* of 4:1 and, after separation of the isomers, alkylation product **33** in 75 % yield as single stereoisomer. The double bond of **33** was reduced by a flavin‐catalyzed diimide reduction^[26]^ followed by deacetylation providing **35** in 98 % yield over two steps. The hydroxyl moiety of **35** was then removed by a Barton‐McCombie deoxygenation reaction in excellent yield. After Pd‐catalyzed hydrogenolysis of the benzyl ether in **36**, alcohol **37** was oxidized to the corresponding aldehyde and subjected to a Wittig olefination delivering α,β‐unsaturated thioester **39**. The last methyl‐branched stereocenter of Fragment B was then introduced in an excellent *dr* of 20:1 (see Supporting Information for details) by copper‐catalyzed asymmetric conjugate addition of methylmagnesium bromide^[27]^ producing **40** in 87 % yield. With the last stereocenter of the biphytane core of crenarchaeol set, the dithiane moiety of **10** was installed, after MOM deprotection of **40**, through thioester reduction and treatment with 1,3‐propanedithiol in the presence of BF_3_⋅OEt_2_. Dithiane **10** was obtained in 82 % over the three steps. Notably, dithiane synthesis in presence of the MOM ether resulted in a complex mixture of **10** and various trans‐acetalization products. With **10** in hand, the last dithiane alkylation was performed, in presence of the free hydroxyl group. After optimization of the lithiation conditions, the reaction of lithiated **10** with iodide **9** smoothly provided the coupling product **8** in 67 % yield, containing the entire carbon‐skeleton of Fragment B. The synthesis of Fragment B was concluded by a two‐step sequence, involving removal of the dithianes with Raney‐nickel, followed by Pd‐catalyzed hydrogenolysis of the remaining benzyl ether.

### Endgame—Completion of the Total Synthesis of the Proposed Structure of Crenarchaeol

**Scheme 4:**
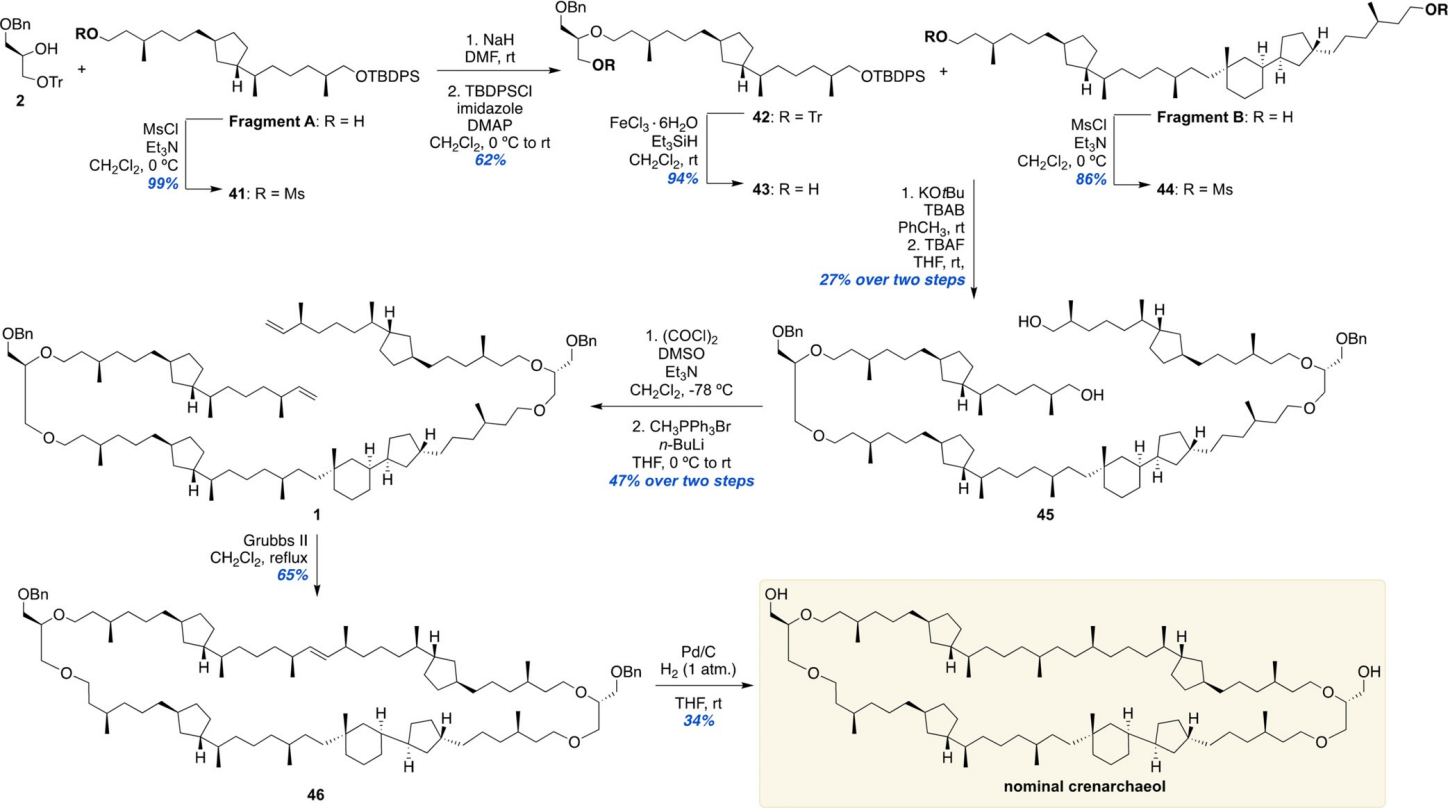
Completion of the synthesis of the proposed structure of crenarchaeol.

After the successful stereoselective synthesis of both Fragment A and B, the macrocycle of crenarchaeol was assembled (Scheme 4). The endgame of the synthesis started with the *O*‐alkylation of protected glycerol **2** with mesylate **41** prepared from Fragment A. During the reaction using sodium hydride in DMF, partial cleavage of the TBDPS ether was observed. Therefore, after *O*‐alkylation, the silyl ether was reintroduced, giving alkylation product **42** in 62 % yield. The trityl ether was removed delivering **43**, the substrate for the next ether synthesis, in 94 % yield. The double *O*‐alkylation of **43** with bis‐mesylate **44** came about after considerable experimentation, by reaction with KO*t*Bu as the base in toluene in the presence of TBAB as phase‐transfer catalyst. After desilylation of the crude double alkylation product, the desired diol **45** was obtained in a poor yield of 27 % over the two steps. There are multiple factors complicating this reaction. It is a double *O*‐alkylation of a bis‐mesylate. The sheer size and flexibility of this electrophile plays a role in the reaction rate as we expect that the site of alkylation is not always exposed for reaction with the weak alkoxide nucleophile. In addition, small amounts of elimination products were observed. Consequently, given the difficulty of this step, we continued with the synthesis. In order to perform the final ring closure of the macrocycle, **45** was converted to bis‐alkene **1** by oxidation and Wittig reaction. The 66‐membered macrocycle was closed by means of ring‐closing metathesis with Grubbs 2^nd^ generation catalyst, a method often used for the construction of large rings.^[28]^ This provided **46** in 65 % yield, given the size of the produced macrocycle a more than satisfactory result. In the final step, the double bond as well as the benzyl ethers were removed by hydrogenolysis with palladium on carbon in low yield of 34 %, which could be partially attributed to the scale of the reaction. This concluded the synthesis of this structurally complex lipid and provided 1.2 mg of synthetic crenarchaeol. With both synthetic crenarchaeol and the tricyclic intermediate Fragment B in hand we sought to investigate the chemical structure of natural crenarchaeol. For this purpose, we re‐isolated natural crenarchaeol in a laborious procedure (see Supporting Information) and made a comparison of their NMR spectra. Furthermore, we performed chemical derivatization in combination with GC–MS analysis.

### Comparison of Natural Crenarchaeol and Fragment B by GC–MS

**Figure 2:**
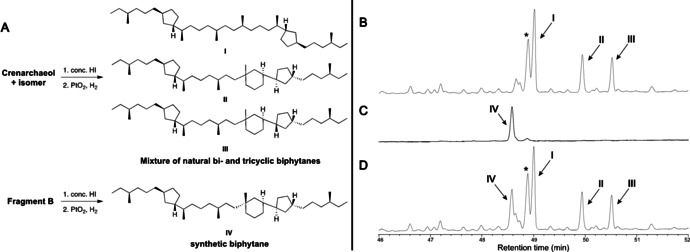
A: Conversion of natural crenarchaeol and Fragment B into biphytanes. B–D: Partial gas chromatograms of the formed biphytane(s). B: biphytanes I–III from the GDGTs in the Bligh Dyer extract of “*Ca. Nitrosotenuis uzonensis*”.^[29]^ C: biphytane IV from Fragment B. D: Co‐injection of IV with the biphytane mixture of “*Ca. N. uzonensis*”. * Indicates an isomeric bicyclic biphytane, most likely originating from GDGT‐4.

The Bligh Dyer extract of the thermophilic Thaumarchaeota “*Ca. Nitrosotenuis uzonensis*” (dominated by crenarchaeol and its *cis*‐cyclopentyl isomer,^[29]^ see Figure 2 A) has previously been treated with HI. This cleaves the ether bonds to produce a mixture of biphytane diiodides.^[29]^ Reduction of the iodides with H_2_/PtO_2_ led to the corresponding hydrocarbons **I**–**III**, which were analyzed by GC–MS.^[29]^ This showed a ratio of bi‐ and tricyclic biphytanes of approximately 1:1 (Figure 2 B). As a direct comparison of the configuration of the tricyclic biphytane unit within synthetic and natural crenarchaeol was considered complicated, we subjected also Fragment B to this derivatization (Figure 2 A).[^29^, ^30^] This enabled a precise comparison by GC–MS. Treatment of fragment B with HI followed by reduction yielded biphytane **IV** which appeared, as expected, as a single peak in the gas chromatogram (Figure 2 C), but much to our surprise with a significantly different retention time than the supposedly identical **II** derived from natural crenarchaeol. The mismatch in chemical structure was confirmed by co‐injection, showing retention time differences of **IV** and **II** or **III** of approximately 1.5 and 2 min, respectively (Figure 2 D).

Next, we turned our attention to the mass spectra of **II**–**IV** (see Supporting Information). The fragmentation patterns of natural **II** and **III** were equivalent to their previously reported mass spectra,[^29^, ^31^] and featured the characteristic fragment *m*/*z* 262, originating from bond cleavage adjacent to the quaternary stereocenter. This fragment was also clearly visible in the mass spectrum of synthetic **IV**.

Furthermore, the remaining fragmentation patterns of **II/III** and **IV** are also virtually identical, providing strong evidence that the overall chemical connectivity of **II/III** and synthetic **IV** is identical. Thus, we concluded that **II** and **IV** are stereoisomers.

### Comparison of Fragment B with Isolated Natural Crenarchaeol by NMR

In order to elucidate the exact structural difference between synthetic Fragment B and the tricyclic biphytanyl moiety of natural crenarchaeol, we compared their NMR spectra. The ^1^H and ^13^C signals of natural crenarchaeol^[11]^ and Fragment B were assigned by thorough 2D NMR analysis. In addition, the ^13^C signals of synthetic crenarchaeol were assigned based on the NMR analysis of Fragment B.

The comparison of selected ^13^C NMR signals of Fragment B and synthetic crenarchaeol with those of natural crenarchaeol is shown in Table 2 (see Supporting Information for a table with all signal assignments).

**Table 2:** Comparison of ^13^C NMR values of natural crenarchaeol with Fragment B and synthetic nominal crenarchaeol.

Carbon number^[a]^	^13^C shift natural crenarchaeol (ppm)	^13^C shift Fragment B (ppm)^[b]^	Δ*δ* (ppm)^[c]^
A1, A1′	70.23, 70.26	61.42 (70.28, 70.25)	−8.81, −8.84
A2, A2′	36.72, 36.75	40.14, 40.15 (36.74)	+3.42, +3.40
A11′	39.38	39.01 (39.10)	−0.37
A12′	32.27	31.80 (31.81)	−0.47
A13′	22.40	22.10 (22.12)	−0.30
A14′	44.13	38.17 (38.30)	−5.96
A15′	33.20	32.92 (32.93)	−0.28
A16	30.12	30.50 (30.51)	+0.38
A16′	37.80	33.51 (33.46)	−4.29
A20′	22.55	30.12 (30.13)	+7.57

[a] Assignments of ^13^C NMR chemical shifts of crenarchaeol^[11]^ and Fragment B. Signals are reported relative to the solvent residual signal (CDCl_3_ *δ*=77.16 ppm). [b] Corresponding signals of synthetic nominal crenarchaeol are shown in brackets.

**Figure 3:**
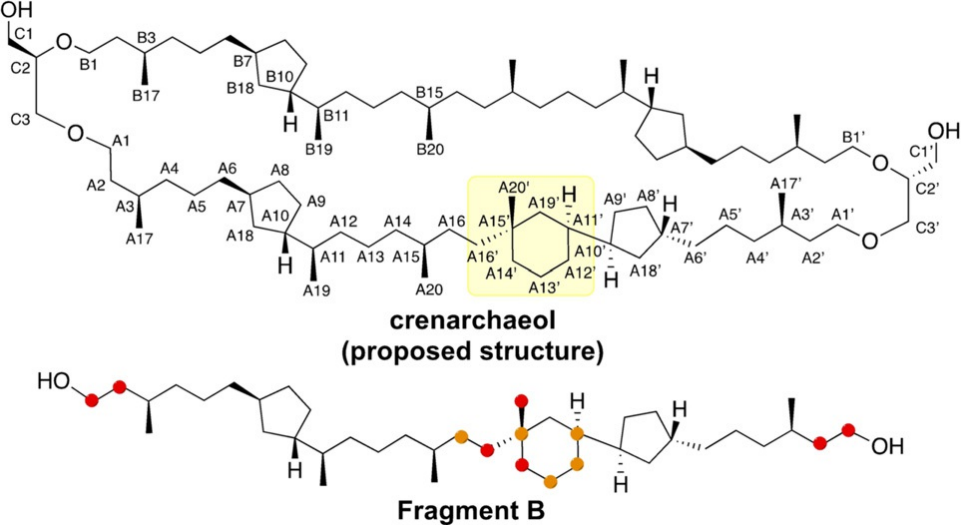
Alleged structure of crenarchaeol with carbon numbering and the synthetic Fragment B. Carbons with moderate (Δ*δ*=0.25–21 ppm) and larger ^13^C chemical shift differences are marked in orange and red, respectively.

The carbon numbering is shown in Figure 3, and significant differences in ^13^C NMR shifts between Fragment B and natural crenarchaeol are marked in orange (Δ*δ*=0.25–1 ppm) and red (Δ*δ*>1 ppm). Upon comparison of the ^13^C NMR signals of Fragment B with those of natural crenarchaeol,^[11]^ it becomes clear that the majority of the chemical shifts of Fragment B are in very good agreement (Δ*δ*<0.25 ppm) with those of the tricyclic biphytane of crenarchaeol. In particular, the ^13^C chemical shifts of the three cyclopentane rings (which are not connected to the cyclohexane ring) and their alkyl substituents are virtually identical (see Supporting Information).

Moderate chemical shift differences (Δ*δ*=0.25–1 ppm) were ascribed to the cyclohexane ring carbons (A11′, A12′, A13′ and A15′) and the alkyl chain adjacent to the cyclohexyl ring (A16). Large differences (Δ*δ*>1 ppm) in chemical shift at the (sub)terminal carbons (A1, A1′, A2 and A2′) of Fragment B originate from the presence of primary hydroxyl moieties contrary to the ether linkages in crenarchaeol. More importantly, however, three carbon atoms around the *all*‐carbon quaternary stereocenter elicit large differences in chemical shift at positions A14′ (Δ*δ*=−5.96 ppm), A16′ (Δ*δ*=−4.29 ppm) and A20′ (Δ*δ*=+7.57 ppm), indicating a difference in structure around these positions. It is noteworthy that the ^13^C signals of the remaining stereocenters of the 5–6‐ring system (A10′ and A7′) in Fragment B show no significant difference. In particular the good agreement of A10′ is indicative for the ascribed stereochemistry of the single bond connecting the 5‐ and 6‐membered ring. It is expected that a difference in stereochemistry on A11′ would translate to a significant ^13^C chemical shift difference in A10′. This indicates that, on these positions, the chemical structure of natural crenarchaeol matches that of Fragment B. The chemical shifts of synthetic nominal crenarchaeol (chemical shifts in brackets in Table 2) show the same pattern of chemical shift differences. It should be highlighted that there is no significant ^13^C chemical shift difference between Fragment B and the tricyclic biphytane of synthetic crenarchaeol (except for the terminal carbons A1/A1′ and A2/A2′) excluding an influence of the macrocyclic structure on the chemical shifts.

Besides the good agreement of most of the ^13^C NMR chemical shifts of crenarchaeol and Fragment B, the ^1^H NMR chemical shifts of A7′, A10′ and A11′ correlate well (Table 3, see Supporting Information for all assignments). At position A19′ (axial) and A20′, only minor ^1^H shift differences were observed. Only three positions show significant chemical shift differences: the equatorial proton of A14′ (Δ*δ*=0.27 ppm), A16′ (Δ*δ*=0.47 ppm) and the equatorial proton of A19′ (Δ*δ*=0.15 ppm). This provides further evidence that the difference in structure of natural and synthetic crenarchaeol is located around these positions.

**Table 3:** Comparison of ^1^H NMR values of natural crenarchaeol and Fragment B.

Carbon number^[a]^	^1^H shift crenarchaeol (ppm)	^1^H shift Fragment B (ppm)
A7′	1.79	1.78
A10′	1.47	1.46
A11′	1.17	1.12
A14′	1.15	1.42
A16′	1.31	1.78
A19′	ax.: 0.70; eq.: 1.39	ax.: 0.64; eq.: 1.52
A20′	0.84	0.79

[a] Assignments of ^1^H NMR chemical shifts of crenarchaeol^[11]^ and Fragment B. Signals are reported relative to the solvent residual signal (CDCl_3_ *δ*=77.16 ppm).

Since the relative and absolute stereochemistry of Fragment B is known, the methyl substituent A20′ of Fragment B is assigned to be equatorial due to the 1,3‐*cis* relationship of the methyl and cyclopentyl substituents on the cyclohexane ring. As a result of the deshielding γ‐gauche effect, the ^13^C NMR chemical shift of axial substituents in cyclohexanes is more upfield relative to equatorial substituents.^[32]^ In Fragment B the ^13^C signal of methyl group A20′ resonates at 30.12 ppm, while the methyl group A20′ of natural crenarchaeol is shifted more upfield at 22.55 ppm. This strongly suggests that the methyl group A20′ in natural crenarchaeol is in axial position in contrast to the initially proposed structure. To further support this, we considered the ^13^C chemical shifts of A16′. In Fragment B, the carbon atom A16′ of the alkyl side‐chain of the cyclohexyl ring is axially oriented. The ^13^C signal resonates at 33.51 ppm, whereas in crenarchaeol the ^13^C signal of A16′ is shifted downfield to 37.80 ppm. Thus, the downfield shift of A16′ in natural crenarchaeol strongly suggests equatorial substitution of the alkyl chain substituent on the cyclohexyl ring.

Further support comes from the computationally calculated ^13^C shift values for A16′ and A20′. First, MD simulations in chloroform were carried out on fragment B and its isomer to determine the lowest energy conformations. Subsequently, the energies of the conformers from the MD trajectory were evaluated using DFT calculations and the chemical shifts calculated (See Supporting Information for the protocol and the calculated shifts). The DFT prediction is in good agreement with the upfield shift of methyl group A20′ in natural crenarchaeol and the expected downfield shift of methylene A16′.

**Figure 4:**
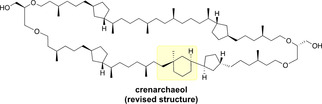
Revised chemical structure of natural crenarchaeol.

All in all this combined data provides overwhelming evidence for an inverted stereochemistry of crenarchaeol at A15′ compared to Fragment B. On the basis of the evidence from chemical derivatization, NMR studies, and computation, we therefore propose a revised structure of crenarchaeol (Figure 4), in which the stereochemistry of the *all*‐carbon quaternary stereocenter is inverted compared to the original proposal.

## Conclusion

The first total synthesis of the originally proposed structure of the thaumarchaeotal GDGT crenarchaeol has been achieved. The synthesis involved the stereoselective construction of a unique 5–6 ring motif as well as a late‐stage 66‐membered macrocyclization by means of RCM. The structure determination of crenarchaeol has a considerable history.^[11]^ Due to the very complex structure, including 22 stereocenters, as well as the highly aliphatic character and its lack of rigidity, NMR‐based structural studies have been heavily complicated. Furthermore, since this lipidic molecule does not have the tendency to crystallize, X‐ray diffraction was not possible. The synthesis of the proposed structure of crenarchaeol and the key intermediate Fragment B enabled direct comparison with natural crenarchaeol by chemical derivatization and GC–MS analysis. This revealed a mismatch of the chemical structure of the tricyclic biphytane chain. Subsequently, detailed NMR analysis including computational simulation of ^13^C chemical shifts, of Fragment B and synthetic crenarchaeol, and comparison with natural crenarchaeol isolated from sea surface sediments was performed. Ultimately, from the spectroscopic data of fragment B, synthetic and natural crenarchaeol, we were able to revise the originally proposed structure beyond reasonable doubt. Through this extensive analysis we identified the inversion of just one out of the 22 stereocenters of crenarchaeol, namely the quaternary stereocenter embedded in the cyclohexane ring.

Total synthesis not only comprises the access to complex molecules, but serves also as a breeding ground for new synthetic methodology as well as probing current synthetic methods. Mistakes in the proposed structure of a natural product are by no means a rare occurrence.^[33]^ The architectural and stereochemical complexity of a new unknown structure, in combination with very small amounts of isolated material often make assignments extremely difficult, in particular in a case such as crenarchaeol, which features almost no heteroatom functionalities and is highly flexible. By using the information gathered from the synthetic epimer of natural crenarchaeol, we were able to reassign the structure without the need to repeat the entire, very complex, synthesis.

The correction of the structure of crenarchaeol has important implications for the study of its role in archaeal membranes. The current hypothesis is that the presence of crenarchaeol regulates membrane fluidity and packing, an important adaptation to temperature and pressure changes in the environment. As the stereochemistry of the quaternary center in crenarchaeol has a significant influence on its conformation, and thus membrane packing, we expect that an explanation (supported by for instance molecular dynamics simulations) for its role in membrane behavior is now within reach.

## Conflict of interest

The authors declare no conflict of interest.

## Supporting information

As a service to our authors and readers, this journal provides supporting information supplied by the authors. Such materials are peer reviewed and may be re‐organized for online delivery, but are not copy‐edited or typeset. Technical support issues arising from supporting information (other than missing files) should be addressed to the authors.

Supporting Information
