# Supplementary material for: Total Synthesis of the Alleged Structure of Crenarchaeol Enables Structure Revision
Source: Angew Chem Int Ed Engl. 2021 Jun 30;60(32):17504–13. doi: 10.1002/anie.202105384 (PMC8361987; doi:10.1002/anie.202105384)
Supplement: Supplementary file 1 — Supporting Information [file ANIE-60-17504-s001.pdf]

Angewandte Chemie
Eine Zeitschrift der Gesellschaft Deutscher Chemiker

Supporting Information

Total Synthesis of the Alleged Structure of Crenarchaeol Enables Structure Revision**

*Mira Holzheimer, Jaap S. Sinninghe Damsté, Stefan Schouten, Remco W. A. Havenith, Ana V. Cunha, and Adriaan J. Minnaard**

anie_202105384_sm_miscellaneous_information.pdf

Table of Contents

General Methods and Materials 4

Synthetic Procedures and Analytical Data 5

Preparation of alkene 16 5

Epoxyketone S1 5
Epoxy tosylate S3 5
β-Hydroxy ketone 7 6
Acetal 15 8
Xanthate S4 8
Alkene 16 9

Preparation of Bromide 5 9

Alcohol 17 9
Alkene 16 from epi-17 12
Silylether 18 13
Ketone 19 14
Alkene 20 15
Alcohol 21 16
Bromide 5 16

Preparation of Dithiane 4 17

Thioester S10 17
Alcohol S11 19
Benzyl ether S13 20
Aldehyde S14 21
Dithiane 4 21

Dithiane Alkylation of Bromide 5 22

Dithiane 22 22

Preparation of Iodide 9 23

Preparation of Dithiane 6 24

Silylether S16 24
Alcohol S17 25
Iodide S18 26
Dithiane 6 26

Dithiane Alkylation of Iodide 9 27

Dithiane 3 27
Fragment A 28

Preparation of cyclopentene acetate 14 29

Diacetate 24 29
Monoacetate 25 29
Silylether 14 31

Preparation of Ligands L1 and L2 31

Diamine (±)-S21 31
^{1}H NMR (400 MHz, Chloroform-d) δ 7.37 – 7.27 (m, 4H), 7.19 – 7.10 (m, 4H), 4.06 – 3.99 (m, 2H), 2.69 – 2.63 (m, 2H), 1.44 (s, 4H). 32
Diamine S21 32
(R,R)-Phenyl-ANDEN-Trost L1 34
Bromide S25 35
(R)-t-ButylPHOX L2 36

Preparation of Ketones 27 and 28 37

2,2-Dimethylcyclohexanone 28 37
rac-ketone S27 37
(R)-2-allyl-2-methylcyclohexanone 13 38
Alcohol 26 40
Benzyl ether S29 41

Ketone 27 41
Palladium cataylzed intermolecular allylic alkylation **43**
Reaction optimization 43
Ketone 29 44
Ketone 30 45
Preparation of Diacetate 11 .... **47**
Three-step synthesis of diacetate 11 47
Acetate 31 50
Diacetate 11 51
Preparation of Grignard Reagent 32 .... **53**
MOM ether S34 53
Alcohol S35 54
Bromide S36 54
Grignard reagent 32 55
Preparation of Benzyl Ether 36 .... **55**
Alkene 33 55
MOM ether S37 57
Alcohol 35 58
Benzyl ether 36 58
Synthesis of dithiane 10 .... **59**
Alcohol 37 59
Thioester 39 60
Thioester 40 61
Alcohol S39 62
Dithiane 10 63
Dithiane 8 64
Fragment B 64
Preparation of glycerol building block 2 .... **65**
Diol S42 65
Trityl ether 2 66
Crenarchaeol assembly **67**
Mesylate 41 67
Tritylether 42 68
Alcohol 43 69
Bis-mesylate 44 69
Diol 45 70
Alkene 1 71
Alkene 46 72
Crenarchaeol 72

NMR analysis chemical shifts comparison of Fragment B, synthetic and natural crenarchaeol **74**

NMR analysis of Fragment B **74**
NMR analysis of synthetic crenarchaeol **80**
NMR analysis of natural isolated crenarchaeol **82**
^{1}H and ^{13}C NMR chemical shift comparison of Fragment B, natural and synthetic crenarchaeol **87**

Computational prediction of ^{13}C chemical shift values in natural and nominal crenarchaeol **92**

References **94**

General Methods and Materials

All reactions were performed using flame-dried glassware under N_2-atmosphere by Schlenk techniques, using anhydrous solvents (unless specified otherwise). Reaction temperatures refer to the temperature of the heating mantle or cooling bath.

Anhydrous solvents (MTBE, CH_2Cl_2, THF, toluene) were taken from a MBraun solvent purification system (SPS-800). Other anhydrous solvents were purchased from Sigma Aldrich or Acros Organics and used without further purification. Other reagents were purchased and used without further purification.

TLC analysis was performed on silica gel 60/Kieselguhr F_{254}, 0.25 mm (Merck). Compounds were visualized using elemental iodine followed by either Seebach stain or *p*-anis aldehyde stain.

^{1}H and ^{13}C NMR spectra were recorded on an Agilent 400 NMR spectrometer at 400 and 100.59 MHz, respectively, using $CDCl_3$ or CD_3OD as the solvent. Chemical shifts are reported in ppm with the solvent resonance as the internal standard (for $CDCl_3$: δ 7.26 ppm for ^{1}H, δ 77.16 ppm for ^{13}C, CD_3OD δ 3.31 ppm for ^{1}H, δ 49.00 ppm for ^{13}C). Data are reported as follows: chemical shifts (δ), multiplicity (s = singlet, d = doublet, dd = double doublet, ddd = double double doublet, ddp = double double pentet, td = triple doublet, t = triplet, q = quartet, b = broad, m = multiplet), coupling constant *J* (Hz), and integration value.

Enantiomeric excesses were determined by chiral HPLC analysis using a Shimadzu LC-10ADVP HPLC instrument equipped with a diode-array detector. Integration at three different wavelengths (254 nm and 220 nm) was performed and the reported enantiomeric excess is an average of the two integrations.

Enantiomeric excesses were determined by chiral GC analysis on an Agilent 7890A GC instrument equipped with a flame ionization detector.

High resolution mass spectra (HRMS) were recorded on a Thermo Scientific LTQ Orbitrap XL mass spectrometer with electron spray ionization (ESI) in positive or negative mode.

Optical rotations were measured on a polarimeter (Schmidt+Haensch Polartronic MH8) with a 10 cm long cell (c given in g/100 mL) at ambient temperature (±20 °C).

Synthetic Procedures and Analytical Data

Preparation of alkene 16

Epoxyketone S1

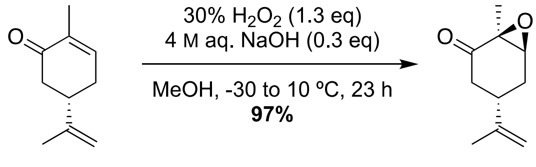

A flask was charged with (*S*)-carvone (10.6 mL, 10.2 g, 66.4 mmol, 1.0 eq., 98% purity) and placed under N_2 atmosphere by three cycles of evacuating and N_2 backfilling. The starting material was dissolved in 165 mL methanol and cooled to -30 °C (dry ice/acetone bath). Then 4 M aq. NaOH (5.0 mL, 20.0 mmol, 0.3 eq.) was added dropwise over 5 min followed by addition of aq. 30% H_2O_2 (8.2 mL, 86.6 mmol, 1.3 eq.) over 5 min. The mixture was stirred for 23 h and was allowed to warm up slowly to 10 °C. The reaction was quenched by addition of 10 mL 2 M aq. HCl followed by addition of 3 mL sat. aq. $Na_2S_2O_3$. The solution was transferred to a separatory funnel, 100 mL water and 100 mL brine were added and the aqueous layer was extracted with Et_2O (3 x 120 mL). The combined organic extracts were washed with brine (300 mL), dried over $MgSO_4$ and concentrated (25 mbar, 40 °C) to give the product **S1** (10.7 g, 64.1 mmol, 97% yield) as pale yellow liquid.

^{1}H NMR (400 MHz, Chloroform-*d*) δ 4.76 (t, *J* = 1.5 Hz, 1H), 4.69 (s, 1H), 3.42 (dd, *J* = 3.0, 1.4 Hz, 1H), 2.69 (tt, *J* = 10.8, 4.6 Hz, 1H), 2.55 (ddq, *J* = 17.6, 4.7, 1.6 Hz, 1H), 2.34 (dddd, *J* = 14.8, 4.6, 3.1, 1.6 Hz, 1H), 2.00 (ddt, *J* = 17.6, 11.6, 1.4 Hz, 1H), 1.92 – 1.83 (m, 1H), 1.68 (s, 3H), 1.38 (s, 3H).

HRMS (ESI+) $M+H^+$ calculated for $C_{10}H_{14}O_2$: 167.1067, found: 167.1065.

The analytical data is in agreement with previous reports.[1]

Epoxy tosylate S3

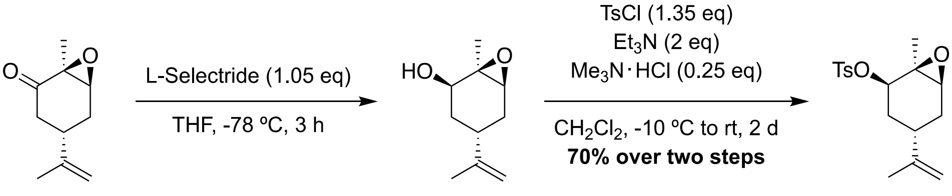

A Schlenk flask was flame-dried under vacuo and subjected to three cycles of evacuating and N_2 backfilling. The flask was charged with **S1** (4.99 g, 30.0 mmol, 1.0 eq.) dissolved in dry THF (200 mL) and cooled to -78 °C. 1 M L-Selectride in THF (31.5 mL, 31.5 mmol, 1.05 eq.) was added over 10 min. The reaction mixture was stirred for 3 h at -78 °C, then placed in an ice bath and quenched by slow addition of 5 mL water followed by addition of aq. 30% H_2O_2 (3.7 mL) and 2 M aq. NaOH (15 mL). The mixture was stirred for 1 h at 0 °C, then warmed to room temperature and stirred for 1 h. Water (150 mL) was added and the aqueous layer extracted with Et_2O (3 x 100 mL). The combined organic extracts were washed with brine (100 mL), dried over $MgSO_4$ and

concentrated to give the crude product **S2** (8.34 g) as pale yellow oil. The crude was used in the next step without purification.

HRMS (ESI+) M+H$^+$ calculated for $C_{10}H_{16}O_2$: 169.1223, found: 169.1223.

A 3-neck flask equipped with stirring bar and dropping funnel was flame-dried under vacuo and subjected to three cycles of evacuating and N_2 backfilling. $Me_3N{\cdot}HCl$ (438 mg, 4.58 mmol, 0.15 eq.) was added as solid under N_2 flow followed by addition of the crude alcohol **S2** (30.0 mmol, 1 eq.) dissolved in dry CH_2Cl_2 (30 mL) and dry Et_3N (8.34 mL, 59.8 mmol, 2 eq.). The solution was cooled to approx. -10 °C (NaCl/ice bath). TsCl (6.32 g, 33.1 mmol, 1.1 eq.) dissolved in dry CH_2Cl_2 (30 mL) was added dropwise over 1 h by means of a dropping funnel. After another 30 min stirring at -10 °C, the cooling bath was removed and the solution was stirred at room temperature for 22 h. The reaction was monitored by NMR sampling and when still starting material was detected, $Me_3N{\cdot}HCl$ (285 mg, 2.98 mmol, 0.1 eq.) was added. Then the mixture was again cooled to -10 °C and TsCl (1.45 g, 7.62 mmol, 0.25 eq.) dissolved in dry CH_2Cl_2 (7.5 mL) was added over 1 h. The reaction was stirred for another hour at -10 °C and then for 19 h at room temperature. The reaction mixture was diluted with CH_2Cl_2 (100 mL), washed 1 M aq. HCl (2 x 200 mL), brine (200 mL), dried over $MgSO_4$ and concentrated. The crude product was purified by flash chromatography (pentane/Et_2O 8/2) to give the product **S3** (6.74 g, 20.9 mmol, 70% yield over two steps).

^{1}H NMR (400 MHz, Chloroform-*d*) δ 7.82 (dd, *J* = 8.4, 1.7 Hz, 2H), 7.34 (d, *J* = 6.8 Hz, 2H), 4.86 – 4.82 (m, 1H), 4.80 (s, 1H), 4.61 (s, 1H), 3.15 – 3.09 (m, 1H), 2.45 (s, 3H), 2.35 – 2.26 (m, 1H), 2.07 (dd, *J* = 15.3, 5.4 Hz, 1H), 1.84 – 1.75 (m, 2H), 1.66 (s, 3H), 1.58 (ddd, *J* = 14.2, 9.0, 5.6 Hz, 1H), 1.21 (s, 3H).

^{13}C NMR (101 MHz, Chloroform-*d*) δ 146.78, 144.85, 134.47, 129.91, 127.95, 110.27, 79.96, 60.93, 56.95, 34.69, 31.15, 28.75, 21.79, 21.54, 20.36.

HRMS (ESI+) M+H$^+$ calculated for $C_{17}H_{22}O_4S$: 323.1312, found: 323.1311.

HRMS (ESI+) M+Na$^+$ calculated for $C_{17}H_{22}O_4S$: 345.1131, found: 345.1125.

HRMS (ESI+) M+NH_4^+ calculated for $C_{17}H_{22}O_4S$: 340.1577, found: 340.1579.

Melting point: 61 °C.

Optical Rotation: $[\alpha]_D^{23}$ = +39.7° (*c* = 1.42, $CHCl_3$)

The analytical data is in agreement with previous reports.[1]

β-Hydroxy ketone 7

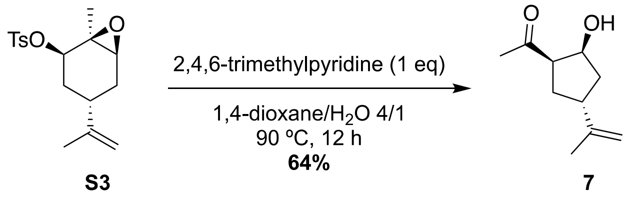

A flask was charged with **S3** (6.40 g, 19.9 mmol, 1.0 eq.), 1,4-dioxane (160 mL), and water (40 mL). 2,4,6-trimethylpyridine (2.7 mL, 2.46 g, 19.9 mmol, 1.0 eq.) was added and the solution was heated to 90 °C and stirred for 12 h. The reaction mixture was allowed to cool to room temperature and then brine (200 mL) was added. The layers

were separated and the aqueous layer was extracted with Et_2O (2 x 200 mL). The combined organic phases were washed with sat. aq. $CuSO_4$ (2 x 150 mL), water (200 mL) and brine (200 mL), dried over $MgSO_4$ and concentrated. The crude was purified by flash chromatography (pentane/Et_2O 1/1) to give the product **7** (2.15 g, 12.8 mmol, 64% yield) as pale yellow oil.

^{1}H NMR (400 MHz, Chloroform-*d*) δ 4.73 – 4.68 (m, 2H), 4.56 (q, *J* = 3.9 Hz, 1H), 3.12 (d, *J* = 3.2 Hz, 1H), 3.05 – 2.98 (m, 1H), 2.95 (dd, *J* = 9.6, 4.0 Hz, 1H), 2.29 – 2.21 (m, 1H), 2.20 (s, 3H), 1.92 (ddd, *J* = 13.3, 7.6, 1.4 Hz, 1H), 1.79 (ddd, *J* = 13.2, 10.0, 6.7 Hz, 1H), 1.73 (s, 3H), 1.65 (ddd, *J* = 13.5, 10.4, 4.3 Hz, 1H).

^{13}C NMR (101 MHz, Chloroform-*d*) δ 211.45, 147.84, 108.85, 74.14, 56.34, 43.05, 40.18, 30.38, 30.36, 21.13.

HRMS (ESI+) $M+H^+$ calculated for $C_{10}H_{16}O_2$: 169.1223, found: 169.1222.

HRMS (ESI+) $M+Na^+$ calculated for $C_{10}H_{16}O_2$: 191.1043, found: 191.1041.

Optical Rotation: $[\alpha]_D^{23}$ = +67.6° (*c* = 0.46, $CHCl_3$)

The analytical data is in agreement with previous reports.[2]

<u>NOESY NMR analysis of **7**</u>

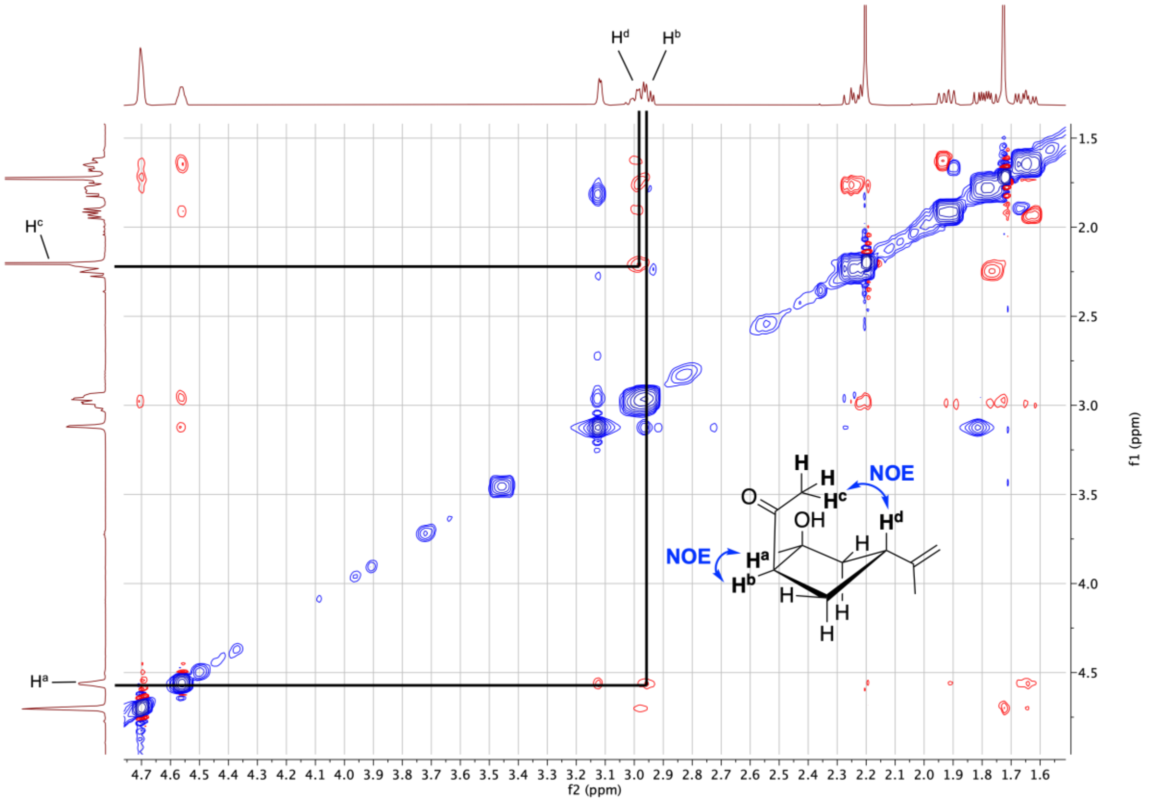

Acetal 15

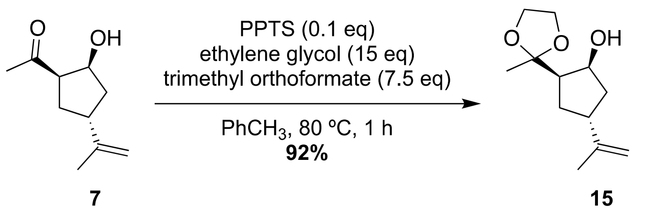

A flask was placed under N_2 atmosphere by three cycles of evacuating and N_2 backfilling, then charged with **7** (2.02 g, 12.0 mmol, 1.0 eq.) and dissolved in dry toluene (48 mL). PPTS (301 mg, 1.20 mmol, 0.1 eq.), ethylene glycol (10.0 mL, 11.1 g, 178.84 mmol, 15 eq.) trimethyl orthoformate (9.8 mL, 9.51 g, 89.6 mmol, 7.5 eq.) were added and the reaction mixture was stirred at 80 °C for 1 h. After cooling to room temperature water (75 mL) was added and the aqueous phase was extracted with Et_2O (3 x 70 mL). The combined organic extracts were washed with brine (150 mL), dried over $MgSO_4$, and concentrated. The crude was purified by flash chromatography (pentane/Et_2O 2/1) to give the product **15** (2.34 g, 11.0 mmol, 92% yield) as pale yellow oil.

^{1}H NMR (400 MHz, Chloroform-*d*) δ 4.71 (s, 1H), 4.66 (s, 1H), 4.38 (s, 1H), 4.08 – 3.91 (m, 4H), 3.54 (s, 1H), 2.97 (td, *J* = 10.8, 5.4 Hz, 1H), 2.32 (td, *J* = 10.0, 3.6 Hz, 1H), 2.03 – 1.90 (m, 2H), 1.72 (s, 3H), 1.64 (ddd, *J* = 13.3, 9.6, 5.8 Hz, 1H), 1.53 (ddd, *J* = 13.6, 10.5, 3.6 Hz, 1H), 1.39 (s, 3H).

^{13}C NMR (101 MHz, Chloroform-*d*) δ 148.87, 111.00, 108.39, 74.12, 65.55, 63.93, 51.99, 42.71, 40.68, 28.13, 24.07, 20.86.

HRMS (ESI+) M+Na$^+$ calculated for $C_{12}H_{20}O_3$: 235.1305, found: 235.1310.

Optical Rotation: $[\alpha]_D^{23}$ = -3.7° (*c* = 0.67, $CHCl_3$).

Xanthate S4

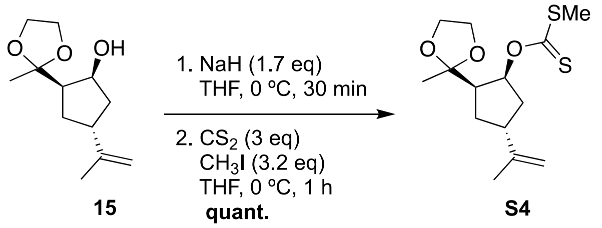

A Schlenk flask was flame-dried under vacuo and subjected to three cycles of evacuating and N_2 backfilling. The flask was charged with solid NaH (678 mg, 17.0 mmol, 1.7 eq., 60% in mineral oil), suspended in dry THF (20 mL) and cooled to 0 °C (ice bath). To the stirred suspension was added **15** (2.12 g, 10.0 mmol, 1.0 eq.) and after stirring for 30 min at 0 °C, CS_2 (1.78 mL, 2.27 g, 29.8 mmol, 3.0 eq.) was added. After an additional 5 min stirring at 0 °C, iodomethane (2.3 mL, 4.56 g, 32.1 mmol, 3.2 eq.) was added and the resulting mixture was stirred for 1 h at 0 °C. The reaction was quenched by careful addition of sat. aq. NH_4Cl (20 mL), warmed to room temperature and the aqueous phase was extracted with Et_2O (3 x 15 mL). The combined organic extracts were washed with water (15 mL) and brine (15 mL), dried over $MgSO_4$ and concentrated to give the product **S4** (3.01 g, 9.95 mmol, quant. yield) which was used without further purification.

^1H NMR (400 MHz, Chloroform-*d*) δ 6.12 (t, *J* = 4.2 Hz, 1H), 4.73 – 4.68 (m, 2H), 3.98 (dddd, *J* = 7.5, 5.3, 4.6, 1.8 Hz, 1H), 3.95 – 3.88 (m, 3H), 2.88 (ddd, *J* = 17.1, 10.4, 6.6 Hz, 1H), 2.56 (s, 3H), 2.51 (td, *J* = 10.0, 4.4 Hz, 1H), 2.24 – 2.10 (m, 2H), 1.85 – 1.76 (m, 2H), 1.73 (s, 3H), 1.33 (s, 3H), 0.90 – 0.81 (m, 1H).

^{13}C NMR (101 MHz, Chloroform-*d*) δ 214.67, 147.87, 109.75, 108.90, 86.35, 65.42, 64.30, 51.86, 42.79, 37.81, 29.58, 23.85, 20.95, 18.64.

HRMS (ESI+) M+Na$^+$ calculated for $C_{14}H_{22}O_3S_2$: 325.0903, found: 325.0905.

Alkene 16

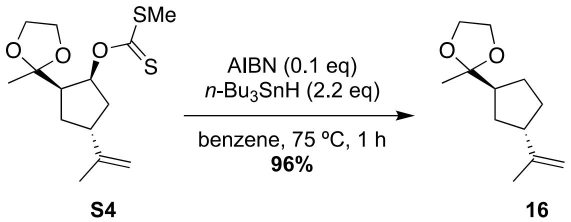

A flask was placed under N_2 atmosphere by three cycles of evacuating and N_2 backfilling, charged with **S4** (3.0 g, 9.9 mmol, 1.0 eq.) and AIBN (166 mg, 0.99 mmol, 0.1 eq.). Dry benzene (67 mL) was added and to the resulting solution was added *n*-Bu_3SnH (6.0 mL, 6.49 g, 21.6 mmol, 2.2 eq.). The reaction was stirred for 1 h at 75 °C. After cooling to room temperature, Et_2O (100 mL) was added and the organic layer was washed with 2 M aq. NaOH (2 x 100 mL), brine (50 mL), dried over $MgSO_4$ and concentrated. The crude was purified by flash chromatography (pentane/Et_2O 95/5) to give the product **16** (1.87 g, 9.47 mmol, 96% yield) as pale yellow liquid.

^1H NMR (400 MHz, Chloroform-*d*) δ 4.67 (d, *J* = 11.1 Hz, 1H), 4.03 – 3.86 (m, 4H), 2.49 (p, *J* = 8.5 Hz, 1H), 2.33 (td, *J* = 9.6, 4.6 Hz, 1H), 1.82 (dt, *J* = 12.7, 6.3 Hz, 2H), 1.77 – 1.69 (m, 4H), 1.62 – 1.53 (m, 1H), 1.52 – 1.38 (m, 2H), 1.28 (s, 3H).

^{13}C NMR (101 MHz, Chloroform-*d*) δ 148.98, 112.04, 108.16, 64.97, 64.93, 47.16, 46.62, 32.03, 31.94, 28.20, 22.56, 21.42.

HRMS (ESI+) M+H$^+$ calculated for $C_{12}H_{20}O_2$: 197.1536, found: 197.1548.

Optical Rotation: $[\alpha]_D^{23}$ = -3.3° (*c* = 0.58, $CHCl_3$).

Preparation of Bromide 5

Alcohol 17

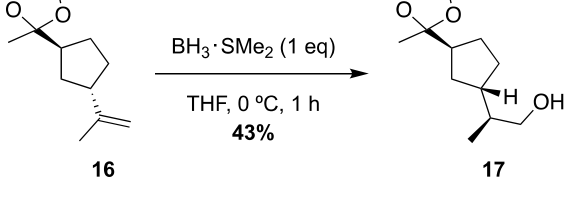

A Schlenk flask was flame-dried under vacuo and subjected to three cycles of evacuating and N_2 backfilling. The flask was charged with **16** (3.03 g, 15.3 mmol, 1 eq.) dissolved in dry THF (75 mL) and cooled to 0 °C. 2 M BH_3·SMe_2 in THF (7.64 mL, 15.3 mmol, 1 eq.) was dropwise over 5 min and the resulting solution was stirred for 2 h at

0 °C. The reaction was quenched by careful addition of 2 M aq. NaOH (38 mL, 76 mmol, 5.0 eq). The ice bath was removed and aq. 30% H_2O_2 (14.5 mL, 153 mmol, 10.0 eq.) was added. The reaction mixture was stirred for 16 h at room temperature. Water (100 mL) was added and the aqueous phase was extracted with Et_2O (3 x 80 mL). The combined organic extracts were washed with water (150 mL) and brine (80 mL), dried over $MgSO_4$ and concentrated. The crude was purified by flash chromatography (Et_2O/pentane 2/1) to give the product **17** (1.43 g, 6.58 mmol, 43% yield) as colourless oil. The product was obtained as single diastereoisomer.

^{1}H NMR (400 MHz, Chloroform-*d*) δ 3.99 – 3.89 (m, 4H), 3.63 (dd, *J* = 10.6, 4.5 Hz, 1H), 3.40 (dd, *J* = 10.5, 7.1 Hz, 1H), 2.25 (dddd, *J* = 12.9, 10.0, 5.8, 3.4 Hz, 1H), 1.86 – 1.66 (m, 4H), 1.48 – 1.33 (m, 4H), 1.26 (s, 3H), 1.20 – 1.09 (m, 1H), 0.94 (d, *J* = 6.7 Hz, 3H).

^{13}C NMR (101 MHz, Chloroform-*d*) δ 112.05, 67.53, 64.95, 64.90, 47.04, 42.36, 41.26, 31.93, 31.10, 28.53, 22.45, 15.49.

HRMS (ESI+) M+H$^+$ calculated for $C_{12}H_{22}O_3$: 215.1642, found: 215.1644.

Optical Rotation: $[\alpha]_D^{23}$ = -1.7° (*c* = 0.58, $CHCl_3$).

Synthesis of PGME amides S6 and *epi*-S6

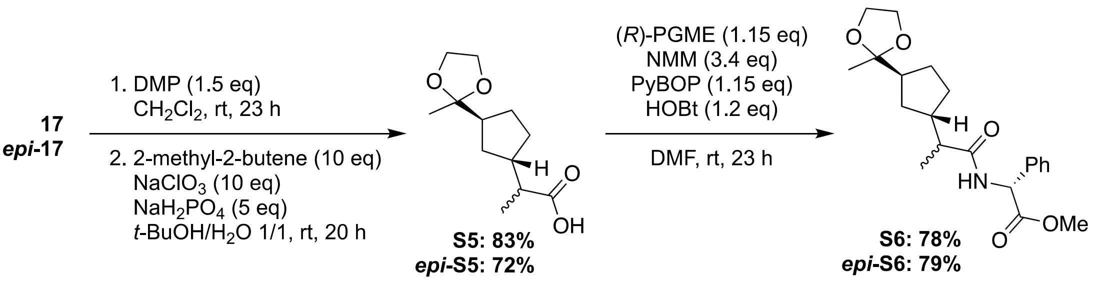

A flask was charged with **17** (28.1 mg, 0.13 mmol, 1 eq.) and Dess-Martin periodinane (85.6 mg, 0.20 mmol, 1.5 eq). Dry CH_2Cl_2 (1.3 mL) was added and the reaction mixture was stirred for 23 h at rt. The suspension was then diluted with CH_2Cl_2 and washed with sat. aq. NaHCO3 (2 x 5 mL). The organic layer was then dried over $MgSO_4$ and concentrated. And the crude aldehyde was used without further purification in the next step.

A flask was charged with crude aldehyde and *t*-BuOH/H2O 1/1 (5.2 mL) was added. To this, 2-methyl-2-butene (0.14 mL, 1.32 mmol, 10 eq.), $NaHPO_4{\cdot}2H_2O$ (102 mg, 0.65 mmol, 5 eq.) and $NaClO_2$ (120 mg, 1.33 mmol, 10.1 eq.) was added. The reaction mixture was stirred for 20 h at rt. Sat. aq. NH_4Cl (10 mL) was added and the aqueous phase was extracted with CH_2Cl_2 (3 x 10 mL). The combined organic extracts were dried over $MgSO_4$ and concentrated. The crude was purified by flash chromatography (pentane/EtOAc 1/1) to give the product **S5** (24.7 mg, 0.11 mmol, 83% yield) as colorless oil.

^{1}H NMR (400 MHz, Chloroform-*d*) δ 3.99 – 3.86 (m, 4H), 2.34 – 2.19 (m, 2H), 2.16 – 2.04 (m, 1H), 1.81 (dtt, *J* = 35.4, 15.0, 6.9 Hz, 3H), 1.51 – 1.29 (m, 3H), 1.27 – 1.21 (m, 4H), 1.17 (d, *J* = 7.0 Hz, 3H).

HRMS (ESI+) M+H$^+$ calculated for $C_{12}H_{20}O_4$: 227.1278, found: 227.1291.

A flask was charged with carboxylic acid **S5** (24.5 mg, 0.11 mmol, 1 eq.) and dissolved in dry DMF (0.55 mL). To this, (*R*)-phenylglycine methyl ester HCl salt (24.9 mg, 0.12 mmol, 1.15 eq.) was added. The resulting solution was cooled to 0 ºC and PyBOP (64.6 mg, 0.12 mmol, 1.2 eq.), HOBt (18 mg, 0.13 mmol, 1.2 eq.) and *N*-methylmorpholine (40 µL, 0.36 mmol, 3.4 eq.) were added. After stirring for 5 min at 0 ºC, the reaction mixture was stirred for 23 h at rt. The reaction was diluted with EtOAc (15 mL), washed with 2 M aq. HCl (2 x 10 mL), sat. aq. $NaHCO_3$ (10 mL) and brine (10 mL). The organic layer was dried over $MgSO_4$ and concentrated. The crude was purified by flash chromatography (pentane/EtOAc 1/1) to give amide **S6** (36.5 mg, 0.08 mmol, 78% yield) as colourless oil.

^{1}H NMR (400 MHz, Chloroform-*d*) δ 7.35 – 7.29 (m, 5H), 6.45 (d, *J* = 7.5 Hz, 1H), 5.57 (d, *J* = 7.0 Hz, 1H), 4.00 – 3.81 (m, 4H), 3.71 (s, 3H), 2.28 – 2.16 (m, 1H), 2.06 – 1.98 (m, 2H), 1.79 – 1.57 (m, 3H), 1.44 – 1.27 (m, 2H), 1.23 (s, 3H), 1.15 (d, *J* = 5.7 Hz, 3H), 1.11 – 1.01 (m, 1H).

^{13}C NMR (101 MHz, Chloroform-*d*) δ 175.63, 171.62, 136.80, 129.01, 128.51, 127.34, 111.76, 64.90, 64.84, 56.27, 52.83, 47.13, 46.96, 43.47, 32.03, 31.27, 28.08, 22.38, 16.78.

HRMS (ESI+) M+H$^+$ calculated for $C_{21}H_{29}NO_5$: 376.2119, found: 376.2122.

Synthesis of ***epi*-S5** and ***epi*-S6** from ***epi*-17** was performed in analogy in 72% (2 steps) and 79% yield, respectively.

Analytical data ***epi*-S5**:

^{1}H NMR (400 MHz, Chloroform-*d*) δ 3.99 – 3.88 (m, 4H), 2.35 – 2.21 (m, 2H), 2.13 – 2.04 (m, 1H), 1.87 (dq, *J* = 12.9, 6.8, 6.4 Hz, 1H), 1.78 (dtd, *J* = 16.0, 8.8, 8.3, 4.1 Hz, 2H), 1.50 – 1.37 (m, 2H), 1.26 (s, 3H), 1.22 – 1.12 (m, 4H).

^{13}C NMR (101 MHz, Chloroform-*d*) δ 182.78, 111.81, 64.95, 64.90, 47.31, 45.06, 42.38, 31.71, 31.15, 27.99, 21.46, 15.94.

HRMS (ESI+) M+H$^+$ calculated for $C_{12}H_{20}O_4$: 227.1278, found: 227.1292.

Analytical data ***epi*-S6**:

^{1}H NMR (400 MHz, Chloroform-*d*) δ 7.38 – 7.29 (m, 5H), 6.44 (d, *J* = 7.3 Hz, 1H), 5.55 (d, *J* = 7.2 Hz, 1H), 3.95 – 3.85 (m, 4H), 3.70 (s, 3H), 2.25 (td, *J* = 10.0, 5.0 Hz, 1H), 2.08 – 2.00 (m, 2H), 1.91 – 1.82 (m, 1H), 1.81 – 1.68 (m, 2H), 1.49 – 1.31 (m, 2H), 1.23 (s, 3H), 1.15 – 1.11 (m, 1H), 1.10 (d, *J* = 6.0 Hz, 3H).

^{13}C NMR (101 MHz, Chloroform-*d*) δ 175.56, 171.47, 136.84, 128.99, 128.48, 127.27, 111.71, 64.90, 56.23, 52.77, 47.30, 47.14, 42.97, 31.74, 31.43, 27.96, 22.58, 16.62.

HRMS (ESI+) M+H$^+$ calculated for $C_{21}H_{29}NO_5$: 376.21185, found: 376.21217.

Comparison of ^{1}H NMR spectra for stereochemistry elucidation

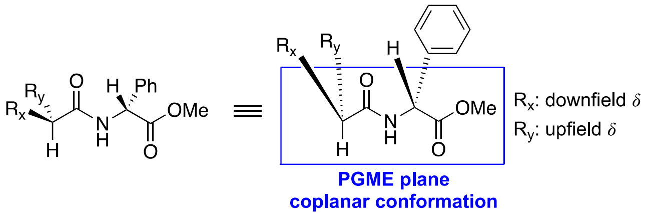

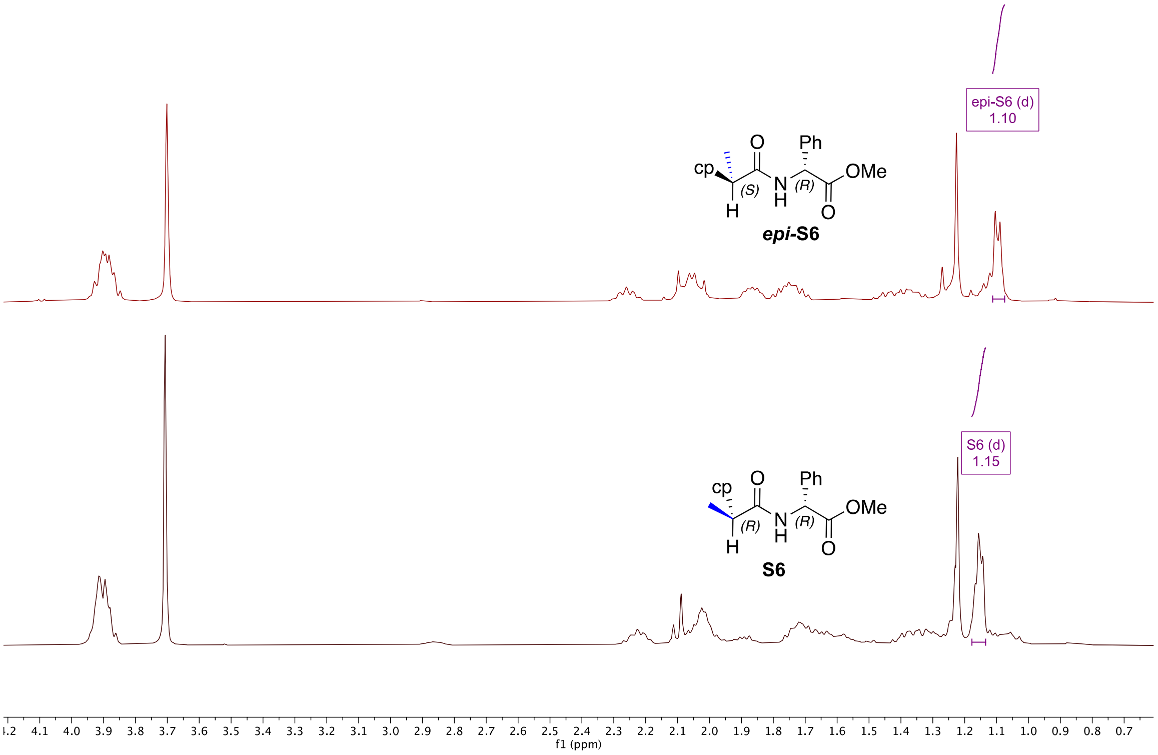

Alkene 16 from *epi*-17

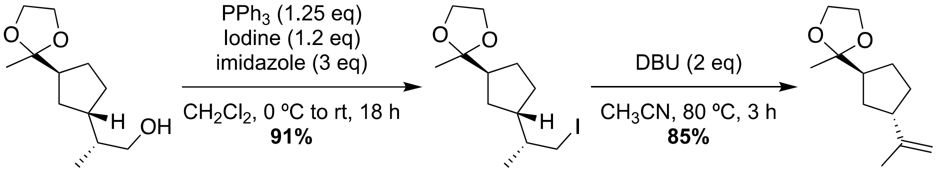

***epi*-17** **S7** **16**

A flask was placed under N_2 atmosphere by three cycles of evacuating and N_2 backfilling and charged with solid imidazole (3.29 g, 48.4 mmol, 3.0 eq.) and PPh_3 (5.28 g, 20.1 mmol, 1.25 eq.) under N_2 flow followed by addition of alcohol ***epi*-17** (3.45 g, 16.1 mmol, 1.0 eq.) dissolved in dry CH_2Cl_2 (32 mL). The solution was cooled to 0 ºC and iodine (4.90 g, 19.3 mmol, 1.2 eq.) was added as solid in one portion under N_2 flow. The resulting mixture was stirred for 18 h and allowed to warm to room temperature. The solvent was evaporated and the crude was purified by flash chromatography (pentane/Et_2O 96/4 to 9/1) to give the product **S7** (4.78 g, 14.7 mmol, 91%) yield) as colourless liquid.

^{1}H NMR (400 MHz, Chloroform-*d*) δ 3.99 – 3.89 (m, 4H), 3.32 (dd, *J* = 9.6, 3.1 Hz, 1H), 3.14 (dd, *J* = 9.6, 6.6 Hz, 1H), 2.33 – 2.22 (m, 1H), 1.90 – 1.66 (m, 4H), 1.49 – 1.37 (m, 1H), 1.31 – 1.22 (m, 4H), 1.21 – 1.10 (m, 2H), 0.97 (d, *J* = 6.5 Hz, 3H).

^{13}C NMR (101 MHz, Chloroform-*d*) δ 111.91, 64.99, 64.90, 47.25, 45.28, 40.50, 31.69, 31.57, 28.41, 22.38, 19.53, 18.13.

A flask was charged with iodide **S7** (4.78 g, 14.7 mmol, 1 eq.) and dissolved in acetonitrile (30 mL). To the solution was added DBU (4.4 mL, 29.5 mmol, 2 eq.) and the mixture was heated to 80 °C and stirred for 3 h. After cooling to room temperature, the solvent was evaporated and the crude was purified by flash chromatography (pentane/Et_2O 9/1) to give the product **16** (2.44 g, 12.5 mmol, 85% yield) as colourless liquid.

Silylether 18

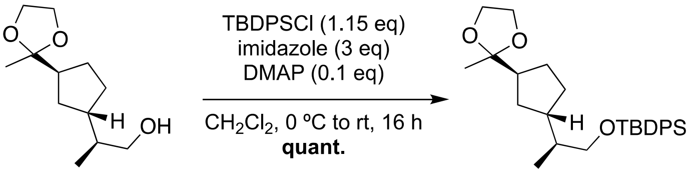

A flask was placed under N_2 atmosphere by three cycles of evacuating and N_2 backfilling, then charged with imidazole (1.71 g, 25.1 mmol, 3.0 eq.), DMAP (105 mg, 0.86 mmol, 0.1 eq.) and **17** (1.78 g, 8.32 mmol, 1.0 eq.) dissolved in dry CH_2Cl_2 (42 mL). The solution was cooled to 0 °C and TBDPSCl (2.50 mL, 9.57 mmol, 1.15 eq.) was added. After 5 min stirring at 0 °C, the ice bath was removed and the reaction mixture was stirred at room temperature for 16 h. The reaction was quenched by addition of sat. aq. NH_4Cl (75 mL) and the layers were separated. The aqueous phase was extracted with Et_2O (3 x 60 mL) and the combined organic phases were washed with water (75 mL) and brine (75 mL), dried over $MgSO_4$ and concentrated. The crude was purified by flash chromatography (pentane 100% to pentane/Et_2O 95/5) to give the product **18** (3.79 g, 8.30 mmol, quant. yield) as colourless oil.

^1H NMR (400 MHz, Chloroform-*d*) δ 7.68 (dt, *J* = 7.8, 1.5 Hz, 4H), 7.46 – 7.35 (m, 6H), 3.99 – 3.89 (m, 4H), 3.64 (dd, *J* = 9.8, 4.5 Hz, 1H), 3.44 (dd, *J* = 9.8, 7.0 Hz, 1H), 2.22 (ddt, *J* = 10.3, 7.8, 4.7 Hz, 1H), 1.86 – 1.63 (m, 4H), 1.55 – 1.46 (m, 1H), 1.45 – 1.31 (m, 2H), 1.27 (s, 3H), 1.12 – 1.04 (m, 10H), 0.99 (d, *J* = 6.7 Hz, 3H).

^{13}C NMR (101 MHz, Chloroform-*d*) δ 135.77, 134.29, 134.27, 129.60, 127.68, 112.12, 68.25, 64.94, 64.90, 47.11, 42.33, 41.31, 31.91, 31.08, 28.52, 27.03, 22.44, 19.47, 15.82.

HRMS (ESI+) $M+H^+$ calculated for $C_{28}H_{40}O_3Si$: 453.2820, found: 453.2819.

Optical Rotation: $[\alpha]_D^{23}$ = -2.2° (*c* = 0.50, $CHCl_3$).

Optimization of acetal deprotection of **18**

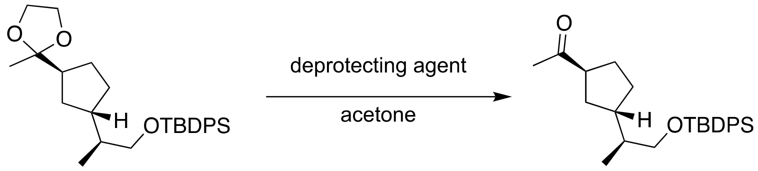

18 **19**

Entry	Reagent	Temperature	Time	Yield[a]	*dr*[b]
**1**	PPTS (0.5 eq)	60 ºC	5.5 h	97%	88:12<br>82:18[c]
**2**	I_2 (0.1 eq)	rt	1.5 h	quant.	80:20
**3**	$FeCl_3 \cdot SiO_2$ (12.5 wt.%)	rt	12 min[d]	quant.	97:3

[a] Isolated yield. [b] Diastereomeric ratio after workup, determined by ^{13}C NMR integration (desired isomer at 42.2 ppm and epimer at 43.3 ppm). [c] Diastereomeric ratio determined after silica column. [d] Optimal reaction time for 1 g reaction scale.

Ketone 19

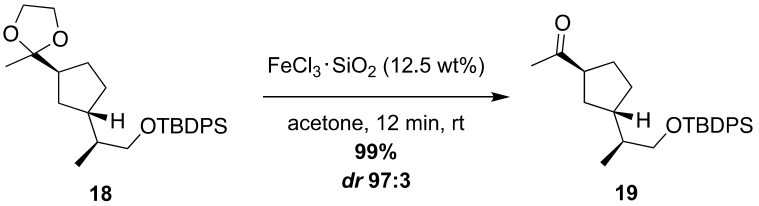

18 **19**

In a flask, **18** (1.0 g, 2.21 mmol, 1.0 eq.) was dissolved in acetone (15 mL). To the solution was added of $FeCl_3$ adsorbed on silica[3] (128 mg, 12.5 wt%). The suspension was stirred for 12 min. Brine (30 mL) was added and the aqueous phase was extracted with Et_2O (3 x 50 mL). The combined organic extracts were washed with water (10 mL), dried over $MgSO_4$ and concentrated to give the product **19** (907 mg, 2.22 mmol, quant. yield) as pale yellow oil.

The degree of epimerization was determined by ^{13}C NMR: major 42.33 ppm, minor 43.40 ppm, *dr* = 97/3.

^{1}H NMR (400 MHz, Chloroform-*d*) δ 7.69 – 7.64 (m, 4H), 7.45 – 7.35 (m, 6H), 3.62 (dd, *J* = 9.9, 4.5 Hz, 1H), 3.44 (dd, *J* = 9.9, 6.7 Hz, 1H), 2.87 (dtd, *J* = 10.1, 8.3, 5.0 Hz, 1H), 2.14 (s, 3H), 2.03 – 1.95 (m, 1H), 1.89 (dtd, *J* = 12.7, 7.9, 1.8 Hz, 1H), 1.81 – 1.72 (m, 2H), 1.68 – 1.60 (m, 1H), 1.53 – 1.47 (m, 1H), 1.45 – 1.38 (m, 1H), 1.18 – 1.09 (m, 1H), 1.06 (s, 9H), 0.99 (d, *J* = 6.7 Hz, 3H).

^{13}C NMR (101 MHz, Chloroform-*d*) δ 211.14, 135.76, 135.75, 134.14, 129.65, 127.72, 127.71, 68.17, 51.37, 42.33, 40.99, 32.12, 31.62, 29.04, 28.74, 27.02, 19.45, 15.83.

HRMS (ESI+) M+Na+ calculated for $C_{26}H_{36}O_3Si$: 431.2377, found: 431.2374.

Note: as reliable reaction monitoring by TLC was impossible due to virtually identical R_f values of starting material and product, the reaction time was optimized for a scale of 1 g of acetal **18**.

Alkene 20

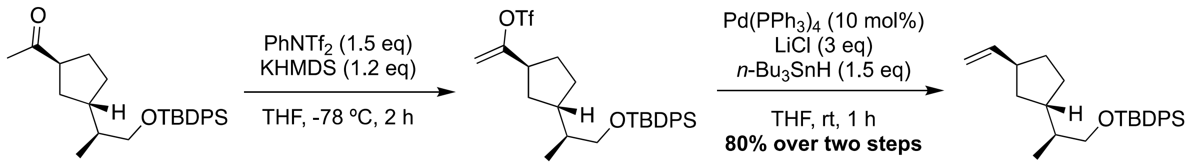

19 **S8** **20**

A Schlenk flask was flame-dried under vacuo and subjected to three cycles of evacuating and N_2 backfilling. The flask was charged with $PhNTf_2$ (1.16 g, 3.27 mmol, 1.5 eq.) as solid under N_2 flow. Then **19** (887 mg, 2.17 mmol, 1.0 eq.) dissolved in dry THF (11 mL) was added and the resulting solution was cooled to -78 °C. 1 M KHMDS in THF (2.6 mL, 2.6 mmol, 1.2 eq.) was added dropwise and the reaction mixture was stirred for 2 h at -78 °C. The reaction was quenched by addition of sat. aq. NH_4Cl (15 mL) and allowed to come to room temperature. The aqueous phase was extracted Et_2O (3 x 15 mL). The combined organic extracts were washed with brine (10 mL), dried over $MgSO_4$ and concentrated. The crude was purified by flash chromatography (pentane/Et_2O 99.5/0.5 to 99.2/0.8 to 99/1) to give the product (1.20 g) contaminated with $PhNTf_2$ residues. The impure enol triflate **S8** was used without further purification in the next step.

HRMS (ESI+) $M+H^+$ calculated for $C_{27}H_{35}F_3O_4SSi$: 541.2050, found: 541.2054.

HRMS (ESI+) $M+Na^+$ calculated for $C_{27}H_{35}F_3O_4SSi$: 563.1870, found: 563.1878.

A Schlenk flask was flame-dried under vacuo and subjected to three cycles of evacuating and N_2 backfilling. The flask was charged with solid LiCl (279 mg, 6.58 mmol, 3 eq.) and $Pd(PPh_3)_4$ (253 mg, 0.22 mmol, 0.1 eq.) under N_2 flow, **S8** (1.20 g, contaminated) dissolved in dry THF (43 mL) and *n*-Bu_3SnH (0.88 mL, 3.27 mmol, 1.5 eq.) and the reaction mixture was stirred at room temperature for 1 h. The reaction was diluted with Et_2O (250 mL), washed 2 M aq. NaOH (2 x 75 mL) and brine (50 mL), dried over $MgSO_4$ and concentrated. The crude was purified by flash chromatography (pentane/Et_2O 99.7/0.3 to 99.5/0.5) to give the product **20** (679 mg, 1.73 mmol, 80% yield over two steps) as colourless oil.

^{1}H NMR (400 MHz, Chloroform-*d*) δ 7.68 (d, *J* = 7.7 Hz, 4H), 7.46 – 7.35 (m, 6H), 5.79 (ddd, *J* = 17.4, 9.9, 7.6 Hz, 1H), 4.94 (d, *J* = 17.2 Hz, 1H), 4.86 (d, *J* = 9.0 Hz, 1H), 3.62 (dd, *J* = 9.8, 4.6 Hz, 1H), 3.44 (dd, *J* = 9.7, 7.0 Hz, 1H), 2.49 (h, *J* = 7.6 Hz, 1H), 1.91 – 1.68 (m, 3H), 1.57 – 1.47 (m, 3H), 1.30 (tdd, *J* = 11.6, 8.7, 6.9 Hz, 1H), 1.17 – 1.09 (m, 1H), 1.08 – 1.00 (m, 10H), 0.98 (d, *J* = 6.7 Hz, 3H).

^{13}C NMR (101 MHz, Chloroform-*d*) δ 144.22, 135.77, 134.25, 134.22, 129.62, 127.70, 111.98, 68.22, 43.05, 41.37, 41.29, 35.97, 33.24, 31.28, 27.03, 19.47, 15.73.

HRMS (ESI+) $M+H^+$ calculated for $C_{26}H_{36}OSi$: 393.2608, found: 393.2610.

Optical Rotation: $[\alpha]_D^{23}$ = -8.8° (*c* = 0.54, $CHCl_3$).

Alcohol 21

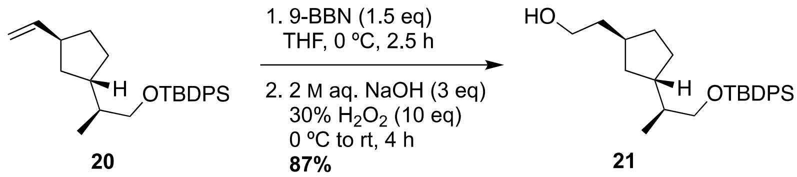

A Schlenk flask was flame-dried under vacuo and subjected to three cycles of evacuating and N_2 backfilling. The flask was charged with **20** (612 mg, 1.56 mmol, 1.0 eq.) dissolved in dry THF (15 mL) and cooled to 0 °C. 0.5 M 9-BBN in THF (4.7 mL, 2.35 mmol, 1.5 eq.) was added dropwise. The reaction mixture was stirred for 2.5 h at 0 °C. The reaction was quenched by addition of 2 M aq. NaOH (2.3 mL, 4.6 mmol, 3 eq.) followed by aq. 30% H_2O_2 (1.5 mL, 17.6 mmol, 10 eq.). The reaction was allowed to come to room temperature and stirred for 4 h. The reaction was diluted with sat. aq. NH_4Cl (15 mL) and water (10 mL). The aqueous phase was extracted with Et_2O (3 x 30 mL) and the combined organic extracts were washed with brine (20 mL), dried over $MgSO_4$ and concentrated. The crude product was purified by flash chromatography (pentane/Et_2O 5/2) to give the product **21** (554 mg, 1.35 mmol, 87% yield) as colourless oil.

^{1}H NMR (400 MHz, Chloroform-*d*) δ 7.71 – 7.62 (m, 4H), 7.45 – 7.34 (m, 6H), 3.67 – 3.57 (m, 3H), 3.44 (dd, *J* = 9.8, 6.8 Hz, 1H), 1.97 – 1.86 (m, 1H), 1.85 – 1.75 (m, 2H), 1.72 – 1.65 (m, 1H), 1.56 (q, *J* = 7.6, 7.0, 1.3 Hz, 2H), 1.51 – 1.46 (m, 1H), 1.46 – 1.40 (m, 1H), 1.38 – 1.32 (m, 2H), 1.10 – 1.07 (m, 1H), 1.05 (s, 9H), 0.97 (d, *J* = 6.7 Hz, 3H).

^{13}C NMR (101 MHz, Chloroform-*d*) δ 135.78, 135.77, 134.30, 134.26, 129.61, 127.69, 68.25, 62.47, 41.28, 41.27, 39.86, 35.85, 35.25, 33.42, 31.33, 27.04, 19.48, 15.74.

HRMS (ESI+) M+Na^+ calculated for $C_{26}H_{38}O_2Si$: 433.2533, found: 433.2532.

Optical Rotation: $[\alpha]_D^{23}$ = -4.7° (*c* = 1.0, $CHCl_3$).

Bromide 5

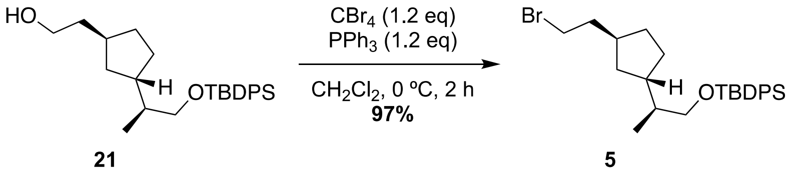

A flask was placed under N_2 atmosphere by three cycles of evacuating and N_2 backfilling, then charged with CBr_4 (538 mg, 1.62 mmol, 1.2 eq.) as solid under N_2 flow and **20** (554 mg, 1.35 mmol, 1.0 eq.) dissolved in CH_2Cl_2 (13 mL). The solution was cooled to 0 °C and PPh_3 (423 mg, 1.61 mmol, 1.2 eq.) was added as solid under N_2 flow in one portion. The reaction mixture was stirred at 0 °C for 2 h. Pentane was added and the solids were filtered off over Celite. The filtrate was concentrated and the crude was purified by flash chromatography (pentane/Et_2O 95/5) to give the product **5** (619 mg, 1.31 mmol, 97% yield) as colourless oil.

^{1}H NMR (400 MHz, Chloroform-*d*) δ 7.72 – 7.64 (m, 4H), 7.47 – 7.35 (m, 6H), 3.62 (dd, *J* = 9.8, 4.6 Hz, 1H), 3.45 (dd, *J* = 9.8, 6.7 Hz, 1H), 3.39 (t, *J* = 7.2 Hz, 2H), 2.00 (hept, *J* = 7.2 Hz, 1H), 1.89 – 1.77 (m, 4H), 1.70 (dtt, *J* = 8.9, 6.2, 3.4 Hz, 1H), 1.54 – 1.44

(m, 2H), 1.33 (ddd, J = 13.1, 8.9, 6.1 Hz, 1H), 1.14 – 1.03 (m,11H), 0.98 (d, J = 6.7 Hz, 3H).

^{13}C NMR (101 MHz, Chloroform-*d*) δ 135.78, 135.76, 134.25, 134.21, 129.64, 127.70, 68.19, 41.33, 41.16, 39.95, 37.45, 35.24, 33.02, 32.82, 31.25, 27.04, 19.48, 15.69.

HRMS (ESI+) M+H$^+$ calculated for $C_{26}H_{37}BrOSi$: 473.1870, found: 473.1870.

Optical Rotation: $[\alpha]_D^{23}$ = -7.0° (c = 0.44, $CHCl_3$).

Preparation of Dithiane 4

Thioester S10

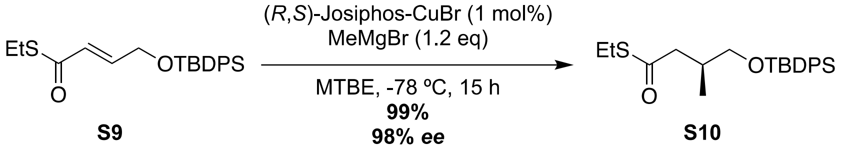

A Schlenk flask was flame-dried under vacuo and subjected to three cycles of evacuating and N_2 backfilling. The flask was charged with (*R*,*S*)-Josiphos-CuBr complex (72.0 mg, 0.09 mmol, 0.01 eq.) and dissolved in dry MTBE (55 mL). The solution was cooled to -78 °C. 3 M MeMgBr in Et_2O (3.7 mL, 11.0 mmol, 1.2 eq.) was added dropwise and the mixture was stirred for 30 min at -78 °C. Thioester **S9** (3.51 g, 9.13 mmol, 1.0 eq.) was dissolved in dry MTBE (15 mL) and added dropwise over 2 h by means of a syringe pump. After complete addition, the reaction mixture was stirred at -78 °C for 15 h. The reaction was quenched by addition of methanol (10 mL) at -78 °C, then sat. aq. NH_4Cl (75 mL) was added and the mixture was warmed to room temperature. The layers were separated and the aqueous layer was extracted with Et_2O (3 x 70 mL). The combined organic layers were washed with water (50 mL), brine (50 mL), dried over $MgSO_4$ and concentrated to give the pure product **S10** (3.62 g, 9.04 mmol, 99% yield, 98% *ee*) as pale orange oil.

^{1}H NMR (400 MHz, Chloroform-*d*) δ 7.76 – 7.61 (m, 4H), 7.52 – 7.32 (m, 6H), 3.57 (dd, J = 9.9, 5.2 Hz, 1H), 3.48 (dd, J = 9.9, 6.2 Hz, 1H), 2.94 – 2.81 (m, 3H), 2.39 (dd, J = 14.5, 8.5 Hz, 1H), 2.35 – 2.25 (m, 1H), 1.26 (t, J = 7.4 Hz, 2H), 1.08 (s, 9H), 0.98 (d, J = 6.6 Hz, 3H).

^{13}C NMR (101 MHz, Chloroform-*d*) δ 199.22, 135.73, 133.79, 133.76, 129.74, 127.78, 68.04, 47.89, 33.90, 26.99, 23.41, 19.43, 16.55, 14.92.

HRMS (ESI+) M+H$^+$ calculated for $C_{23}H_{32}O_2SSi$: 401.1965, found: 401.1964.

Optical Rotation: $[\alpha]_D^{23}$ = -9.4° (c = 0.67, $CHCl_3$).

The enantiomeric excess was determined by chiral HPLC:

Chiracel OD-H column, *n*-heptane : *i*-PrOH = 95:5, 40 °C, flow = 0.5 mL/min, UV detection at 190 nm, 220 nm, 254 nm, retention times for racemate (min): 12.5 (major) and 13.4 (minor).

The analytical data is in agreement with previous reports.[4]

Racemate:

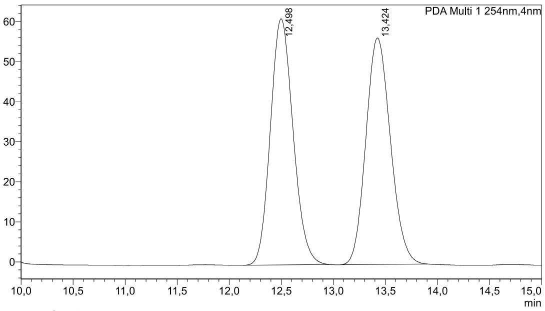

PDA Ch1 254nm

Peak#	Ret. Time	Area	Height	Area%
1	12,498	934477	61443	50,134
2	13,424	929499	56496	49,866
Total		1863976	117939	100,000

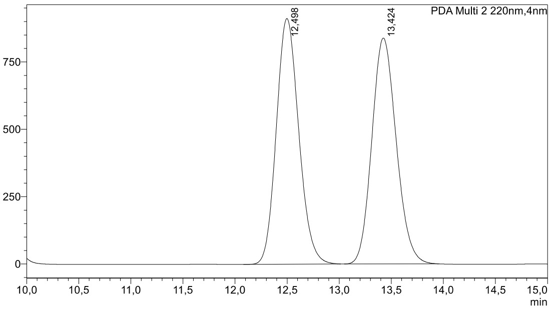

PDA Ch2 220nm

Peak#	Ret. Time	Area	Height	Area%
1	12,498	13686330	911435	50,045
2	13,424	13661621	837892	49,955
Total		27347951	1749326	100,000

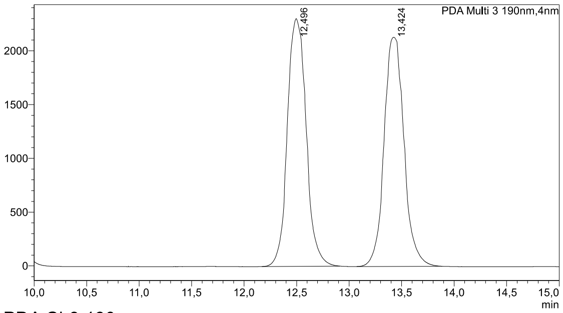

PDA Ch3 190nm

Peak#	Ret. Time	Area	Height	Area%
1	12,496	28707288	2299919	50,582
2	13,424	28046596	2129243	49,418
Total		56753884	4429162	100,000

Enantiomerically enriched product **S10**

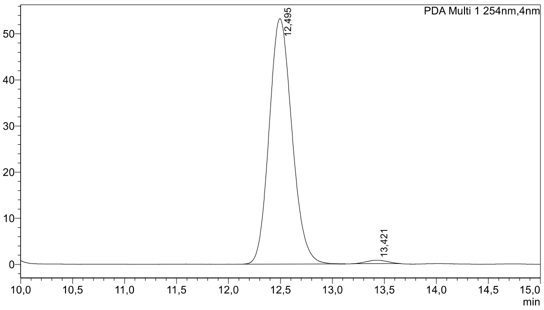

PDA Ch1 254nm

Peak#	Ret. Time	Area	Height	Area%
1	12,495	814008	53227	98,701
2	13,421	10715	749	1,299
Total		824724	53977	100,000

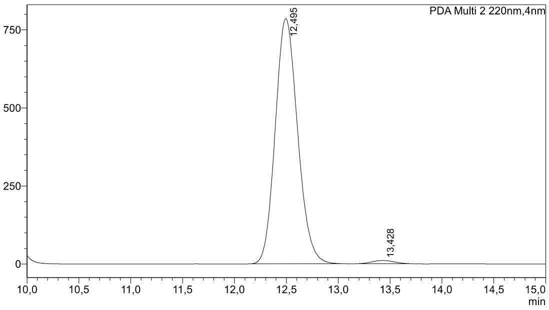

PDA Ch2 220nm

Peak#	Ret. Time	Area	Height	Area%
1	12,495	11811613	785394	98,695
2	13,428	156203	10497	1,305
Total		11967816	795891	100,000

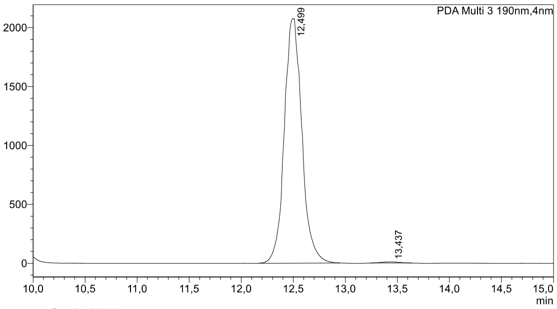

PDA Ch3 190nm

Peak#	Ret. Time	Area	Height	Area%
1	12,499	24348956	2076825	99,482
2	13,437	126802	9721	0,518
Total		24475758	2086546	100,000

Alcohol S11

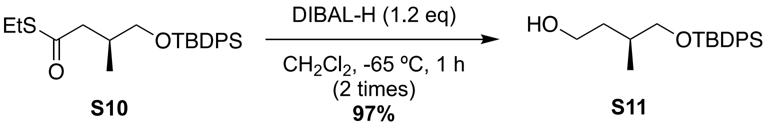

S10 **97%** **S11**

A Schlenk flask was flame-dried under vacuo and subjected to three cycles of evacuating and N_2 backfilling. The flask was charged with **S10** (3.43 g, 8.56 mmol, 1.0 eq.) dissolved in dry CH_2Cl_2 (43 mL) and cooled to -65 °C. 1 M DIBAL-H in CH_2Cl_2 (10.5 mL, 10.5 mmol, 1.2 eq.) was added and the solution was stirred for 1 h. The reaction was quenched by addition of sat. Rochelle salt (50 mL), the reaction mixture was then

warmed to room temperature and stirred for 2 h. The layers were separated and the aqueous phase was extracted with Et_2O (3 x 50 mL). The combined organic phases were washed with water (50 mL) and brine (50 mL), dried over $MgSO_4$ and concentrated. The resulting crude aldehyde was subjected to another cycle of DIBAL-H reduction (conditions as above) and the pure product **S11** (2.85 g, 8.31 mmol, 97% yield) was obtained after workup as colourless oil.

^{1}H NMR (400 MHz, Chloroform-*d*) δ 7.80 – 7.67 (m, 4H), 7.54 – 7.36 (m, 6H), 3.79 – 3.65 (m, 2H), 3.57 (ddd, *J* = 6.2, 3.2, 1.5 Hz, 2H), 2.40 (s, 1H), 1.87 (h, *J* = 6.6 Hz, 1H), 1.73 (dq, *J* = 13.5, 6.6 Hz, 1H), 1.61 – 1.49 (m, 1H), 1.12 (s, 9H), 0.95 (d, *J* = 6.8 Hz, 3H).

^{13}C NMR (101 MHz, Chloroform-*d*) δ 135.71, 135.69, 133.68, 129.75, 127.76, 69.33, 61.16, 61.14, 37.32, 37.30, 33.31, 26.96, 19.34, 17.27.

HRMS (ESI+) M+Na$^+$ calculated for $C_{21}H_{30}O_2Si$: 365.1907. found: 365.1906.

Optical Rotation: $[\alpha]_D^{23}$ = -3.4° (*c* = 0.58, $CHCl_3$).

The analytical data is in agreement with previous reports.[5]

Benzyl ether S13

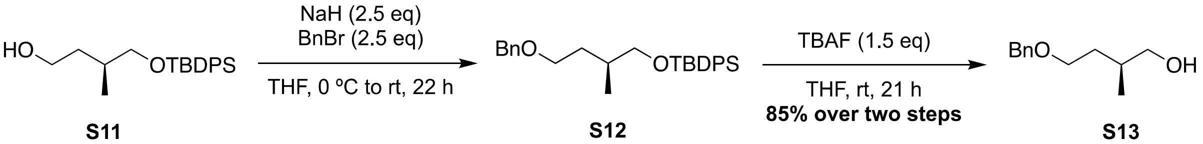

S11 **S12** **S13**

A Schlenk flask was flame-dried under vacuo and subjected to three cycles of evacuating and N_2 backfilling. The flask was charged with NaH (60% in mineral oil, 817 mg, 20.4 mmol, 2.5 eq.) under N_2 flow. The NaH dispersion was washed with pentane (3 x 5 mL), dried under vacuo, suspended in dry THF (10 mL) and cooled to 0 °C. Alcohol **S11** (2.80 g, 8.17 mmol, 1.0 eq.) was added dissolved in dry THF (6 mL) and stirred for 5 min. Then benzyl bromide (2.4 mL, 2.44 mmol, 2.5 eq.) was added. After stirring for 5 min at 0 °C, the reaction mixture was warmed to room temperature and stirred for 22 h. The reaction was quenched by addition of sat. aq. NH_4Cl (25 mL) and the aqueous phase was extracted with Et_2O (3 x 25 mL), washed with water (20 mL) and brine (20 mL), dried over MgSO4 and concentrated to give the crude product **S12** containing benzyl bromide. The crude was used in the next step without further purification.

^{1}H NMR (400 MHz, Chloroform-*d*) δ 7.73 (dt, *J* = 8.0, 1.7 Hz, 4H), 7.51 – 7.29 (m, 11H), 4.53 (s, 2H), 3.63 – 3.52 (m, 4H), 1.89 (dq, *J* = 19.6, 7.0, 6.3 Hz, 2H), 1.58 – 1.48 (m, 1H), 1.13 (s, 9H), 1.02 (d, *J* = 6.7 Hz, 3H).

^{13}C NMR (101 MHz, Chloroform-*d*) δ 138.82, 135.76, 134.14, 134.13, 129.64, 128.46, 127.72, 127.57, 72.96, 68.97, 68.78, 33.34, 33.09, 27.04, 19.47, 17.09.

HRMS (ESI+) M+H$^+$ calculated for $C_{28}H_{36}O_2Si$: 433.2557, found: 433.2558.

A flask was charged with crude **S12** and dissolved in THF (27 mL). To the stirred solution was added 1 M TBAF in THF (12.3 mL, 12.3 mmol, 1.5 eq.) and the reaction mixture was stirred at room temperature for 21 h. Sat. aq. NH_4Cl (50 mL) was added and the aqueous phase was extracted with Et_2O (3 x 50 mL). The combined organic extracts were washed with brine (50 mL), dried over $MgSO_4$ and concentrated. The

crude was purified by flash chromatography (pentane/Et_2O 6/2 to 6/5) to give the product **S13** (1.353 g, 6.96 mmol, 85% yield over two steps) as colourless liquid.

H NMR (400 MHz, Chloroform-*d*) δ 7.38 – 7.26 (m, 5H), 4.51 (s, 2H), 3.61 – 3.49 (m, 2H), 3.44 (qd, *J* = 10.8, 5.8 Hz, 2H), 2.92 (s, 1H), 1.85 – 1.67 (m, 2H), 1.54 (dtd, *J* = 14.3, 6.2, 5.0 Hz, 1H), 0.92 (d, *J* = 6.8 Hz, 3H).

^{13}C NMR (101 MHz, Chloroform-*d*) δ 138.08, 128.43, 128.42, 127.75, 127.70, 73.09, 68.68, 67.94, 33.91, 33.89, 17.12.

HRMS (ESI+) M+H$^+$ calculated for $C_{12}H_{18}O_2$: 195.1380, found: 195.1378.

Optical Rotation: $[\alpha]_D^{23}$ = -12.4° (*c* = 0.37, $CHCl_3$).

The analytical data is in agreement with previous reports.[6]

Aldehyde S14

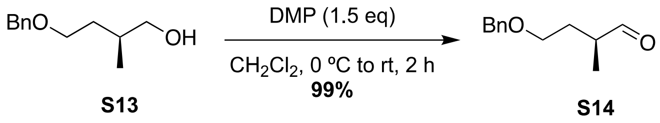

A flask was placed under N_2 atmosphere by three cycles of evacuating and N_2 backfilling, then charged with **S13** (856 mg, 4.41 mmol, 1.0 eq.) dissolved in dry CH_2Cl_2 (22 mL) and cooled to 0 °C. Solid Dess-Martin-periodinane (2.80 g, 6.61 mmol, 1.5 eq.) was added in one portion under N_2 flow. The reaction mixture was stirred for 10 min at 0 °C and then for 2 h at room temperature. The reaction was quenched by addition of sat. aq. $NaHCO_3$ (50 mL). The layers were separated and the organic phase was extracted with Et_2O (3 x 30 mL). The combined organic layers were washed with brine (40 mL), dried over $MgSO_4$ and concentrated. The crude was purified by filtration over a short silica column (pentane/Et_2O 3/1) to give the product **S14** (836 mg, 4.35 mmol, 99% yield) as colourless liquid.

^{1}H NMR (400 MHz, Chloroform-*d*) δ 9.65 (d, *J* = 1.6 Hz, 1H), 7.40 – 7.25 (m, 5H), 4.48 (s, 2H), 3.63 – 3.47 (m, 2H), 2.55 (h, *J* = 7.1 Hz, 1H), 2.12 – 1.99 (m, 1H), 1.76 – 1.64 (m, 1H), 1.11 (d, *J* = 7.1 Hz, 3H).

^{13}C NMR (101 MHz, Chloroform-*d*) δ 204.83, 138.27, 128.49, 127.74, 73.14, 67.46, 43.85, 30.90, 13.34.

The analytical data is in agreement with previous reports.[7]

Dithiane 4

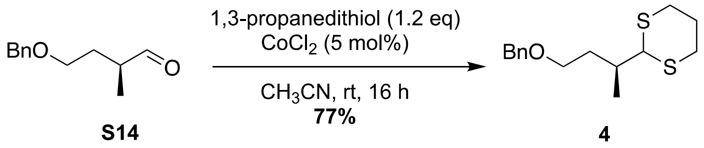

A flask was charged with **S14** (836 mg, 4.35 mmol, 1.0 eq.) and dissolved in CH_3CN (22 mL). 1,3-Propanedithiol (0.52 mL, 5.18 mmol, 1.2 eq.) was added followed by $CoCl_2$ (28.2 mg, 0.22 mmol, 0.05 eq.). The resulting solution was stirred at room temperature for 16 h. The solvent was evaporated and the crude was purified by flash

chromatography (pentane/Et_2O 95/5) to give the product **4** (949 mg, 3.36 mmol, 77% yield) as colourless liquid.

^{1}H NMR (400 MHz, Chloroform-*d*) δ 7.40 – 7.25 (m, 5H), 4.51 (s, 2H), 4.13 (d, *J* = 4.0 Hz, 1H), 3.59 – 3.48 (m, 2H), 2.92 – 2.79 (m, 4H), 2.19 – 2.05 (m, 2H), 1.98 (ddt, *J* = 12.0, 7.1, 5.9 Hz, 1H), 1.89 – 1.76 (m, 1H), 1.61 (ddt, *J* = 14.2, 8.3, 6.0 Hz, 1H), 1.10 (d, *J* = 6.9 Hz, 3H).

^{13}C NMR (101 MHz, Chloroform-*d*) δ 138.70, 128.50, 127.79, 127.65, 72.91, 68.15, 55.49, 35.59, 33.94, 31.25, 30.92, 26.51, 17.12.

HRMS (ESI+) M+Na$^+$ calculated for $C_{15}H_{22}OS_2$: 305.1004, found: 305.1002.

Optical Rotation: $[\alpha]_D^{23}$ = -1.3° (*c* = 0.38, $CHCl_3$).

Dithiane Alkylation of Bromide 5

Optimization of lithiation conditions

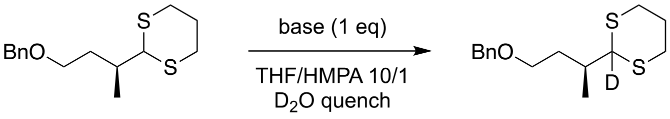

4 **S15**

Entry	Base	Temperature	Time	Lithiation[a]	Remarks
**1**	*n*-BuLi	0 ºC	10 min	0%	
**2**	*n*-BuLi	0 ºC	1 h	n.d.	complete degradation
**3**	*t*-BuLi	-40 ºC	10 min	n.d.	partial degradation
**4**	*t*-BuLi	-78 ºC	1 h	60%	
**5**	*t*-BuLi	-78 ºC	15 min	67%	
**6**	*t*-BuLi	-78 ºC	5 min	64%	

[a] Determined by ^{1}H NMR by integration and comparison of the signals of the benzylic CH_2 (4.44 ppm, s, 2H) and remaining dithiane CH (4.06 ppm, d, 1H).

Dithiane 22

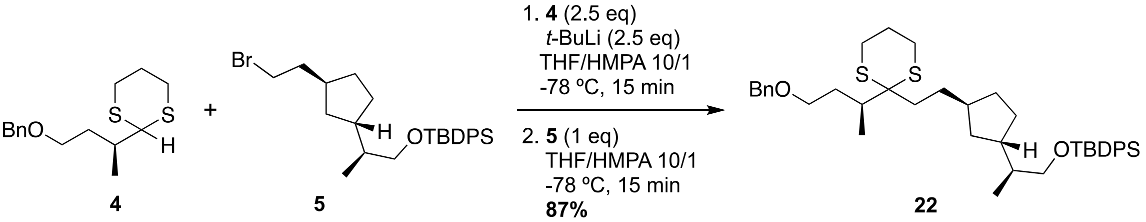

4 **5** **22**

A Schlenk flask was flame-dried under vacuo and subjected to three cycles of evacuating and N_2 backfilling. The flask was charged with dithiane **4** (157 mg, 0.55 mmol, 2.5 eq.) dissolved in dry THF (1.1 mL) and HMPA (0.11 mL) and the solution was cooled to -78 °C. 1.7 M *t*-BuLi in hexanes (0.33 mL, 0.56 mmol, 2.5 eq.) was added. The resulting dark red solution was stirred for 15 min at -78 °C. Bromide **5** (105 mg,

0.22 mmol, 1.0 eq.) was added dissolved in dry THF (0.8 mL) and HMPA (0.08 mL). The flask was rinsed with additional dry THF (0.3 mL) and HMPA (0.03 mL). After stirring for 15 min, the reaction was quenched by addition of sat. aq. NH_4Cl (3 mL). After warming to room temperature, water (5 mL) was added and the aqueous phase was extracted with Et_2O (3 x 15 mL). The combined organic extracts were washed with brine (10 mL), dried over $MgSO_4$ and concentrated. The crude was purified by flash chromatography (pentane/Et_2O 96/4) to give the product **22** (134 mg, 0.19 mmol, 87% yield) as colourless oil.

^{1}H NMR (400 MHz, Chloroform-*d*) δ 7.69 – 7.63 (m, 4H), 7.46 – 7.32 (m, 10H), 7.32 – 7.26 (m, 1H), 4.56 (d, *J* = 12.0 Hz, 1H), 4.48 (d, *J* = 12.0 Hz, 1H), 3.64 – 3.48 (m, 3H), 3.42 (ddd, *J* = 9.8, 7.0, 0.9 Hz, 1H), 2.86 – 2.70 (m, 4H), 2.39 (dt, *J* = 14.9, 7.8 Hz, 1H), 2.15 (dq, *J* = 15.6, 7.6, 6.7 Hz, 1H), 1.99 – 1.83 (m, 4H), 1.84 – 1.57 (m, 5H), 1.52 – 1.38 (m, 5H), 1.38 – 1.31 (m, 1H), 1.09 (d, *J* = 6.8 Hz, 3H), 1.05 (s, 9H), 0.97 (d, *J* = 6.7 Hz, 3H).

^{13}C NMR (101 MHz, Chloroform-*d*) δ 138.82, 135.78, 134.32, 134.29, 129.60, 128.47, 127.69, 127.59, 77.36, 72.79, 69.32, 68.31, 59.33, 41.43, 41.38, 39.25, 36.14, 35.68, 34.62, 33.48, 31.93, 31.50, 31.40, 27.05, 25.89, 25.73, 25.47, 19.48, 15.83, 14.60.

HRMS (ESI+) M+H^{+} calculated for $C_{41}H_{58}O_2S_2Si$: 675.3720, found: 675.3708.

HRMS (ESI+) M+H^{+} calculated for $C_{41}H_{58}O_2S_2Si$: 697.3540, found: 697.3525.

Optical Rotation: $[\alpha]_D^{23}$ = -21.3° (*c* = 0.31, $CHCl_3$).

Preparation of Iodide 9

Alcohol 23

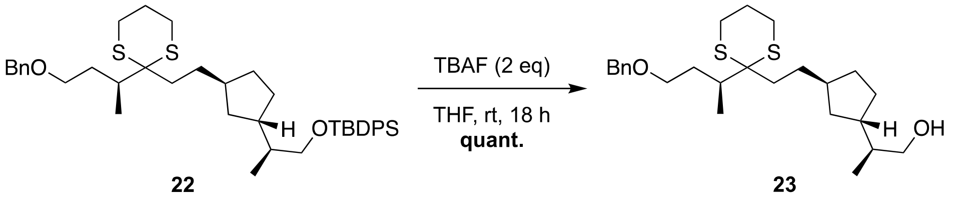

22 23

A flask was charged with **22** (200 mg, 0.30 mmol, 1.0 eq.) and dissolved in THF (3 mL). 1 M TBAF in THF (0.59 mL, 0.59 mmol, 2.0 eq.) was added and the solution stirred at room temperature for 18 h. Sat. aq. NH_4Cl (10 mL) was added and the aqueous phase was extracted with Et_2O (3 x 10 mL). The combined organic extracts were washed with brine (10 mL), dried over $MgSO_4$ and concentrate. The crude was purified by flash chromatography (pentane/Et_2O 1/1) to give the product **23** (129 mg, 0.30 mmol, quant. yield) as colourless oil.

^{1}H NMR (400 MHz, Chloroform-*d*) δ 7.34 (d, *J* = 4.3 Hz, 4H), 7.30 – 7.27 (m, 1H), 4.56 (d, *J* = 12.0 Hz, 1H), 4.48 (d, *J* = 12.0 Hz, 1H), 3.63 (dd, *J* = 10.4, 4.6 Hz, 1H), 3.59 – 3.48 (m, 2H), 3.39 (dd, *J* = 10.5, 7.2 Hz, 1H), 2.87 – 2.70 (m, 4H), 2.38 (dt, *J* = 14.7, 7.8 Hz, 1H), 2.16 (dtd, *J* = 15.3, 7.4, 3.8 Hz, 1H), 2.00 – 1.82 (m, 5H), 1.82 – 1.71 (m, 3H), 1.57 – 1.46 (m, 2H), 1.45 – 1.36 (m, 4H), 1.22 – 1.11 (m, 2H), 1.09 (d, *J* = 6.8 Hz, 3H), 0.94 (d, *J* = 6.7 Hz, 3H).

^{13}C NMR (101 MHz, Chloroform-*d*) δ 138.74, 128.45, 127.69, 127.59, 72.77, 69.26, 67.60, 59.25, 41.35, 41.26, 39.13, 36.12, 35.55, 34.54, 33.43, 31.84, 31.50, 31.42, 25.83, 25.70, 25.42, 15.46, 14.58.

HRMS (ESI+) M+Na+ calculated for $C_{25}H_{40}O_2S_2$: 459.2362, found: 459.2347.

Optical Rotation: $[\alpha]_D^{23}$ = -27.4° (*c* = 0.93, $CHCl_3$).

Iodide 9

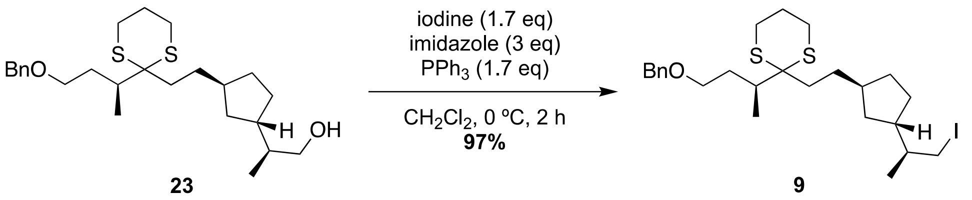

A flask was placed under N_2 atmosphere by three cycles of evacuating and N_2 backfilling, then charged with imidazole (71.6 mg, 1.05 mmol, 3.0 eq.) and PPh_3 (156 mg, 0.60 mmol, 1.7 eq.) as solids under N_2 flow and **23** (157 mg, 0.36 mmol, 1 eq.) dissolved in CH_2Cl_2 (2.4 mL). The solution was cooled to 0 °C and Iodine (155 mg, 0.61 mmol, 1.7 eq.) was added in one portion as solid under N_2 flow. The reaction mixture was stirred for 2 h at 0 °C. The solvent was evaporated and the crude was purified by flash chromatography (pentane/Et_2O 95/5) to give the product **9** (191 mg, 0.35 mmol, 97% yield.) as colourless oil.

^{1}H NMR (400 MHz, Chloroform-*d*) δ 7.34 (d, *J* = 4.4 Hz, 4H), 7.30 – 7.26 (m, 1H), 4.56 (d, *J* = 11.9 Hz, 1H), 4.48 (d, *J* = 12.0 Hz, 1H), 3.61 – 3.48 (m, 2H), 3.32 (dd, *J* = 9.5, 3.4 Hz, 1H), 3.13 (dd, *J* = 9.5, 6.8 Hz, 1H), 2.85 – 2.72 (m, 4H), 2.38 (dddd, *J* = 13.9, 8.9, 6.8, 2.0 Hz, 1H), 2.16 (dtd, *J* = 13.5, 6.9, 2.2 Hz, 1H), 1.99 – 1.70 (m, 8H), 1.56 – 1.36 (m, 5H), 1.23 – 1.13 (m, 2H), 1.13 – 1.07 (m, 4H), 0.99 (d, *J* = 6.5 Hz, 3H).

^{13}C NMR (101 MHz, Chloroform-*d*) δ 138.80, 128.47, 127.69, 127.60, 72.81, 69.29, 59.26, 44.46, 40.55, 39.52, 36.27, 35.63, 34.57, 33.39, 31.92, 31.47, 31.44, 25.88, 25.75, 25.43, 19.76, 17.81, 14.62.

HRMS (ESI+) M+H+ calculated for $C_{25}H_{39}IOS_2$: 547.1560, found: 547.1545.

Optical Rotation: $[\alpha]_D^{23}$ = -15.1° (*c* = 0.85, $CHCl_3$).

Preparation of Dithiane 6

Silylether S16

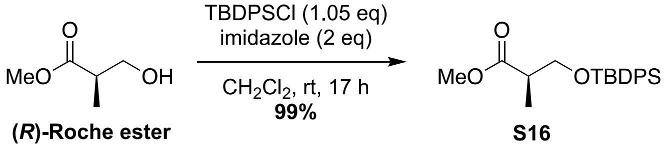

A Schlenk flask was flame-dried under vacuo and subjected to three cycles of evacuating and N_2 backfilling. The flask was charged with (*R*)-Roche ester (4.7 mL, 42.4 mmol, 1.0 eq.) and imidazole (5.68 g, 83.4 mmol, 2.0 eq.) as solid under N_2 flow. Dry CH_2Cl_2 (83 mL) was added and to the stirred solution was added TBDPSCl (11.6 mL, 12.3 g, 44.5 mmol, 1.05 eq.). The reaction mixture was stirred at room temperature for 17 h. The reaction was quenched by addition of sat. aq. NH_4Cl (100 mL). The layers were separated and the aqueous phase was extracted with CH_2Cl_2 (3 x 60 mL). The combined organic layers were washed with 1 M aq. HCl (100 mL) and brine (75 mL),

dried over $MgSO_4$ and concentrated to give the product **S16** (14.9 g, 41.8 mmol, 99% yield) as colourless oil.

^{1}H NMR (400 MHz, Chloroform-*d*) δ 7.71 – 7.63 (m, 4H), 7.48 – 7.36 (m, 6H), 3.86 (dd, *J* = 9.7, 6.9 Hz, 1H), 3.76 (dd, *J* = 9.7, 5.7 Hz, 1H), 3.70 (d, *J* = 1.5 Hz, 3H), 2.79 – 2.69 (m, 1H), 1.18 (d, *J* = 7.1 Hz, 3H), 1.06 (s, 9H).

^{13}C NMR (101 MHz, Chloroform-*d*) δ 175.49, 135.70, 133.65, 133.59, 129.79, 127.79, 66.04, 51.65, 42.52, 26.84, 19.36, 13.60.

HRMS (ESI+) M+Na$^+$ calculated for $C_{21}H_{28}O_3Si$: 379.1700, found: 379.1702.

Optical Rotation: $[\alpha]_D^{23}$ = -18.3° (*c* = 0.54, $CHCl_3$).

The analytical data is in agreement with previous reports.[8]

Alcohol S17

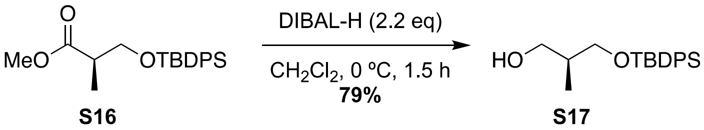

A Schlenk flask was flame-dried under vacuo and subjected to three cycles of evacuating and N_2 backfilling. The flask was charged with **S16** (14.9 g, 41.8 mmol, 1.0 eq.) dissolved in dry CH_2Cl_2 (210 mL) and cooled to 0 °C. 1 M DIBAL-H in CH_2Cl_2 (93 mL, 93 mmol, 2.2 eq.) was added and the reaction mixture was stirred at 0 °C for 1.5 h. The reaction was quenched by slow addition of sat. aq. Rochelle salt (175 mL) and allowed to come to room temperature and stirred for 20 h. The layers were separated and the aqueous phase was extracted with EtOAc (3 x 100 mL). The combined organic phases were washed with brine (200 mL), dried over $MgSO_4$ and concentrated. The crude was purified by flash chromatography (pentane/Et_2O 9/1 to 1/1) to give the product **S17** (10.8 g, 33.0 mmol, 79% yield) as colourless oil.

^{1}H NMR (400 MHz, Chloroform-*d*) δ 7.39 – 7.24 (m, 5H), 4.60 (d, *J* = 12.1 Hz, 1H), 4.55 (d, *J* = 12.1 Hz, 1H), 4.35 – 4.25 (m, 1H), 4.05 (dd, *J* = 8.3, 6.4 Hz, 1H), 3.74 (dd, *J* = 8.3, 6.3 Hz, 1H), 3.56 (dd, *J* = 9.8, 5.7 Hz, 1H), 3.47 (dd, *J* = 9.8, 5.5 Hz, 1H), 1.43 (s, 3H), 1.37 (s, 3H).

^{13}C NMR (101 MHz, Chloroform-*d*) δ 138.05, 128.46, 127.79, 127.77, 109.45, 74.81, 73.56, 71.16, 66.94, 26.84, 25.47.

HRMS (ESI+) M+Na$^+$ calculated for $C_{20}H_{28}O_2Si$: 351.1751, found: 351.1753.

Optical Rotation: $[\alpha]_D^{23}$ = -9.3° (*c* = 0.29, $CHCl_3$).

The analytical data is in agreement with previous reports.[9]

Iodide S18

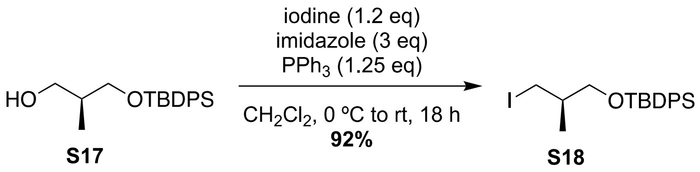

A flask charged with **S17** (2.15 g, 6.56 mmol, 1.0 eq.), PPh_3 (2.14 g, 8.16 mmol, 1.25 eq.) and imidazole (1.33 g, 19.6 mmol, 3.0 eq) was placed under N_2 atmosphere by three cycles of evacuating and N_2 backfilling. Dry CH_2Cl_2 (26 mL) was added and the resulting solution was cooled to 0 °C. Iodine (1.97 g, 7.77 mmol, 1.2 eq) was added as solid under N_2 flow. After stirring for 5 min at 0 °C the reaction mixture was stirred at room temperature for 18 h. The reaction was diluted with Et_2O (100 mL) and washed with aq. $Na_2S_2O_3$ (2 g/100 mL, 100 mL), 1 M aq. HCl (2 x 50 mL), brine (50 mL), dried over $MgSO_4$ and concentrated. The crude was purified by flash chromatography (pentane 100%) to give the product **S18** (2.63 g, 6.00 mmol, 92% yield) as colourless oil.

^{1}H NMR (400 MHz, Chloroform-*d*) δ 7.72 – 7.66 (m, 4H), 7.50 – 7.37 (m, 6H), 3.74 (ddd, *J* = 10.1, 4.5, 1.0 Hz, 1H), 3.69 (dd, *J* = 5.8, 1.6 Hz, 1H), 3.61 (dd, *J* = 9.9, 7.8 Hz, 1H), 2.57 (s, 1H), 2.10 – 1.95 (m, 1H), 1.08 (s, 4H), 0.85 (d, *J* = 7.0 Hz, 2H).

^{13}C NMR (101 MHz, Chloroform-*d*) δ 135.72, 135.71, 133.31, 133.29, 129.93, 127.90, 127.87, 68.82, 67.75, 37.45, 26.97, 19.28, 13.30.

HRMS (ESI+) M+H$^+$ calculated for $C_{20}H_{27}IOSi$: 439.0949, found: 439.0950.

Optical Rotation: $[\alpha]_D^{23}$ = -5.6° (*c* = 0.52, $CHCl_3$).

The analytical data is in agreement with previous reports.[10]

Dithiane 6

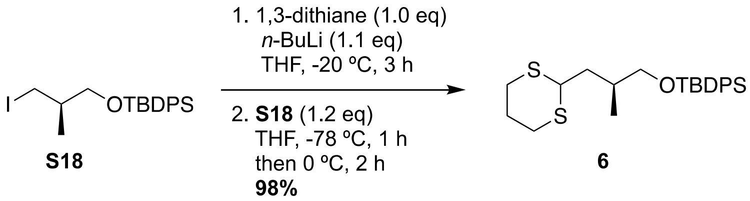

A Schlenk flask was flame-dried under vacuo and subjected to three cycles of evacuating and N_2 backfilling. The flask was charged with 1,3-dithiane (312 mg, 2.55 mmol, 1.0 eq.) under N_2 flow. Dry THF (8 mL) was added and the resulting solution was cooled to -20 °C (ice/methanol bath). 1.6 M *n*-BuLi in hexanes (1.8 mL, 2.85 mmol, 1.1 eq.) was added and the reaction mixture was stirred for 3 h before cooling to -78 °C. Iodide **S18** (1.36 g, 3.11 mmol, 1.2 eq.) was added dissolved in dry THF (2.0 mL). The reaction mixture was stirred for 1 h at -78 °C and then for 2 h at 0 °C. The reaction was quenched by addition of sat. aq. NH_4Cl (20 mL) and the aqueous phase was extracted with Et_2O (3 x 25 mL). The combined organic extracts were washed with brine (25 mL), dried over $MgSO_4$ and concentrated. The crude was purified by flash chromatography (pentane 100% to pentane/Et_2O 98/2) to give the product **6** (1.10 g, 2.55 mmol, 98% yield) as colourless liquid.

^{1}H NMR (400 MHz, Chloroform-*d*) δ 7.69 (ddt, *J* = 7.8, 3.5, 1.8 Hz, 4H), 7.48 – 7.33 (m, 6H), 4.06 (dd, *J* = 8.0, 6.9 Hz, 1H), 3.53 (d, *J* = 5.5 Hz, 2H), 2.82 (dt, *J* = 9.5, 3.1 Hz, 4H), 2.15 – 2.03 (m, 2H), 1.97 (ddd, *J* = 14.1, 8.0, 6.2 Hz, 1H), 1.93 – 1.82 (m, 1H), 1.56 (dt, *J* = 14.3, 7.3 Hz, 1H), 1.08 (s, 9H), 0.98 (d, *J* = 6.7 Hz, 3H).

^{13}C NMR (101 MHz, Chloroform-*d*) δ 135.78, 135.76, 133.92, 133.90, 129.68, 129.67, 127.75, 68.40, 45.61, 39.11, 32.68, 30.51, 30.40, 27.01, 26.22, 19.43, 16.97.

HRMS (ESI+) M+Na$^+$ calculated for $C_{23}H_{32}OS_2Si$: 453.1713, found: 453.1709.

Optical Rotation: $[\alpha]_D^{23}$ = -11.9° (*c* = 0.36, $CHCl_3$).

Dithiane Alkylation of Iodide 9

Optimization of lithiation conditions

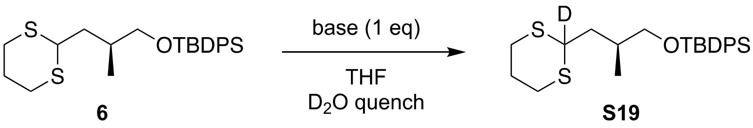

Entry	Base	Co-solvent (10%)	Temperature	Time	Lithiation[a]
**1**	*t*-BuLi	HMPA	-78 ºC	15 min	14%
**2**	*t*-BuLi	HMPA	0 ºC	15 min	7%
**3**	*t*-BuLi	–	0 ºC	15 min	5%
**4**	*n*-BuLi	HMPA	0 ºC	15 min	18%
**5**	*n*-BuLi	–	0 ºC	15 min	62%
**6**	*n*-BuLi	–	0 ºC	1 h	40%

[a] Determined by ^{1}H NMR by integration and comparison of signals of CH_2OTBDPS (3.51 ppm, d, 2H) and remaining dithiane proton (4.05 ppm, dd, 1H).

Dithiane 3

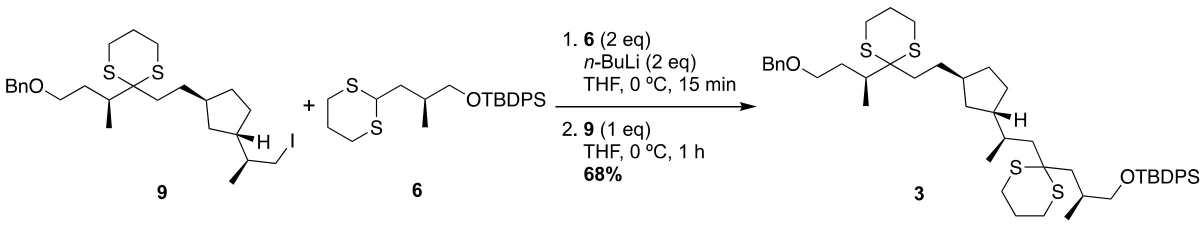

A Schlenk flask was flame-dried under vacuo and subjected to three cycles of evacuating and N_2 backfilling. The flask was charged with ditihiane **6** (634 mg, 1.47 mmol, 2.0 eq.) dissolved in 7.5 mL dry THF and the solution was cooled to 0 °C. 1.6 M *n*-BuLi in hexanes (0.92 mL, 1.47 mmol, 2.0 eq.) was added and the solution was stirred for 15 min. Iodide **9** (402 mg, 0.74 mmol, 1.0 eq.) was added dissolved in 1.5 mL dry THF and the reaction mixture was stirred for 1 h at 0 °C. The reaction was quenched by addition of sat. aq. NH_4Cl (30 mL) and allowed to come to room temperature. The aqueous phase was extracted with Et_2O (3 x 30 mL) and the combined organic extracts were washed with brine (25 mL), dried over $MgSO_4$ and concentrated. The crude. was

purified by flash chromatography (pentane/Et_2O 95/5) to give the product **3** (424 mg, 0.50 mmol, 68% yield) as colourless oil.

^{1}H NMR (400 MHz, Chloroform-*d*) δ 7.71 – 7.65 (m, 4H), 7.45 – 7.32 (m, 10H), 7.31 – 7.27 (m, 1H), 4.57 (d, *J* = 12.0 Hz, 1H), 4.48 (d, *J* = 12.0 Hz, 1H), 3.62 – 3.46 (m, 4H), 2.90 – 2.60 (m, 8H), 2.40 (dt, *J* = 14.4, 7.6 Hz, 1H), 2.23 (dd, *J* = 15.0, 3.6 Hz, 1H), 2.19 – 2.12 (m, 1H), 2.05 (h, *J* = 6.2 Hz, 1H), 2.00 – 1.69 (m, 13H), 1.65 (ddd, *J* = 14.9, 6.4, 4.6 Hz, 2H), 1.54 – 1.38 (m, 4H), 1.37 – 1.30 (m, 1H), 1.24 – 1.17 (m, 1H), 1.10 (d, *J* = 6.8 Hz, 6H), 1.07 (s, 9H), 1.01 (d, *J* = 6.3 Hz, 3H).

^{13}C NMR (101 MHz, Chloroform-*d*) δ 138.77, 135.79, 135.78, 134.04, 133.96, 129.65, 128.45, 127.72, 127.71, 127.68, 127.58, 72.75, 69.57, 69.26, 59.28, 55.14, 46.12, 46.03, 42.77, 39.70, 35.58, 35.40, 34.53, 34.16, 33.39, 33.04, 31.87, 31.48, 31.11, 27.04, 26.71, 26.58, 25.86, 25.69, 25.45, 25.10, 20.64, 19.84, 19.46, 14.56.

HRMS (ESI+) M+H$^+$ calculated for $C_{49}H_{72}O_2S_4Si$: 849.4257, found: 849.4245.

Optical Rotation: $[\alpha]_D^{23}$ = -9.8° (*c* = 0.60, $CHCl_3$).

Fragment A

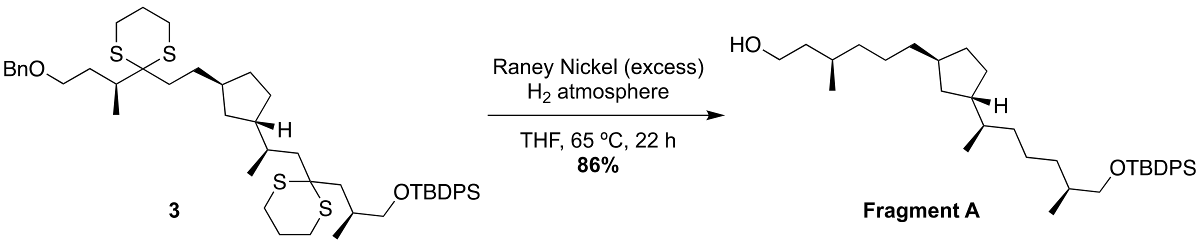

A flask was charged with Raney-Nickel (4 mL, 50% dispersion in water, 10 eq. by wt., W.R. Grace and Co. Raney®2800, purchased from Sigma-Aldrich) and washed with THF (3 x 5 mL), then **3** (197 mg, 0.23 mmol, 1.0 eq.) was added dissolved in THF (5 mL). H_2 was bubbled through the suspension for 2 min. The suspension was then heated to 65 °C and stirred for 22 h under H_2 atmosphere (balloon). The suspension was cooled to room temperature and the solids were removed by filtration over Celite. The filtrate was concentrated and the crude was purified by flash chromatography (pentane/EtOAc 9/1) to give the product **Fragment A** (110 mg, 0.20 mmol, 86% yield) as colourless oil.

^{1}H NMR (400 MHz, Chloroform-*d*) δ 7.68 (d, *J* = 6.2 Hz, 4H), 7.47 – 7.33 (m, 6H), 3.75 – 3.63 (m, 2H), 3.53 (dd, *J* = 9.8, 5.6 Hz, 1H), 3.44 (dd, *J* = 9.8, 6.4 Hz, 1H), 1.86 – 1.71 (m, 3H), 1.69 – 1.52 (m, 4H), 1.46 – 1.34 (m, 6H), 1.33 – 1.19 (m, 8H), 1.07 (d, *J* = 1.3 Hz, 12H), 1.04 – 0.97 (m, 1H), 0.93 (d, *J* = 6.7 Hz, 3H), 0.90 (d, *J* = 6.6 Hz, 3H), 0.82 (d, *J* = 6.6 Hz, 3H).

^{13}C NMR (101 MHz, Chloroform-*d*) δ 135.77, 134.29, 134.28, 129.59, 127.68, 69.00, 61.41, 44.92, 40.13, 39.22, 38.35, 37.51, 37.30, 36.14, 35.89, 35.80, 33.71, 33.49, 31.40, 29.62, 27.02, 26.05, 24.45, 19.80, 19.78, 19.47, 17.86, 17.20.

HRMS (ESI+) M+Na$^+$ calculated for $C_{36}H_{58}O_2Si$: 573.4098, found: 573.4084.

Optical Rotation: $[\alpha]_D^{23}$ = -5.48° (*c* = 0.13, $CHCl_3$).

Preparation of cyclopentene acetate 14

Diacetate 24

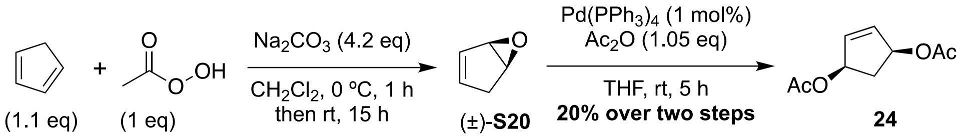

A 2-L flask was charged with Na_2CO_3 (128 g, 1.2 mol, 4.2 eq.) and suspended in CH_2Cl_2 (380 mL). Freshly prepared cyclopentadiene (26.5 mL, 315 mmol, 1.1 eq.) was added and the suspension was cooled to 0 °C. Peracetic acid (35% in acetic acid, 54 mL, 286 mmol, 1 eq.) was added dropwise over 20 min. The reaction mixture was stirred at 0 °C for one h and then for 15 h at room temperature. The solids were filtered off and the filtrate was concentrated (22 °C water bath temperature, 300 mbar) to give the crude epoxide (±)-**S20** (30.88 g, contained CH_2Cl_2) which was used without further purification.

^{1}H NMR (400 MHz, Chloroform-*d*) δ 6.14 (dtd, *J* = 5.6, 2.2, 1.1 Hz, 1H), 5.98 (dt, *J* = 5.9, 2.2 Hz, 1H), 3.90 (td, *J* = 3.2, 2.2 Hz, 1H), 3.81 (dq, *J* = 2.9, 0.9 Hz, 1H), 2.62 (dq, *J* = 19.1, 2.2 Hz, 1H), 2.43 – 2.34 (m, 1H).

^{13}C NMR (101 MHz, Chloroform-*d*) δ 137.96, 131.39, 59.31, 56.93, 35.71.

The analytical data is in agreement with previous reports.[11]

A flask was placed under N_2 atmosphere by three cycles of evacuating and N_2 backfilling and charged with $Pd(PPh_3)_4$ (1.74 g, 1.51 mmol, 0.01 eq.) under N_2 flow. THF (150 mL) and Ac_2O (15 mL, 159 mmol, 1.1 eq.) were added. To the remaining solution was added crude epoxide (±)-**S20** dissolved in THF (30 mL). The reaction mixture was stirred for 5 h at room temperature. The solvent was evaporated (45 °C water bath temperature, 100 mbar). The crude was purified by flash chromatography (pentane/Et_2O 5/2) to give the product **24** (11.0 g, 59.7 g, 20% yield over 2 steps).

^{1}H NMR (400 MHz, Chloroform-*d*) δ 6.08 (s, 2H), 5.54 (dd, *J* = 7.5, 3.8 Hz, 2H), 2.87 (dt, *J* = 15.1, 7.6 Hz, 1H), 2.05 (s, 6H), 1.73 (dt, *J* = 14.9, 3.8 Hz, 1H).

^{13}C NMR (101 MHz, Chloroform-*d*) δ 170.73, 134.68, 76.67, 37.20, 21.17.

HRMS (ESI+) M+Na$^+$ calculated for $C_9H_{12}O_4$: 207.0628, found: 207.0627.

The analytical data is in agreement with previous reports.[12]

Monoacetate 25

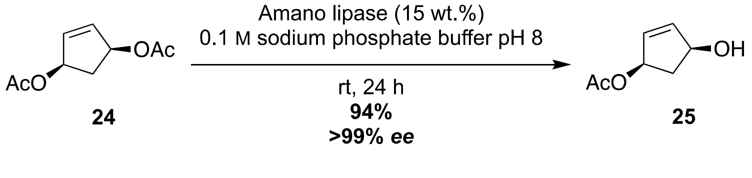

A flask was charged with diacetate **24** (3.03 g, 16.5 mmol, 1.0 eq.) and suspended in 0.1 M phosphate buffer pH 8 (165 mL). Amano lipase (452 mg, 15% by wt.) was added and the reaction mixture was stirred at room temperature for 24 h. Brine (50 mL) was added and the aqueous phase was extracted with EtOAc (4 x 100 mL), dried over

$MgSO_4$ and concentrated (40 °C water bath temperature, 100 mbar) to give the product **25** (2.21 g, 15.5 mmol, 94% yield) as pale orange half solid.

^{1}H NMR (400 MHz, Chloroform-*d*) δ 6.10 (d, *J* = 5.7 Hz, 1H), 5.97 (d, *J* = 6.8 Hz, 1H), 5.52 – 5.44 (m, 1H), 4.74 – 4.68 (m, 1H), 2.79 (dt, *J* = 14.7, 7.4 Hz, 1H), 2.04 (s, 3H), 1.64 (dt, *J* = 14.6, 3.9 Hz, 1H).

^{13}C NMR (101 MHz, Chloroform-*d*) δ 170.95, 138.63, 132.67, 77.21, 74.94, 40.60, 21.31.

HRMS (ESI+) M+Na$^+$ calculated for $C_7H_{10}O_3$: 165.0522, found: 165.0523.

Optical Rotation: $[\alpha]_D^{23}$ = +55.7° (*c* = 0.96, $CHCl_3$).

The enantiomeric excess was determined by chiral GC

A GC instrument equipped with a FS-Hydrodex-beta-3P column (25.0 m × 0.25 mm x 0.25 µm) and a flame ionization detector. The conditions for cis-4-hydroxycyclopent-2-en-1-yl acetateare 40 °C to 230 °C (ramp 5 °C/min) and back to 40 °C (10 °C/min), 1 mL H_2 carrier gas flow, split ratio 50:1. Retention times racemate (min): 22.19 (*R,S*)-enantiomer), 22.54 (*S,R*)-enantiomer).

The enantiomeric excess was determined to be >99%, no (*S*,*R*)-enantiomer detected.

The analytical data is in agreement with previous reports.[13]

Racemate:

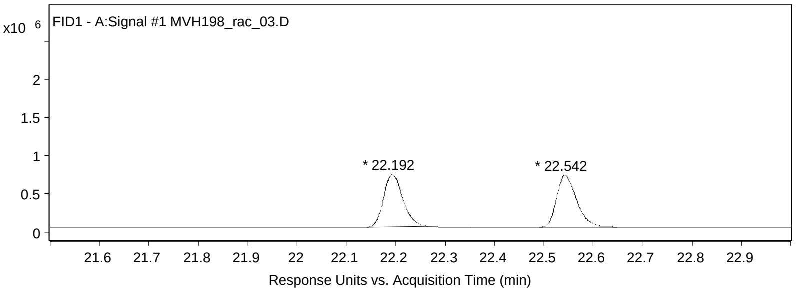

Integration Peak List

Peak	Start	RT	End	Height	Height %	Area	Area %	Area sum %
1	22.141	22.192	22.283	690951.92	100	1887619	98.87	49.71
2	22.491	22.542	22.649	686019.58	99.29	1909280.15	100	50.29

Enantiomerically enriched product:

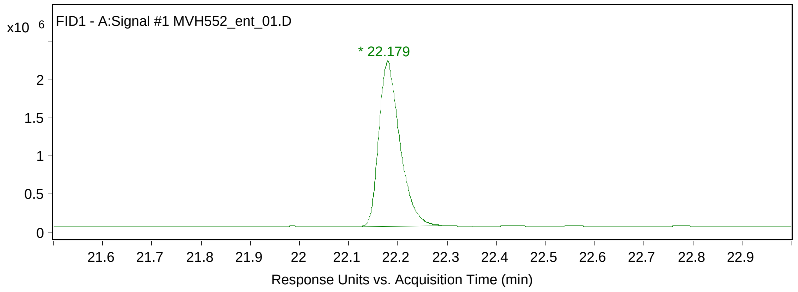

Integration Peak List

Peak	Start	RT	End	Height	Height %	Area	Area %	Area sum %
1	22.127	22.179	22.29	2167998.16	100	6431433.55	100	100

Silylether 14

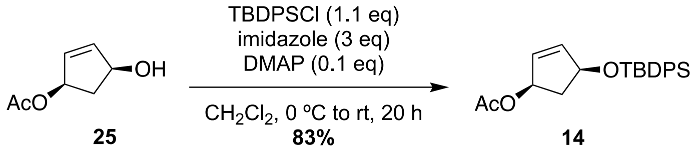

A flask was placed under N_2 atmosphere by three cycles of evacuating and N_2 backfilling and charged with imidazole (1.42 g, 20.9 mmol, 3.0 eq.) and DMAP (84.9 mg, 0.70 mmol, 0.1 eq.) under N_2 flow. Acetate **25** (986 mg, 6.94 mmol, 1.0 eq.) was added dissolved in dry CH_2Cl_2 (28 mL) and the resulting solution was cooled to 0 °C. TBDPSCl (2.0 mL, 7.69 mmol, 1.1 eq.) was added. After stirring for 15 min, the ice bath was removed and the reaction mixture was stirred for 20 h at room temperature. Sat. aq. NH_4Cl (50 mL) was added, the layers were separated and the aqueous phase was extracted with CH_2Cl_2 (2 x 30 mL). The combined organic layers were dried over $MgSO_4$ and concentrated. The crude was purified by flash chromatography (pentane/Et_2O 95/5) to give the product **14** (2.18 g, 5.72 mmol, 83% yield) as colourless oil.

^{1}H NMR (400 MHz, Chloroform-*d*) δ 7.73 – 7.67 (m, 4H), 7.47 – 7.37 (m, 6H), 5.94 – 5.91 (m, 1H), 5.88 – 5.83 (m, 1H), 5.42 – 5.35 (m, 1H), 4.73 – 4.67 (m, 1H), 2.66 (dtd, *J* = 13.8, 7.4, 0.8 Hz, 1H), 2.08 (s, 3H), 1.76 (dtd, *J* = 13.8, 5.0, 0.7 Hz, 1H), 1.08 (s, 9H).

^{13}C NMR (101 MHz, Chloroform-*d*) δ 170.96, 138.86, 135.86, 135.84, 134.14, 133.97, 131.39, 129.87, 129.84, 127.81, 127.79, 76.93, 75.81, 41.10, 27.00, 21.33, 19.21.

HRMS (ESI+) M+H$^+$ calculated for $C_{23}H_{28}O_3Si$: 381.1881, found: 381.1884.

HRMS (ESI+) M+Na$^+$ calculated for $C_{23}H_{28}O_3Si$: 403.1670, found: 403.1707.

Optical Rotation: $[\alpha]_D^{23}$ = +21.4° (*c* = 0.87, $CHCl_3$).

Preparation of Ligands L1 and L2

Diamine (±)-S21

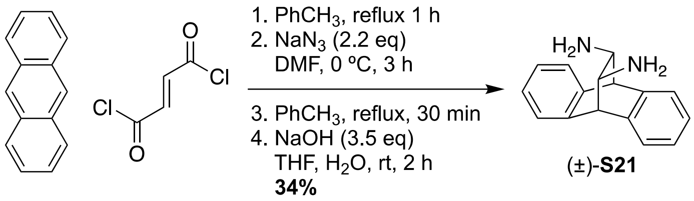

A flask was placed under N_2 atmosphere by three cycles of evacuating and N_2 backfilling and charged with anthracene (4.50 g, 25.0 mmol, 1 eq.) under N_2 flow. Dry toluene (7.5 mL) was added and to the resulting suspension was added dropwise fumaryl chloride (2.84 mL, 25.0 mmol, 1 eq.). The resulting bright red solution was heated to reflux for 1 h. After cooling to room temperature, a small amount of Et_2O was added (~1-2 mL) upon which crystals formed. The crystals were filtered off, dried *in vacuo*, dissolved in dry DMF (60 mL) under N_2 atmosphere and cooled to 0 ºC. Solid sodium azide (3.58 g, 55.1 mmol, 2.2 eq.) was added under N_2 flow. The reaction mixture was stirred for 3 h at 0 ºC and then poured onto ice water (350 mL). The aqueous phase was extracted with cold toluene (3 x 100 mL), dried over $MgSO_4$. The toluene layer was then heated to reflux for 30 min (caution: N_2 evolution). After cooling

to room temperature, the solvent was evaporated and the residue was taken up in THF (75 mL). 2 M aq. NaOH (44 mL, 88.0 mmol, 3.5 eq.) was added and the reaction mixture was stirred for 2 h at room temperature. The reaction mixture was then acidified to pH <1 by dropwise addition of conc. HCl. The aqueous phase was washed with Et_2O (2 x 80 mL). The aqueous layer was then treated carefully with NaOH pellets until pH 12 was reached and then extracted with CH_2Cl_2 (2 x 100 mL), dried over $MgSO_4$ and concentrated. The product (±)-**S21** was obtained as pale yellow thick oil (1.98 g, 8.40 mmol, 34% yield).

^{1}H NMR (400 MHz, Chloroform-*d*) δ 7.37 – 7.27 (m, 4H), 7.19 – 7.10 (m, 4H), 4.06 – 3.99 (m, 2H), 2.69 – 2.63 (m, 2H), 1.44 (s, 4H).

^{13}C NMR (101 MHz, Chloroform-*d*) δ 142.13, 138.96, 126.58, 126.39, 126.22, 124.21, 62.24, 53.15.

The analytical data is in agreement with previous reports.[14]

Diamine S21

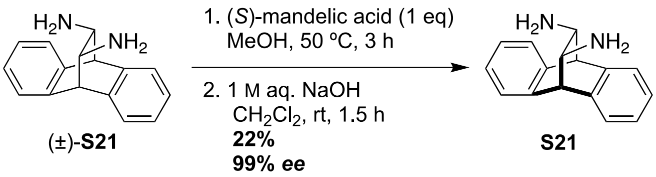

A flask was charged with (±)-**S21** (1.98 g, 8.40 mmol, 1 eq.) and dissolved in methanol (42 mL). (*S*)-Mandelic acid (1.28 g, 8.41 mmol, 1 eq.) was added and the reaction mixture was stirred for 3 h at 50 ºC. The resulting suspension was cooled to 0 ºC, the solids were filtered off, washed with methanol and dried *in vacuo*. The mandelate salt was suspended in 1 M aq. NaOH (13 mL) and stirred for 90 min at room temperature. Then CH_2Cl_2 (15 mL) was added and the biphasic system was stirred for 1 h until two clear layers were obtained. The layers were separated and the aqeous phase was extracted with CH_2Cl_2 (2 x 20 mL). The combined organic phases were dried over $MgSO_4$ and concentrated to give the product **S21** (438 mg, 1.85 mmol, 22% yield) as an off-white solid.

Determination of the enantiomeric excess by derivatization

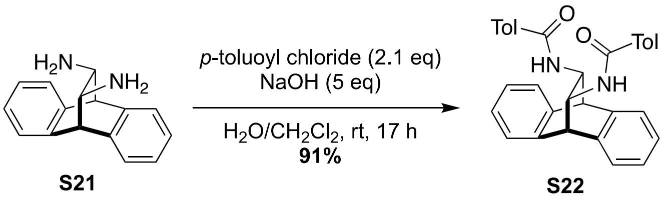

A flask was charged with **S21** (50.0 mg, 0.21 mmol, 1 eq.), water (3 mL) was added followed by solid NaOH (42.3 mg, 1.06 mmol, 5 eq.) and the mixture was stirred vigorously until dissolved. A solution of *p*-toluoyl chloride (0.06 mL, 0.45 mmol, 2.1 eq.) in CH_2Cl_2 (3 mL) was added and the reaction mixture was stirred for 17 h at room temperature. Then water (10 mL) was added and the aqueous phase was extracted with CH_2Cl_2 (2 x 10 mL). The combined organic extracts were dried over $MgSO_4$ and concentrated. The crude product was purified by flash chromatography (pentane/EtOAc 3/1) to give the product **S22** (90.5 mg, 0.19 mmol, 91%) as white solid.

The enantiomeric excess was determined by chiral HPLC

Chiracel OD-H column, *n*-heptane/*i*-PrOH = 93/7, 40 °C, flow = 0.5 mL/min, UV detection at 220 nm and 254 nm, retention times for racemate (min): 11.3 (major) and 14.9 (minor).

The enantiomeric excess was determined to be 99%.

Racemate

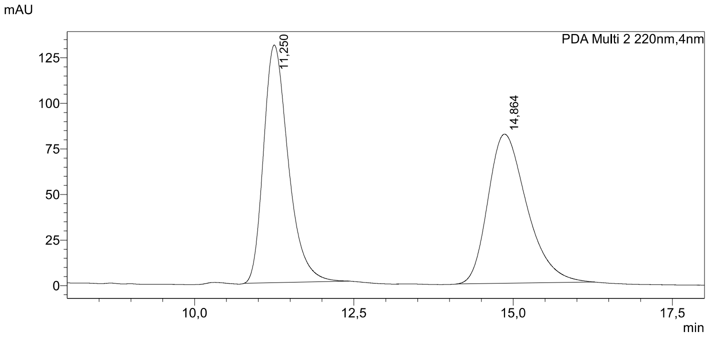

PDA Ch2 220nm

Peak#	Ret. Time	Area	Area%
1	11,250	3528469	50,335
2	14,864	3481443	49,665
Total		7009912	100,000

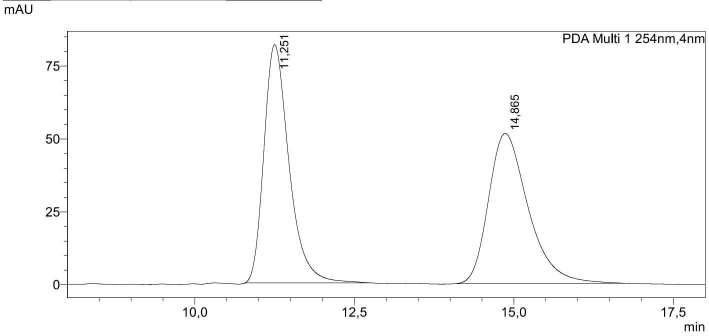

PDA Ch1 254nm

Peak#	Ret. Time	Area	Area%
1	11,251	2243469	50,199
2	14,865	2225682	49,801
Total		4469151	100,000

Enantiomerically enriched product

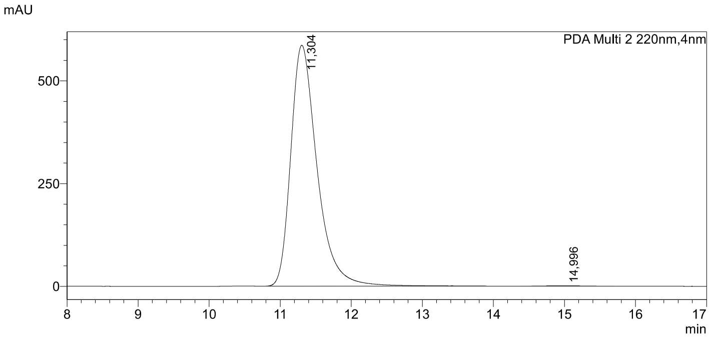

PDA Ch2 220nm

Peak#	Ret. Time	Area	Area%
1	11,304	15074669	99,763
2	14,996	35743	0,237
Total		15110412	100,000

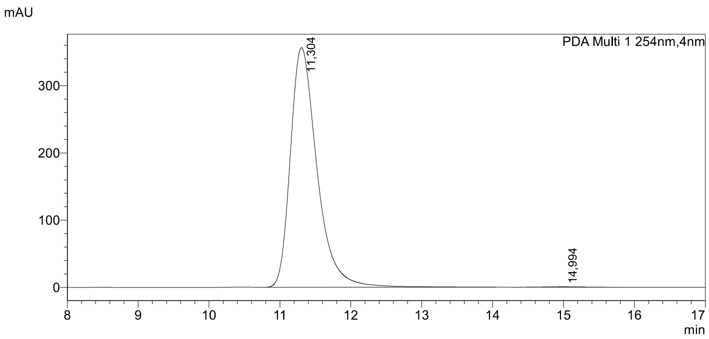

PDA Ch1 254nm

Peak#	Ret. Time	Area	Area%
1	11,304	9309178	99,770
2	14,994	21422	0,230
Total		9330600	100,000

(*R*,*R*)-Phenyl-ANDEN-Trost L1

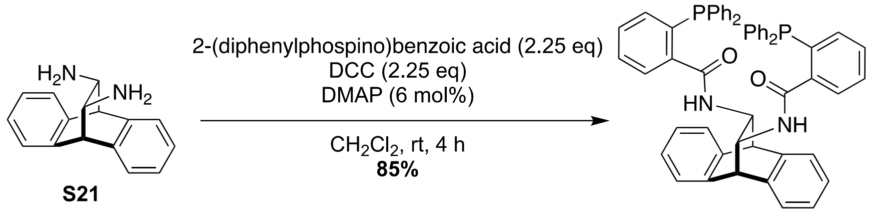

(*R*,*R*)-Phenyl-ANDEN-Trost L1

A flask was placed under N_2 atmosphere by three cycles of evacuating and N_2 backfilling and charged with **S21** (383 mg, 1.62 mmol, 1 eq.), DMAP (11.6 mg, 0.10 mmol, 0.06 eq.) and 2-(diphenylphospino)benzoic acid (1.07 g, 3.65 mmol, 2.25 eq.) under N_2 flow and dissolved in dry CH_2Cl_2 (8 mL). To the solution was added DCC (752 mg, 3.65 mmol, 2.25 eq.) and the reaction mixture was stirred at room temperature for 5 h. The suspension was filtered over Celite and the filtrate was concentrated. The crude product was purified by flash chromatography (pentane/EtOAc 3/1) to give the product **L1** (1.18 g, 1.38 mmol, 85% yield) as white solid.

^{1}H NMR (400 MHz, Chloroform-*d*) δ 7.48 – 7.43 (m, 2H), 7.40 – 7.25 (m, 26H), 7.20 – 7.12 (m, 4H), 7.07 (t, *J* = 7.3 Hz, 2H), 6.97 (t, *J* = 6.0 Hz, 2H), 5.82 (d, *J* = 7.1 Hz, 2H), 4.44 (s, 2H), 3.94 (d, *J* = 6.8 Hz, 2H).

^{13}C NMR (101 MHz, Chloroform-*d*) δ 168.86, 141.27, 140.99, 138.81, 134.65, 134.08, 133.94, 133.88, 133.74, 130.47, 129.14, 128.99, 128.90, 128.82, 128.79, 128.75, 128.71, 128.68, 128.64, 127.76, 127.71, 126.82, 126.71, 126.13, 124.90, 57.83, 48.73.

HRMS (ESI+) M+H$^+$ calculated for $C_{54}H_{42}N_2O_2P_2$: 813.2794, found: 813.2782.

Optical Rotation: $[\alpha]_D^{23}$ = -102.6° (*c* = 3.37, CH_2Cl_2).

The analytical data is in agreement with previous reports.[15]

Bromide S25

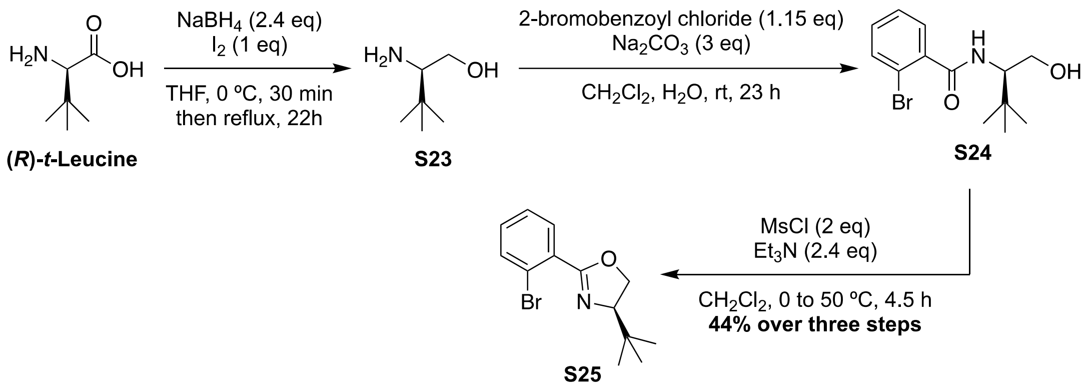

A 3-neck flask equipped with a reflux condenser was flame-dried *in vacuo* and subjected to three cycles of evacuating and N_2 backfilling. The flask was charged with (*R*)-*t*-leucine (5.0 g, 38.0 mmol, 1 eq.) under N_2 flow. Dry THF (100 mL) was added and the resulting suspension was cooled to 0 °C. Solid sodium borohydride (3.49 g, 91.3 mmol, 2.4 eq.) was added followed by dropwise addition of a solution of iodine (9.67 g, 38.0 mmol, 1 eq.) in dry THF (25 mL) over 25 min. After complete addition, the dropping funnel was rinsed with dry THF (3 mL) and the reaction mixture was heated to 80 °C and stirred for 22 h at 80 °C. After cooling to room temperature, the reaction was quenched carefully by dropwise addition of methanol. The solvent was evaporated and the residue was taken up in 20% aq. NaOH (75 mL) and stirred for 19 h at room temperature. The aqueous phase was extracted with CH_2Cl_2 (6 x 60 mL) and the combined organic extracts were dried over $MgSO_4$ and concentrated. The crude alcohol **S23** was used without further purification.

^{1}H NMR (400 MHz, Chloroform-*d*) δ 3.70 (dd, *J* = 10.3, 3.9 Hz, 1H), 3.20 (t, *J* = 10.2 Hz, 1H), 2.50 (dd, *J* = 10.1, 3.8 Hz, 1H), 0.89 (s, 9H).

A flask was charged with Na_2CO_3 (11.8 g, 112 mmol, 3 eq.) and dissolved in water (95 mL). To this, a solution of crude alcohol **S23** (4.37 g, 37.3 mmol, 1 eq.) in CH_2Cl_2 was added followed by addition of 2-bromobenzoyl chloride (5.6 mL, 42.8 mmol, 1.15 eq.). The reaction mixture was stirred for 23 h at room temperature. The layers were separated and the aqueous phase was extracted with CH_2Cl_2 (4 x 50 mL). To the combined organic extracts was added 1 M KOH in methanol (19 mL) and the mixture was stirred for 30 min and then neutralized with 1 M aq. HCl. Water (25 mL) was added, the layers were separated and the aqueous phase was extracted with CH_2Cl_2 (4 x 35 mL). The combined organic layers were washed with brine (100 mL), dried over $MgSO_4$ and concentrated. The crude product **S24** was used without further purification.

^{1}H NMR (400 MHz, Chloroform-*d*) δ 7.59 (ddd, *J* = 9.3, 7.8, 1.5 Hz, 2H), 7.38 (td, *J* = 7.5, 1.2 Hz, 1H), 7.29 (td, *J* = 7.7, 1.8 Hz, 1H), 6.15 (d, *J* = 9.4 Hz, 1H), 4.08 (ddd, *J* = 9.3, 7.5, 3.5 Hz, 1H), 3.97 (dd, *J* = 11.4, 3.5 Hz, 1H), 3.70 (dd, *J* = 11.4, 7.5 Hz, 1H), 2.23 (br s, 1H), 1.05 (s, 9H).

A 3-neck flask equipped with a reflux condenser was flame-dried *in vacuo* and subjected to three cycles of evacuating and N_2 backfilling. The flask was charged with crude **S24** dissolved in dry CH_2Cl_2 (170 mL) and cooled to 0 °C. Dry triethylamine (12.5 mL, 89.9 mmol, 2.4 eq.) and mesyl chloride (6.0 mL, 77.5 mmol, 2.0 eq.) were added and the reaction mixture was heated to 50 °C and stirred for 4.5 h. After cooling to room temperature, sat. aq. $NaHCO_3$ (60 mL) was added, the layers were separated and the aqueous phase was extracted with CH_2Cl_2 (2 x 40 mL). The combined organic phases were washed with brine (100 mL), dried over $MgSO_4$ and concentrated. The

crude product was purified by flash chromatography (pentane/Et_2O 4/1) to give the product **S25** (4.62 g, 16.4 mmol, 44% yield over three steps) as off-white solid.

^{1}H NMR (400 MHz, Chloroform-*d*) δ 7.65 (ddt, *J* = 13.6, 7.9, 1.6 Hz, 2H), 7.33 (tt, *J* = 7.5, 1.4 Hz, 1H), 7.27 (dtd, *J* = 7.7, 4.4, 3.9, 1.7 Hz, 1H), 4.39 (ddd, *J* = 10.1, 8.6, 1.3 Hz, 1H), 4.26 (td, *J* = 8.3, 1.4 Hz, 1H), 4.11 (dd, *J* = 10.1, 8.1 Hz, 1H), 1.00 (s, 9H).

The analytical data is in agreement with previous reports.[16]

(*R*)-*t*-ButylPHOX L2

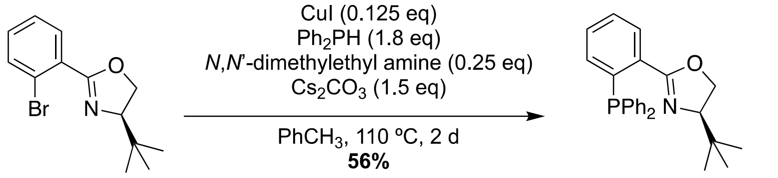

S25 **(*R*)-*t*-ButylPHOX L2**

A 2-neck flask was flame-dried *in vacuo* and subjected to three cycles of evacuating and N_2 backfilling. The flask was charged with copper iodide (390.7 mg, 2.05 mmol, 0.125 eq.) under N_2 flow. Dry toluene (17 mL) was added followed by addition of *N*,*N*'-dimethylethyl amine (0.46 mL, 4.15 mmol, 0.25 eq.) and diphenylphosphine (5.2 mL, 29.9 mmol, 1.8 eq.). The mixture was stirred for 40 min at room temperature and then Cs_2CO_3 (8.04 g, 24.7 mmol, 1.5 eq.) was added followed by **S25** (4.62 g, 16.4 mmol, 1 eq.) dissolved in dry toluene (17 mL). The reaction mixture was heated to 110 ºC and stirred for 48 h. After cooling to room temperature, the reaction mixture was filtered over Celite and the filtrate was concentrated. The crude product was purified by flash chromatography (pentane/Et_2O 25/1 to CH_2Cl_2/Et_2O 9/1) to give the product **L2** (3.54 g, 9.13 mmol, 56% yield) as white solid.

^{1}H NMR (400 MHz, Chloroform-*d*) δ 7.95 (dd, *J* = 8.0, 3.5 Hz, 1H), 7.39 – 7.19 (m, 12H), 6.91 – 6.85 (m, 1H), 4.09 (ddd, *J* = 10.0, 8.4, 1.2 Hz, 1H), 4.05 – 3.99 (m, 1H), 3.96 – 3.83 (m, 1H), 0.73 (s, 9H).

^{13}C NMR (101 MHz, Chloroform-*d*) δ 162.82, 139.06, 138.80, 138.73, 138.60, 138.44, 138.34, 134.53, 134.32, 134.22, 133.79, 133.59, 132.12, 131.92, 130.46, 129.97, 129.94, 128.60, 128.51, 128.44, 128.39, 128.33, 128.32, 128.13, 76.78, 68.39, 33.71, 25.86.

HRMS (ESI+) M+H$^+$ calculated for $C_{25}H_{26}NOP$: 388.1825, found: 388.1834.

Optical Rotation (after enrichment): $[\alpha]_D^{23}$ = +53.0° (*c* = 0.925, $CHCl_3$).

The analytical data is in agreement with previous reports.[16]

Preparation of Ketones 27 and 28

2,2-Dimethylcyclohexanone 28

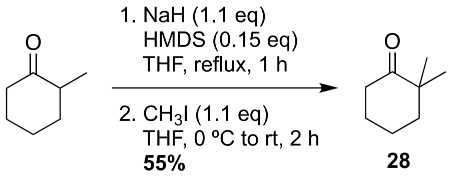

A 3-neck flask equipped with a reflux condenser was flame-dried under vacuo and subjected to three cycles of evacuating and N_2 backfilling. The flask was charged with NaH (60% in mineral oil, 1.79 g, 44.9 mmol, 1.1 eq.) under N_2 flow. Dry THF (40 mL) was added and to the resulting suspension was added 2-methylcyclohexanone (5.0 mL, 40.8 mmol, 1 eq.). The reaction mixture was heated to reflux for one h. Hexamethyldisilazane (1.3 mL, 6.16 mmol, 0.15 eq.) was added and after 15 min stirring at reflux, the reaction mixture was cooled to 0 ºC. Iodomethane (2.8 mL, 44.5 mmol, 1.1 eq.) was added dropwise over 10 min and after addition the cooling bath was removed and the reaction mixture was stirred at room temperature for 2 h. The solvent was evaporated (40 ºC, 210 mbar), Et_2O (100 mL) was added and the resulting solids were filtered off. The filtrate was concentrated (40 ºC, 210 mbar). The crude was purified by flash chromatography (100% pentane to pentane/Et_2O 95/5 to 9/1) to give the product **28** (2.84 g, 22.5 mmol, 55% yield) as colourless liquid.

^{1}H NMR (400 MHz, Chloroform-*d*) δ 2.55 – 2.49 (m, 2H), 2.01 – 1.92 (m, 2H), 1.90 – 1.83 (m, 2H), 1.81 – 1.76 (m, 2H), 1.24 (s, 6H).

^{13}C NMR (101 MHz, Chloroform-*d*) δ 216.44, 45.42, 41.29, 38.50, 27.77, 25.36, 25.36, 21.60.

HRMS (ESI+) M+H$^+$ calculated for $C_8H_{14}O$: 127.1117, found: 127.1116.

The analytical data is in agreement with previous reports.[17]

rac-ketone S27

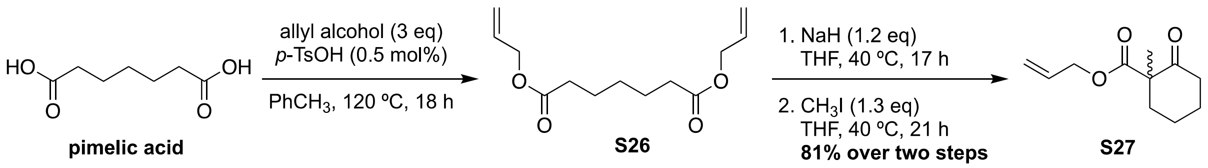

A flask was charged with pimelic acid (50.5 g, 315 mmol, 1 eq.) and suspended in toluene (157 mL). Allyl alcohol (65.3 mL, 946 mmol, 3 eq.) and *p*-TsOH·H_2O (320 mg, 1.68 mmol, 0.005 eq.) was added. The flask was equipped with a Dean-Stark-trap and a condenser. The reaction mixture was then heated to 120 °C and stirred for 18 h. After cooling to room temperature, the organic phase was washed with sat. aq. $NaHCO_3$ (2 x 30 mL) and brine (2 x 30 mL), dried over $MgSO_4$ and concentrated to give the product **S26** (75.7 g, 315 mmol, quant. yield) as pale yellow liquid.

^{1}H NMR (400 MHz, Chloroform-*d*) δ 5.91 (ddt, *J* = 16.2, 10.5, 5.7 Hz, 2H), 5.30 (d, *J* = 17.2 Hz, 2H), 5.22 (d, *J* = 10.4 Hz, 2H), 4.57 (d, *J* = 5.6 Hz, 4H), 2.33 (t, *J* = 7.4 Hz, 4H), 1.65 (p, *J* = 7.5 Hz, 4H), 1.37 (p, *J* = 7.7, 7.2 Hz, 2H).

^{13}C NMR (101 MHz, Chloroform-*d*) δ 173.34, 132.41, 118.29, 65.13, 34.14, 28.72, 24.69.

The analytical data is in agreement with previous reports.[18]

A flask was flame-dried under vacuo and subjected to three cycles of evacuating and N_2 backfilling. The flask was charged with NaH (60% in mineral oil, 15.1 g, 378 mmol, 1.2 eq.) and suspended in dry THF (260 mL). The flask was placed in a water bath and **S26** (75.7 g, 315 mmol, 1.0 eq.) dissolved in dry THF (50 mL) was added dropwise over 15 min. The reaction was then heated to 40 °C and stirred for 17 h. The reaction mixture was cooled to room temperature and then iodomethane (25.5 mL, 410 mmol, 1.3 eq.) was added. Stirring was then continued at 40 °C for 23 h. The reaction was cooled to room temperature and quenched by addition of water (60 mL). The organic solvent was evaporated and the residual aqueous phase was extracted with EtOAc (4 x 75 mL). The combined organic extracts were washed with brine (100 mL), dried over $MgSO_4$ and concentrated. The crude was purified by short path distillation (heating bath temp. 126-135 °C, 3.5 mbar, vapour temp. 82-92 °C) to give the product **S27** (49.8 g, 254 mmol, 81% yield) as colourless liquid.

^{1}H NMR (400 MHz, Chloroform-*d*) δ 5.95 – 5.81 (m, 1H), 5.30 (dd, *J* = 17.2, 1.3 Hz, 1H), 5.23 (dd, *J* = 10.5, 1.2 Hz, 1H), 4.65 – 4.57 (m, 2H), 2.55 – 2.43 (m, 3H), 2.05 – 1.96 (m, 1H), 1.76 – 1.62 (m, 3H), 1.50 – 1.42 (m, 1H), 1.30 (s, 3H).

^{13}C NMR (101 MHz, Chloroform-*d*) δ 208.23, 172.89, 131.66, 118.97, 65.88, 57.31, 40.77, 38.36, 27.62, 22.72, 21.39.

HRMS (ESI+) $M+H^+$ calculated for $C_{11}H_{16}O_3$: 197.1172, found: 197.1174.

The analytical data is in agreement with previous reports.[18]

(*R*)-2-allyl-2-methylcyclohexanone 13

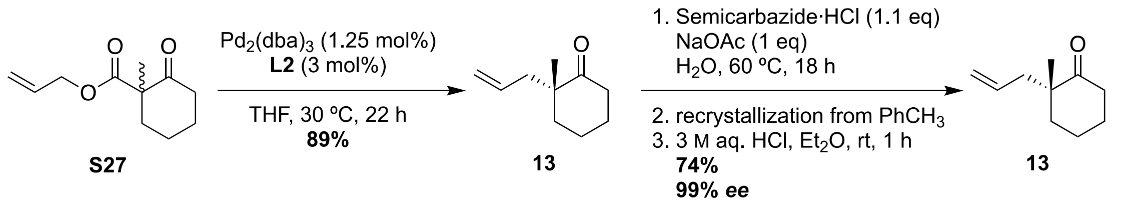

A 2-neck flask was flame-dried under vacuo and subjected to three cycles of evacuating and N_2 backfilling. The flask was filled with dry THF (500 mL) and Argon was bubbled through the solvent for 1 h. Then $Pd_2(dba)_3$ (583 mg, 0.64 mmol, 0.012 eq.) and (*R*)-*t*-Bu-PHOX **L1** (596 mg, 1.54 mmol, 0.03 eq.) were added as solids and the solution was heated to 30 °C and stirred for 1 h. Then **S27** (10.0 g, 51.0 mmol, 1.0 eq.) was added neat over 10 min. The reaction mixture was then stirred at 30 °C for 21 h. The heating was stopped and the reaction mixture was filtered over a pad of Celite (5 cm diameter, 5 cm length), the filter cake was rinsed with Et_2O (approx. 150 mL) and the filtrate was concentrated. The crude was purified by flash chromatography (pentane/Et_2O 9/1) to give the product **13** (7.22 g, 45.4 mmol, 96% purity by wt., 89% yield).

^{1}H NMR (400 MHz, Chloroform-*d*) δ 5.69 (ddt, *J* = 15.9, 11.3, 7.4 Hz, 1H), 5.08 – 4.99 (m, 2H), 2.43 – 2.30 (m, 3H), 2.22 (dd, *J* = 13.9, 7.3 Hz, 1H), 1.88 – 1.67 (m, 5H), 1.62 – 1.53 (m, 1H), 1.06 (s, 3H).

^{13}C NMR (101 MHz, Chloroform-*d*) δ 215.48, 133.94, 118.00, 48.55, 42.09, 38.93, 38.72, 27.51, 22.78, 21.19.

HRMS (ESI+) $M+H^+$ calculated for $C_{10}H_{16}O$: 153.1274, found: 153.1275.

Optical Rotation (after enrichment): $[\alpha]_D^{23}$ = +51.4° (*c* = 0.98, $CHCl_3$).

The analytical data is in agreement with previous reports.[18]

Enantioenrichment

A flask was charged with NaOAc (3.65 g, 44.5 mmol, 1.0 eq.) and semicarbazide·HCl (5.46 g, 49.0 mmol, 1.1 eq.) and dissolved in water (49 mL). Then neat **13** (7.08 g, 44.5 mmol, 1.0 eq.) was added and the reaction mixture was stirred at 60 °C for 16 h. The reaction was cooled to room temperature and the solids were filtered off, washed with water (4 x 15 mL) and dried under vacuo at 50 °C for 6 h. The dried solids were then recrystallized from toluene (100 mL) by dissolving at 110 °C and cooling down slowly while stirring at approx. 400 rpm. The solids were then filtered off to give white crystals (7.05 g). The crystalline solid was then suspended in Et_2O (30 mL) and 3 M aq. HCl (15 mL) was added and the resulting biphasic system was stirred until both layers were clear. The layers were separated and the aqueous phase was extracted (3 x 10 mL). The combined organic phases were washed with sat. aq. $NaHCO_3$ (2 x 5 mL), water (10 mL), brine (10 mL), dried over $MgSO_4$ and concentrated to give the enantioenriched product **13** (5.22 g, 32.8 mmol, 96% purity by wt., 74% yield) as colourless liquid in >99% *ee*.

The enantiomeric excess was determined by chiral GC

A GC instrument equipped with a FS-Hydrodex-beta-3P column (25.0 m × 0.25 mm x 0.25 µm) and a flame ionization detector. The conditions for 2-allyl-2-methylcyclohexanone are 40 °C to 100 °C (ramp 0.4 °C/min) and back to 40 °C (10 °C/min), 69.1 kPa H_2 carrier gas, split ratio 50:1. Retention times racemate (min): 110.3 (*S*)-enantiomer), 111.2 (*R*)-enantiomer).

The enantiomeric excess was determined to be >99%, no (*S*)-enantiomer was detected.

Racemate

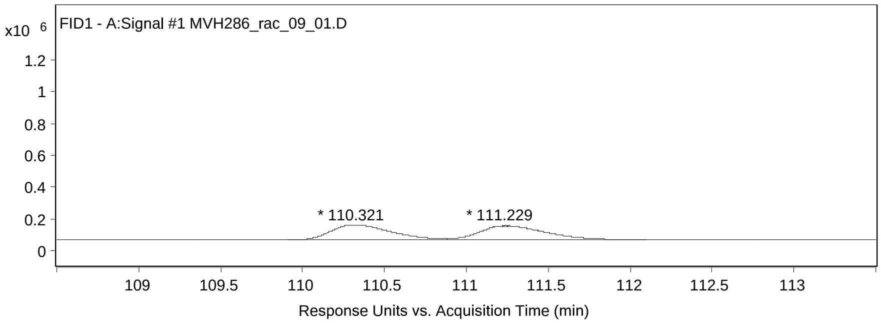

Integration Peak List

Peak	Start	RT	End	Height	Height %	Area	Area %	Area sum %
1	109.909	110.321	110.882	97297.02	100	2503938.29	100	50
2	110.882	111.229	112.101	89993.52	92.49	2503891.72	100	50

Enantiomerically enriched product **13**

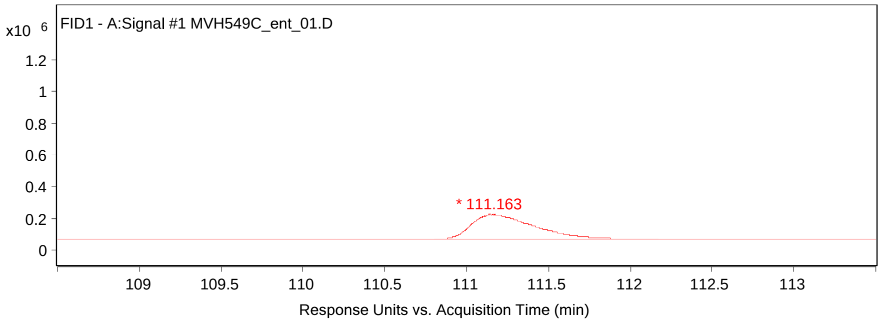

Integration Peak List

Peak	Start	RT	End	Height	Height %	Area	Area %	Area sum %
1	110.8	111.163	112.172	159558.26	100	4252375.45	100	100

Alcohol 26

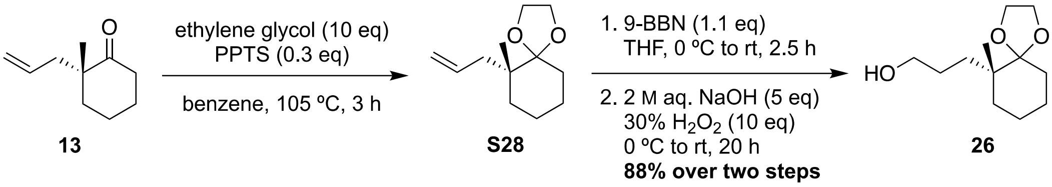

A flask was charged with **13** (4.28 g, 27.1 mmol, 1.0 eq.) and dissolved in benzene (54 mL). Ethylene glycol (15.2 mL, 271 mmol, 10 eq.) and PPTS (2.05 g, 8.15 mmol, 0.3 eq.) were added and the flask was equipped with a Dean-Stark trap and a condenser. The reaction was stirred at 105 °C for 3 h. After cooling to room temperature, the reaction mixture was poured onto cold sat. aq. $NaHCO_3$ (80 mL). The aqueous phase was extracted with Et_2O (2 x 50 mL) and the combined organic extracts were washed with brine (80 mL), dried over $MgSO_4$ and concentrated. The crude colourless liquid **S28** (6.75 g, containing small amounts of benzene) was used in the next step without further purification.

^{1}H NMR (400 MHz, Chloroform-*d*) δ 5.86 – 5.73 (m, 1H), 5.03 – 5.00 (m, 1H), 4.99 – 4.96 (m, 1H), 3.97 – 3.84 (m, 4H), 2.23 (dd, J = 13.7, 7.6 Hz, 1H), 2.14 (ddt, J = 13.6, 7.5, 1.2 Hz, 1H), 1.62 – 1.52 (m, 4H), 1.48 – 1.34 (m, 4H), 0.91 (s, 3H).

^{13}C NMR (101 MHz, Chloroform-*d*) δ 135.71, 116.74, 112.79, 65.08, 64.89, 41.59, 39.50, 34.57, 30.76, 23.77, 20.90, 19.67.

Optical Rotation: $[\alpha]_D^{23}$ = -10.0° (c = 0.60, $CHCl_3$).

A Schlenk flask was flame-dried under vacuo and subjected to three cycles of evacuating and N_2 backfilling. The flask was charged with **S28** (5.32 g, 27.1 mmol, 1.0 eq.) dissolved in dry THF (75 mL) and cooled to 0 °C. 0.5 M 9-BBN in THF (60 mL, 30.0 mmol, 1.1 eq.) was added over 5 min. After stirring 30 min at 0 °C, the reaction was stirred for 2 h at room temperature. The reaction was quenched after cooling to 0 °C by addition of 2 M aq. NaOH (68 mL, 136 mmol, 5.0 eq.) followed by 30% aq. H_2O_2 (26 mL, 274 mmol, 10 eq.) and then stirred at room temperature for 20 h. Sat. aq. NH_4Cl (75 mL) was added and the layers were separated. The aqueous phase was extracted with Et_2O (2 x 100 mL). The combined organic layers were washed with brine (100 mL), dried over $MgSO_4$ and concentrated. The crude was purified by flash chromatography (pentane/Et_2O 1/1) to give the product **26** (5.13 g, 23.9 mmol, 88% yield) as colourless liquid.

^{1}H NMR (400 MHz, Chloroform-*d*) δ 3.96 – 3.81 (m, 4H), 3.62 – 3.52 (m, 2H), 1.99 (s, 1H), 1.63 – 1.33 (m, 12H), 0.87 (s, 3H).

^{13}C NMR (101 MHz, Chloroform-*d*) δ 113.08, 64.99, 64.79, 63.96, 41.04, 34.32, 30.54, 30.42, 27.04, 23.66, 20.91, 19.51.

HRMS (ESI+) M+Na^{+} calculated for $C_{12}H_{22}O_3$: 237.1461, found: 237.1458.

Optical Rotation: $[\alpha]_D^{23}$ = -0.2° (*c* = 0.76, $CHCl_3$).

Benzyl ether S29

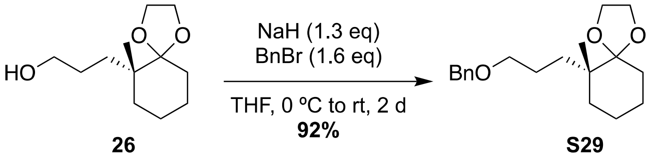

26 S29

A Schlenk flask was flame-dried under vacuo and subjected to three cycles of evacuating and N_2 backfilling. The flask was charged with NaH (60% in mineral oil, 1.23 g, 30.7 mmol, 1.3 eq.) under N_2 flow. NaH was washed with pentane (3 x 5 mL), dried under vacuo and, then suspended in dry THF (30 mL) and cooled to 0 °C. Alcohol **26** (5.06 g, 23.6 mmol, 1.0 eq.) was added dissolved in dry THF (15 mL). After stirring for 15 min at 0 °C, benzyl bromide (3.9 mL, 32.8 mmol, 1.4 eq.) was added. The ice bath was removed and the reaction mixture was stirred at room temperature for 18 h. When NMR sampling showed that still starting material was present, another portion of benzyl bromide (0.6 mL, 5.05 mmol, 0.2 eq.) was added. The reaction was stirred for 23 h, then quenched by addition of sat. aq. NH_4Cl (75 mL) and the layers were separated. The aqueous phase was extracted with Et_2O (2 x 75 mL) and the combined organic phases were washed with brine (50 mL), dried over $MgSO_4$ and concentrated. The crude was purified by flash chromatography (pentane 100% to pentane/Et_2O 9/1) to give the product **S29** (6.59 g, 21.7, 92% yield) as colourless liquid.

^{1}H NMR (400 MHz, Chloroform-*d*) δ 7.38 – 7.31 (m, 4H), 7.30 – 7.26 (m, 1H), 4.51 (s, 2H), 3.98 – 3.86 (m, 4H), 3.50 – 3.41 (m, 2H), 1.68 – 1.36 (m, 12H), 0.92 (s, 3H).

^{13}C NMR (101 MHz, Chloroform-*d*) δ 138.84, 128.48, 128.44, 127.75, 127.56, 113.13, 72.97, 71.71, 65.08, 64.88, 41.18, 34.36, 30.77, 30.66, 24.09, 23.76, 21.00, 19.55.

HRMS (ESI+) M+H^{+} calculated for $C_{19}H_{28}O_3$: 305.2111, found: 305.2111.

Optical Rotation: $[\alpha]_D^{23}$ = +5.5° (*c* = 0.84, $CHCl_3$).

Ketone 27

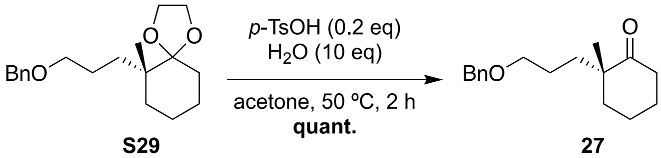

S29 27

A flask was charged with **S29** (6.54 g, 21.5 mmol, 1.0 eq.) and *p*-TsOH·H_2O (817 mg, 4.29 mmol, 0.2 eq.). The reagents were dissolved in acetone (215 mL) and water (3.9 mL, 216 mmol, 10 eq.) was added. The reaction mixture was heated to 50 °C and stirred for 2 h. The solvent was evaporated and the residue was taken up in Et_2O (150

mL) and the organic phase washed with sat. aq. $NaHCO_3$ (2 x 100 mL), dried over $MgSO_4$ and concentrated to give the product **27** (5.59 g, 21.5 mmol, quant. yield) as colourless liquid.

^{1}H NMR (400 MHz, Chloroform-*d*) δ 7.37 – 7.30 (m, 4H), 7.30 – 7.24 (m, 1H), 4.49 (s, 2H), 3.45 (t, *J* = 6.2 Hz, 2H), 2.45 – 2.29 (m, 2H), 1.93 – 1.83 (m, 1H), 1.82 – 1.54 (m, 7H), 1.52 – 1.34 (m, 2H), 1.05 (s, 3H).

^{13}C NMR (101 MHz, Chloroform-*d*) δ 216.07, 216.07, 138.68, 128.47, 127.72, 127.63, 72.95, 70.75, 48.45, 39.44, 38.87, 34.15, 27.63, 24.32, 22.66, 21.16.

HRMS (ESI+) $M+H^+$ calculated for $C_{17}H_{24}O_2$: 261.1849, found: 261.1848.

Optical Rotation: $[\alpha]_D^{23}$ = +46.9° (*c* = 0.91, $CHCl_3$).

Palladium cataylzed intermolecular allylic alkylation

Reaction optimization

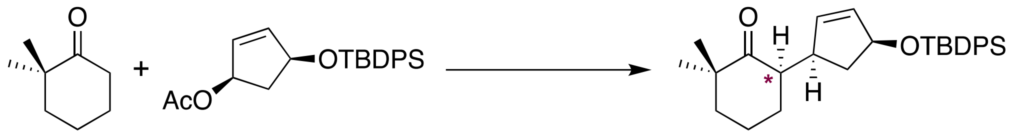

28 **14** **29**

Entry[a]	Pd-cat.	Ligand	Base	Temp.	Solvent	Time	Conv..[b] (yield[c])	Additive	*dr*[d]
**1**	2.6 mol%	**L1** (6 mol%)	LDA (1.1 eq)	rt[e]	THF	5 h	68% (46%)	–	38:62
**2**	2.6 mol%	**L1** (6 mol%)	LDA (1.1 eq)	0 ºC	THF	2 h	54% (35%)	–	24:76
**3**	5 mol%	**L1** (11 mol%)	LHMDS (1.1 eq)	0 ºC	THF	2 h	80%	–	25:75
**4**	5 mol%	**L2** (11 mol%)	LHMDS (1.1 eq)	0 ºC	THF	2 h	40%	–	51:49
**5**	5 mol%	**L3** (11 mol%)	LHMDS (1.1 eq)	0 ºC	THF	2 h	40%	–	81:19
**6**	5 mol%	**L4** (11 mol%)	LHMDS (1.1 eq)	0 ºC	THF	2 h	40% (27%)	–	86:14
**7**	5 mol%	**L4** (11 mol%)	LHMDS (1.1 eq)	0 ºC	toluene	5 h	40%	–	85:15
**8**	5 mol%	**L4** (11 mol%)	LHMDS (1.1 eq)	0 ºC	DME/dioxan e 1/1	5 h	24%	–	95:5
**9**	5 mol%	**L4** (11 mol%)	LHMDS (1.1 eq)	rt	DME/dioxan e 1/1	5 h	52%	–	85:15
**10**	5 mol%	**L4** (11 mol%)	LHMDS (1.1 eq)	0 ºC	DME	5 h	42%	–	94:6
**11**	5 mol%	**L4** (11 mol%)	NaHMDS (1.1 eq)	0 ºC	DME	5 h	~10–15%	–.	92:8
**12**	5 mol%	**L4** (11 mol%)	LDA (1.1 eq)	0 ºC	DME	5 h	41%	–.	92:8
**13**	5 mol%	**L4** (11 mol%)	LHMDS (2.1 eq)	0 ºC	DME	4 h	full (48%)	–	93:7
**14**	5 mol%	**L4** (11 mol%)	LHMDS (1.6 eq)	0 ºC	DME	7 h	full (53%)	LiCl (3 eq.)	93:7

[a] Scale 0.2 mmol ketone; enolization at 0 ºC for 30 min.; $Pd_2(dba)_3CHCl_3$, ligand and acetate pre-stirred at rt for 30 min. [b] Conversion of acetate determined by ^1H NMR and corrected for ketone starting material. [c] Isolated yield. [d] Determined by ^{13}C NMR of the crude product (desired isomer 43.00 ppm, undesired isomer 43.41 ppm).

Ketone 29

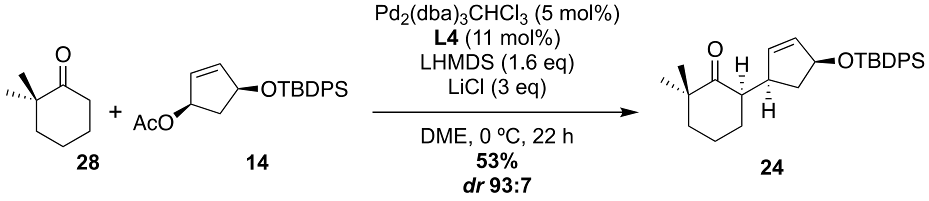

A Schlenk flask was flame-dried *in vacuo* and subjected to three cycles of evacuating and N_2 backfilling and charged with LiCl (25.6 mg, 0.60 mmol, 3 eq., stored and weighed out in a glovebox) under N_2 flow. The flask was again subjected to three cycles of evacuating and N_2 backfilling. Ketone **28** (93% purity by wt., 27.1 mg, 0.20 mmol, 1 eq.) was added dissolved in dry DME (1 mL). The resulting suspension was cooled to 0 ºC (cryostat). 1 M LHMDS in THF (0.32 mL, 0.32 mmol, 1.6 eq.) was added dropwise and the resulting mixture was stirred at 0 ºC for 30 min. Another flask was charged with acetate **14** (84.8 mg, 0.223 mmol, 1.1 eq.), $Pd_2(dba)_3{\cdot}CHCl_3$ (10.4 mg, 0.01 mmol, 0.05 eq.) and (*R*,*R*)-DACH-Phenyl-Trost Ligand **L4** (15.2 mg, 0.02 mmol, 0.11 eq.). The flask was placed under N_2 atmosphere (three cycles evacuating and N_2 backfilling), dry DME (0.8 mL) was added and the resulting mixture was stirred for 30 min at room temperature. Then the catalyst solution was added dropwise to the enolate at 0 ºC and rinsed with additional DME (0.2 mL). The reaction mixture was stirred for 22 h at 0 ºC and then quenched by addition of sat. aq. NH_4Cl (10 mL). The biphasic system was allowed to warm to room temperature and the aqueous phase was extracted with Et_2O (3 x 10 mL). The combined organic extracts were washed with brine (10 mL), dried over $MgSO_4$ and concentrated. The crude product was purified by flash chromatography (pentane/Et_2O 97/3) to give the product **29** (48.5 mg, 0.11 mmol, 53% yield, *dr* 93:7) as colorless oil.

The diastereomeric ratio was determined by ^{13}C NMR: major 51.99 ppm, minor 50.93 ppm, *dr* 93:7.

^1H NMR (400 MHz, Chloroform-*d*) δ 7.68 (td, *J* = 7.5, 1.6 Hz, 4H), 7.46 – 7.34 (m, 6H), 5.82 – 5.75 (m, 1H), 5.64 (dt, *J* = 5.7, 2.1 Hz, 1H), 4.82 – 4.76 (m, 1H), 2.91 – 2.82 (m, 1H), 2.64 (ddd, *J* = 13.0, 7.2, 5.4 Hz, 1H), 2.40 (dt, *J* = 13.7, 7.7 Hz, 1H), 2.26 (ddq, *J* = 12.7, 6.7, 3.6, 3.1 Hz, 1H), 1.94 – 1.67 (m, 3H), 1.55 (td, *J* = 13.2, 4.1 Hz, 1H), 1.42 – 1.34 (m, 1H), 1.33 – 1.24 (m, 1H), 1.20 (s, 3H), 1.06 (d, *J* = 4.8 Hz, 12H).

^{13}C NMR (101 MHz, Chloroform-*d*) δ 216.87, 135.88, 135.35, 134.62, 129.66, 129.65, 127.70, 127.66, 77.95, 51.99, 45.76, 43.03, 42.23, 39.92, 31.80, 27.08, 25.69, 25.12, 21.73, 19.23.

HRMS (ESI+) M+Na$^+$ calculated for $C_{29}H_{38}O_2Si$: 469.2533, found: 469.2545.

NOESY NMR analysis of **29**

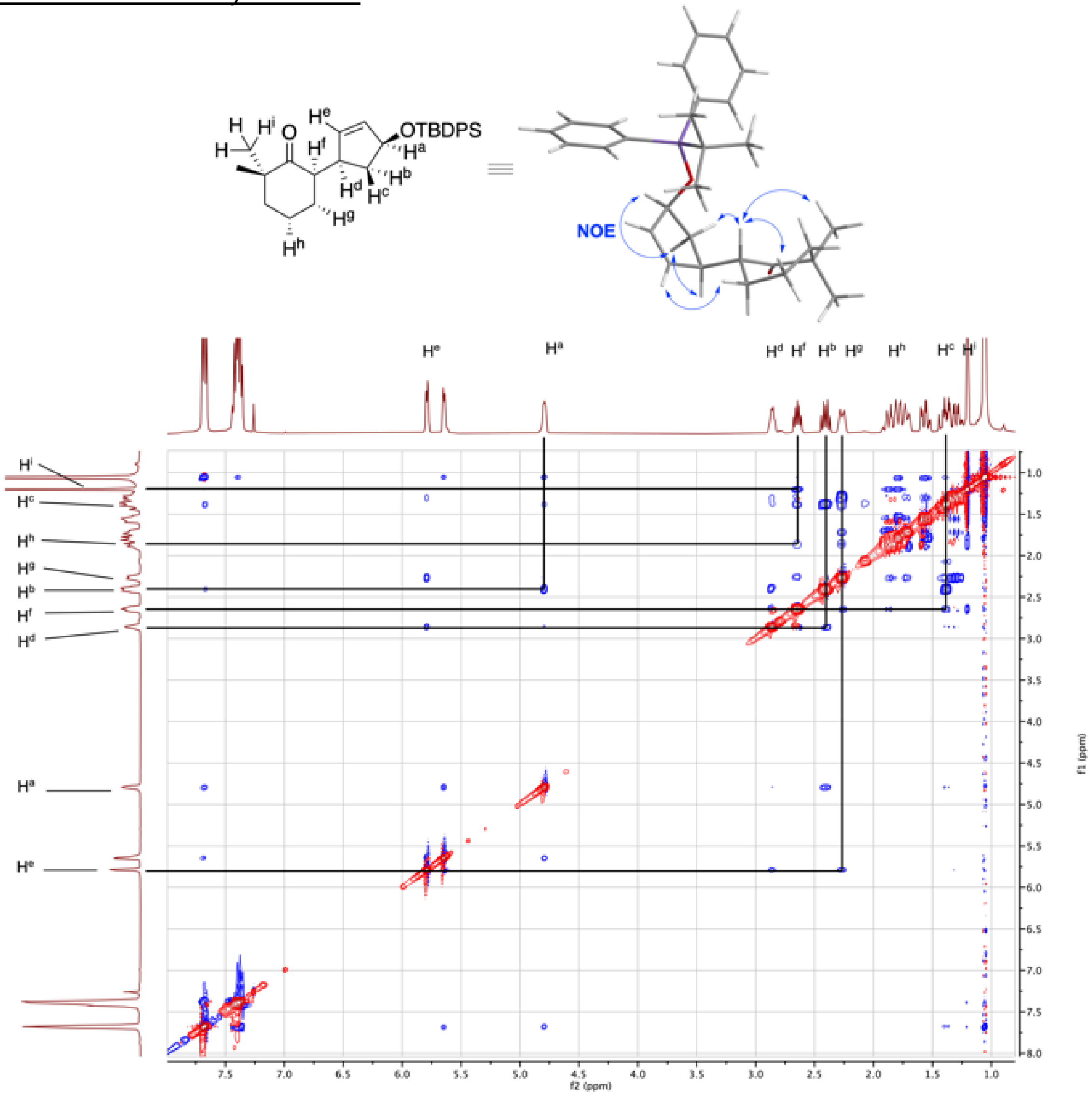

Ketone 30

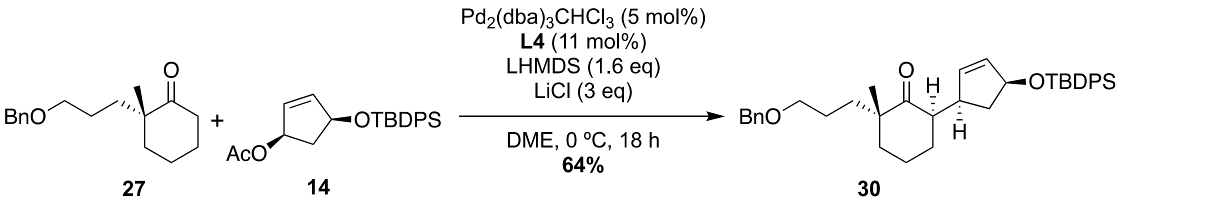

A Schlenk flask was flame-dried under vacuo and subjected to three cycles of evacuating and N_2 backfilling and charged with LiCl (886 mg, 20.9 mmol, 3 eq., weighed and stored in glovebox) under N_2 flow. The flask was again subjected to three cycles of evacuating and N_2 backfilling. Ketone **27** (1.81 g, 6.97 mmol, 1 eq.) was added dissolved in dry DME (50 mL). The resulting suspension was cooled to 0 ºC (cryostat). 1 M LHMDS in THF (11.0 mL, 11.0 mmol, 1.6 eq.) was added dropwise over two min and the resulting mixture was stirred at 0 ºC for 30 min. Another flask was charged with acetate **14** (2.67 g, 7.03 mmol, 1 eq.), $Pd_2(dba)_3 \cdot CHCl_3$ (361 mg, 0.35 mmol, 0.05 eq.) and (*R*,*R*)-DACH-Phenyl-Trost Ligand **L4** (529 mg, 0.77 mmol, 0.11 eq.). The flask was

placed under N_2 atmosphere (three cycles evacuating and N_2 backfilling), dry DME (18 mL) was added and the resulting mixture was stirred for 30 min at room temperature. Then the catalyst solution was added dropwise over 10 min to the enolate at 0 ºC. The reaction mixture was stirred for 18 h at 0 ºC and then quenched by addition of sat. aq. NH_4Cl (100 mL). The biphasic system was allowed to warm to room temperature and the aqueous phase was extracted with Et_2O (3 x 100 mL). The combined organic extracts were washed with brine (75 mL), dried over $MgSO_4$ and concentrated. The crude product was purified by flash chromatography (pentane/Et_2O 92/8) to give the product **30** (2.59 g, 4.46 mmol, 64% yield, single diastereoisomer) as colourless oil.

^{1}H NMR (400 MHz, Chloroform-*d*) δ 7.66 (t, *J* = 7.5 Hz, 4H), 7.46 – 7.27 (m, 11H), 5.80 – 5.73 (m, 1H), 5.62 (dd, *J* = 5.6, 2.6 Hz, 1H), 4.79 (t, *J* = 6.0 Hz, 1H), 4.46 (s, 2H), 3.41 (dt, *J* = 8.9, 4.4 Hz, 2H), 2.84 (q, *J* = 7.1 Hz, 1H), 2.55 (dt, *J* = 12.9, 6.2 Hz, 1H), 2.42 (dt, *J* = 14.8, 7.8 Hz, 1H), 2.25 (dt, *J* = 13.8, 4.1 Hz, 1H), 2.00 – 1.82 (m, 3H), 1.72 – 1.58 (m, 2H), 1.54 – 1.42 (m, 2H), 1.37 – 1.21 (m, 3H), 1.03 (d, *J* = 5.4 Hz, 12H).

^{13}C NMR (101 MHz, Chloroform-*d*) δ 216.70, 138.64, 135.89, 135.88, 135.08, 134.72, 134.69, 134.43, 129.67, 129.66, 128.50, 127.74, 127.71, 127.67, 78.06, 73.00, 70.58, 52.20, 49.04, 43.09, 41.16, 39.96, 34.22, 31.68, 27.10, 24.42, 22.46, 21.25, 19.24.

HRMS (ESI+) M+NH_4^+ calculated for $C_{38}H_{48}O_3Si$: 598.3711, found: 598.3710.

HRMS (ESI+) M+Na^+ calculated for $C_{38}H_{48}O_3Si$: 603.3265, found: 603.3261.

Optical Rotation: $[\alpha]_D^{23}$ = +69.1° (*c* = 0.44, $CHCl_3$).

NOESY NMR analysis of **30**

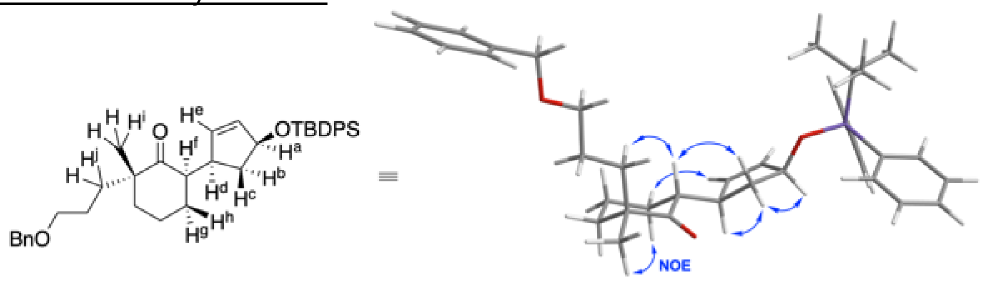

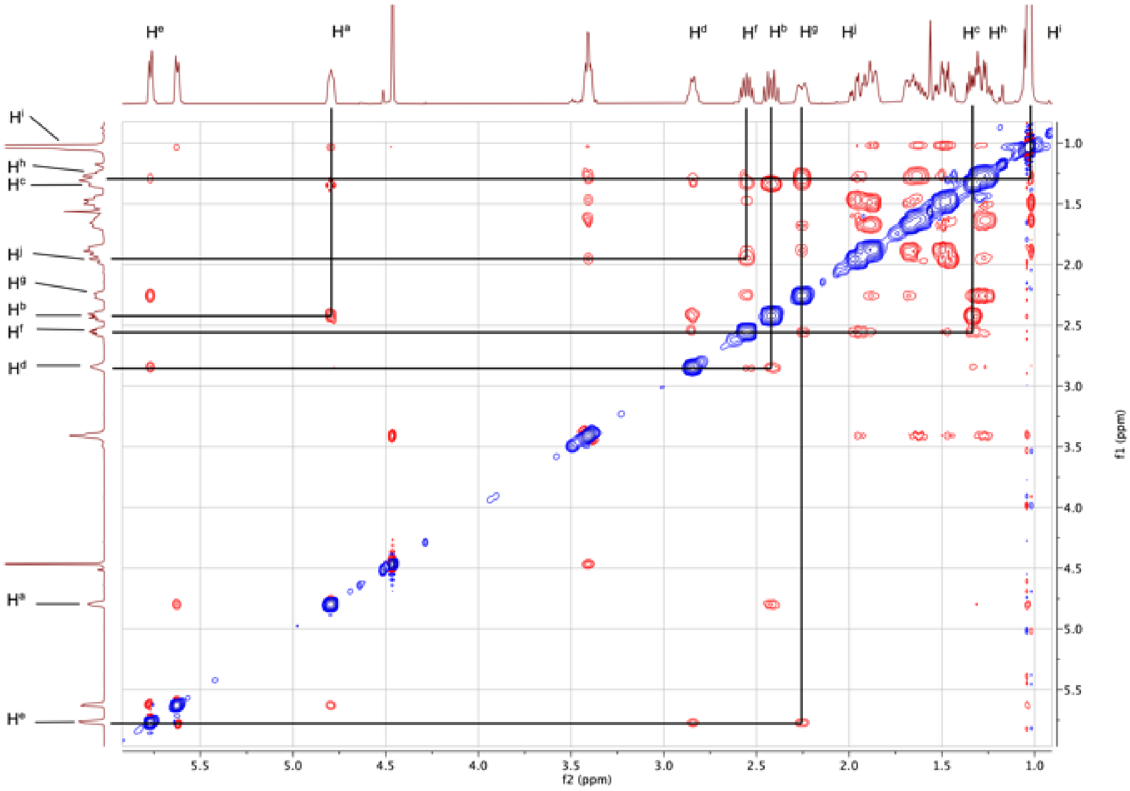

Preparation of Diacetate 11

Three-step synthesis of diacetate 11

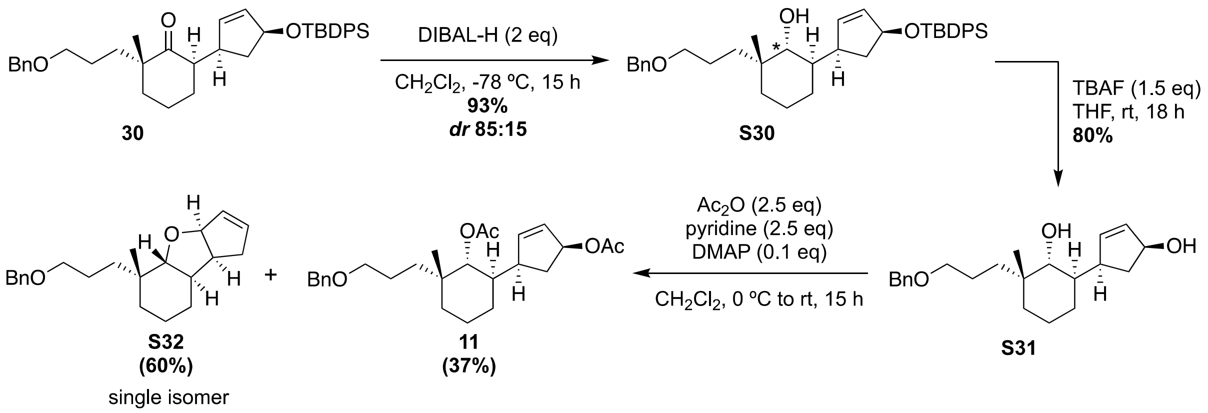

DIBAL-H reduction:

A Schlenk flask was flame-dried under vacuo and subjected to three cycles of evacuating and N_2 backfilling. The flask was charged with ketone **30** (661 mg, 1.14 mmol, 1.0 eq.) dissolved in dry CH_2Cl_2 (5.6 mL). The solution was cooled to -78 °C. 1 M DIBAL-H in CH_2Cl_2 (2.3 mL, 2.3 mmol, 2 eq.) was added. After stirring for 15 h at -78 °C, the reaction was quenched by addition of sat. Rochelle salt (20 mL) and allowed to come to room temperature. The reaction mixture was stirred for 2 h at room temperature. The layers were separated and the aqueous phase was extracted with CH_2Cl_2 (2 x 10 mL). The combined organic layers were dried over $MgSO_4$ and concentrated to give the crude alcohol **S30** (618 mg, 1.06 mmol, 93% yield) as a mixture of diastereomers (*dr* 85:15, see below). The crude was used in the next step without further purification.

TBAF deprotection:

A flask was charged with crude alcohol **S30** (600 mg, 1.03 mmol, 1 eq.) and dissolved in THF (5 mL). 1 M TBAF in THF (1.5 mL, 1.5 mmol, 1.5 eq) was added and the resulting solution was stirred at rt for 18 h. The reaction was quenched by addition of sat. aq. NH_4Cl (20 mL) and the aqueous phase was extracted with EtOAc (3 x 20 mL). The combined organic extracts were washed with brine (20 mL), dried over $MgSO_4$ and concentrated. The crude was purified by flash chromatography (pentane/Et_2O 3/2) to give diol **S31** (317 mg, 0.92 mmol, 89% yield) as colorless oil.

^{1}H NMR (400 MHz, Chloroform-*d*) δ 7.38 – 7.31 (m, 4H), 7.28 (td, *J* = 4.1, 1.9 Hz, 1H), 5.94 (ddq, *J* = 4.2, 3.1, 2.0, 1.5 Hz, 1H), 5.74 (dd, *J* = 5.5, 2.6 Hz, 1H), 4.75 (dtt, *J* = 6.6, 2.2, 1.1 Hz, 1H), 4.55 – 4.47 (m, 2H), 3.47 (tq, *J* = 5.5, 2.6 Hz, 2H), 3.22 (d, *J* = 10.6 Hz, 1H), 2.88 – 2.84 (m, 1H), 2.61 (s, br, 1H), 2.38 – 2.26 (m, 1H), 1.73 (ddt, *J* = 13.9, 10.4, 3.3 Hz, 1H), 1.68 – 1.58 (m, 2H), 1.54 (td, *J* = 7.0, 3.7 Hz, 4H), 1.40 – 1.32 (m, 2H), 1.29 – 1.21 (m, 1H), 1.01 (dd, *J* = 12.8, 8.4 Hz, 1H), 0.96 (s, 3H), 0.92 – 0.87 (m, 1H).

^{13}C NMR (101 MHz, Chloroform-*d*) δ 138.60, 137.97, 132.74, 128.44, 128.42, 127.76, 127.59, 80.98, 76.79, 72.99, 71.56, 46.82, 41.16, 38.18, 35.51, 35.32, 29.99, 26.03, 25.93, 23.71, 21.09.

HRMS (ESI+) M+Na$^+$ calculated for $C_{22}H_{32}O_3$: 367.2244, found: 367.2242.

Double acetylation:

A flask was charged with diol **S31** (282 mg, 0.82 mmol, 1 eq.) and DMAP (10 mg, 0.08 mmol, 0.1 eq.) and placed under N_2 atmosphere by three cycles of evacuating and N_2 backfilling. Dry CH_2Cl_2 (4 mL) and dry pyridine (0.2 mL, 2.49 mmol, 3 eq.) was added and the resulting solution was cooled to 0 ºC. Acetic anhydride (0.23 mL, 2.44 mmol, 3 eq.) was added. After stirring for 15 min at 0 ºC, the reaction mixture was stirred at rt for 15 h. The reaction mixture was diluted with CH_2Cl_2 (10 mL) and then washed with 1 M aq. HCl (2 x 12 mL) and sat. aq. $NaHCO_3$ (15 mL). The organic layer was dried over $MgSO_4$ and concentrated. The crude was purified by flash chromatography (pentane/Et2O 4/1) to give diacetate **11** (131 mg, 0.31 mmol, 37% yield) and cyclization product **S32** (160 mg, 0.49 mmol, 60% yield).

Analytical data cyclization product **S32**

^{1}H NMR (400 MHz, Chloroform-*d*) δ 7.38 – 7.26 (m, 5H), 5.98 (dt, *J* = 5.6, 2.3 Hz, 1H), 5.70 (dq, *J* = 5.7, 2.3 Hz, 1H), 5.04 (dt, *J* = 7.0, 1.7 Hz, 1H), 4.51 (s, 2H), 3.47 (qdd, *J*

= 9.2, 7.3, 6.2 Hz, 2H), 2.86 – 2.74 (m, 2H), 2.34 – 2.19 (m, 2H), 1.87 (ddt, *J* = 11.9, 7.4, 3.7 Hz, 1H), 1.82 – 1.76 (m, 1H), 1.67 – 1.56 (m, 3H), 1.55 – 1.47 (m, 2H), 1.47 – 1.38 (m, 1H), 1.37 – 1.28 (m, 1H), 1.08 (qd, *J* = 12.4, 4.1 Hz, 1H), 0.98 (s, 3H), 0.88 (tdd, *J* = 13.7, 4.1, 1.0 Hz, 1H).

^{13}C NMR (101 MHz, Chloroform-*d*) δ 138.85, 136.42, 129.97, 128.46, 127.78, 127.57, 87.30, 86.16, 72.98, 71.76, 41.67, 41.20, 36.46, 35.55, 32.20, 27.23, 26.79, 26.31, 24.20, 22.13.

<u>LCMS spectrum of **S32**</u>

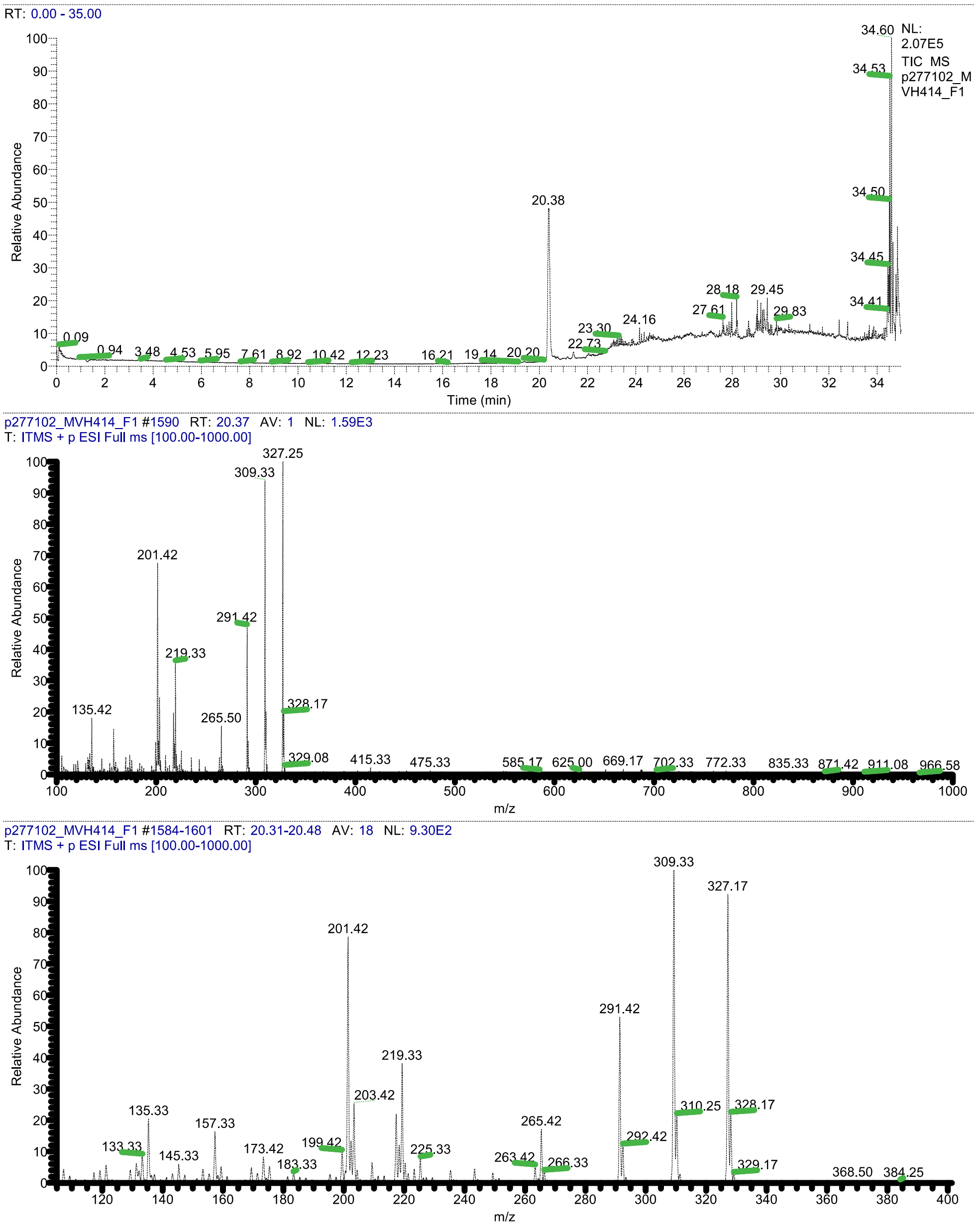

NOESY NMR analysis of **S32**

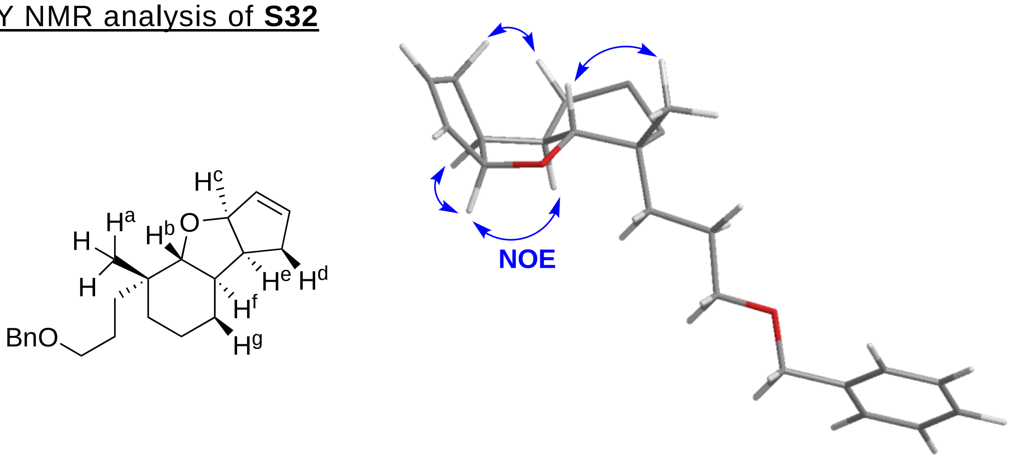

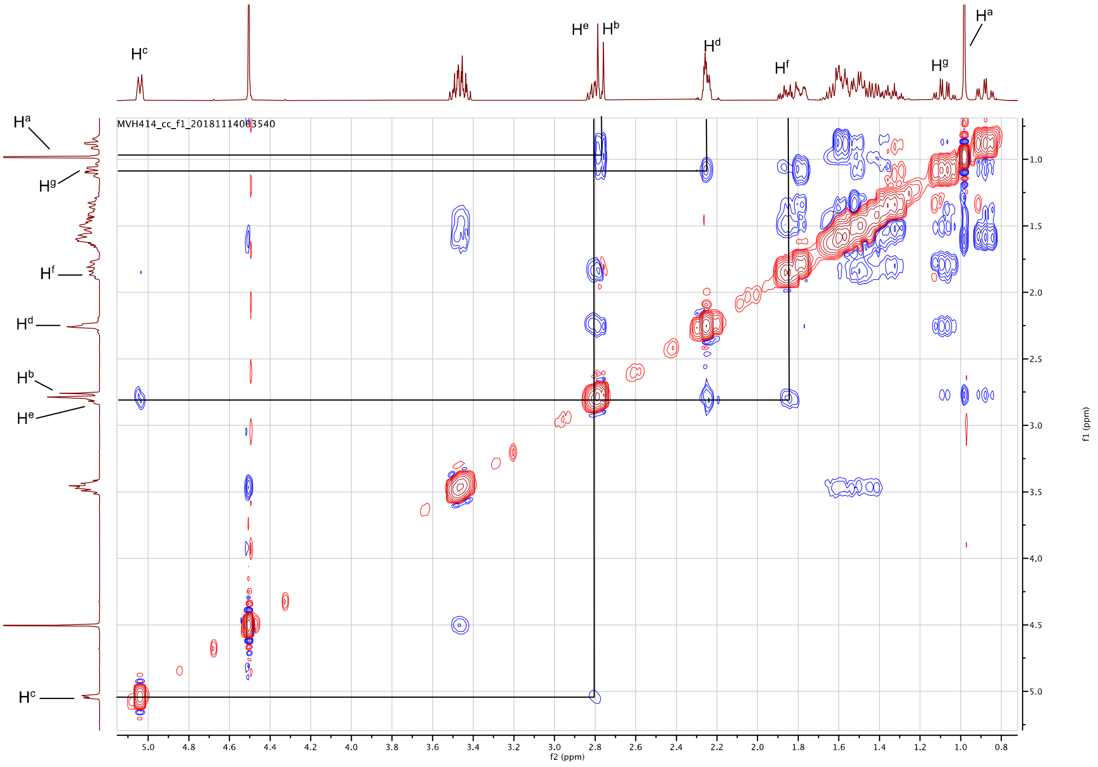

Acetate 31

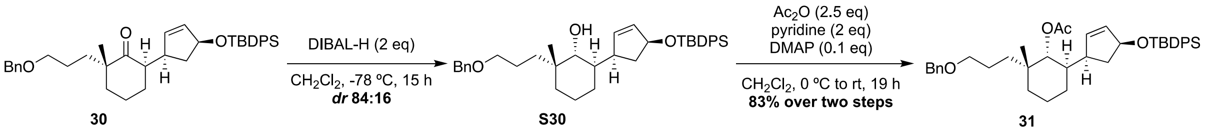

A Schlenk flask was flame-dried under vacuo and subjected to three cycles of evacuating and N_2 backfilling. The flask was charged with ketone **30** (2.56 g, 4.41 mmol, 1.0 eq.) dissolved in dry CH_2Cl_2 (22 mL). The solution was cooled to -78 °C. 1 M DIBAL-H in CH_2Cl_2 (8.8 mL, 8.8 mmol, 2.0 eq.) was added. After stirring for 1 h at -78 °C, the reaction was quenched by addition of sat. Rochelle salt (50 mL) and allowed to come to room temperature. The reaction mixture was stirred for 23 h at room temperature. The layers were separated and the aqueous phase was extracted with Et_2O (3 x 40 mL). The combined organic layers were washed with brine (50 mL), dried

over $MgSO_4$ and concentrated to give the crude alcohol **S30** as a mixture of diastereomers. The crude was used in the next step without further purification.

The diastereomeric ratio was determined by ^{1}H NMR integration: major 5.99 ppm (dd, *J* = 5.7, 2.4 Hz) : minor 5.86 (dd, J = 5.7, 1.8 Hz) = 84:16

HRMS (ESI+) M+Na$^+$ calculated for $C_{38}H_{50}O_3Si$: 605.3421, found: 605.3416.

A flask was charged with crude alcohol **S30** and DMAP (54.0 mg, 0.44 mmol, 0.1 eq.) and placed under N_2 atmosphere by three cycles of evacuating and N_2 backfilling. Dry CH_2Cl_2 (44 mL) and dry pyridine (0.71 mL, 8.82 mmol, 2.0 eq.) was added and the resulting solution was cooled to 0 °C. Ac_2O (1.0 mL, 11.0 mmol, 2.5 eq.) was added and after stirring for 5 min at 0 °C the reaction mixture was stirred at room temperature for 19 h. The reaction was quenched by addition of 50 mL sat. aq. NH_4Cl and the layers were separated. The aqueous phase was extracted with Et_2O (2 x 50 mL) and the combined organic layers were washed with brine (50 mL), dried over $MgSO_4$ and concentrated. The crude product was purified by flash chromatography (pentane/Et_2O 9/1) to give the product **31** (2.30 g, 3.68 mmol, 83% yield) as single diastereoisomer.

^{1}H NMR (400 MHz, Chloroform-*d*) δ 7.68 (dt, *J* = 8.0, 1.9 Hz, 4H), 7.45 – 7.33 (m, 10H), 7.30 (h, *J* = 4.7, 4.3 Hz, 1H), 5.81 (dt, *J* = 5.7, 1.5 Hz, 1H), 5.62 (dt, *J* = 5.7, 2.1 Hz, 1H), 4.77 (ddt, *J* = 6.2, 4.7, 1.5 Hz, 1H), 4.72 (d, *J* = 11.3 Hz, 1H), 4.53 (s, 2H), 3.48 (t, *J* = 6.4 Hz, 2H), 2.50 – 2.39 (m, 1H), 2.12 (dt, *J* = 13.3, 8.0 Hz, 1H), 2.02 (s, 3H), 1.98 – 1.90 (m, 1H), 1.75 (tt, *J* = 12.0, 3.9 Hz, 1H), 1.70 – 1.63 (m, 1H), 1.62 – 1.48 (m, 5H), 1.43 – 1.36 (m, 1H), 1.36 – 1.27 (m, 1H), 1.12 (dd, *J* = 13.7, 3.9 Hz, 1H), 1.06 (s, 9H), 1.02 – 0.92 (m, 1H), 0.85 (s, 3H).

^{13}C NMR (101 MHz, Chloroform-*d*) δ 170.97, 138.77, 135.93, 135.91, 134.69, 134.65, 134.42, 134.34, 129.63, 128.51, 127.81, 127.67, 127.65, 82.48, 78.07, 73.08, 71.53, 44.34, 40.13, 38.36, 38.15, 35.56, 27.26, 27.07, 26.99, 25.69, 23.72, 21.21, 20.82, 19.25.

HRMS (ESI+) M+Na$^+$ calculated for $C_{40}H_{52}O_4Si$: 647.3527, found: 647.3535.

Optical Rotation: $[\alpha]_D^{23}$ = +45.0° (*c* = 0.23, $CHCl_3$).

Diacetate 11

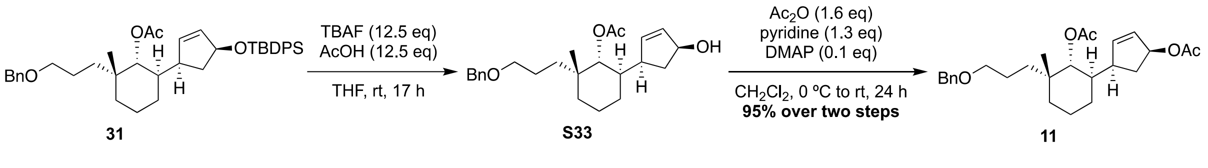

A flask was charged with acetate **31** (2.05 g, 3.28 mmol, 1 eq.) and dissolved in THF (21 mL). To the stirred solution was added glacial AcOH (2.35 mL, 41.0 mmol, 12.5 eq.) and 1 M TBAF in THF (41.0 mL, 41.0 mmol, 12.5 eq.). The reaction mixture was stirred for 17 h at room temperature. The reaction was quenched by addition of sat. aq. NH_4Cl (75 mL) and the layers were separated. The aqueous phase was extracted with Et_2O (2 x 75 mL) and the combined organic layers were washed with sat. aq. $NaHCO_3$ (3 x 30 mL) and brine (30 mL), dried over $MgSO_4$ and concentrated to give the crude product **S33** which was used in the next step without further purification.

HRMS (ESI+) M+Na$^+$ calculated for $C_{24}H_{34}O_4$: 409.2349, found: 409.2345.

A flask was charged with crude alcohol **S33** and DMAP (40.0 mg, 0.33 mmol, 0.1 eq.) and placed under N_2 atmosphere by three cycles of evacuating and N_2 backfilling. Dry CH_2Cl_2 (33 mL) and dry pyridine (0.34 mL, 4.23 mmol, 1.3 eq.) was added and the resulting solution was cooled to 0 °C. Ac_2O (0.5 mL, 5.30 mmol, 1.6 eq.) was added and after stirring for 15 min at 0 °C the reaction mixture was stirred at room temperature for 24 h. The reaction was quenched by addition of sat. aq. NH_4Cl (75 mL) and the layers were separated. The aqueous phase was extracted with Et_2O (3 x 50 mL) and the combined organic layers were washed with brine (50 mL), dried over $MgSO_4$ and concentrated. The crude was purified by flash chromatography (pentane/Et_2O 7/2) to give the product **11** (1.34 g, 3.12 mmol, 95% yield) as colourless oil.

^{1}H NMR (400 MHz, Chloroform-*d*) δ 7.40 – 7.24 (m, 5H), 6.05 – 5.99 (m, 1H), 5.78 – 5.71 (m, 1H), 5.61 – 5.55 (m, 1H), 4.72 (d, *J* = 11.4 Hz, 1H), 4.51 (s, 2H), 3.51 – 3.43 (m, 2H), 2.68 – 2.60 (m, 1H), 2.48 – 2.39 (m, 1H), 2.06 (s, 3H), 2.03 (s, 3H), 1.82 – 1.72 (m, 2H), 1.66 (d, *J* = 13.9 Hz, 1H), 1.61 – 1.38 (m, 6H), 1.35 – 1.25 (m, 1H), 1.12 – 1.03 (m, 1H), 0.93 (qd, *J* = 13.5, 4.5 Hz, 1H), 0.84 (s, 3H).

^{13}C NMR (101 MHz, Chloroform-*d*) δ 171.00, 170.92, 138.69, 138.17, 129.68, 128.44, 127.72, 127.61, 82.10, 79.62, 73.01, 71.40, 44.69, 39.97, 38.09, 35.40, 34.38, 27.13, 26.94, 25.59, 23.64, 21.41, 21.17, 20.69.

HRMS (ESI+) $M+Na^+$ calculated for $C_{26}H_{36}O_5$: 451.2455, found: 451.2460.

Optical Rotation: $[\alpha]_D^{23}$ = +47.6° (*c* = 0.44, $CHCl_3$).

NOESY NMR analysis of **11**

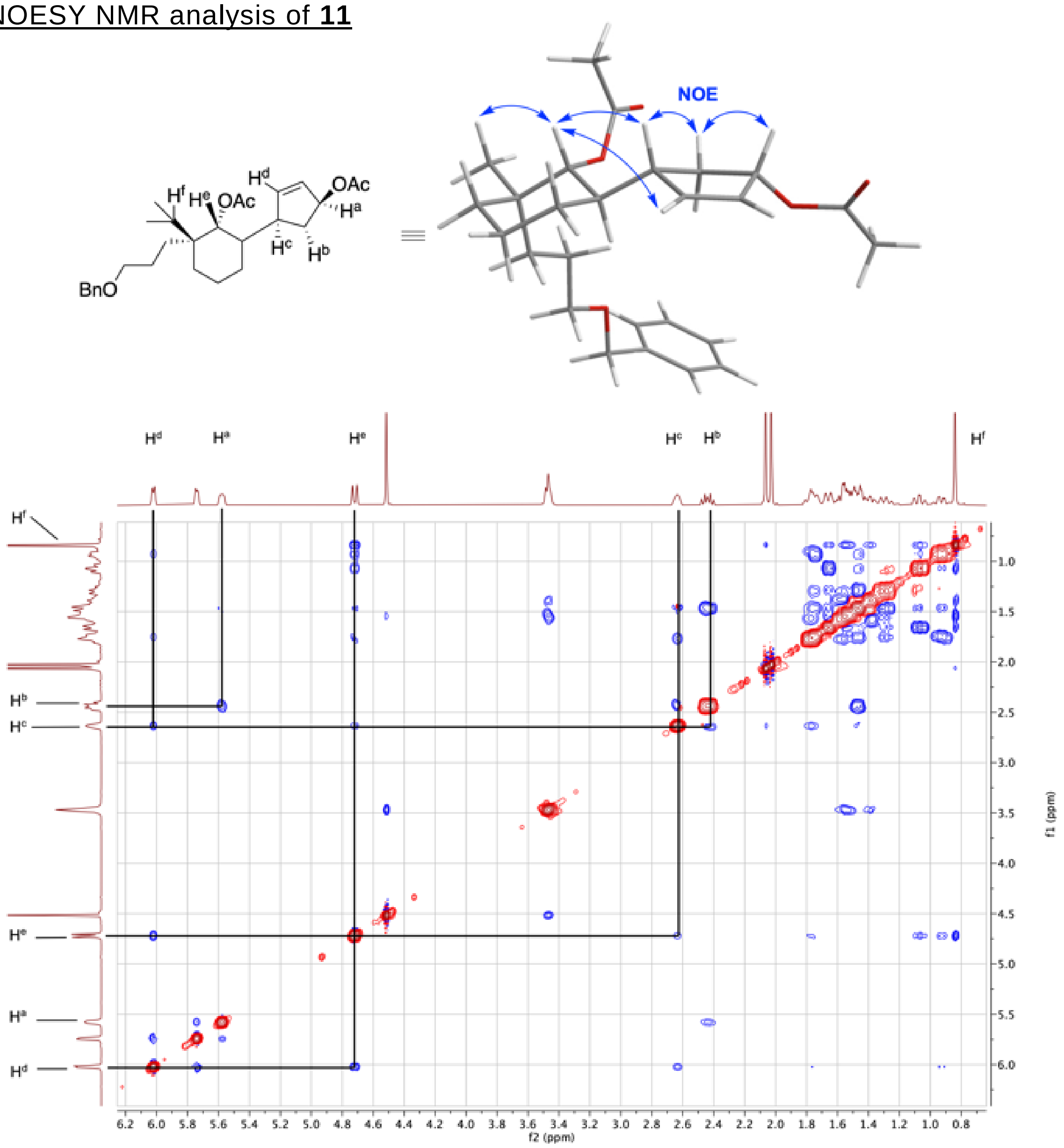

Preparation of Grignard Reagent 32

MOM ether S34

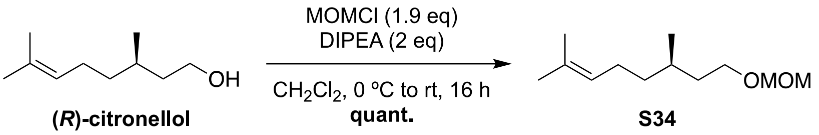

A flask was placed under N_2 atmosphere by three cycles of evacuating and N_2 backfilling and charged with (*R*)-citronellol (5.50 g, 35.2 mmol, 1.0 eq.). Dry CH_2Cl_2 (59 mL) and DIPEA (12.3 mL, 70.6 mmol, 2.0 eq.) were added and the solution was cooled to 0 °C. MOMCl (5.2 mL, 68.5 mmol, 1.9 eq.) was added dropwise over 2 min. After stirring for 15 min, the ice bath was removed and the reaction mixture was stirred for 16 h at room temperature. The solution was diluted with CH_2Cl_2 (100 mL), washed with

sat. aq. $NaHCO_3$ (50 mL), dried over $MgSO_4$ and concentrated. The crude product **S34** (7.03 g, 35.1 mmol, quant. yield) was used in the next step without further purification.

^{1}H NMR (400 MHz, Chloroform-*d*) δ 5.09 (t, *J* = 6.6 Hz, 1H), 4.60 (s, 2H), 3.60 – 3.49 (m, 2H), 3.35 (s, 3H), 2.06 – 1.88 (m, 2H), 1.67 (s, 3H), 1.66 – 1.50 (m, 5H), 1.44 – 1.29 (m, 2H), 1.21 – 1.11 (m, 1H), 0.89 (d, *J* = 6.0 Hz, 3H).

^{13}C NMR (101 MHz, Chloroform-*d*) δ 131.26, 124.90, 96.54, 66.18, 55.20, 37.30, 36.84, 29.66, 25.82, 25.60, 19.58, 17.72.

Optical Rotation: $[\alpha]_D^{23}$ = +2.6° (*c* = 0.72, $CHCl_3$).

Alcohol S35

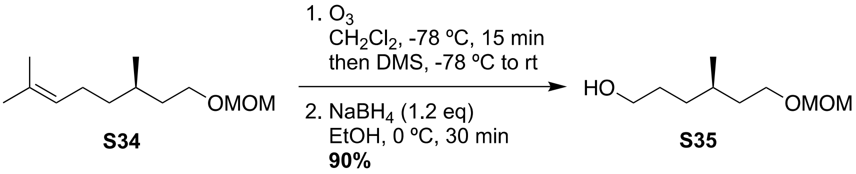

A 3-neck flask was charged with **S34** (6.05 g, 30.2 mmol, 1.0 eq.) and dissolved in dry CH_2Cl_2 (150 mL). N_2 was bubbled through the solution for 15 min and then cooled to -78 °C. Ozone was then bubbled through the solution for 15 min until a faint blue colour appeared and TLC indicated full conversion of the starting material. N_2 was bubbled through the solution for 10 min followed by addition of dimethyl sulfide (4.4 mL, 60.4 mmol, 2.0 eq.). The cooling bath was removed and the reaction mixture was allowed to come to room temperature. The organic layer was washed with water (50 mL), dried over $MgSO_4$ and concentrated. The crude aldehyde was then dissolved in abs. ethanol (150 mL) and cooled to 0 °C. $NaBH_4$ (1.38 g, 36.6 mmol, 1.2 eq.) was added in portions and the reaction mixture was stirred for 30 min. The reaction was quenched by addition of sat. aq. NH_4Cl (150 mL) and the aqueous phase was extracted with Et_2O (3 x 100 mL). The combined organic extracts were washed with sat. aq. $NaHCO_3$ (2 x 50 mL) and brine (50 mL), dried over $MgSO_4$ and concentrated to give the product **S35** (4.80 g, 27.2 mmol, 90% yield over two steps) as colourless liquid.

^{1}H NMR (400 MHz, Chloroform-*d*) δ 4.60 (s, 2H), 3.62 (t, *J* = 6.6 Hz, 1H), 3.58 – 3.51 (m, 1H), 3.35 (s, 3H), 1.68 – 1.49 (m, 5H), 1.40 (dddd, *J* = 10.7, 8.7, 7.7, 5.7 Hz, 2H), 1.24 – 1.14 (m, 1H), 0.91 (d, *J* = 6.6 Hz, 3H).

^{13}C NMR (101 MHz, Chloroform-*d*) δ 96.52, 66.11, 63.42, 63.23, 55.25, 36.80, 36.78, 33.10, 30.27, 29.78, 19.60.

HRMS (ESI+) M+Na$^+$ calculated for $C_9H_{20}O_3$: 199.1305, found: 199.1301.

Bromide S36

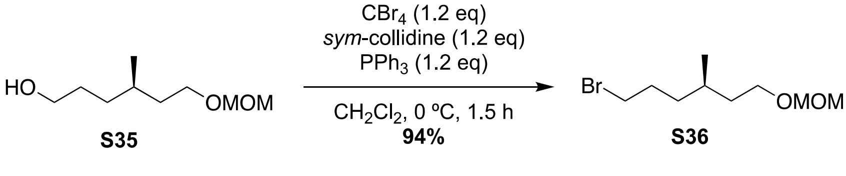

A flask was placed under N_2 atmosphere by three cycles of evacuating and N_2 backfilling and charged with **S35** (1.97 g, 11.2 mmol, 1.0 eq.) dissolved in dry CH_2Cl_2 (45 mL). CBr_4 (4.45 g, 13.4 mmol, 1.2 eq.) and *sym*-collidine (1.8 mL, 13.6 mmol, 1.2

eq.) were added and the solution was cooled to 0 °C. PPh_3 (3.51 g, 13.4 mmol, 1.2 eq.) was added in portions under N_2 flow. The reaction mixture was stirred for 1.5 h at 0 °C and then poured onto pentane (150 mL). The solids were filtered off and the filtrate was concentrated. The crude was purified by flash chromatography (pentane/Et_2O 9/1) to give the product **S36** (2.51 g, 9.68 mmol, 94% yield) as colourless liquid.

^{1}H NMR (400 MHz, Chloroform-*d*) δ 4.60 (s, 2H), 3.55 (tq, *J* = 6.4, 3.1, 2.6 Hz, 2H), 3.38 (t, *J* = 6.9 Hz, 2H), 3.35 (s, 3H), 1.96 – 1.77 (m, 2H), 1.62 (dp, *J* = 17.6, 5.8 Hz, 2H), 1.51 – 1.36 (m, 2H), 1.32 – 1.22 (m, 1H), 0.91 (d, *J* = 6.5 Hz, 3H).

^{13}C NMR (101 MHz, Chloroform-*d*) δ 96.58, 65.93, 55.28, 36.71, 35.60, 34.25, 30.49, 29.41, 19.55.

Optical Rotation: $[\alpha]_D^{23}$ = +3.9° (*c* = 0.67, $CHCl_3$).

Grignard reagent 32

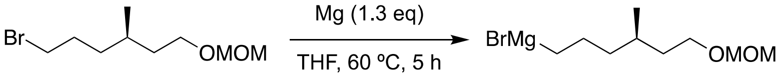

S36 **32**

A two-neck flask was charged with freshly ground magnesium turnings (233 mg, 9.59 mmol, 1.3 eq.) and then flame-dried under vacuo and subjected to three cycles of evacuating and N_2 backfilling. Dry THF (10 mL) was added and the magnesium was activated by addition of a few drops of dibromoethane and soft heating with a heat gun. When the solvent turned slightly cloudy, a solution of bromide **S36** (1.77 g, 7.38 mmol, 1 eq.) in dry THF (5 mL) was added dropwise over 15 min. The addition was kept at a rate where the reaction mixture remained mildly exothermic. After complete addition, the reaction mixture was heated to 60 ºC and stirred for 5 h at this temperature. The concentration of **32** was determined to be 0.14 M by indirect titration with a 0.5 M solution of menthol in THF using 1,10-phenanthroline as indicator.

Preparation of Benzyl Ether 36

Alkene 33

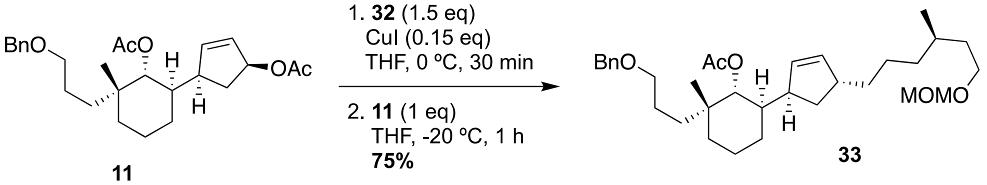

11 **33**

A Schlenk flask was flame-dried under vacuo and subjected to three cycles of evacuating and N_2 backfilling. The flask was charged with CuI (34.3 mg, 0.18 mmol, 0.15 eq.) under N_2 flow and again subjected to three cycles of evacuating and N_2 backfilling. Dry THF (2.0 mL) was added and the suspension was cooled to 0 °C. A solution of freshly prepared Grignard **32** in THF (c = 0.14 M, 12.6 mL, 1.76 mmol, 1.5 eq.) was added dropwise and the resulting deep purple solution was stirred for 30 min at 0 °C and the cooled to -20 °C. Then diacetate **11** (504 mg, 1.76 mmol, 1.0 eq.) dissolved in dry THF (7 mL) was added dropwise. The flask was rinsed with another portion of dry THF (2 mL) and the reaction mixture was stirred for 1 h at -20 °C and then quenched by addition of sat. aq. NH_4Cl (40 mL). After warming to room temperature, the aqueous phase was extracted with Et_2O (3 x 40 mL) and the combined organic extracts were washed with brine (50 mL), dried over $MgSO_4$ and concentrated

to give the crude product (crude *dr* ~ 4/1 by NMR) which was purified by two rounds of flash chromatography (toluene/Et_2O 95/5 to separate diastereoisomers, then 95/5 pentane/Et_2O to remove Grignard impurities) to give the product **33** (464 mg, 0.88 mmol, 75% yield) as colourless oil as single diastereoisomer.

^{1}H NMR (400 MHz, Chloroform-*d*) δ 7.38 – 7.26 (m, 5H), 5.68 – 5.64 (m, 1H), 5.64 – 5.60 (m, 1H), 4.66 (d, *J* = 11.3 Hz, 1H), 4.61 (s, 2H), 4.52 (s, 2H), 3.54 (ddt, *J* = 9.5, 6.4, 2.5 Hz, 2H), 3.47 (t, *J* = 5.9 Hz, 2H), 3.35 (s, 3H), 2.78 – 2.71 (m, 1H), 2.65 – 2.55 (m, 1H), 2.05 (s, 3H), 1.85 – 1.73 (m, 2H), 1.70 – 1.59 (m, 4H), 1.58 – 1.48 (m, 5H), 1.45 – 1.37 (m, 3H), 1.33 – 1.26 (m, 4H), 1.21 – 1.16 (m, 1H), 1.16 – 1.11 (m, 1H), 1.02 (tt, *J* = 13.1, 9.4 Hz, 2H), 0.88 (d, *J* = 6.6 Hz, 3H), 0.83 (s, 3H).

^{13}C NMR (101 MHz, Chloroform-*d*) δ 170.90, 138.75, 136.06, 131.29, 128.47, 127.76, 127.62, 96.54, 82.06, 73.03, 71.52, 66.20, 55.23, 45.53, 45.47, 41.06, 38.04, 37.38, 37.21, 36.90, 36.88, 36.86, 36.84, 35.43, 34.61, 30.07, 29.95, 29.93, 27.06, 26.98, 26.60, 25.66, 25.39, 23.68, 21.24, 20.72, 19.67.

HRMS (ESI+) M+Na^+ calculated for $C_{33}H_{52}O_5$: 551.3707, found: 551.3692.

Optical Rotation: $[\alpha]_D^{23}$ = +134.7° (*c* = 0.58, $CHCl_3$).

<u>NOESY NMR analysis **33**</u>

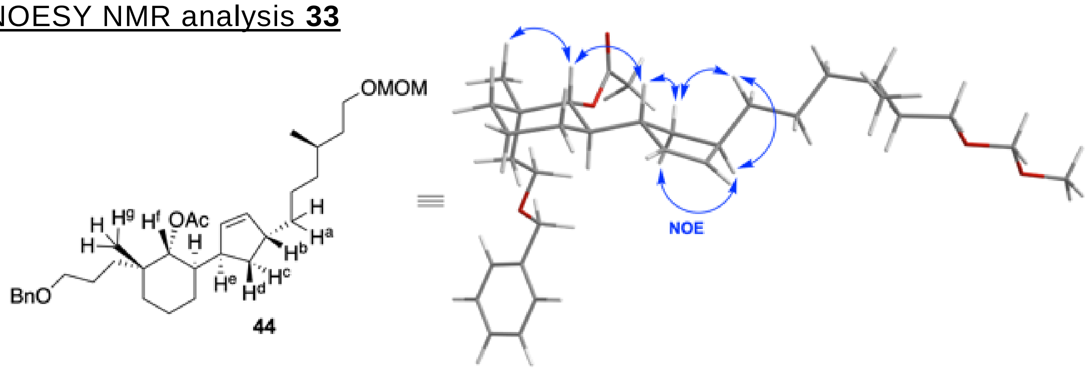

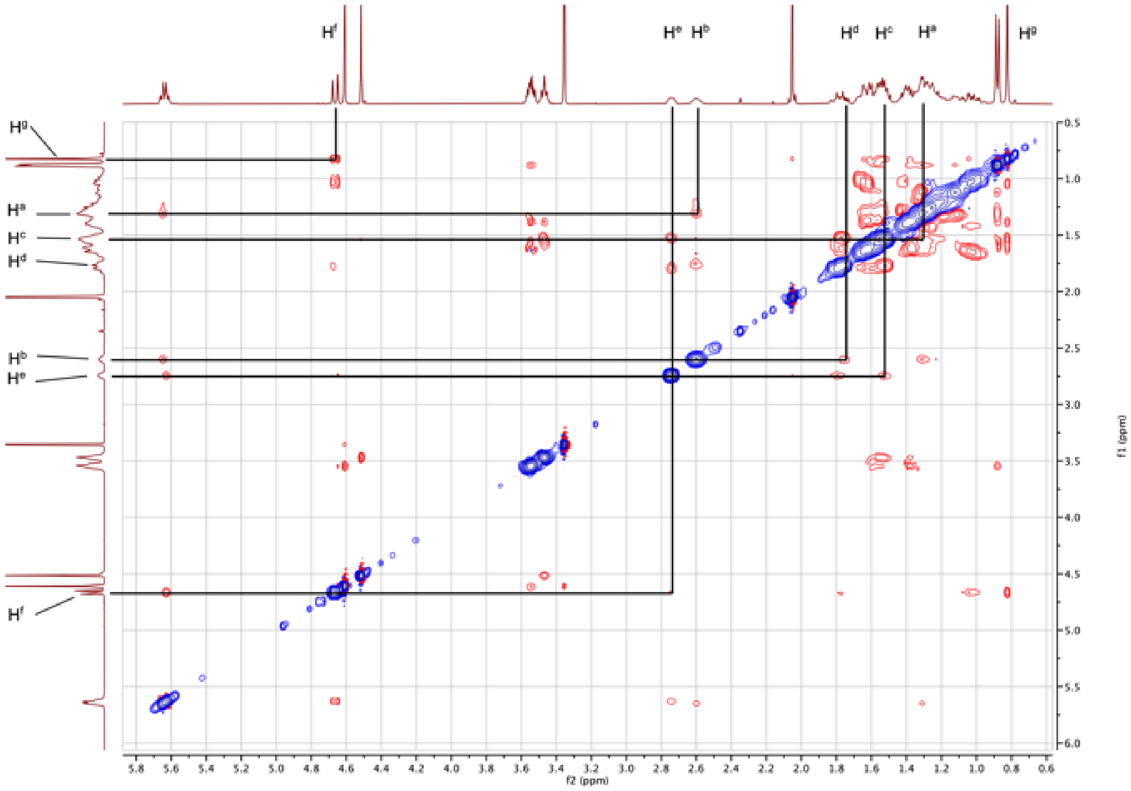

MOM ether S37

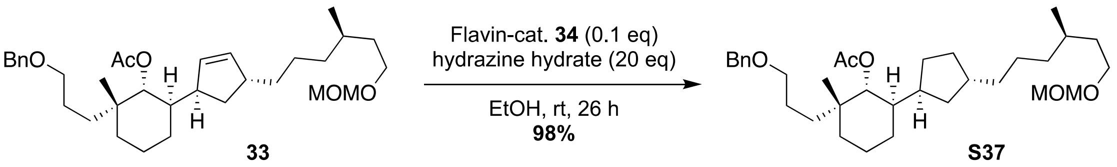

A flask was charged with alkene **33** (441 mg, 0.83 mmol, 1.0 eq.) and dissolved in abs. ethanol (5.6 mL). Flavine catalyst **34** (33.9 mg, 0.08 mmol, 0.1 eq.) was added followed by addition of hydrazine hydrate (0.81 mL, 16.7 mmol, 20.0 eq.). The reaction mixture was stirred at room temperature for 26 h, then taken up in Et_2O (50 mL) and washed with water (10 mL) and brine (2 x 10 mL), dried over $MgSO_4$ and concentrated to give the product **S37** (433 mg, 0.82 mmol, 98% yield) as pale yellow oil.

^{1}H NMR (400 MHz, Chloroform-*d*) δ 7.38 – 7.31 (m, 4H), 7.31 – 7.26 (m, 1H), 4.63 – 4.58 (m, 3H), 4.52 (s, 2H), 3.54 (ddt, *J* = 9.7, 6.4, 3.0 Hz, 2H), 3.47 (td, *J* = 6.6, 1.7 Hz, 2H), 3.36 (s, 3H), 2.06 (s, 3H), 1.86 (dddd, *J* = 17.3, 12.4, 8.6, 5.3 Hz, 1H), 1.77 – 1.68

(m, 4H), 1.66 – 1.57 (m, 4H), 1.56 – 1.45 (m, 5H), 1.42 – 1.33 (m, 2H), 1.30 – 1.18 (m, 8H), 1.12 – 1.02 (m, 3H), 0.98 – 0.92 (m, 1H), 0.88 (d, *J* = 6.5 Hz, 3H), 0.82 (s, 3H).

^{13}C NMR (101 MHz, Chloroform-*d*) δ 171.00, 138.78, 128.49, 127.79, 127.65, 96.56, 83.43, 73.05, 71.56, 66.24, 55.25, 40.60, 39.69, 39.61, 38.17, 37.44, 37.04, 36.92, 35.49, 33.46, 29.97, 28.51, 27.28, 27.04, 26.08, 25.69, 23.69, 21.42, 20.88, 19.67.

HRMS (ESI+) M+Na$^+$ calculated for $C_{33}H_{54}O_5$: 553.3864, found: 553.3862.

Optical Rotation: $[\alpha]_D^{23}$ = +19.1° (*c* = 0.53, $CHCl_3$).

Alcohol 35

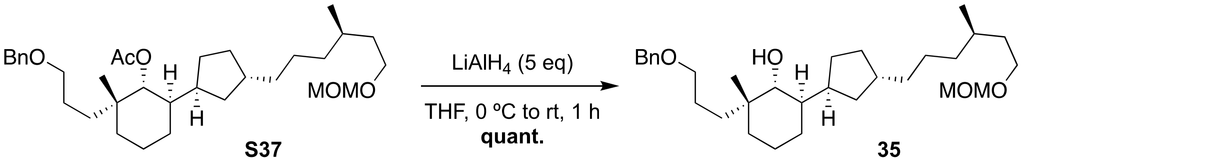

A flask was charged with acetate **S37** (424 mg 0.80 mmol, 1.0 eq.) and placed under N_2 atmosphere by three cycles of evacuating and N_2 backfilling. Dry THF (5.2 mL) was added and the resulting solution was cooled to 0 °C. 1 M $LiAlH_4$ in THF (4.0 mL, 4.0 mmol, 5.0 eq.) was added and after stirring for 5 min at 0 °C the reaction mixture was stirred at room temperature for one h. The reaction was cooled again to 0 °C and quenched by careful addition of sat. aq. Rochelle salt (15 mL) and then diluted with Et_2O (15 mL). The biphasic mixture was stirred for 14 h at room temperature. The layers were separated and the aqueous phase was extracted with Et_2O (2 x 15 mL). The combined organic layers were washed with brine (15 mL), dried over $MgSO_4$ and concentrated to give the product **35** (392 mg, 0.80 mmol, quant. yield) as colourless oil.

^{1}H NMR (400 MHz, Chloroform-*d*) δ 7.34 (d, *J* = 4.4 Hz, 4H), 7.31 – 7.26 (m, 1H), 4.62 (s, 2H), 4.51 (s, 2H), 3.55 (tt, *J* = 7.0, 3.4 Hz, 2H), 3.49 – 3.44 (m, 2H), 3.36 (s, 3H), 3.04 (d, *J* = 10.5 Hz, 1H), 2.29 – 2.18 (m, 1H), 1.77 (p, *J* = 7.0 Hz, 2H), 1.70 – 1.58 (m, 5H), 1.56 – 1.47 (m, 5H), 1.45 – 1.23 (m, 12H), 1.16 – 1.08 (m, 1H), 1.04 – 0.98 (m, 1H), 0.96 (s, 3H), 0.93 (t, *J* = 4.8 Hz, 1H), 0.88 (d, *J* = 6.6 Hz, 3H).

^{13}C NMR (101 MHz, cdcl$_3$) δ 138.78, 128.49, 127.82, 127.63, 96.56, 82.58, 73.10, 71.71, 66.27, 55.27, 41.80, 39.55, 39.25, 38.19, 37.45, 37.16, 36.92, 36.76, 35.49, 33.38, 29.98, 27.39, 26.48, 26.20, 26.06, 23.83, 21.14, 19.72.

HRMS (ESI+) M+Na$^+$ calculated for $C_{31}H_{52}O_4$: 511.3758, found: 511.3744.

Optical Rotation: $[\alpha]_D^{23}$ = +13.6° (*c* = 0.31, $CHCl_3$).

Benzyl ether 36

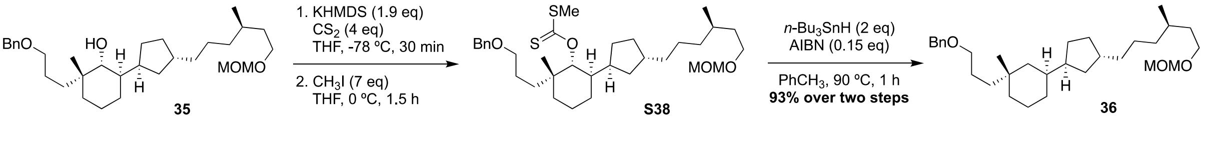

A Schlenk flask was flame-dried under vacuo and subjected to three cycles of evacuating and N_2 backfilling. The flask was charged with alcohol **35** (305 mg, 0.62 mmol, 1.0 eq.) dissolved in dry THF (6.2 mL). Carbon disulfide (0.15 mL, 2.51 mmol,

4.0 eq.) was added and the solution was cooled to -78 °C. 1 M KHMDS in THF (1.2 mL, 1.2 mmol, 1.9 eq.) was added and the resulting bright yellow solution was stirred for 30 min. The reaction mixture was then warmed to 0 °C and iodomethane (0.27 mL, 4.34 mmol, 7.0 eq.) was added. After stirring for 1.5 h at 0 °C the reaction was quenched by addition of sat. aq. NH_4Cl (15 mL). The aqueous phase was extracted with Et_2O (3 x 15 mL) and the combined organic extracts were washed with brine (20 mL), dried over $MgSO_4$ and concentrated. The crude xanthate **S38** (370 mg) was used in the next step without further purification.

A flask was charged with crude xanthate **S38** and AIBN (15.4 mg, 0.09 mmol, 0.15 eq.) and placed under N_2 atmosphere by three cycles of evacuating and N_2 backfilling. The reagents were dissolved in dry toluene (6.2 mL) and *n*-Bu_3SnH (0.33 mL, 1.23 mmol, 2.0 eq.) was added. The reaction mixture was then heated to 90 °C and stirred for 1 h. After cooling to room temperature, Et_2O (30 mL) was added and the organic phase was washed with 1M aq. NaOH (2 x 15 mL) and brine (15 mL), dried over $MgSO_4$ and concentrated. The crude was purified by flash chromatography (pentane/Et_2O 95/5) to give the product **36** (273 mg, 0.58 mmol, 93% yield over two steps) as colourless oil.

^{1}H NMR (400 MHz, Chloroform-*d*) δ 7.38 – 7.26 (m, 5H), 4.62 (s, 2H), 4.51 (s, 2H), 3.60 – 3.52 (m, 2H), 3.46 (t, *J* = 6.8 Hz, 2H), 3.36 (s, 3H), 1.84 – 1.69 (m, 4H), 1.69 – 1.57 (m, 2H), 1.55 – 1.48 (m, 4H), 1.47 – 1.36 (m, 4H), 1.36 – 1.29 (m, 5H), 1.28 – 1.21 (m, 4H), 1.16 – 1.08 (m, 2H), 1.07 – 0.94 (m, 3H), 0.89 (d, *J* = 6.5 Hz, 3H), 0.83 (s, 3H), 0.79 – 0.64 (m, 2H).

^{13}C NMR (101 MHz, Chloroform-*d*) δ 138.84, 128.46, 127.76, 127.58, 96.55, 72.96, 71.67, 66.25, 55.23, 45.69, 43.86, 38.99, 38.95, 37.97, 37.48, 37.38, 36.92, 36.57, 33.38, 32.84, 32.47, 31.70, 31.32, 30.06, 29.97, 26.03, 24.16, 22.03, 19.71.

HRMS (ESI+) M+Na$^+$ calculated for $C_{31}H_{52}O_3$: 495.3809, found: 495.3806.

Optical Rotation: $[\alpha]_D^{23}$ = +1.3° (*c* = 0.50, $CHCl_3$).

Synthesis of dithiane 10

Alcohol 37

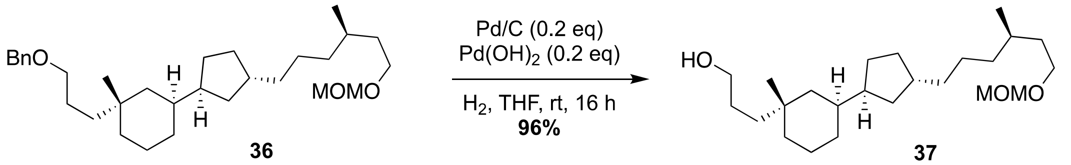

A flask was charged with benzyl ether **36** (225 mg, 0.48 mmol, 1.0 eq.), Pd/C (10% Pd by weight, 102 mg, 0.10 mmol, 0.2 eq.) and $Pd(OH)_2$ (20% Pd by weight, 66.7 mg, 0.10 mmol, 0.2 eq.). THF (4.8 mL) was added and then H_2 was bubbled through the suspension for 5 min. After that, the reaction mixture was stirred for 16 h under H_2 atmosphere (balloon) at room temperature. The reaction mixture was filtered over Celite and the filtrate was concentrated to give the product **37** (175 mg, 0,46 mmol, 96% yield) as colourless oil.

^{1}H NMR (400 MHz, Chloroform-*d*) δ 4.62 (s, 2H), 3.63 (t, *J* = 6.6 Hz, 2H), 3.58 – 3.51 (m, 2H), 3.36 (s, 3H), 1.82 – 1.69 (m, 4H), 1.63 (dq, *J* = 13.4, 6.8 Hz, 1H), 1.51 – 1.39 (m, 9H), 1.36 – 1.21 (m, 9H), 1.15 – 1.07 (m, 2H), 1.06 – 0.94 (m, 3H), 0.88 (d, *J* = 6.5 Hz, 3H), 0.83 (s, 3H), 0.77 – 0.64 (m, 2H).

^{13}C NMR (101 MHz, Chloroform-*d*) δ 96.52, 66.27, 64.11, 55.22, 45.65, 43.90, 38.97, 38.96, 37.91, 37.43, 37.31, 36.89, 36.55, 33.38, 32.76, 32.18, 31.66, 31.31, 30.05, 29.96, 27.23, 26.00, 22.00, 19.71.

HRMS (ESI+) M+Na$^+$ calculated for for $C_{24}H_{46}O_3$: 405.3339, found: 405.3334.

Optical Rotation: $[\alpha]_D^{23}$ = +14.4° (*c* = 0.12, $CHCl_3$).

Thioester 39

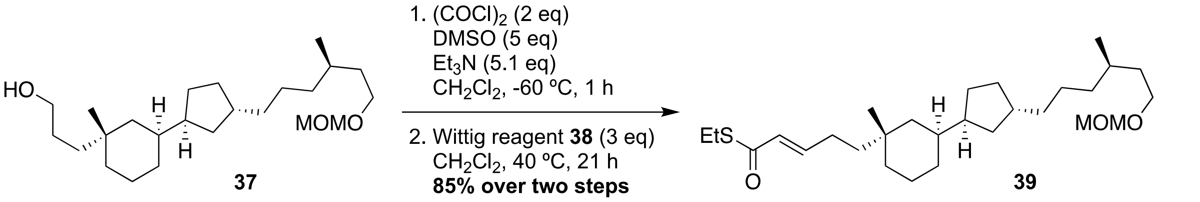

A Schlenk flask was flame-dried under vacuo and subjected to three cycles of evacuating and N_2 backfilling. The flask was charged with oxalyl chloride (0.14 mL, 1.63 mmol, 2 eq.) and dissolved in dry CH_2Cl_2 (6 mL). The solution was cooled to -78 ºC and dry DMSO (0.29 mL, 4.08 mmol, 5 eq.) was added. After stirring for 15 min at -78 ºC, alcohol **37** (315 mg, 0.82 mmol, 1 eq.) was added dissolved in CH_2Cl_2 (2.2 mL). After stirring for another 15 min, dry Et_3N (0.57 mL, 4.10 mmol, 5 eq.) was added. The reaction mixture was stirred for another 15 min at -78 C and then warmed to 0 ºC and after 15 min stirring, the reaction was quenched by addition of sat. aq. NH_4Cl (15 mL). The aqueous phase was then extracted with Et_2O (3 x 10 mL) and the combined organic extracts were washed with brine (10 mL), dried over $MgSO_4$ and concentrated. The crude aldehyde was used in the following step without further purification.

A pressure tube was placed under N_2 atmosphere by three cycles of evacuating and N_2 backfilling and charged with solid Wittig reagent **38** (900 mg, 2.47 mmol, 3 eq.) and crude aldehyde dissolved in dry CH_2Cl_2 (5.5 mL). The reaction mixture was then heated to 40 ºC and stirred for 21 h. The solvent was then removed under reduced pressure and the crude was purified by flash chromatography (pentane/Et_2O 96/4) to give the product **39** (327 mg, 0.70 mmol, 85% yield over two steps) as colourless oil.

^1H NMR (400 MHz, Chloroform-*d*) δ 6.92 (dtd, *J* = 15.3, 6.8, 1.3 Hz, 1H), 6.10 (d, *J* = 13.9 Hz, 1H), 4.61 (s, 2H), 3.59 – 3.50 (m, 2H), 3.36 (s, 3H), 2.93 (q, *J* = 7.4 Hz, 2H), 2.07 (dd, *J* = 16.5, 7.6 Hz, 2H), 1.82 – 1.70 (m, 4H), 1.62 (dt, *J* = 13.2, 6.9 Hz, 1H), 1.54 – 1.38 (m, 7H), 1.37 – 1.20 (m, 12H), 1.14 – 0.96 (m, 5H), 0.88 (d, *J* = 6.5 Hz, 3H), 0.83 (s, 3H), 0.78 – 0.65 (m, 2H).

^{13}C NMR (101 MHz, Chloroform-*d*) δ 190.32, 146.53, 128.42, 96.53, 66.23, 55.25, 45.59, 43.71, 38.99, 38.95, 37.77, 37.46, 37.35, 36.88, 36.60, 34.57, 33.36, 33.06, 31.59, 31.34, 29.94, 29.89, 26.85, 26.01, 23.15, 21.94, 19.69, 15.00.

HRMS (ESI+) M+Na$^+$ calculated for $C_{28}H_{50}O_3S$: 489.3373, found: 489.3361.

Optical Rotation: $[\alpha]_D^{23}$ = +21° (*c* = 0.65, $CHCl_3$).

Thioester 40

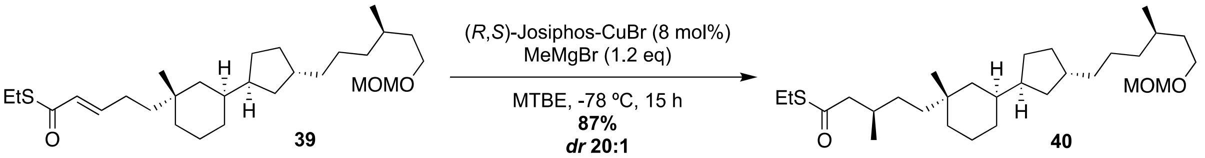

A Schlenk flask was flame-dried under vacuo and subjected to three cycles of evacuating and N_2 backfilling. The flask was charged with (*R*,*S*)-Josiphos-CuBr complex (56.4 mg, 0.07 mmol, 0.08 eq.) under N_2 flow. Dry MTBE (6 mL) was added and the resulting solution was cooled to -78 ºC. 3 M MeMgBr in Et_2O (0.35 mL, 1.05 mmol, 1.2 eq.) was added and the reaction mixture was stirred for 30 min at -78 ºC. Thioester **39** (410 mg, 0.88 mmol, 1 eq.) was dissolved in dry MTBE (2.8 mL) and added dropwise over 2 h by means of a syringe pump. The reaction mixture was stirred for another 15 h at -78 ºC and then quenched by addition of methanol (3 mL). The cooling bath was removed, sat. aq. NH_4Cl (30 mL) was added. The aqueous phase was extracted with Et_2O (3 x 25 mL). The combined organic extracts were washed with brine (10 mL), dried over $MgSO_4$ and concentrated. The crude was purified by flash chromatography (pentane/Et_2O 96/4) to give the product **40** (370 mg, 0.77 mmol, 87% yield, *dr* 20:1) as colourless oil.

^{1}H NMR (400 MHz, Chloroform-*d*) δ 4.61 (s, 2H), 3.54 (ddt, *J* = 9.7, 6.7, 3.1 Hz, 2H), 3.35 (s, 3H), 2.86 (q, *J* = 7.5 Hz, 2H), 2.54 (dd, *J* = 14.4, 6.1 Hz, 1H), 2.35 (dd, *J* = 14.4, 8.1 Hz, 1H), 1.95 (dq, *J* = 12.9, 6.7 Hz, 1H), 1.82 – 1.67 (m, 4H), 1.65 – 1.58 (m, 1H), 1.55 – 1.36 (m, 6H), 1.35 – 1.15 (m, 15H), 1.13 – 0.97 (m, 5H), 0.94 (d, *J* = 6.7 Hz, 3H), 0.88 (d, *J* = 6.6 Hz, 3H), 0.78 (s, 3H), 0.76 – 0.60 (m, 2H).

^{13}C NMR (101 MHz, Chloroform-*d*) δ 199.47, 96.55, 66.25, 55.24, 51.58, 45.69, 43.87, 38.98, 38.95, 37.96, 37.49, 37.40, 36.91, 36.56, 33.35, 33.20, 32.89, 32.00, 31.71, 31.31, 30.53, 30.01, 29.97, 26.03, 23.39, 22.03, 19.90, 19.70, 14.98.

HRMS (ESI+) M+Na$^+$ calculated for $C_{29}H_{54}O_3S$: 505.3686, found: 505.3675.

Optical Rotation: $[\alpha]_D^{23}$ = +4° (*c* = 0.24, $CHCl_3$).

Non-stereoselective conjugate addition for determination of the *dr* of **40**

A Schlenk flask was flame-dried under vacuo and subjected to three cycles of evacuating and N_2 backfilling. The flask was charged with $CuBr_2{\cdot}SMe_2$ (0.9 mg, 4.4 µmol, 0.25 eq.) and PPh_3 (2.3 mg, 8.8 µmol, 0.5 eq) as solids under N_2 flow. Dry MTBE (0.1 mL) was added and the resulting mixture was cooled to -10 ºC. 3 M MeMgBr in Et_2O (8 µL, 22.8 µmol, 1.3 eq.) was added the reaction mixture was stirred for 30 min. Then a solution of thioester X (8.2 mg, 17.6 µmol, 1 eq.) in dry MTBE (0.25 mL) was added. The reaction mixture was stirred for 2 h at -10 ºC and then quenched by addition of sat. aq. NH_4Cl (5 mL). The aqueous phase was extracted with Et_2O (3 x 5 mL) and the combined organic extracts were dried over $MgSO_4$ and concentrated. The crude product, a mixture of **40** and ***epi*-40** was analyzed by ^{13}C NMR.

Determination of the diastereomeric ratio by ^{13}C NMR

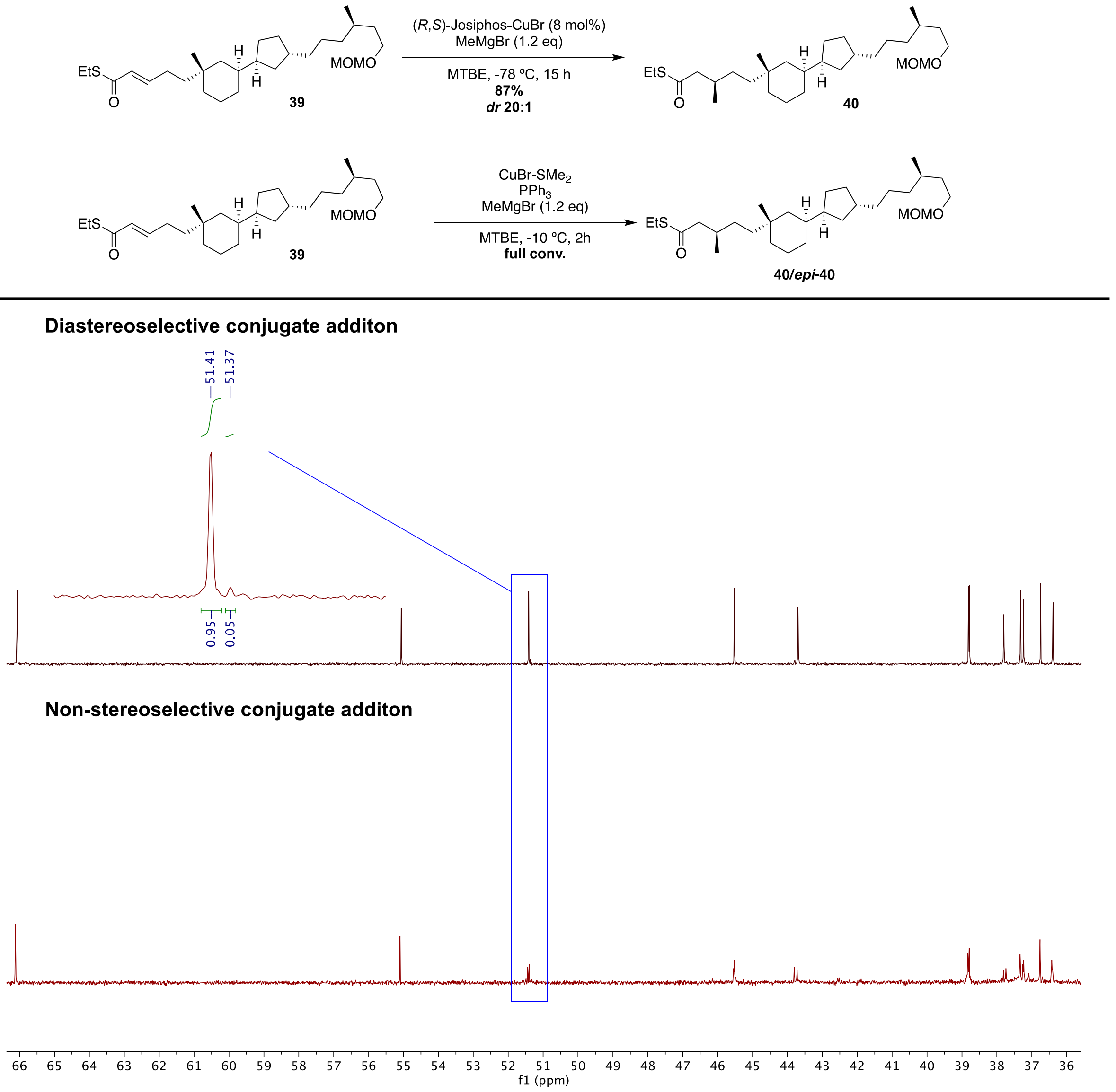

Alcohol S39

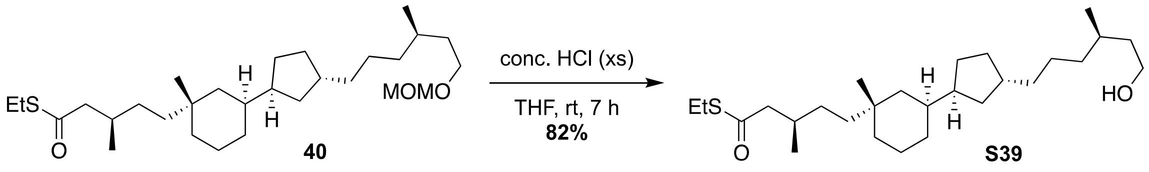

A flask was charged with **40** (41.0 mg, 0.09 mmol, 1 eq.) dissolved in THF (1.7 mL). To the stirred solution was added 12 M aq. HCl (0.25 mL, 3.0 mmol, 35 eq.) and the reaction mixture was stirred for 7 h at room temperature. Et_2O (10 mL) was added and the organic phase was washed with sat. aq. $NaHCO_3$ (2 x 7 mL) and brine (5 mL), dried over $MgSO_4$ and concentrated. The crude was purified by flash chromatography (pentane/Et_2O 2/1) to give the product **S39** (30.6 mg, 0.07 mmol, 82% yield) as colourless oil.

^{1}H NMR (400 MHz, Chloroform-*d*) δ 3.75 – 3.61 (m, 2H), 2.87 (q, *J* = 7.4 Hz, 2H), 2.54 (dd, *J* = 14.4, 6.1 Hz, 1H), 2.35 (dd, *J* = 14.4, 8.1 Hz, 1H), 2.00 – 1.90 (m, 1H), 1.83 – 1.68 (m, 4H), 1.65 – 1.52 (m, 2H), 1.50 – 1.20 (m, 20H), 1.14 – 0.99 (m, 5H), 0.94 (d, *J* = 6.7 Hz, 3H), 0.89 (d, *J* = 6.5 Hz, 3H), 0.79 (s, 3H), 0.76 – 0.60 (m, 2H).

^{13}C NMR (101 MHz, Chloroform-*d*) δ 199.56, 61.41, 51.61, 45.68, 43.86, 40.15, 38.99, 38.96, 38.00, 37.52, 37.36, 36.57, 33.37, 33.21, 32.90, 32.01, 31.71, 31.30, 30.55, 30.01, 29.64, 26.04, 23.41, 22.04, 19.91, 19.80, 14.98.

HRMS (ESI+) M+Na$^+$ calculated for $C_{27}H_{50}O_2S$: 461.3424, found: 461.3430.

Dithiane 10

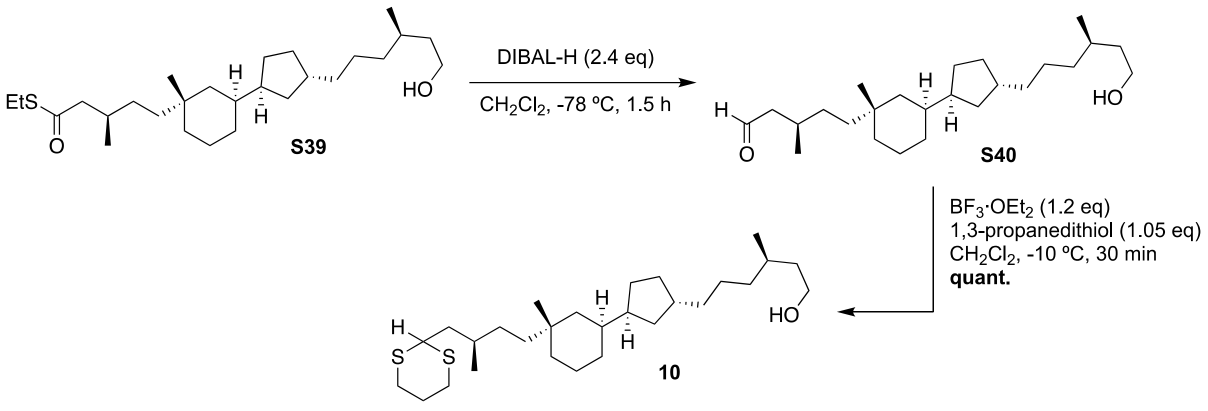

A Schlenk flask was flame-dried *in vacuo* and subjected to three cycles of evacuating and N_2 backfilling. The flask was charged with **S39** (91.7 mg, 0.21 mmol, 1 eq.) dissolved in dry CH_2Cl_2 (4.2 mL) and cooled to -78 ºC. 1 M DIBAL-H in CH_2Cl_2 (0.5 mL, 0.5 mmol, 2.4 eq.) was added and the reaction mixture was stirred for 1.5 h. The reaction was quenched by addition of sat. aq. Rochelle salt solution (5 mL) and then warmed to room temperature and stirred for 1 h. The aqueous phase was extracted with Et_2O (3 x 10 mL) and the combined organic extracts were washed with brine (5 mL), dried over $MgSO_4$ and concentrated. The crude aldehyde **S40** was used directly in the next step without purification.

A Schlenk flask was flame-dried *in vacuo* and subjected to three cycles of evacuating and N_2 backfilling. The flask was charged with crude **S40** (0.21 mmol) dissolved in dry CH_2Cl_2 (1.4 mL) and 1,3-propanedithiol (22 µL, 0.22 mmol, 1.05 eq.) and cooled to -10 ºC (NaCl/ice bath). $BF_3·OEt_2$ (30 µL, 0.24 mmol, 1.2 eq.) was added and the reaction mixture was stirred for 30 min. The reaction was quenched by addition of sat. aq. NH_4Cl (5 mL). The aqueous phase was extracted with CH_2Cl_2 (3 x 10 mL) and the combined organic extracts were dried over $MgSO_4$ and concentrated to give the product **10** (98.0 mg, 0.21 mmol, quant. yield over two steps) as colorless oil.

^{1}H NMR (400 MHz, Chloroform-*d*) δ 4.10 (dd, *J* = 8.5, 6.1 Hz, 1H), 3.73 – 3.60 (m, 2H), 2.96 – 2.76 (m, 4H), 2.11 (dtt, *J* = 11.9, 4.9, 2.3 Hz, 1H), 1.92 – 1.83 (m, 1H), 1.82 – 1.67 (m, 6H), 1.61 (ddd, *J* = 12.5, 7.1, 5.4 Hz, 1H), 1.56 – 1.48 (m, 3H), 1.47 – 1.42 (m, 2H), 1.41 – 1.31 (m, 6H), 1.28 – 1.18 (m, 7H), 1.17 – 1.06 (m, 3H), 1.05 – 0.95 (m, 3H), 0.91 (d, *J* = 6.5 Hz, 3H), 0.88 (d, *J* = 6.6 Hz, 3H), 0.78 (s, 3H), 0.76 – 0.59 (m, 2H).

^{13}C NMR (101 MHz, Chloroform-*d*) δ 61.40, 45.88, 45.69, 43.77, 42.69, 40.15, 39.01, 38.97, 38.15, 37.52, 37.36, 36.59, 33.40, 33.08, 32.91, 31.76, 31.33, 30.81, 30.60, 30.57, 30.56, 30.04, 29.65, 26.32, 26.06, 22.04, 19.81, 19.75.

HRMS (ESI+) M+Na+ calculated for $C_{28}H_{52}OS_2$: 469.3532, found: 469.3517.

Optical Rotation: $[\alpha]_D^{23}$ = -6.2° (*c* = 0.21, $CHCl_3$).

Dithiane 8

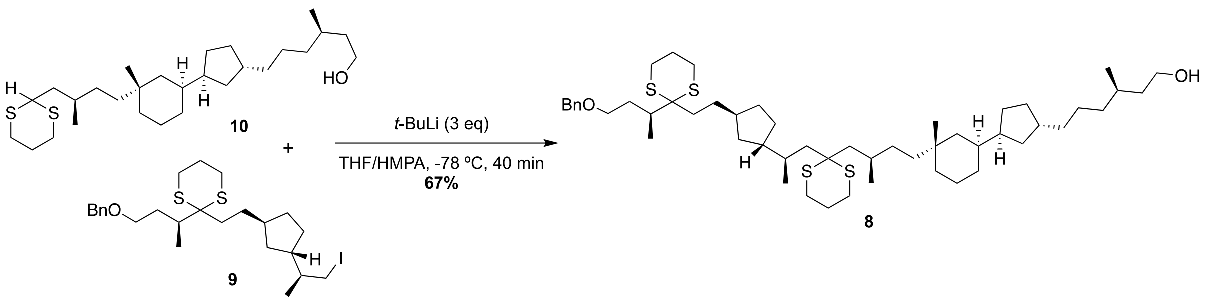

A Schlenk flask was flame-dried *in vacuo* and subjected to three cycles of evacuating and N_2 backfilling and charged with dithiane **10** (128 mg, 0.27 mmol, 1.5 eq.) dissolved in dry THF (2.2 mL). Dry HMPA (0.37 mL) was added and the solution was cooled to -78 ºC. 1.7 M *t*-BuLi in hexanes (0.32 mL, 0.54 mmol, 3 eq.) was added and the reaction mixture was stirred for 10 minutes. Then, iodide **9** (100 mg, 0.18 mmol, 1 eq.) was added, dissolved in dry THF (1.5 mL) at -78 ºC. The reaction mixture was stirred for 30 min and then quenched by addition of sat. aq. NH_4Cl (15 mL). The aqueous phase was extracted with Et_2O (3 x 15 mL). The combined organic extracts were washed with brine (10 mL), dried over $MgSO_4$ and concentrated. The crude product was purified by flash chromatography (pentane/acetone 92/8) to give product **8** (109 mg, 0.12 mmol, 67% yield) as a colorless oil.

^{1}H NMR (400 MHz, Chloroform-*d*) δ 7.37 – 7.26 (m, 5H), 4.56 (d, *J* = 12.0 Hz, 1H), 4.48 (d, *J* = 12.0 Hz, 1H), 3.73 – 3.62 (m, 2H), 3.60 – 3.47 (m, 2H), 2.87 – 2.70 (m, 8H), 2.39 (dt, *J* = 14.9, 7.8 Hz, 1H), 2.15 (ddt, *J* = 11.4, 7.0, 3.5 Hz, 1H), 1.99 – 1.83 (m, 9H), 1.83 – 1.65 (m, 11H), 1.63 – 1.19 (m, 28H), 1.15 – 1.11 (m, 2H), 1.09 (d, *J* = 6.8 Hz, 4H), 1.02 (t, *J* = 6.1 Hz, 6H), 0.89 (d, *J* = 6.4 Hz, 3H), 0.80 (s, 3H), 0.75 – 0.58 (m, 2H).

^{13}C NMR (101 MHz, Chloroform-*d*) δ 138.79, 128.45, 127.69, 127.59, 72.77, 69.30, 61.38, 59.31, 55.37, 47.56, 46.02, 45.72, 43.62, 40.14, 39.78, 39.05, 39.01, 38.40, 37.54, 37.36, 36.58, 35.64, 35.33, 34.59, 34.16, 33.54, 33.41, 33.01, 32.89, 31.90, 31.79, 31.49, 31.37, 31.08, 30.72, 30.08, 29.63, 26.78, 26.07, 25.88, 25.72, 25.45, 25.23, 22.48, 22.07, 20.44, 19.80, 14.58.

HRMS (ESI+) M+H+ calculated for $C_{53}H_{90}O_2S_4$: 887.5896, found: 887.5871.

Optical Rotation: $[\alpha]_D^{23}$ = -12.5° (*c* = 0.12, $CHCl_3$).

Fragment B

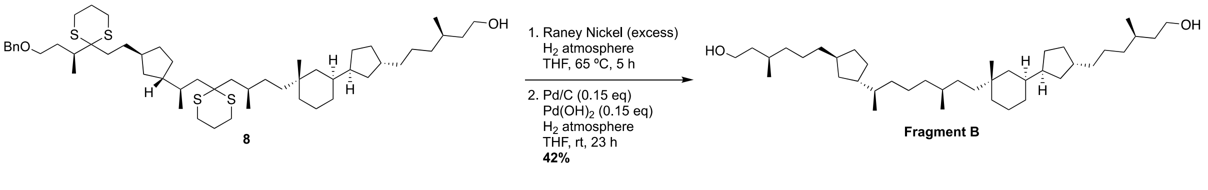

A flask was charged with Raney Nickel (2 mL, 50% dispersion in water, 10 eq. by wt., W.R. Grace and Co. Raney®2800, purchased from Sigma-Aldrich) and washed with

THF (3 x 5 mL), then **8** (100 mg, 0.11 mmol, 1 eq.) was added, dissolved in THF (2.3 mL). H_2 was bubbled through the suspension for 2 min. The suspension was then heated to 65 °C and stirred for 5 h under H_2 atmosphere (balloon). The suspension was cooled to room temperature and the solids were removed by filtration over Celite. The filtrate was concentrated and the crude desulfurized product was dissolved in THF (2.3 mL). Pd/C (10% Pd by weight, 18.0 mg, 0.02 mmol, 0.15 eq.) and $Pd(OH)_2$ (20% Pd by weight, 11.9 mg, 0.02 mmol, 0.15 eq.) were added and H_2 was bubbled through the suspension for 5 min. The suspension was stirred at room temperature under H_2 atmosphere for 23 h. The reaction mixture was filtered over Celite and the filtrate was concentrated. The crude product was purified by flash chromatography (pentane/EtOAc 4/1) to give **Fragment B** (28.1 mg, 0.05 mmol, 42% yield) as colorless oil.

^{1}H NMR (600 MHz, Chloroform-*d*) δ 3.72 – 3.63 (m, 4H), 1.80 – 1.65 (m, 7H), 1.60 (dtd, *J* = 12.7, 7.2, 5.1 Hz, 2H), 1.56 – 1.52 (m, 2H), 1.49 – 1.45 (m, 4H), 1.41 – 1.33 (m, 8H), 1.31 – 1.20 (m, 18H), 1.18 – 1.02 (m, 10H), 1.01 – 0.96 (m, 2H), 0.89 (d, *J* = 6.6 Hz, 6H), 0.86 (d, *J* = 6.5 Hz, 3H), 0.84 (d, *J* = 6.6 Hz, 3H), 0.79 (s, 3H), 0.72 (qd, *J* = 12.7, 4.0 Hz, 1H), 0.67 – 0.61 (m, 1H).

^{13}C NMR (151 MHz, Chloroform-*d*) δ 61.42, 61.42, 45.73, 44.96, 43.90, 40.15, 40.14, 39.24, 39.01, 39.00, 38.45, 38.16, 37.60, 37.55, 37.52, 37.40, 37.31, 36.61, 36.16, 35.87, 33.71, 33.51, 33.39, 33.36, 32.92, 31.80, 31.41, 31.34, 30.50, 30.12, 29.65, 29.63, 26.05, 24.53, 22.10, 20.25, 19.79, 17.90.

HRMS (ESI+) $M+H^+$ calculated for $C_{40}H_{76}O_2$: 589.5918, found: 589.5900.

Optical Rotation: $[\alpha]_D^{23}$ = -4.5° (*c* = 0.11, $CHCl_3$).

Preparation of glycerol building block 2

Diol S42

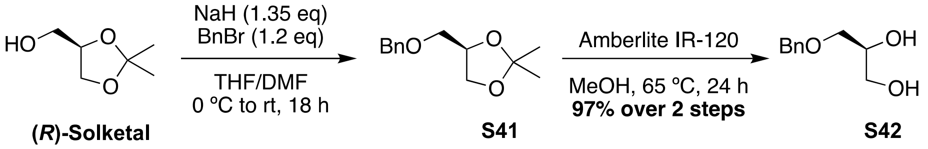

A Schlenk flask was flame-dried under vacuo and subjected to three cycles of evacuating and N_2 backfilling. The flask was charged NaH (60% in mineral oil, 2.00 g, 49.9 mmol, 1.35 eq.) under N_2 flow. The NaH dispersion was washed with pentane (3 x 4 mL) and dried under vacuo. Dry THF (28 mL) was added and the suspension was cooled to 0 ºC. A solution of (R)-solketal (4.88 g, 37.0 mmol, 1 eq.) in dry THF (9 mL) was added and after complete addition the cooling bath was removed. To the thick suspension was added a solution of benzyl bromide (5.27 mL, 44.34 mmol, 1.2 eq.) in dry DMF (5 mL). The suspension was stirred for 18 h at room temperature and then cooled to 0 ºC and quenched by slow addition of water (40 mL). The layers were separated and the aqueous layer was extracted with Et_2O (3 x 40 mL). The combined organic layers were washed with brine, dried over $MgSO_4$ and concentrated. The crude benzyl ether **S41** was used in the next step without purification.

^{1}H NMR (400 MHz, Chloroform-*d*) δ 7.39 – 7.24 (m, 5H), 4.60 (d, *J* = 12.1 Hz, 1H), 4.55 (d, *J* = 12.1 Hz, 1H), 4.35 – 4.25 (m, 1H), 4.05 (dd, *J* = 8.3, 6.4 Hz, 1H), 3.74 (dd, *J* = 8.3, 6.3 Hz, 1H), 3.56 (dd, *J* = 9.8, 5.7 Hz, 1H), 3.47 (dd, *J* = 9.8, 5.5 Hz, 1H), 1.43 (s, 3H), 1.37 (s, 3H).

^{13}C NMR (101 MHz, Chloroform-*d*) δ 138.05, 128.46, 127.79, 127.77, 109.45, 74.81, 73.56, 71.16, 66.94, 26.84, 25.47.

HRMS (ESI+) M+Na$^+$ calculated for $C_{13}H_{18}O_3$: 245.1148, found: 245.1147.

Optical Rotation: $[\alpha]_D^{23}$ = -29.2° (*c* = 0.12, $CHCl_3$).

A flask was charged with crude **S41** which was dissolved in methanol (30 mL). Amberlite IR-120 resin (3 g) was added and the reaction mixture was stirred for 21 h at room temperature. When NMR sampling indicated incomplete conversion, additional Amberlite IR-120 resin (2 g) was added and the reaction mixture was heated to 65 °C and stirred for 18 h. The reaction mixture was allowed to come to room temperature, the resin was filtered off and the filtrate was concentrated to give the product **S42** (6.50 g, 35.6 mmol, 97% yield).

^1H NMR (400 MHz, Chloroform-*d*) δ 7.38 – 7.26 (m, 5H), 4.54 (s, 2H), 3.91 – 3.85 (m, 1H), 3.70 – 3.64 (m, 1H), 3.63 – 3.49 (m, 3H), 2.98 – 2.80 (m, 2H).

^{13}C NMR (101 MHz, Chloroform-*d*) δ 137.80, 128.62, 128.01, 127.91, 73.68, 71.86, 70.81, 64.17, 64.15.

HRMS (ESI+) M+Na$^+$ calculated for $C_{10}H_{14}O_3$: 205.0835, found: 205.0835.

Optical Rotation: $[\alpha]_D^{23}$ = -0.7° (*c* = 0.46, $CHCl_3$).

The analytical data is in agreement with previous reports.[19]

Trityl ether 2

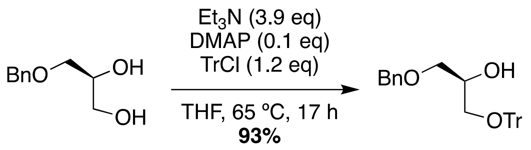

S42 **2**

A flask was charged with DMAP (405 mg, 3.31 mmol, 0.1 eq.) and trityl chloride (11.0 g, 39.4 mmol, 1.2 eq.) and placed under N_2 by three cycles of evacuating and N_2 backfilling. Dry triethylamine (18.0 mL, 129 mmol, 4 eq.) was added followed by addition of a solution of diol **S42** (5.98 g, 32.8 mmol, 1 eq.) in dry THF (44 mL). The solution was heated to 65 °C and stirred for 17 h. After cooling to room temperature, water (100 mL) was added and the aqueous phase was extracted with Et_2O (3 x 75 mL). The combined organic extracts were washed with brine (75 mL), dried over $MgSO_4$ and concentrated. The crude was purified by flash chromatography (pentane/Et_2O 5/2 to 4/2) to give the product **2** (13.0 g, 30.5 mmol, 93% yield) as off-white solid.

^1H NMR (400 MHz, Chloroform-*d*) δ 7.45 (d, *J* = 7.4 Hz, 6H), 7.38 – 7.22 (m, 14H), 4.55 (s, 2H), 4.01 (h, *J* = 5.3 Hz, 1H), 3.60 (qd, *J* = 9.6, 5.3 Hz, 2H), 3.30 – 3.20 (m, 2H), 2.45 (d, *J* = 4.8 Hz, 1H).

^{13}C NMR (101 MHz, Chloroform-*d*) δ 143.97, 138.14, 128.80, 128.53, 128.01, 127.97, 127.82, 127.81, 127.19, 86.80, 73.50, 71.68, 70.07, 64.70.

HRMS (ESI+) M+Na$^+$ calculated for $C_{29}H_{28}O_3$: 447.1931, found: 447.1932.

The analytical data is in agreement with previous reports.[19]

Crenarchaeol assembly

Mesylate 41

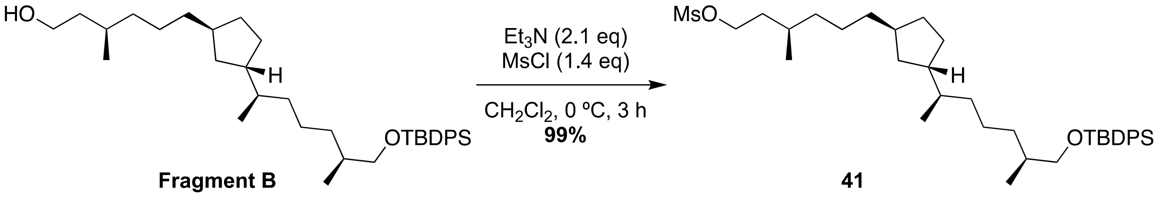

A flask was placed under N_2 atmosphere by three cycles of evacuating and N_2 backfilling, then charged with **Fragment B** (151 mg, 0.27 mmol, 1.0 eq.) dissolved in 2.7 mL dry CH_2Cl_2. The solution was cooled to 0 °C and dry Et_3N (0.08 mL, 0.58 mmol, 2.1 eq.) was added, followed by mesyl chloride (0.03 mL, 0.39 mmol, 1.4 eq.). The reaction mixture was stirred for 3 h at 0 °C. The reaction was quenched by addition of sat. aq. NH_4Cl (10 mL) and allowed to come to room temperature. The aqueous phase was extracted with Et_2O (3 x 10 mL) and the combined organic extracts were washed with 1 M aq. HCl (2 x 10 mL) and brine (10 mL), dried over $MgSO_4$ and concentrated to give the product **41** (171 mg, 0.27 mmol, 99% yield) as colourless oil.

^{1}H NMR (400 MHz, Chloroform-*d*) δ 7.67 (d, *J* = 7.8 Hz, 4H), 7.45 – 7.34 (m, 6H), 4.32 – 4.22 (m, 2H), 3.51 (dd, *J* = 10.1, 5.9 Hz, 1H), 3.43 (dd, *J* = 9.9, 6.3 Hz, 1H), 3.00 (s, 3H), 1.80 – 1.71 (m, 3H), 1.68 – 1.60 (m, 2H), 1.58 – 1.49 (m, 2H), 1.44 – 1.33 (m, 4H), 1.31 – 1.20 (m, 8H), 1.18 – 0.95 (m, 15H), 0.92 (d, *J* = 6.5 Hz, 6H), 0.81 (d, *J* = 6.6 Hz, 3H).

^{13}C NMR (101 MHz, Chloroform-*d*) δ 135.77, 134.28, 129.60, 127.69, 69.00, 68.77, 44.94, 39.16, 38.36, 37.53, 37.21, 37.13, 36.13, 35.90, 35.80, 33.71, 33.49, 31.40, 29.49, 27.02, 25.92, 24.45, 19.48, 19.41, 17.86, 17.20.

HRMS (ESI+) $M+H^+$ calculated for $C_{37}H_{60}O_4SSi$: 629.4054, found: 629.4048.

Tritylether 42

Tested conditions for the *O*-alkylation of mesylate 41 with alcohol 2

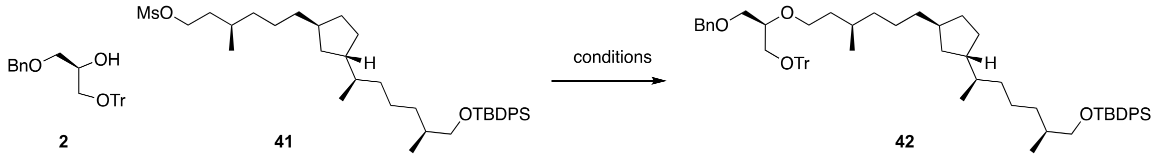

Entry	X	Base	Solvent	Temp.	Time	Yield[a]
1	1.5 eq.	NaH (4.5 eq.)	DMSO	rt	17 h	24%[b]
2	1.5 eq.	KO*t*-Bu (3 eq.)[c]	$PhCH_3$	rt	16 h	41%[d]
3	2 eq.	NaH (2 eq.)	DMF	rt	17 h	62%[b]

[a] Isolated yield. [b] After resilylation. [c] TBAB (3 eq.) was added. [d] Elimination of mesylate observed.

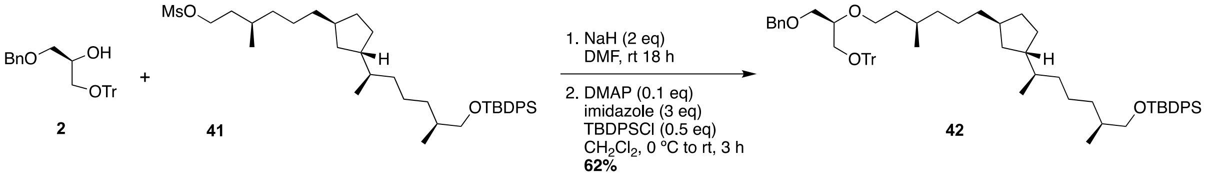

A Schlenk flask was flame-dried under vacuo and subjected to three cycles of evacuating and N_2 backfilling. The flask was charged with NaH (60% in mineral oil, 21.5 mg, 0.54 mmol, 2 eq.) under N_2 flow. The NaH dispersion was washed with pentane (3 x 0.5 mL) and dried under vacuo. A solution of alcohol **2** (230 mg, 0.54 mmol, 2 eq.) in dry DMF (1.3 mL) was added at room temperature and the resulting mixture was stirred for 1 h. A solution of mesylate **41** (171 mg, 0.27 mmol, 1 eq.) in dry DMF (1.4 mL) was added and the reaction mixture was stirred for 17 h. The reaction was quenched by slow addition of sat. aq. NH_4Cl (15 mL) and the aqueous phase was extracted with Et_2O (3 x 15 mL). The combined organic extracts were washed with brine (15 mL), dried over $MgSO_4$ and concentrated. The crude (partially desilylated) was dissolved in dry CH_2Cl_2 (7.7 mL) under an atmosphere of N_2. Imidazole (52.9 mg, 0.78 mmol, 3 eq.) and DMAP (3.3 mg, 0.03 mmol, 0.1 eq.) were added under N_2 flow and the resulting solution was cooled to 0 °C. TBDPSCl (33 µL, 0.13 mmol, 0.5 eq) was added and the reaction mixture was warmed to room temperature and stirred for 3 h. The reaction was quenched by addition of sat. aq. NH_4Cl (7 mL) and the aqueous phase was extracted with Et_2O (3 x 10 mL). The combined organic extracts were washed with brine (5 mL), dried over $MgSO_4$ and concentrated. The crude was purified by flash chromatography (pentane/Et_2O 95/5) to give the product **42** (161 mg, 0.17 mmol, 62% yield) as colourless oil.

^1H NMR (600 MHz, Chloroform-*d*) δ 7.70 – 7.65 (m, 4H), 7.48 – 7.44 (m, 5H), 7.44 – 7.40 (m, 2H), 7.38 (dd, *J* = 7.9, 6.3 Hz, 4H), 7.34 – 7.21 (m, 15H), 4.57 – 4.48 (m, 2H), 3.65 – 3.51 (m, 6H), 3.44 (dd, *J* = 9.8, 6.4 Hz, 1H), 3.22 (d, *J* = 4.6 Hz, 2H), 1.82 – 1.70 (m, 3H), 1.68 – 1.61 (m, 3H), 1.59 – 1.55 (m, 1H), 1.42 – 1.34 (m, 4H), 1.31 – 1.20 (m,

8H), 1.14 – 0.99 (m, 15H), 0.93 (d, J = 6.7 Hz, 3H), 0.87 (d, J = 6.6 Hz, 3H), 0.82 (d, J = 6.6 Hz, 3H).

^{13}C NMR (101 MHz, Chloroform-*d*) δ 144.27, 138.61, 135.77, 134.32, 134.31, 129.59, 128.89, 128.41, 127.87, 127.69, 127.66, 127.56, 127.04, 86.70, 78.51, 73.41, 70.69, 69.15, 69.03, 63.69, 44.96, 39.24, 38.37, 37.60, 37.38, 37.34, 36.17, 35.91, 35.83, 33.74, 33.48, 31.42, 29.96, 27.04, 26.05, 24.47, 19.82, 19.48, 17.87, 17.21.

HRMS (ESI+) M+Na$^+$ calculated for $C_{65}H_{84}O_4Si$: 979.6031, found: 979.6043.

Optical Rotation: $[\alpha]_D^{23}$ = -5.5° (c = 0.11, $CHCl_3$).

Alcohol 43

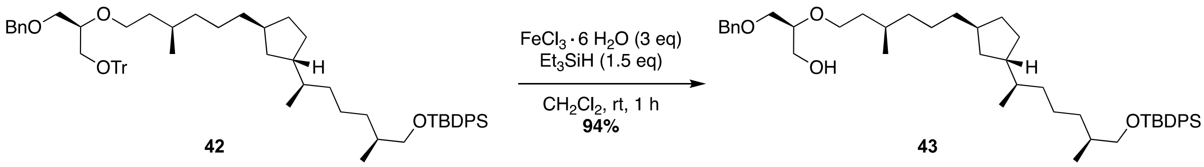

A flask was charged with tritylether **42** (55.5 mg, 59 µmol, 1 eq.) and dissolved in CH_2Cl_2 (0.6 mL). To the solution was added $FeCl_3$ hexahydrate (46.5 mg, 0.176 mmol, 3 eq.) followed by Et_3SiH (14 µL, 88 µmol, 1.5 eq.). The reaction mixture was stirred at room temperature for 1 h and then diluted with Et_2O (10 mL). The organic layer was washed with water (5 mL) and brine (5 ml), dried over $MgSO_4$ and concentrated. The crude purified by flash chromatography (pentane/Et_2O 3/2) to give the product **43** (38.7 mg, 55 µmol, 94% yield) as colourless oil.

^1H NMR (400 MHz, Chloroform-*d*) δ 7.67 (d, J = 6.2 Hz, 4H), 7.45 – 7.27 (m, 11H), 4.55 (s, 2H), 3.78 – 3.71 (m, 1H), 3.69 – 3.48 (m, 7H), 3.44 (dd, J = 9.0, 6.7 Hz, 1H), 2.01 (br s, 1H), 1.83 – 1.71 (m, 3H), 1.69 – 1.58 (m, 3H), 1.57 – 1.49 (m, 1H), 1.44 – 1.34 (m, 4H), 1.33 – 1.18 (m, 8H), 1.16 – 0.96 (m, 15H), 0.93 (d, J = 6.7 Hz, 3H), 0.88 (d, J = 6.5 Hz, 3H), 0.82 (d, J = 6.6 Hz, 3H).

^{13}C NMR (101 MHz, Chloroform-*d*) δ 138.17, 135.78, 134.32, 129.59, 128.57, 127.86, 127.79, 127.69, 78.61, 73.68, 70.18, 69.03, 68.84, 63.06, 44.95, 39.23, 38.36, 37.51, 37.34, 37.25, 36.16, 35.91, 35.83, 33.74, 33.49, 31.41, 29.95, 27.04, 26.04, 24.47, 19.80, 19.48, 17.86, 17.21.

HRMS (ESI+) M+Na$^+$ calculated for $C_{46}H_{70}O_4Si$: 737.4936, found: 737.4928.

Optical Rotation: $[\alpha]_D^{23}$ = -7.6° (c = 0.17, $CHCl_3$).

Bis-mesylate 44

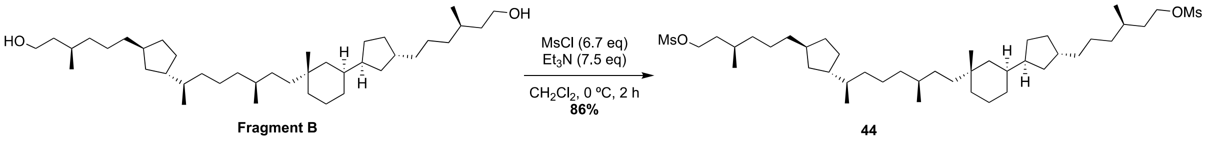

A flask was charged with **Fragment B** (22.5 mg, 38 µmol, 1 eq.) and placed under N_2 atmosphere by three cycles of evacuating and N_2 backfilling. The substrate was dissolved in dry CH_2Cl_2 (0.75 mL) and cooled to 0 ºC. Dry triethylamine (0.04 mL, 0.29 mmol, 7.5 eq.) was added followed by addition of MsCl (0.02 mL, 0.26 mmol, 6.7 eq.). The reaction mixture was stirred for 2 h at 0 ºC and then quenched by addition of sat.

aq. NH_4Cl (5 mL). The aqueous phase was extracted with Et_2O (3 x 5 mL) and the combined organic extracts were washed with 1 M aq. HCl (10 mL) and brine (5 mL), dried over $MgSO_4$ and concentrated. The crude bis-mesylate **44** (24.4 mg, 33 µmol, 86% yield) was used without further purification.

^{1}H NMR (400 MHz, Chloroform-*d*) δ 4.26 (ddt, *J* = 9.8, 7.4, 3.3 Hz, 4H), 3.00 (s, 6H), 1.82 – 1.73 (m, 7H), 1.70 – 1.65 (m, 1H), 1.61 – 1.45 (m, 7H), 1.39 – 1.19 (m, 26H), 1.17 – 0.96 (m, 12H), 0.91 (d, *J* = 6.4 Hz, 6H), 0.84 (dd, *J* = 9.1, 6.5 Hz, 6H), 0.79 (s, 3H), 0.76 – 0.61 (m, 2H).

^{13}C NMR (101 MHz, Chloroform-*d*) δ 68.76, 45.70, 44.95, 43.94, 39.17, 38.94, 38.43, 38.05, 37.56, 37.52, 37.28, 37.19, 37.16, 37.14, 37.12, 36.56, 36.14, 35.85, 33.66, 33.47, 33.33, 33.30, 32.90, 31.78, 31.40, 31.29, 30.50, 30.11, 29.49, 25.89, 24.51, 22.07, 20.21, 19.40, 17.88.

Diol 45

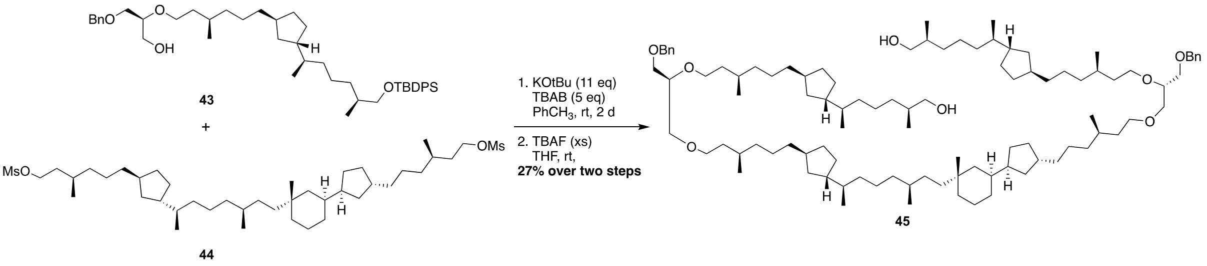

A Schlenk flask was flame-dried under vacuo and subjected to three cycles of evacuating and N_2 backfilling. The flask was charged with tetrabutylammonium bromide (TBAB, 52.8 mg, 0.16 mmol, 5 eq.) and KO*t*Bu (14.7 mg, 0.13 mmol, 4 eq., stored and weighed out in a glovebox) as solids under N_2 flow. A solution of bis-mesylate **44** (24.4 mg, 33 µmol, 1 eq.) and alcohol **43** (70.2 mg, 98 µmol, 3 eq.) in dry toluene (0.65 mL) was added and the resulting mixture was stirred at room temperature. After stirring for 5 h, additional KO*t*Bu (16.1 mg, 0.14 mmol, 4.3 eq.) was added under N_2 flow. After stirring for another 21 h at rt, additional KO*t*Bu (9.8 mg, 87 µmol, 2.6 eq.) was added. The reaction mixture was stirred for another 24 h and then quenched by addition of sat. aq. NH_4Cl (10 mL). The aqueous phase was extracted with Et_2O (3 x 10 mL). The combined organic extracts were dried over $MgSO_4$ and the crude was purified by flash chromatography (pentane/EtOAc 98/2 to 95/5 to 80/20) to give a mixture of silyl protected diol and free diol **45**. The mixture was then dissolved in THF (0.65 mL) and 1 M TBAF in THF (0.16 mL, 0.16 mmol) was added and the reaction was stirred for 20 h at rt. The reaction was quenched by addition of sat. aq. NH_4Cl (10 mL) and the aqueous phase was extracted with Et_2O (3 x 10 mL). The combined organic extracts were dried over $MgSO_4$ and concentrated. The crude was purified by flash chromatography (pentane/EtOAc 5/1 to 3/1) to give the product **45** (13.6 mg, 9.0 µmol, 27% yield over two steps) as colourless oil.

^{1}H NMR (400 MHz, Chloroform-*d*) δ 7.33 (d, *J* = 4.3 Hz, 8H), 7.28 (dd, *J* = 4.9, 3.7 Hz, 2H), 4.55 (s, 4H), 3.64 – 3.55 (m, 8H), 3.54 – 3.39 (m, 14H), 1.82 – 1.69 (m, 15H), 1.68 – 1.58 (m, 10H), 1.55 – 1.47 (m, 8H), 1.41 – 1.32 (m, 18H), 1.29 – 1.20 (m, 30H), 1.16 – 1.02 (m, 20H), 0.92 (d, *J* = 6.7 Hz, 6H), 0.85 (dd, *J* = 9.6, 6.5 Hz, 24H), 0.80 (s, 3H), 0.75 – 0.70 (m, 1H), 0.65 (t, *J* = 12.7 Hz, 1H).

^{13}C NMR (101 MHz, Chloroform-*d*) δ 138.60, 128.46, 127.73, 127.65, 78.11, 73.51, 70.97, 70.50, 70.14, 69.05, 68.52, 45.74, 45.00, 44.95, 44.01, 39.25, 39.04, 38.96, 37.60, 37.57, 37.48, 37.36, 37.29, 36.85, 36.61, 36.16, 35.97, 35.89, 35.81, 33.73,

33.69, 33.50, 33.36, 33.30, 32.92, 31.81, 31.40, 31.31, 30.54, 30.13, 30.03, 29.94, 29.85, 26.06, 25.90, 24.53, 22.10, 20.21, 19.82, 17.93, 17.88, 16.86.

HRMS (ESI+) M+NH_4^+ calculated for $C_{100}H_{176}O_8$: 1524.3737, found: 1524.3760.

Alkene 1

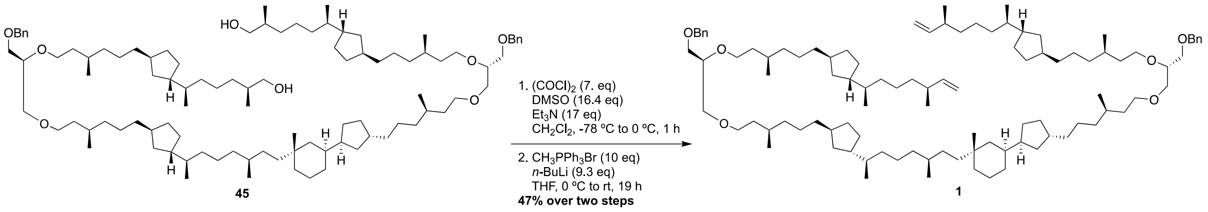

A Schlenk flask was flame-dried under vacuo and subjected to three cycles of evacuating and N_2 backfilling. The flask was charged with oxalyl chloride (5 µL, 60 µmol, 7 eq.) and dry CH_2Cl_2 (0.1 mL) and cooled to -78 ºC (dry ice/acetone). Dry DMSO (10 µL, 0.14 mmol, 16.4 eq.) was added and the resulting suspension was stirred for 20 min. Then a solution of diol **45** (12.9 mg, 8.6 µmol, 1 eq.) was added, dissolved in dry CH_2Cl_2 (0.3 mL). The reaction mixture was stirred at -78 ºC for 25 min. Then dry Et_3N (20 µL, 0.15 mmol, 17 eq.) was added and stirred for another 15 min at -78 ºC and then warmed to 0 ºC and stirred for 15 min. The reaction was quenched by addition of sat. aq. NH_4Cl (5 mL) and allowed to come to rt. The aqueous phase was extracted with CH_2Cl_2 (3 x 8 mL). The combined organic extracts were dried over $MgSO_4$ and concentrated. The crude dialdehyde was used directly in the following step without further purification.

A Schlenk flask was flame-dried under vacuo and subjected to three cycles of evacuating and N_2 backfilling. The flask was charged with methyltriphenylphosphonium bromide (30.7 mg, 86 µmol, 10 eq.) as solid under N_2 flow. Dry THF (0.1 mL) was added and the resulting suspension was cooled to 0 ºC. 1.6 M *n*-BuLi (50 µL, 80 µmol, 9.3 eq.) was added. After stirring for 5 min at 0 ºC, the suspension was stirred at rt for 45 min. After cooling again to 0 ºC, a solution of crude dialdehyde (8.6 µmol, 1 eq.) in dry THF (0.3 mL) was added. After stirring for 5 more min at 0 ºC the reaction mixture was stirred for 19 h at rt. The reaction was quenched by addition of sat. aq. NH_4Cl (5 mL) and the aqueous phase was extracted with CH_2Cl_2 (3 x 10 mL). The combined organic extracts were dried over $MgSO_4$ and concentrated. The crude product was purified by flash chromatography (pentane/EtOAc 98/2) to give the product **1** (6.1 mg, 4.1 µmol, 47% yield over two steps) as colorless oil.

^{1}H NMR (600 MHz, Chloroform-*d*) δ 7.33 (d, *J* = 4.4 Hz, 8H), 7.29 – 7.27 (m, 2H), 5.69 (ddd, *J* = 17.2, 10.3, 7.5 Hz, 2H), 4.94 (ddd, *J* = 17.2, 2.0, 1.2 Hz, 2H), 4.90 (ddd, *J* = 10.3, 2.0, 0.9 Hz, 2H), 4.55 (s, 4H), 3.66 – 3.55 (m, 8H), 3.55 – 3.41 (m, 10H), 2.13 – 2.07 (m, 2H), 1.80 – 1.70 (m, 15H), 1.70 – 1.56 (m, 8H), 1.53 – 1.44 (m, 8H), 1.41 – 1.33 (m, 15H), 1.30 – 1.19 (m, 38H), 1.13 – 1.01 (m, 17H), 0.98 (d, *J* = 6.8 Hz, 6H), 0.87 – 0.85 (m, 15H), 0.83 (t, *J* = 7.0 Hz, 9H), 0.79 (s, 3H), 0.72 (qd, *J* = 12.9, 4.4 Hz, 1H), 0.65 (t, *J* = 12.6 Hz, 1H).

^{13}C NMR (151 MHz, Chloroform-*d*) δ 145.16, 138.61, 128.46, 127.73, 127.65, 112.37, 78.11, 73.51, 70.97, 70.50, 70.14, 69.04, 45.74, 45.01, 44.01, 39.26, 39.24, 39.04, 38.96, 38.49, 38.37, 38.02, 37.94, 37.60, 37.58, 37.56, 37.48, 37.39, 37.37, 37.35, 37.29, 37.28, 37.20, 36.85, 36.61, 36.22, 35.89, 35.55, 33.69, 33.52, 33.50, 33.36, 33.30, 32.92, 31.82, 31.45, 31.41, 31.32, 30.54, 30.14, 30.04, 29.94, 26.08, 26.05, 24.73, 24.55, 22.10, 20.47, 20.20, 19.83, 19.81, 17.93, 17.87.

HRMS (ESI+) M+Na$^+$ calculated for $C_{102}H_{176}O_6$: 1521.3393, found: 1521.3403.

Alkene 46

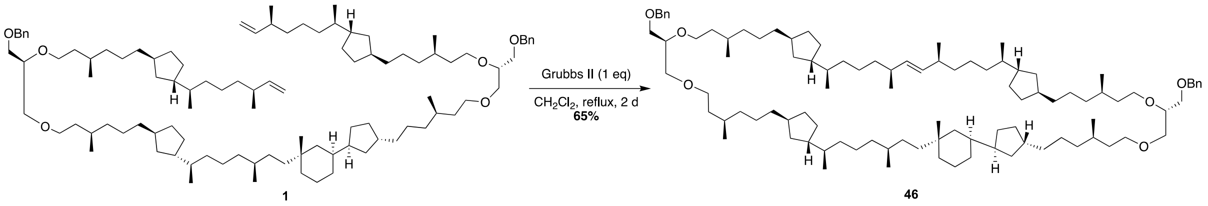

A Schlenk flask was placed under N_2 atmosphere by three cycles of evacuating and N_2 backfilling and charged with Grubbs 2nd Generation catalyst (3.1 mg, 3.7 µmol, 1 eq.) under N_2 flow. A solution of alkene **1** (5.5 mg, 3.7 µmol, 1 eq.) in dry CH_2Cl_2 (3.7 mL) was added. The flask was wrapped in aluminium foil to exclude light and the reaction mixture was then heated to 40 ºC and stirred for 48 h at this temperature. The reaction mixture was filtered over a short plug of silica (packed with pentane and flushed with 9/1 pentane/EtOAc). The filtrate was concentrated and the crude product was purified by flash chromatography (pentane/EtOAc 98/2 to 96/4) to give the product **46** (3.5 mg, 2.4 µmol, 65% yield) as colourless oil.

^{1}H NMR (600 MHz, Chloroform-*d*) δ 7.35 (d, *J* = 4.4 Hz, 8H), 7.33 – 7.28 (m, 2H), 5.16 (dd, *J* = 4.9, 2.3 Hz, 2H), 4.57 (s, 4H), 3.67 – 3.56 (m, 8H), 3.57 – 3.46 (m, 10H), 2.07 – 2.01 (m, 2H), 1.81 – 1.71 (m, 15H), 1.70 – 1.47 (m, 37H), 1.46 – 1.34 (m, 22H), 1.20 – 1.02 (m, 27H), 0.96 (d, *J* = 6.7 Hz, 6H), 0.93 – 0.90 (m, 6H), 0.86 (d, *J* = 6.7 Hz, 12H), 0.84 (d, *J* = 6.6 Hz, 6H), 0.82 (s, 3H), 0.77 – 0.71 (m, 1H), 0.65 (t, *J* = 12.5 Hz, 1H).

^{13}C NMR (151 MHz, Chloroform-*d*) δ 138.59, 134.93, 128.46, 127.73, 127.65, 78.11, 73.51, 71.19, 71.14, 70.49, 70.08, 70.04, 68.94, 45.74, 45.03, 44.93, 43.76, 39.26, 39.23, 39.11, 39.02, 38.43, 37.85, 37.57, 37.51, 37.42, 37.37, 37.24, 37.16, 36.80, 36.64, 36.23, 36.14, 35.84, 35.55, 33.69, 33.58, 33.52, 33.48, 32.93, 32.08, 31.81, 31.41, 30.49, 30.12, 29.98, 29.94, 26.05, 24.95, 24.49, 22.84, 22.11, 21.71, 20.36, 19.90, 19.87, 17.91.

HRMS (ESI+) M+Na$^+$ calculated for $C_{100}H_{172}O_6$: 1493.3080, found: 1493.3072.

Nominal crenarchaeol

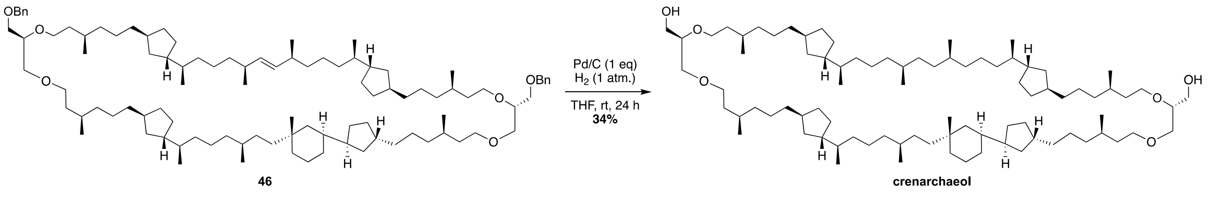

To a flask charged with alkene **46** (3.5 mg, 2.4 µmol, 1 eq.) was added Pd/C (10% Pd by weight, 2.5 mg, 2.4 µmol, 1 eq.). THF (0.25 mL was added) and the suspension was placed under H_2 atmosphere (1 atm., balloon) and stirred for 24 h at rt. The suspension was filtered over Celite, the filtrate was concentrated and the crude was purified by flash chromatography (pentane/EtOAc 4/1) to give the product **nominal crenarchaeol** (1.2 mg, 0.8 µmol, 34%) as colourless oil.

^{1}H NMR (600 MHz, Chloroform-*d*) δ 3.71 (dd, *J* = 11.5, 3.8 Hz, 2H), 3.69 – 3.65 (m, 2H), 3.61 (dd, *J* = 11.4, 5.3 Hz, 2H), 3.53 (ddd, *J* = 14.5, 9.4, 5.0 Hz, 6H), 3.49 – 3.45

(m, 6H), 1.80 – 1.59 (m, 33H), 1.56 – 1.46 (m, 14H), 1.43 – 1.34 (m, 18H), 1.30 – 1.28 (m, 7H), 1.16 – 0.94 (m, 34H), 0.89 – 0.86 (m, 15H), 0.85 (d, *J* = 3.1 Hz, 3H), 0.85 – 0.83 (m, 12H), 0.80 (s, 3H), 0.72 (dd, *J* = 12.4, 4.0 Hz, 1H), 0.64 (t, *J* = 12.7 Hz, 1H).

^{13}C NMR (151 MHz, Chloroform-*d*) δ 78.51, 71.26, 70.28, 70.25, 68.73, 63.23, 45.75, 44.96, 44.86, 43.85, 39.26, 39.23, 39.07, 39.02, 38.44, 38.30, 37.69, 37.58, 37.49, 37.45, 37.40, 37.31, 37.28, 37.19, 36.74, 36.63, 36.16, 36.07, 35.86, 35.79, 34.36, 33.71, 33.53, 33.46, 33.23, 32.93, 31.81, 31.39, 31.32, 30.51, 30.13, 29.98, 29.95, 29.90, 29.86, 26.05, 26.00, 24.54, 24.51, 22.12, 20.32, 20.13, 20.08, 19.93, 19.89, 17.91, 17.88.

HRMS (ESI+) M+Na$^+$ calculated for $C_{86}H_{162}O_6$: 1292.2444, found: 1292.2302.

NMR analysis by chemical shift comparison of Fragment B, nominal, and natural crenarchaeol

Isolation of natural crenarchaeol

Crenarchaeol was isolated from sea surface sediment samples and purified by preparative HPLC according to a previously reported procedure.[20] The purified isolated crenarchaeol was analyzed by a 600 MHz NMR spectrometer.

NMR analysis of Fragment B

<u>Full range HSQC of Fragment B</u>

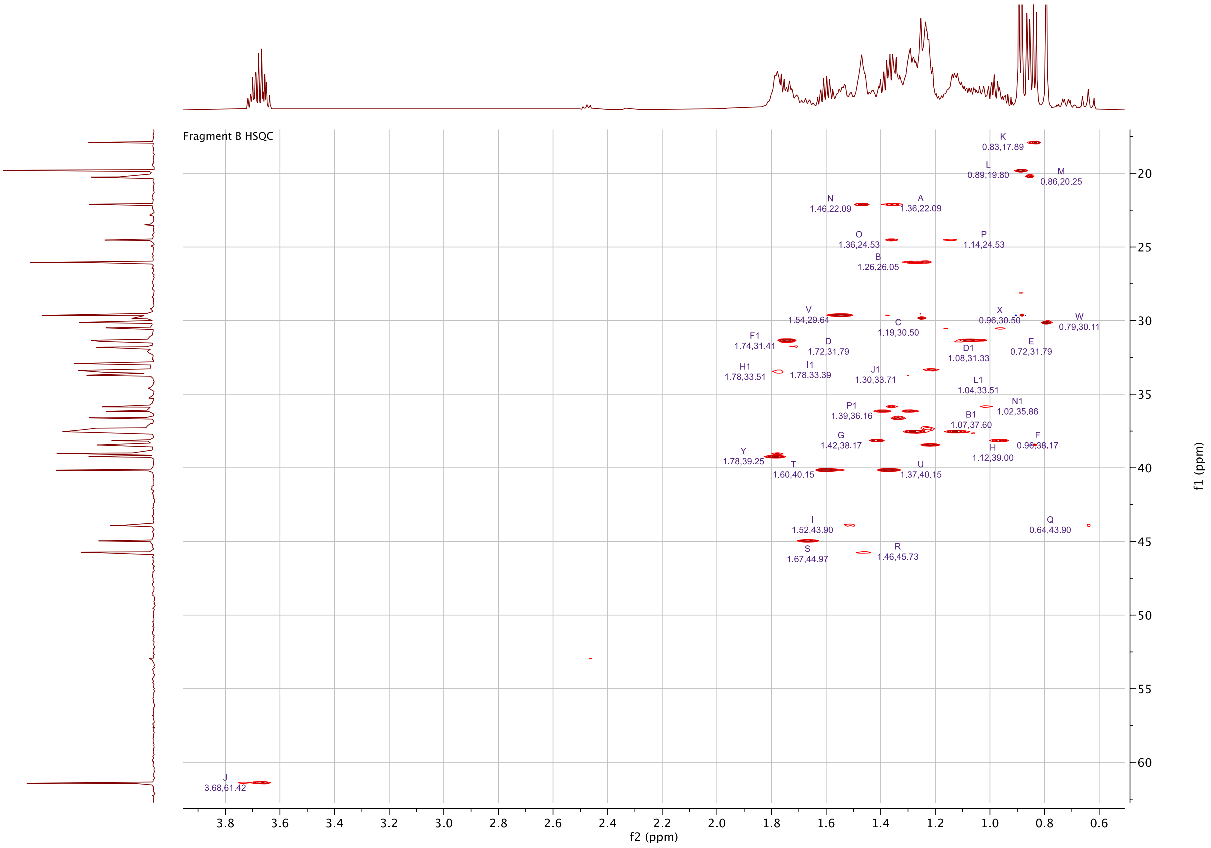

HSQC of Fragment B at different ranges

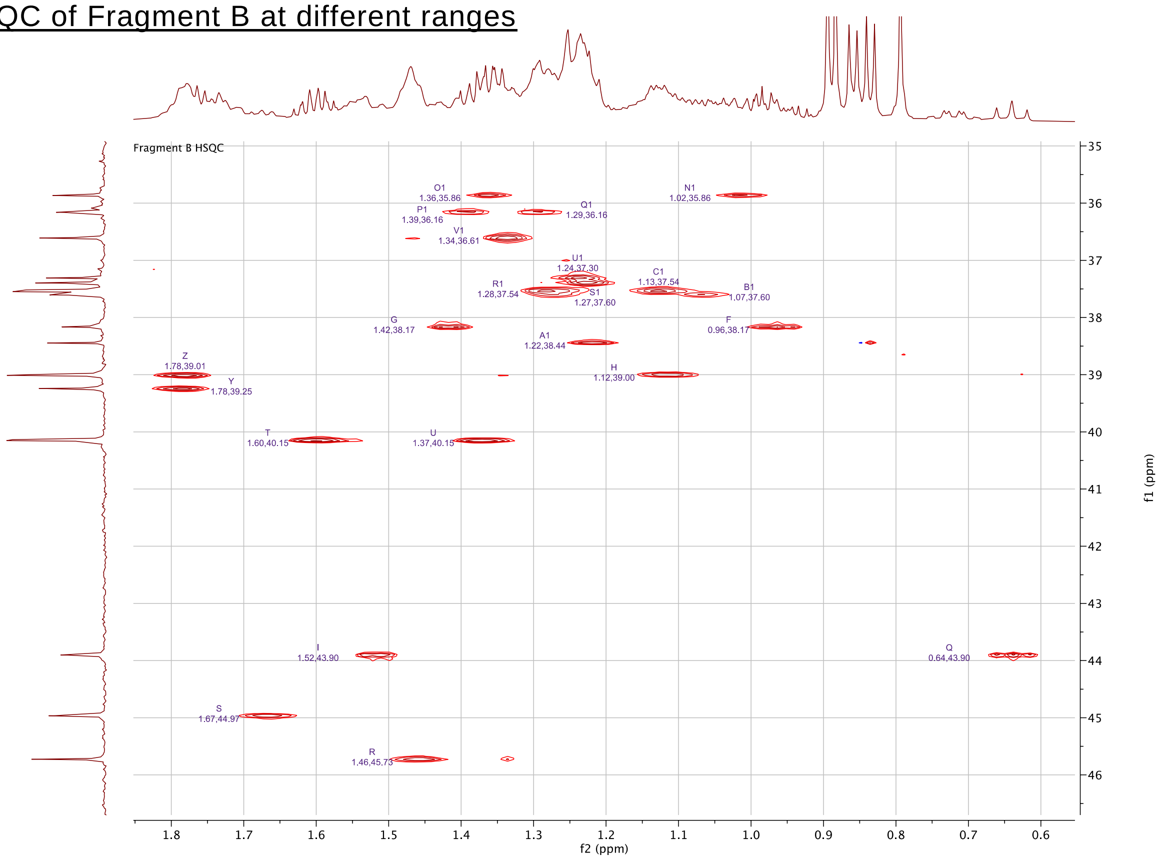

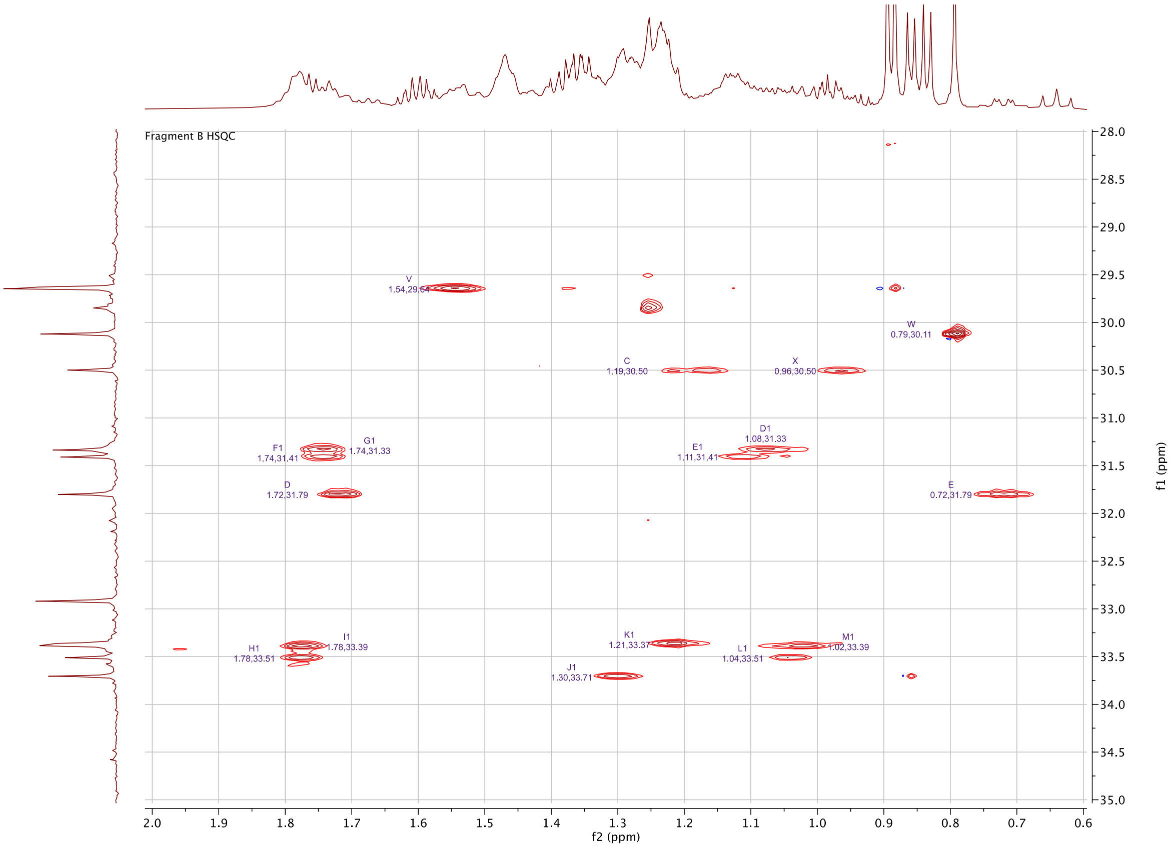

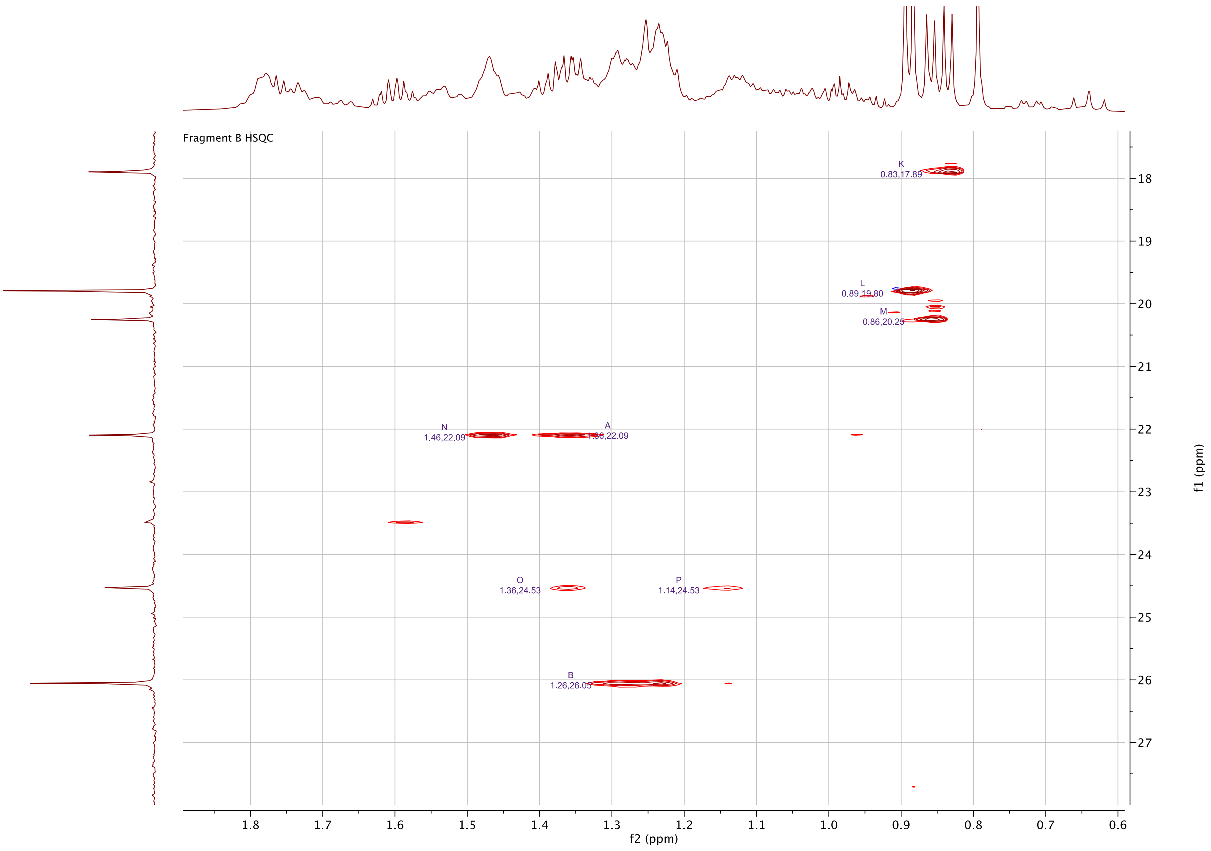

Fragment B HSQC
K
0.83,17.89
L
0.89,19.80
M
0.86,20.25
N
1.46,22.09
A
O
1.36,24.53
P
1.14,24.53
B
1.26,26.05
f1 (ppm)
f2 (ppm)

Fragment B COSY NMR analysis

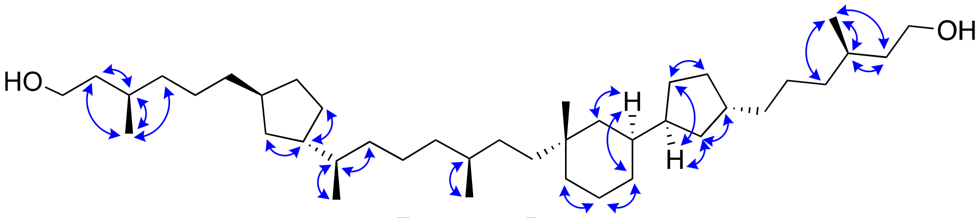

Fragment B

2D COSY correlations

Full range COSY of Fragment B

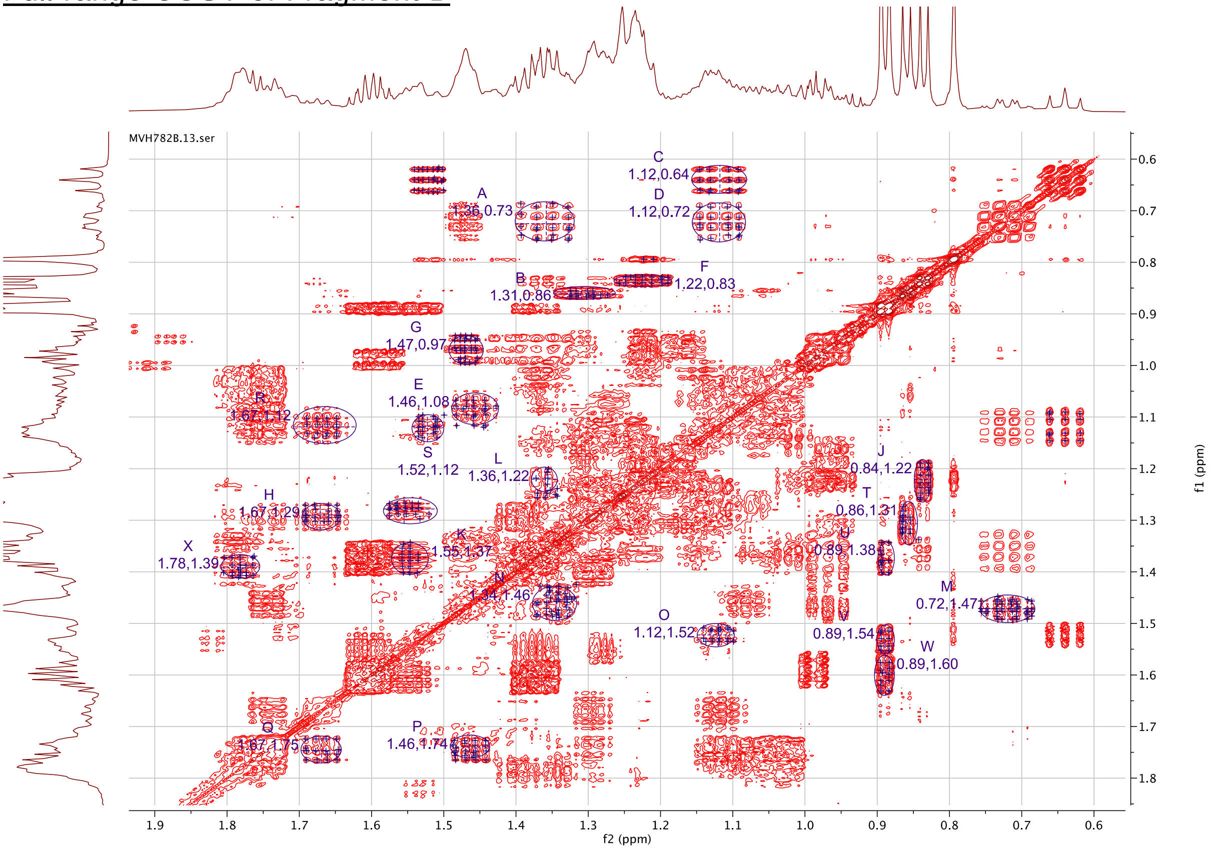

Fragment B HMBC analysis

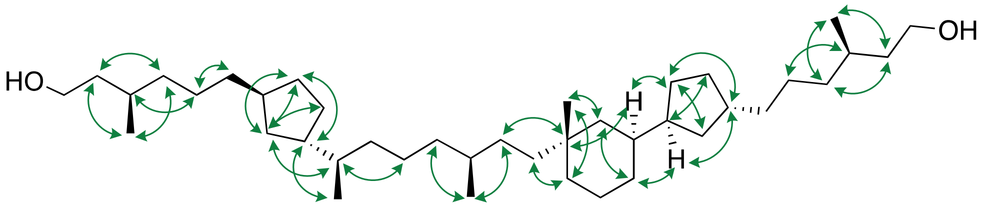

Fragment B

2D HMBC correlations

Full range HMBC of Fragment B

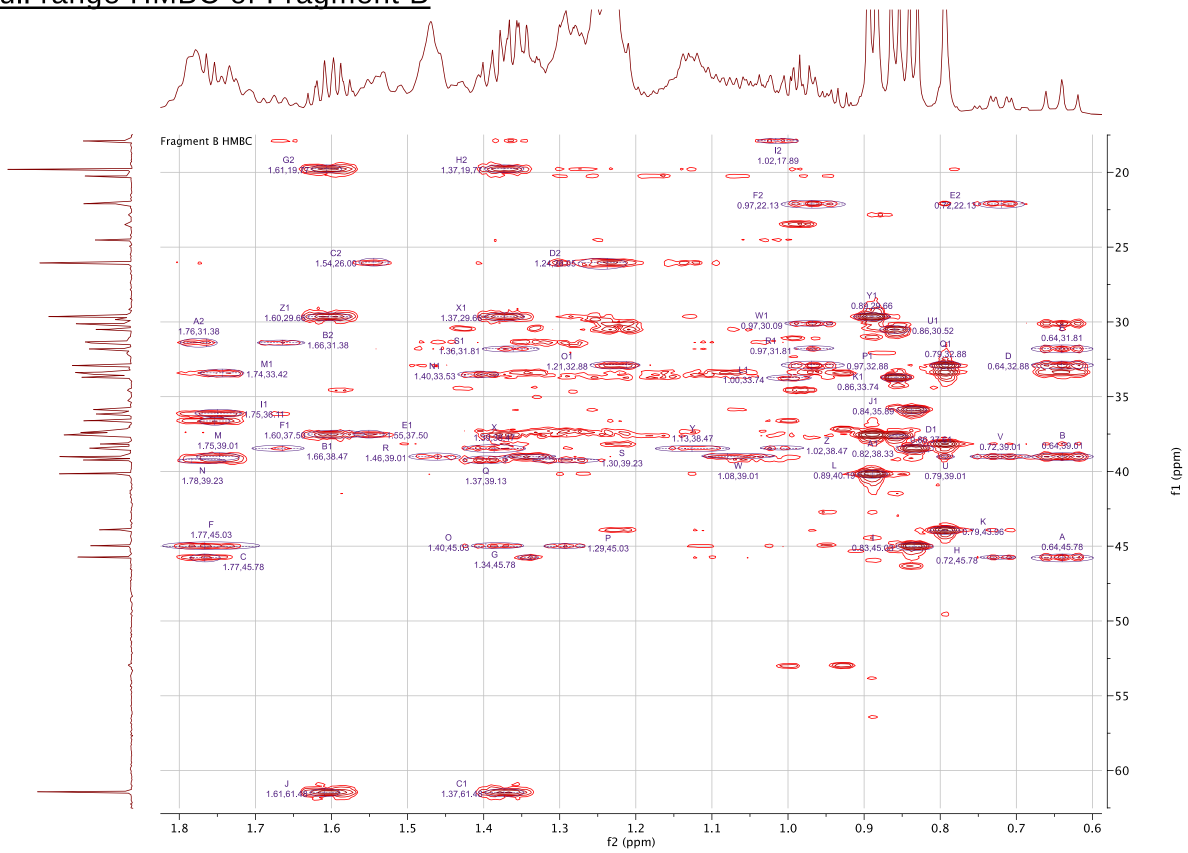

HMBC of Fragment B (zoomed)

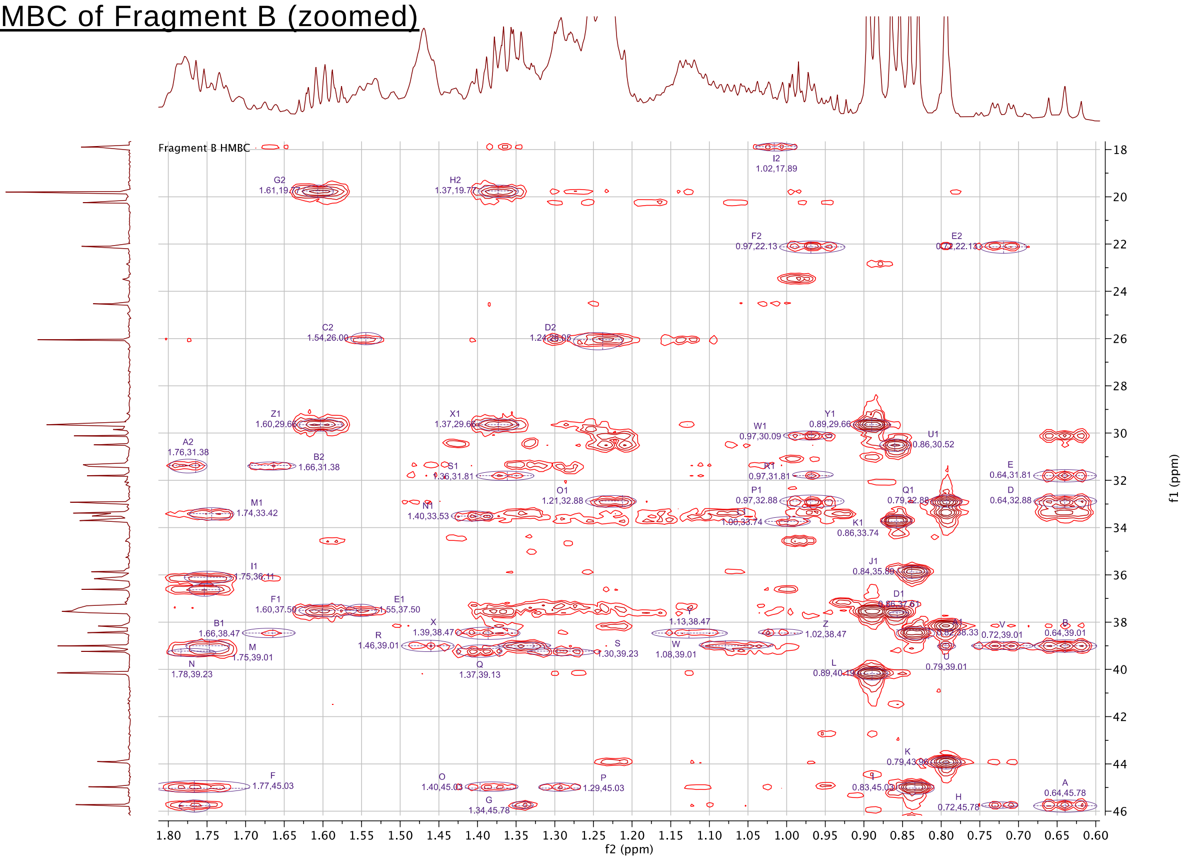

NMR analysis of synthetic nominal crenarchaeol

Full range HSQC of synthetic nominal crenarchaeol

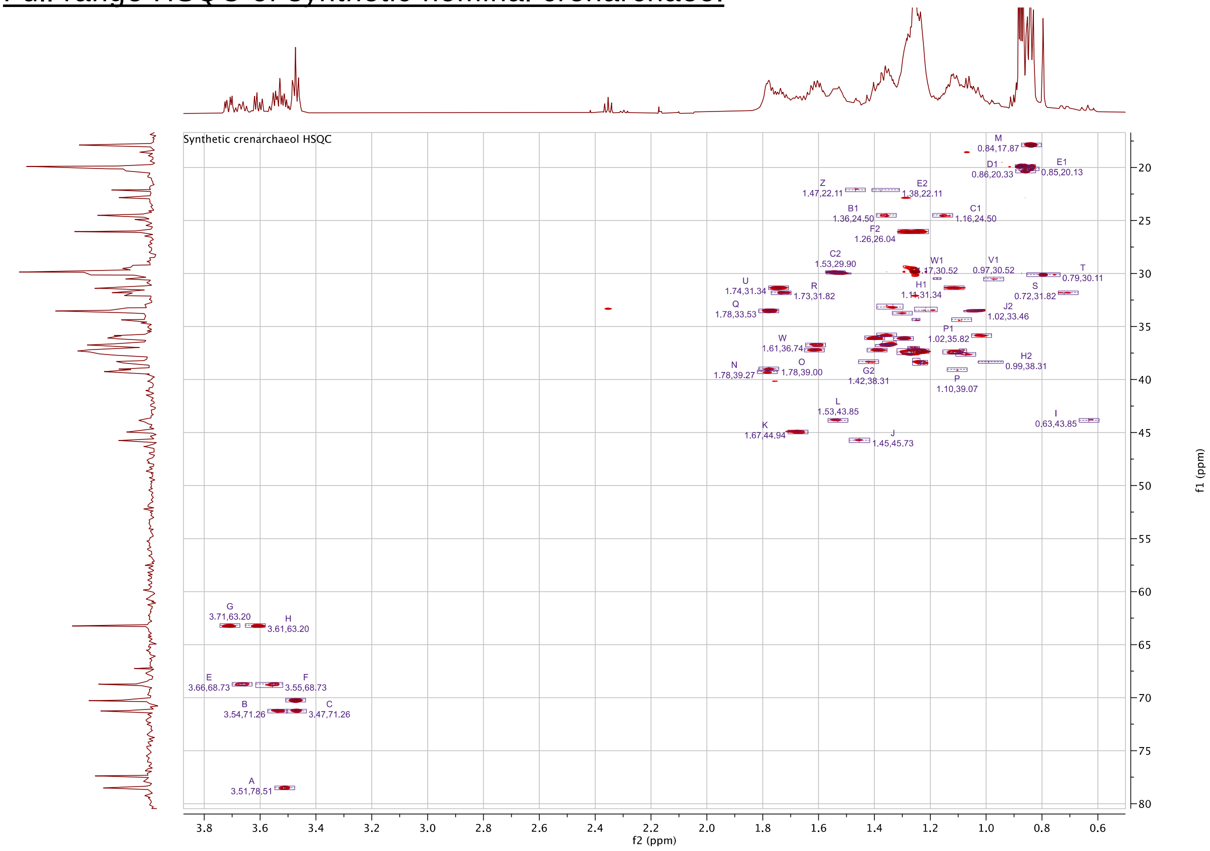

HSQC of synthetic nominal crenarchaeol at different ranges

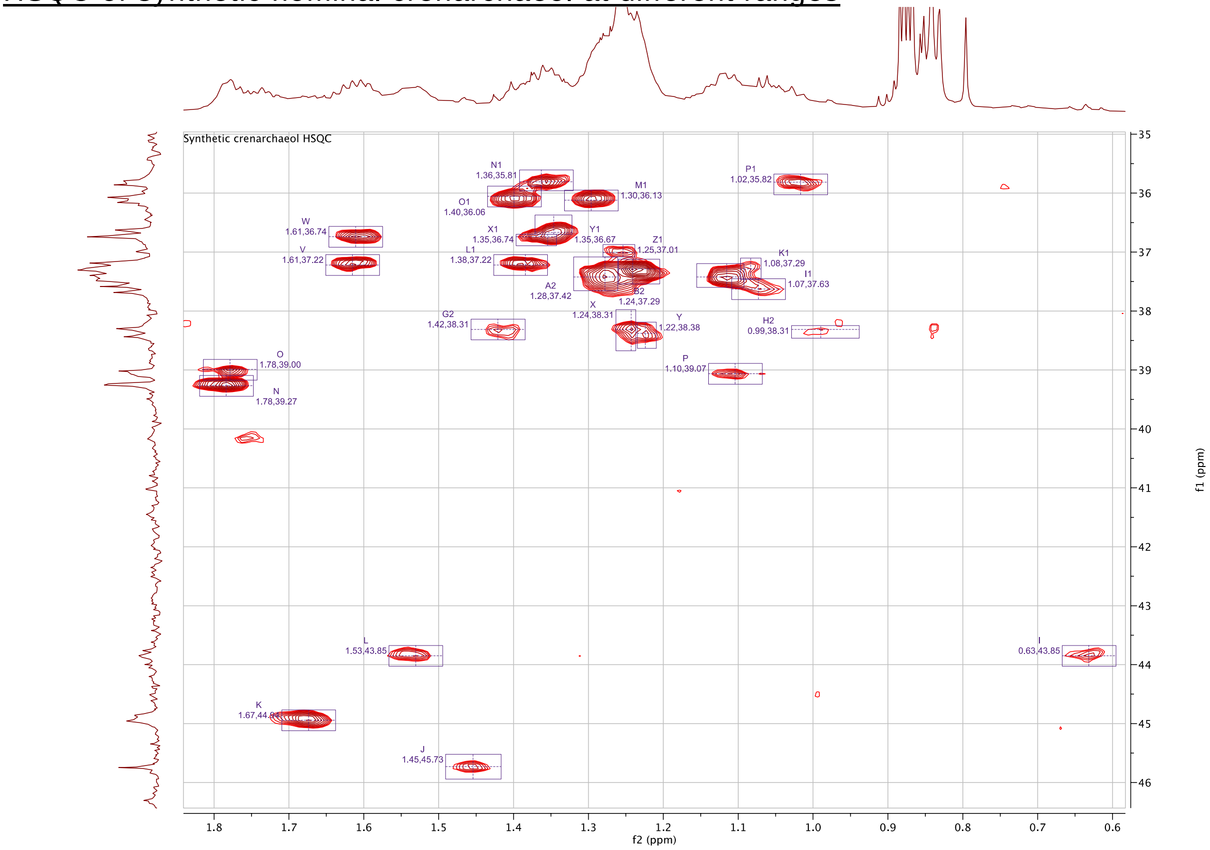

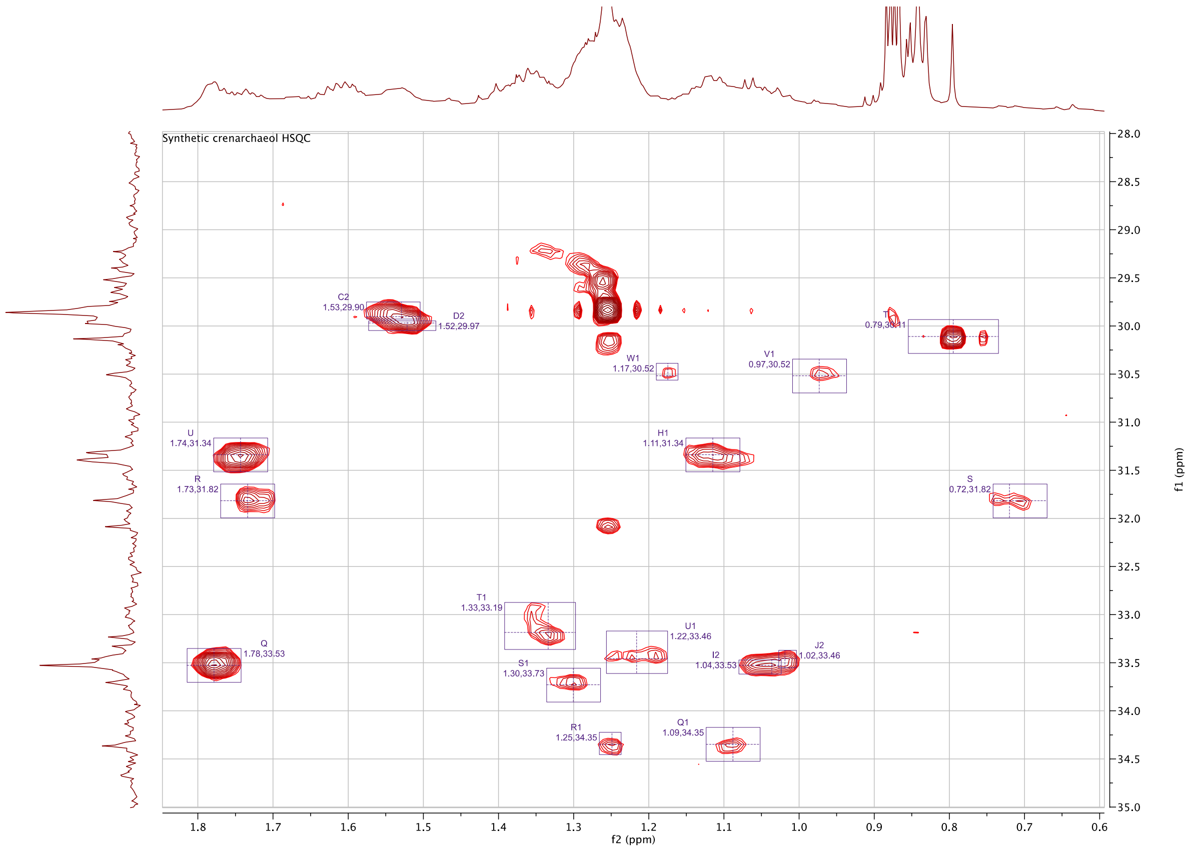
Synthetic crenarchaeol HSQC
C2
1.53,29.90
D2
1.52,29.97
W1
1.17,30.52
V1
0.97,30.52
U
1.74,31.34
H1
1.11,31.34
R
1.73,31.82
S
0.72,31.82
T1
1.33,33.19
U1
1.22,33.46
Q
1.78,33.53
I2
1.04,33.53
J2
1.02,33.46
S1
1.30,33.73
R1
1.25,34.35
Q1
1.09,34.35
f2 (ppm)
f1 (ppm)

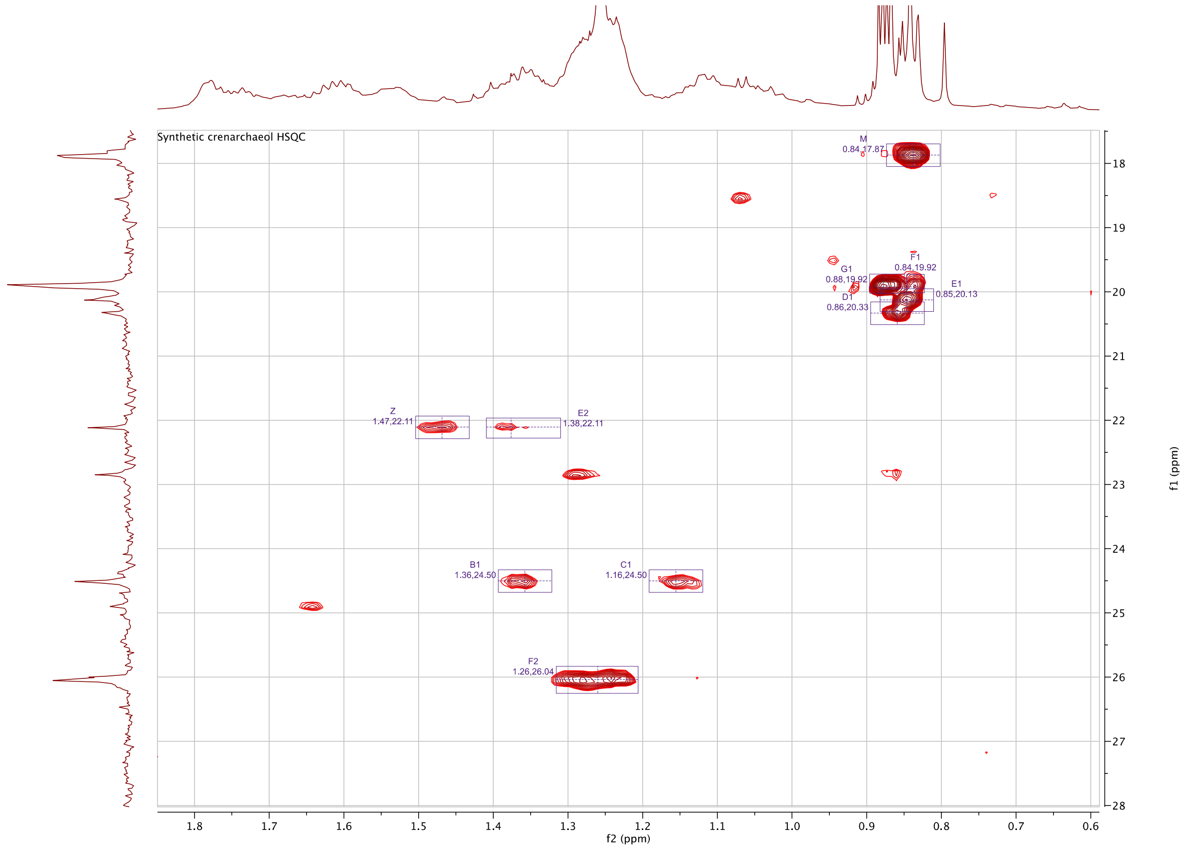
Synthetic crenarchaeol HSQC
M
0.84,17.87
F1
0.84,19.92
G1
0.88,19.92
E1
0.85,20.13
D1
0.86,20.33
Z
1.47,22.11
E2
1.38,22.11
B1
1.36,24.50
C1
1.16,24.50
F2
1.26,26.04
f2 (ppm)
f1 (ppm)

NMR analysis of natural isolated crenarchaeol

^{1}H NMR of natural crenarchaeol

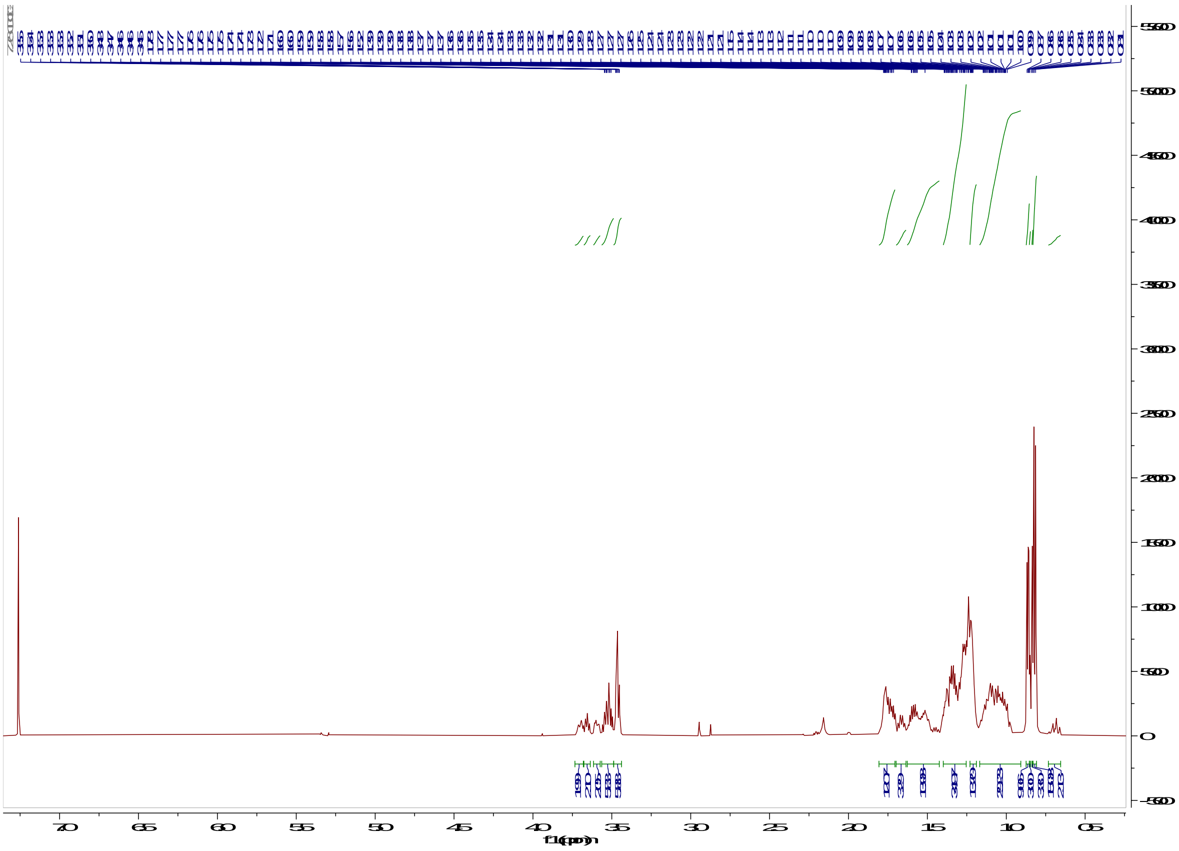

Full range HSQC of natural crenarchaeol

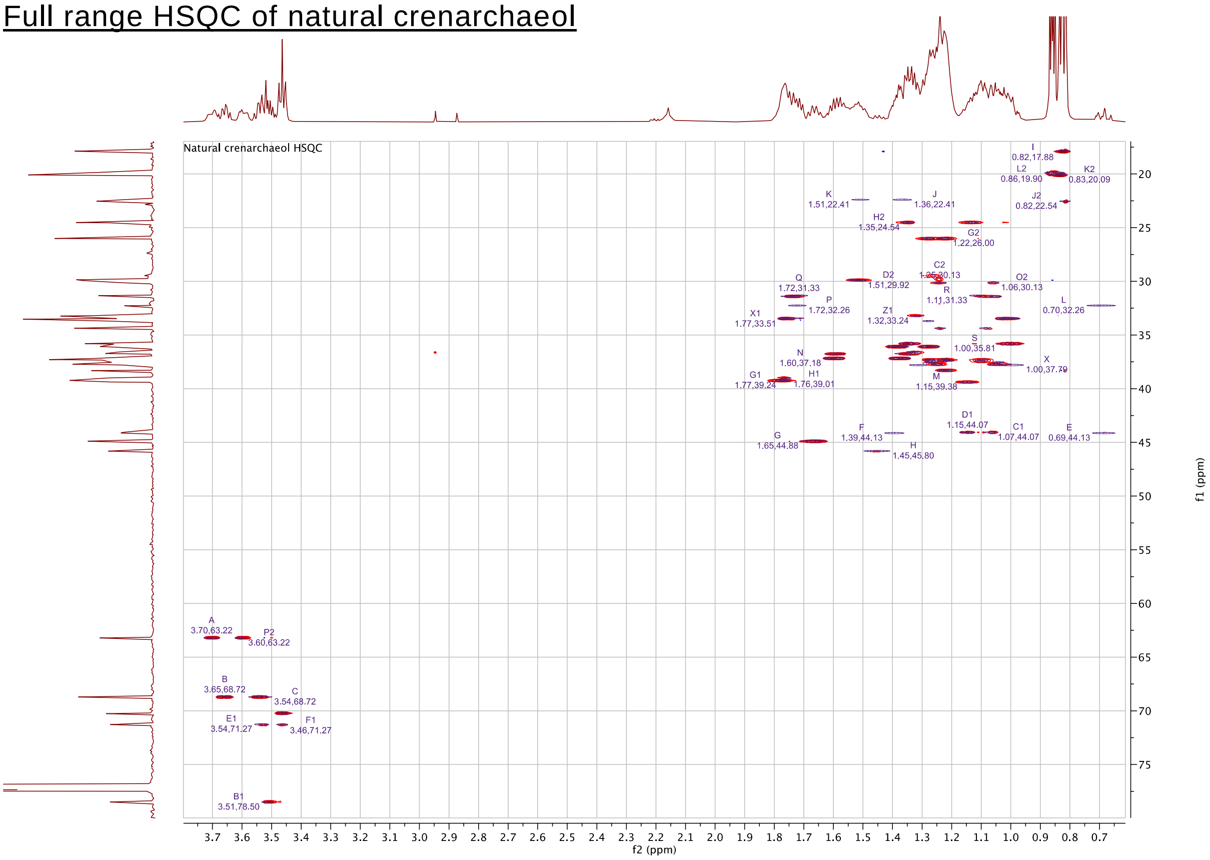

HSQC of natural crenarchaeol at different ranges

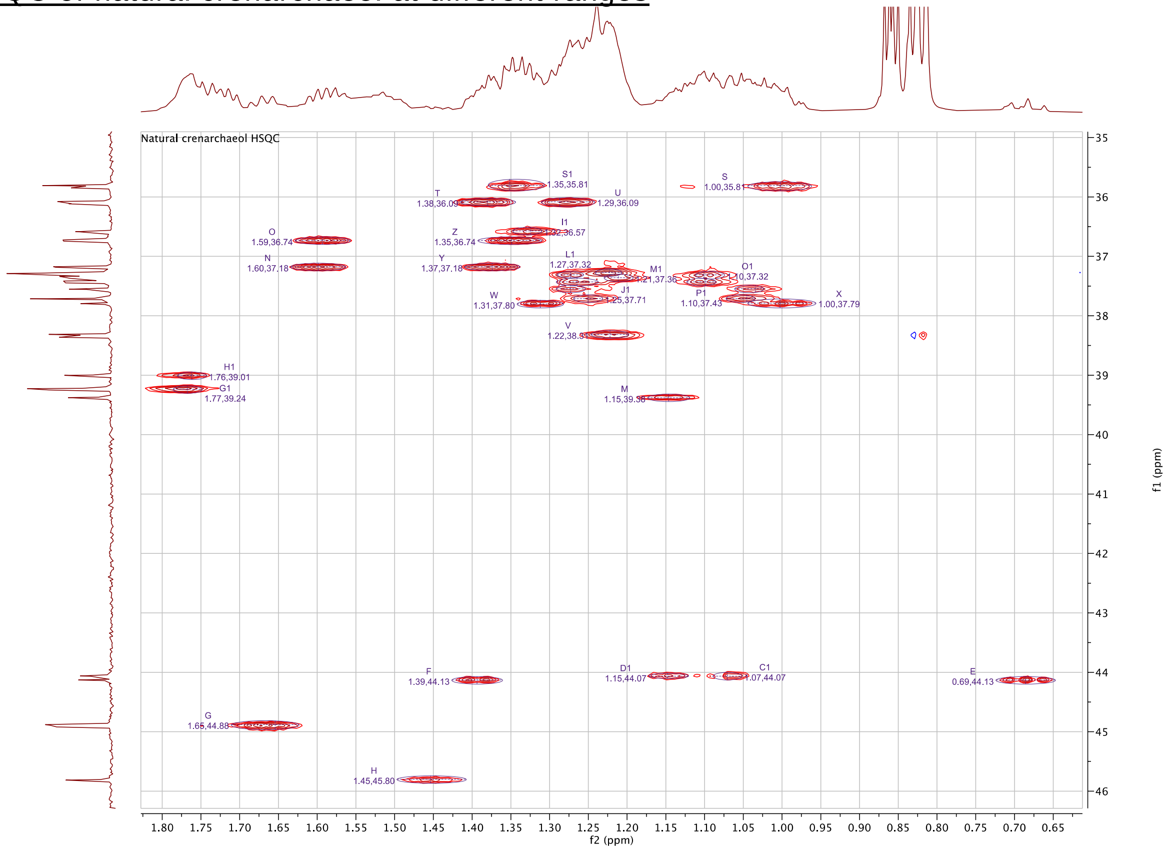

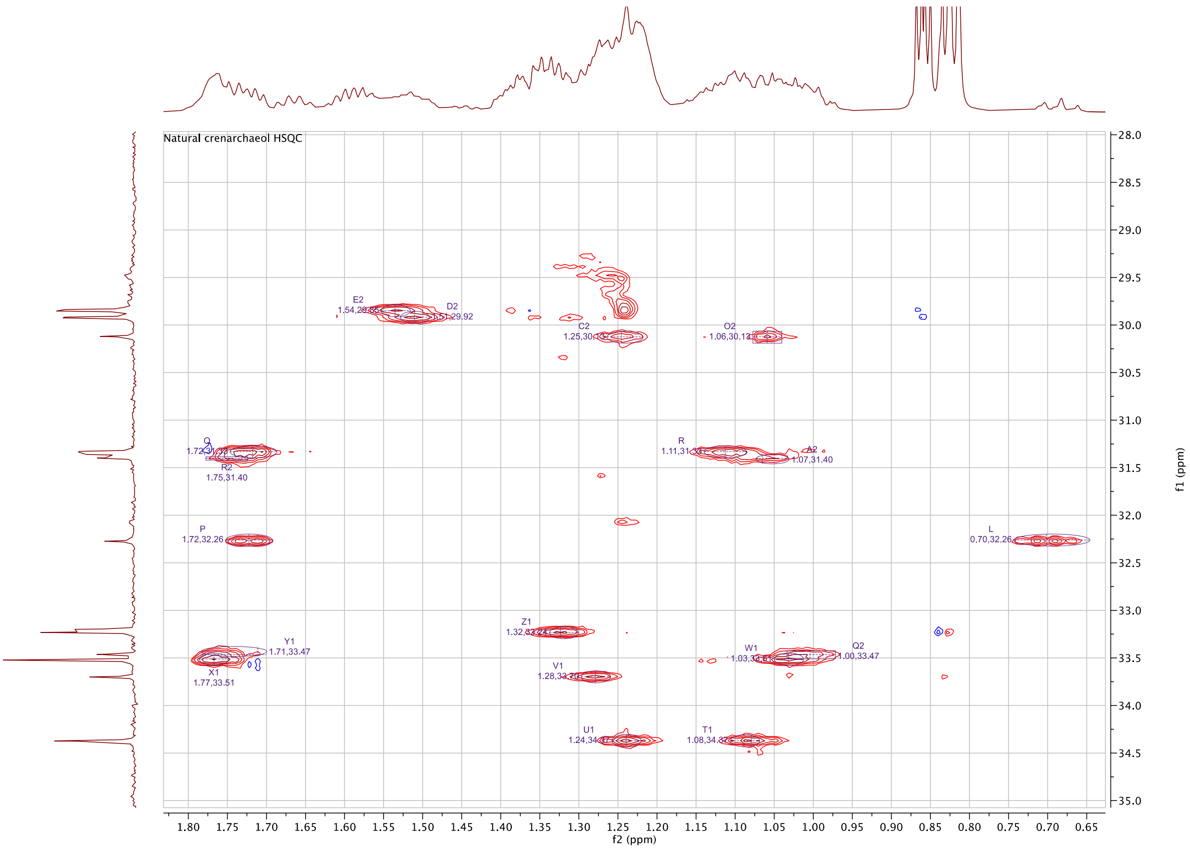
Natural crenarchaeol HSQC
E2
D2
C2
O2
R
R2
1.75,31.40
A2
1.07,31.40
P
1.72,32.26
L
0.70,32.26
Z1
Y1
1.71,33.47
X1
1.77,33.51
V1
W1
Q2
1.00,33.47
U1
T1
f2 (ppm)
f1 (ppm)

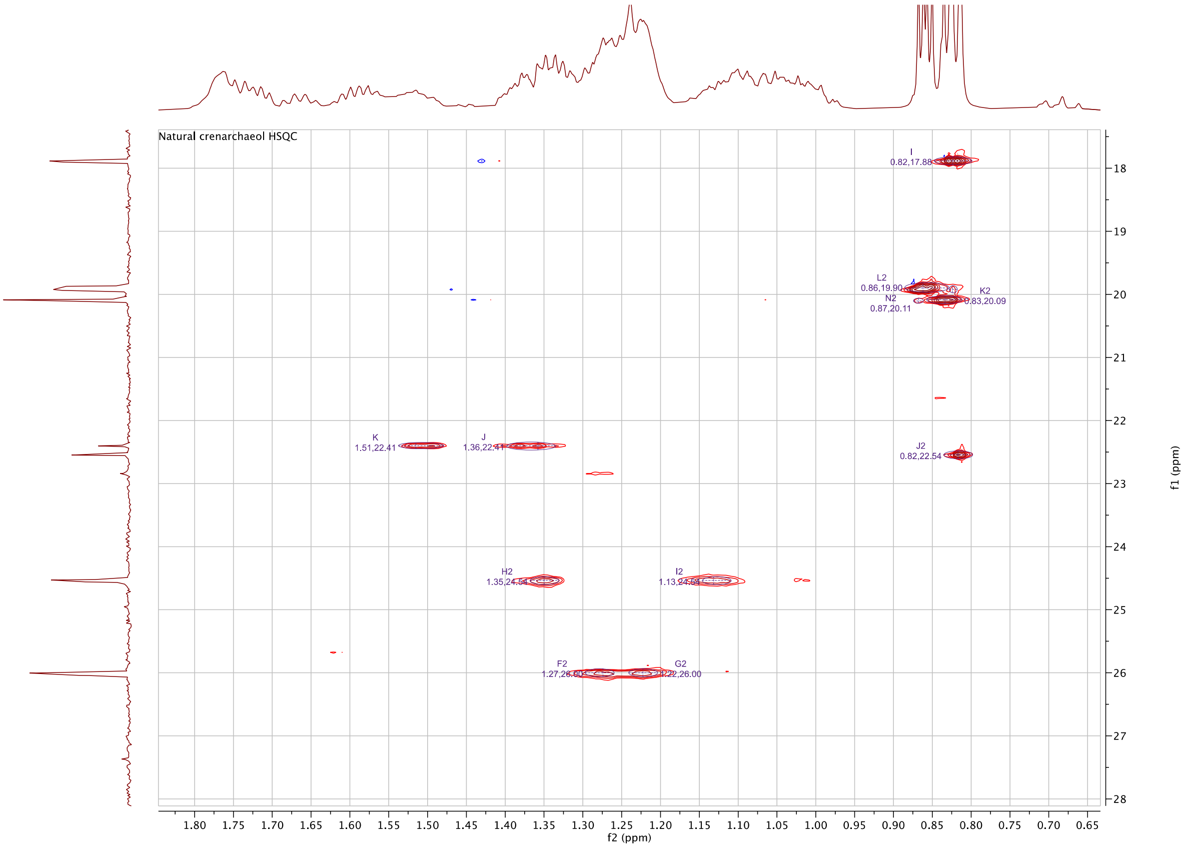
Natural crenarchaeol HSQC
I
0.82,17.88
L2
0.86,19.90
N2
0.87,20.11
K2
K
1.51,22.41
J
J2
0.82,22.54
H2
I2
F2
G2
f2 (ppm)
f1 (ppm)

DEPT of natural crenarchaeol

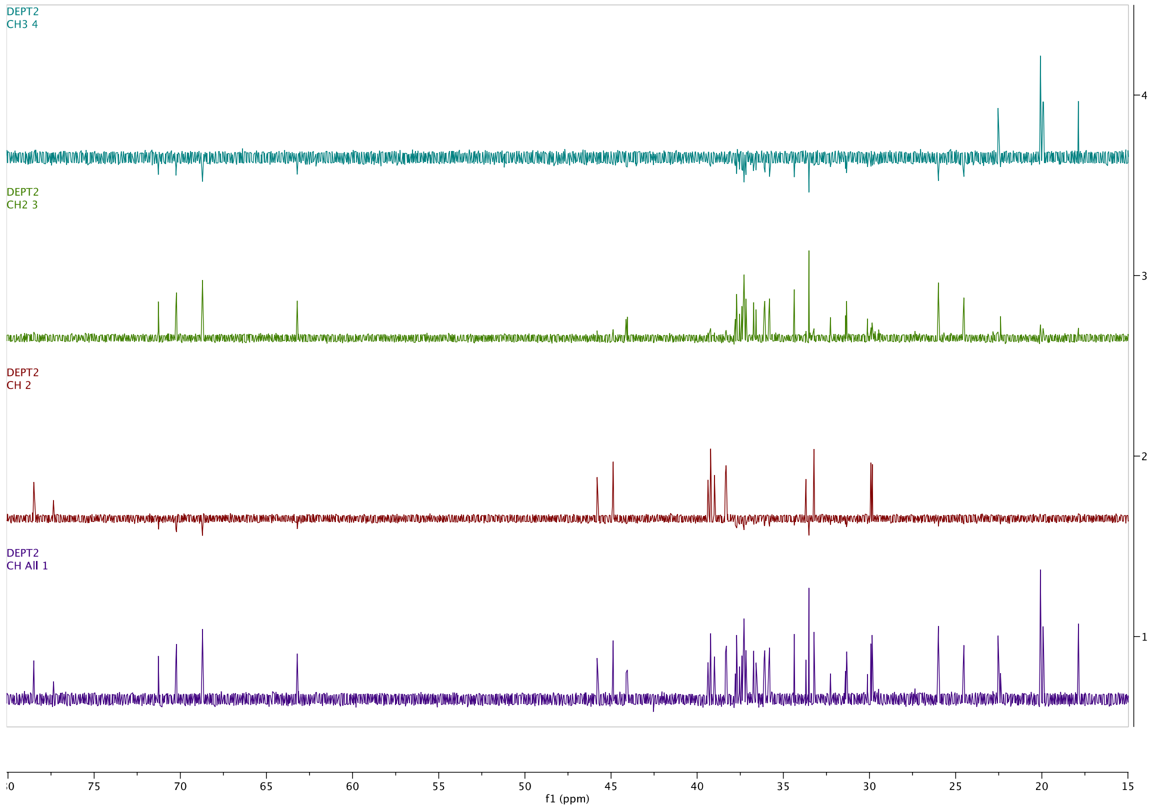

^{13}C NMR of natural crenarchaeol

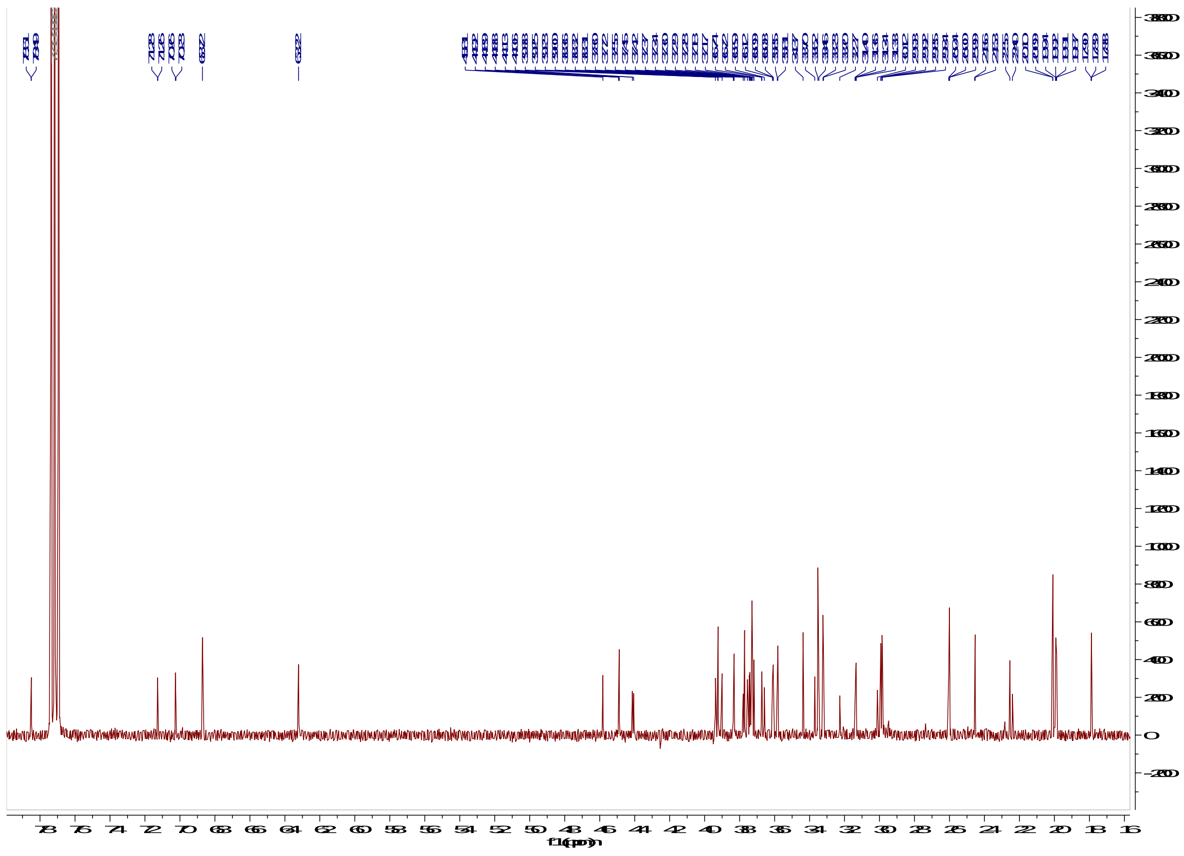

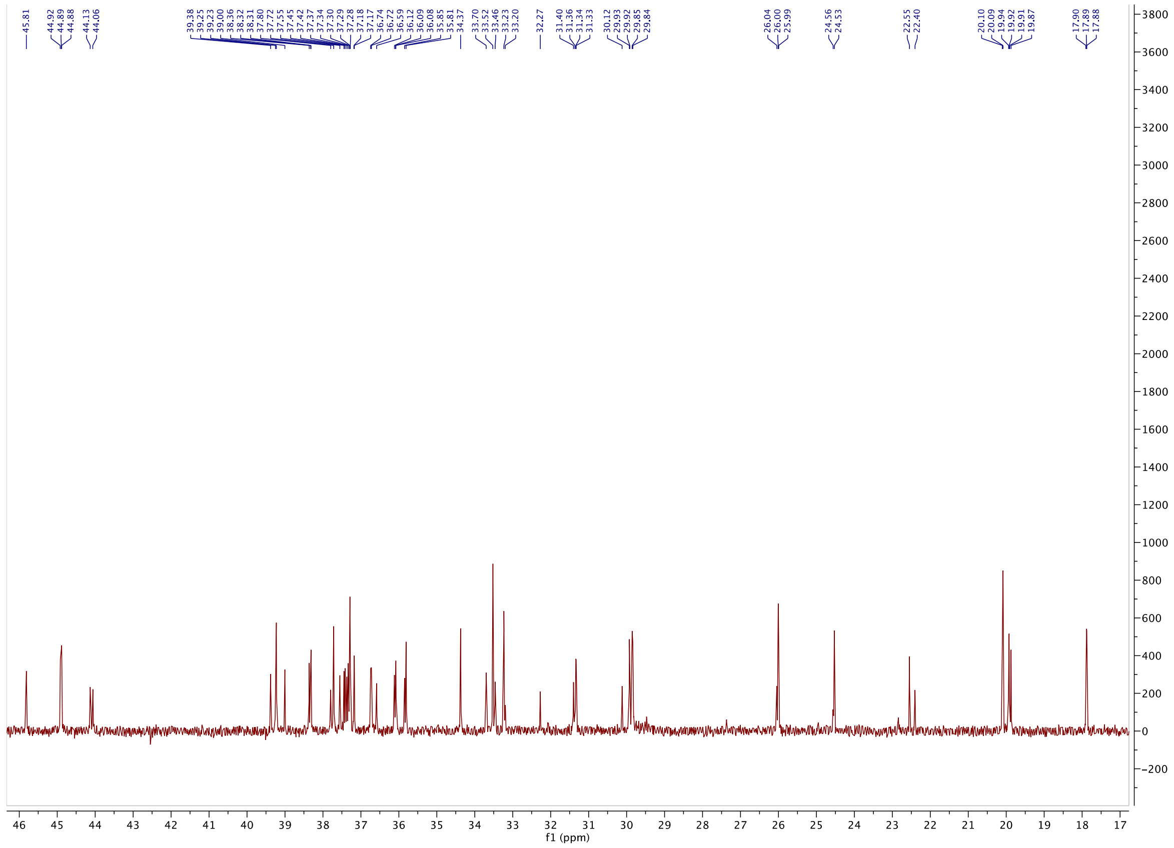

^{1}H and ^{13}C NMR chemical shift comparison of Fragment B, natural, and synthetic nominal crenarchaeol

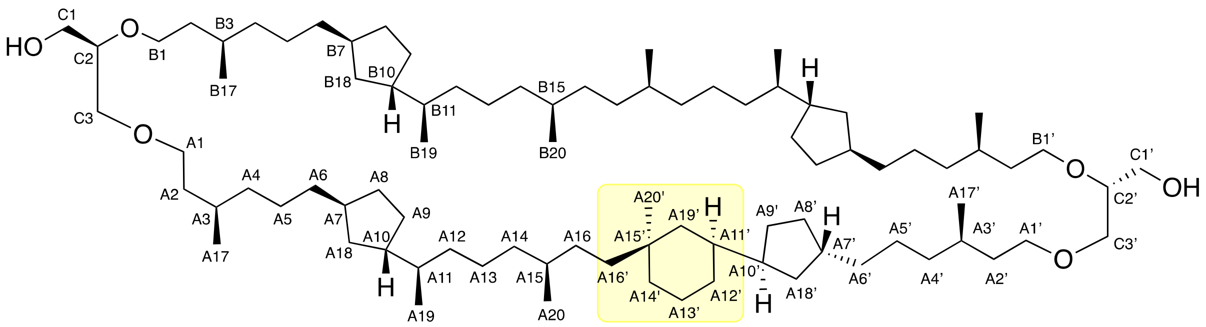

crenarchaeol
(revised structure)

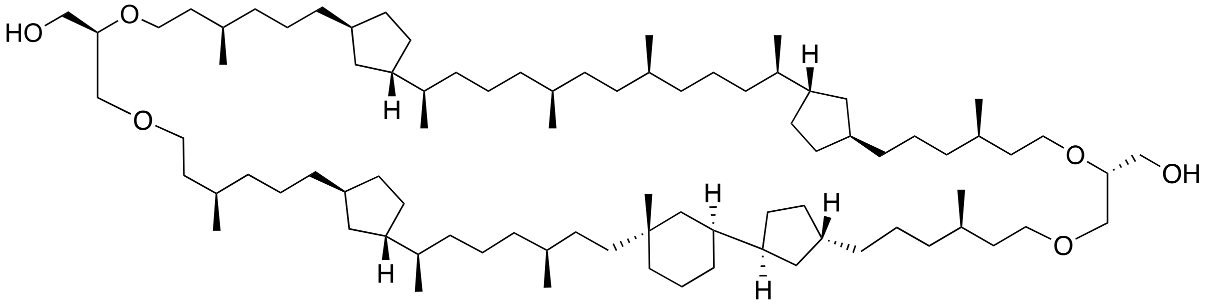

synthetic crenarchaeol
(originally proposed structure)

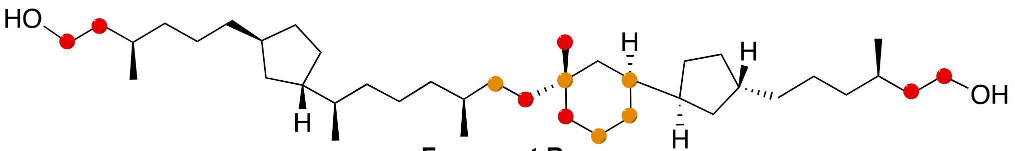

Fragment B

Numbering of the bicyclic biphytane chain by analogy (B1/B1' to B20/B20')[20]

<u>Notes to table below:</u>

^{1}H and ^{13}C NMR of Fragment B, natural and synthetic crenarchaeol were recorded on a 600 MHz NMR spectrometer. Signals are reported in ppm relative to the solvent residual signals ($CDCl_3$, ^{1}H δ 7.26 ppm, ^{13}C δ 77.16 ppm). Moderate ^{13}C chemical shift differences ($\Delta\delta$ 0.25–1 ppm) of Fragment B and synthetic crenarchaeol relative to natural crenarchaeol are highlighted in orange. Larger ^{13}C chemical shift differences ($\Delta\delta$ >1 ppm) are highlighted in red. Assignments of the signals in natural crenarchaeol were done as reported previously.[20]

Carbon Number	^{13}C shift natural crenarchaeol (ppm)	^{1}H shift natural crenarchaeol (ppm)	^{13}C shift nominal synthetic crenarchaeol (ppm)	^{1}H shift nominal synthetic crenarchaeol (ppm)	^{13}C shift Fragment B (ppm)	^{1}H shift Fragment B (ppm)
**A1, B1’**	70.26, 70.23	3.46	70.28, 70.25	3.47	61.42	3.68
**A1’, B1**	68.72	3.65, 3.54	68.73	3.66, 3.55	61.42	3.68
**A2, B2’**	36.74, 36.72	1.59, 1.35	36.74	1.61, 1.35	40.15	1.60, 1.37
**A2’, B2**	37.18, 37.17	1.59, 1.38	37.19	1.61, 1.38	40.15	1.60, 1.37
**A3, A3’**	29.85, 29.84	1.54	29.90, 29.86	1.53	29.65, 29.63	1.54
**B3, B3’**	29.93, 29.92	1.51	29.98, 29.95	1.52		
**A4, A4’, B4, B4’**	37.37, 37.34, 37.45, 37.42	1.27, 1.26, 1.21, 1.10	37.49, 37.45, 37.40, 37.31	1.28, 1.24, 1.12, 1.08, 1.07	37.55, 37.52	1.28, 1.13
**A5, A5’ B5, B5’**	26.00, 25.99	1.27, 1.22	26.05, 26.00	1.26	26.05	1.26
**A6, A6’ B6, B6’**	37.28, 37.29	1.22	37.28	1.25	37.40, 37.31	1.24, 1.23
**A7, B7**	39.25	1.77	39.26, 39.23	1.78	39.24	1.78
**A7’, B7’**	39.00	1.76	39.02	1.78	39.00	1.78
**A8, B8, B8’**	33.52	eq.: 1.77, ax.: 1.03	33.53	eq.: 1.78, ax.: 1.04	33.39	eq.: 1.78, ax.: 1.02
**A8’**	33.46	eq.: 1.71, ax.: 1.01	33.46	eq.: 1.78, ax.: 1.02	33.36	eq.: 1.78, ax.: 1.21
**A9, A9’**	31.40, 31.36	eq.: 1.75, ax.: 1.07	31.39	eq.: 1.74, ax.: 1.11	31.41, 31.34	eq.: 1.74, ax.: 1.11, 1.08
**B9, B9’**	31.34, 31.33	eq.: 1.72, ax.: 1.11	31.32	eq.: 1.74, ax.: 1.11	–	–
**A10**	44.92	1.67	44.96	1.67	44.96	1.67
**A10’**	45.81	1.45	45.75	1.45	45.73	1.46
**B10, B10’**	44.88, 44.89	1.67	44.86	1.67	–	–
**A11**	38.36	1.22	38.30	1.24	38.45	1.22
**A11’**	39.38	1.15	39.07	1.10	39.01	1.12
**B11, B11’**	38.31, 38.32	1.22	38.44	1.22	–	–
**A12**	35.85	1.35, 1.00	35.86	1.37, 1.02	35.87	1.36, 1.02
**A12’**	32.27	eq.: 1.72, ax.: 0.70	31.81	eq.: 1.73, ax.: 0.72	31.80	eq.: 1.72, ax.: 0.72
**B12, B12’**	35.81	1.35, 1.00	35.79	1.37, 1.02	–	–
**A13**	24.56	1.36, 1.13	24.54, 24.51	1.36, 1.16	24.53	1.36, 1.14
**A13’**	22.40	eq.: 1.51, ax.: 1.35	22.12	eq.: 1.47, ax.: 1.38	22.10	eq.: 1.46, ax.: 1.36
**B13, B13’**	24.53	1.36, 1.13	24.54, 24.51	1.36, 1.16	–	–
**A14**	37.55	1.27, 1.03	37.59	1.28, 1.07	37.60	1.27, 1.07
**A14’**	44.06	1.15, 1.07	38.30	1.42, 0.99	38.16	eq.: 1.42, 0.96
**B14, B14’**	37.72	1.25, 1.05	37.69	1.28, 1.07	–	–
**A15**	33.70	1.28	33.71	1.30	33.71	1.30
**A15’**	33.20	–	32.93	–	32.92	–
**B15, B15’**	33.23	1.32	33.23	1.33	–	–
**A16**	30.12	1.25, 1.06	30.51	1.17, 0.97	30.50	1.19, 0.96
**A16’**	37.80	1.31, 1.00	33.46	1.78, 1.04	33.51	1.78, 1.04
**B16, B16’**	34.37	1.24, 1.08	34.36	1.25	–	–
**A17’, B17**	19.90	0.86	19.89	0.88	19.79	0.89
**A17, B17’**	19.92	0.82	19.93	0.84	19.79	0.89
**A18**	36.12	1.38, 1.29	36.16	1.40, 1.30	36.16	1.39, 1.29
**A18’**	36.59	1.32	36.63	1.35	36.61	1.34
**B18, B18’**	36.09, 36.08	1.38, 1.29	36.07	1.40, 1.30	–	–
**A19’**	44.13	eq.: 1.39, ax.: 0.69	43.85	eq.: 1.53, ax.: 0.63	43.90	eq.: 1.52, ax.:0.64
**A19, B19, B19’**	17.88	0.82	17.91, 17.88	0.84	17.90	0.83
**A20**	20.10	0.87	20.32	0.86	20.25	0.86
**A20’**	22.55	0.82	30.13	0.79	30.12	0.79
**B20, B20’**	20.09	0.83	20.13, 20.08	0.85	–	–
**C1, C1’**	63.22	3.70, 3.60	63.23	3.71, 3.61	–	–
**C2, C2’**	78.51, 78.49	3.51	78.51	3.51	–	–
**C3, C3’**	71.28, 71.26	3.54, 3.46	71.26	3.54, 3.47	–	–

Chemical derivatization and GC-MS analysis

Ether cleavage and conversion to hydrocarbons

Isolation and derivatization procedures were reported previously.[21,22] Briefly,an aliquot of Fragment B was subjected to ether cleavage (57% HI) and the resulting primary alkyl iodide was then converted to the corresponding hydrocarbon using H_2/PtO_2.[22]

GC-MS analysis of biphytanes

The biphytanes were analyzed by GC (on-column injection) using an Agilent 7890B GC instrument as well as in splitless mode with GC–MS on an Agilent 7890A GC instrument equipped with an Agilent 5975C VL MSD detector operated at 70 eV. A CP Sil 5CB column (25 m x 0.32 mm; film thickness 0.12 µm; He carrier gas) was used to separate the biphytanes. Samples were injected at 70 °C and the GC oven was programmed to heat to 130°C (at 20 °C min^{-1}) and further heated to 320 °C (4 °C min^{-1}) temperature, which was held for 10 min.

Full range gas-chromatograms

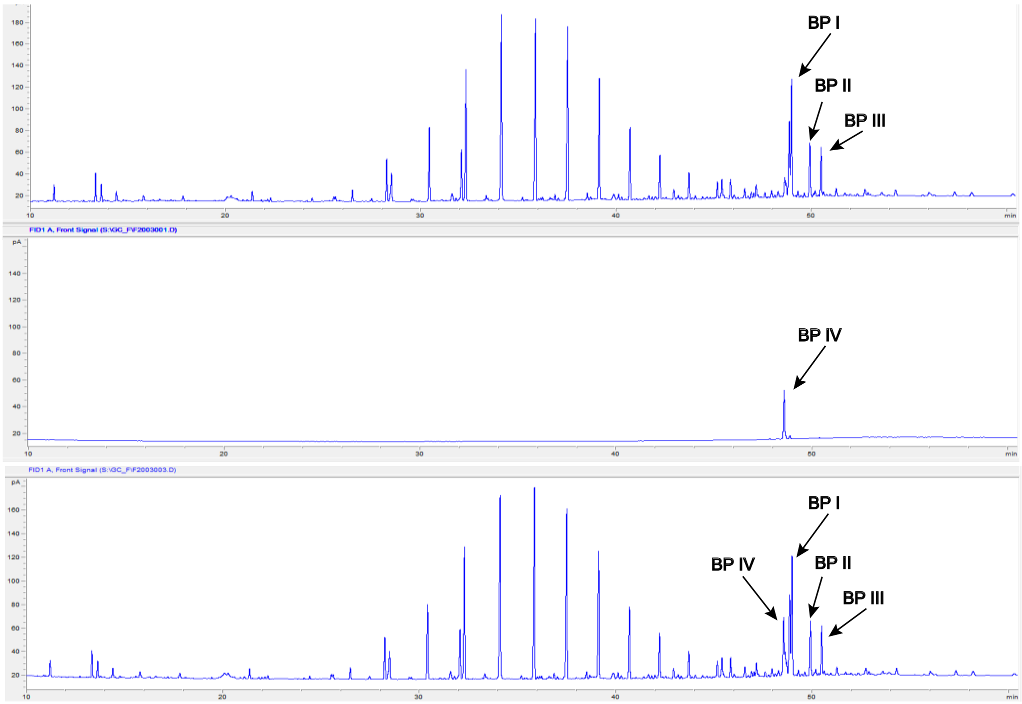

Upper trace: injection of derivatization products of the GDGTs released from the Bligh Dryer extract of the thermophilic Thaumarchaeota “*Ca. Nitrosotenuis uzonensis*”.
Middle trace: injection of derivatization product of Fragment B
Lower trace: Co-injection of derivatization products.

GC-MS spectra of derivatization products BPI-IV

BP I

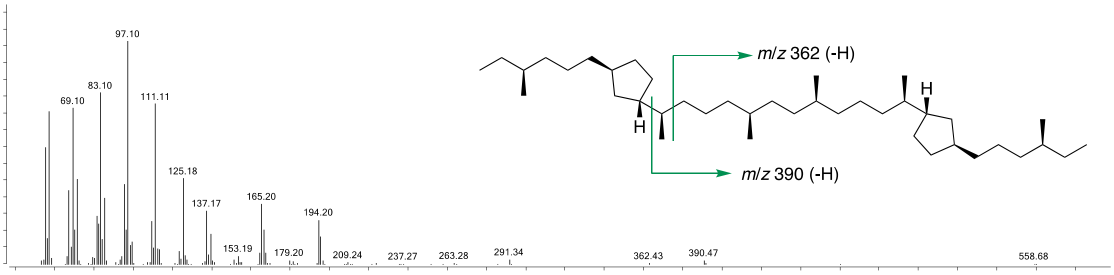

BP II

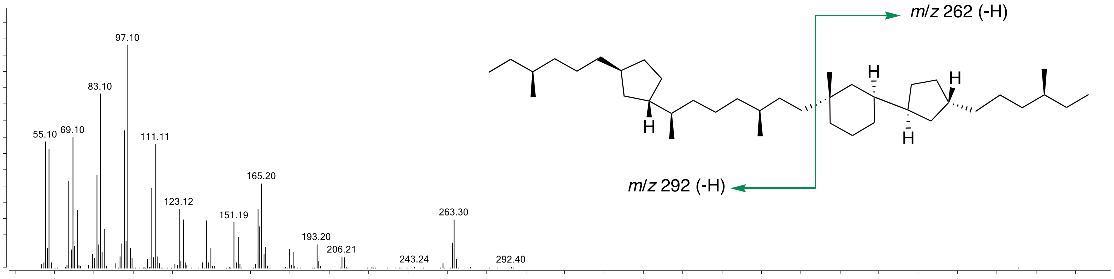

BP III

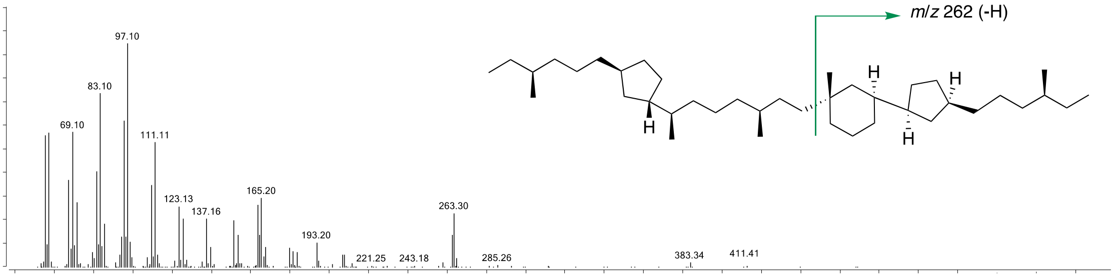

BP IV

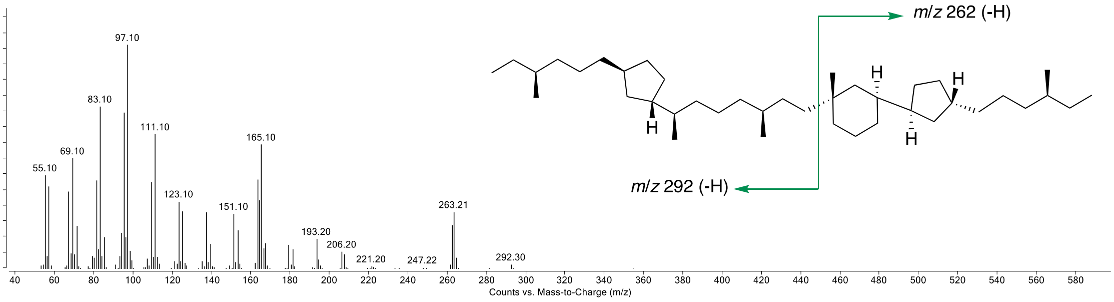

GC-MS spectra of derivatization product BPI and the early eluting isomer of BPI (annotated with * in the manuscript Fig. 2B)

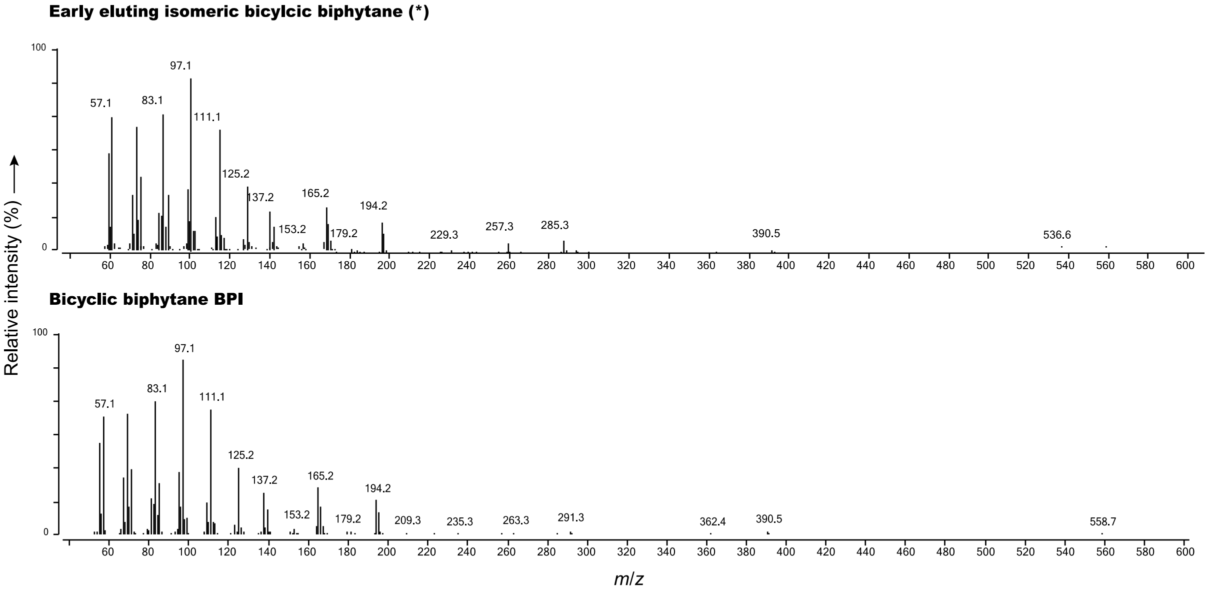

Computational prediction of ^{13}C chemical shift values in natural and nominal crenarchaeol

To search the conformational space for low energy conformations, molecular dynamics (MD) simulations of both isomers were performed in chloroform in a box with initial dimensions of 8 × 8 × 8 nm. The MD simulations were performed with the GROMACS suite 2020.1[23] using the OPLS-AA[24] force field. The system was equilibrated during 5 ns using the NVT and NPT ensembles, respectively. The production run of 10 ns at 298.15 K was performed at constant pressure, in which the temperature was kept constant using the V-rescale,[25] and the pressure with the Parrinello-Rahman barostat.[26] The coordinates were written every 10 ps. The Lennard-Jones and Coulomb interactions were truncated at 1.1 nm cutoff, in which the truncation of the Lennard Jones potential was compensated by introducing analytical corrections to pressure and potential energy. The long-range Coulomb interactions were treated using the Particle Mesh Ewald method, with a grid step of 0.16 nm, and a convergence of 10^{-5}.[27] The H-bonds were constrained using the LINCS algorithm.[28]
Subsequently, the energies of the conformers from the MD trajectory were evaluated using the DFTB (3ob-3-1) method[29] in chloroform with the AMS2020 suite.[30-32] The 15 lowest energy conformers were further optimized using Gaussian[33] followed by single point energy calculations (B3LYP/6-311G**//B3LYP/6-31G**). Boltzmann populations were calculated (298 K) from the energy differences and the conformers with a nonnegligible population (> 0.05) were retained for the calculation of the chemical shifts. The chemical shifts were calculated using the protocol outlined in reference 34; the structures were optimized at the B3LYP/6-31+G(d,p) level in vacuum and the chemical shifts were calculated at the mPW1PW91/6-311+G(2d,p) level in chloroform (PCM). The following scaling factors were used to determine the chemical shift from the calculated shielding: slope = -1.0420, intercept = 186.3567. The reported chemical shift was calculated as a weighted average (weighting factor from the Boltzmann population (298 K), energy differences taken from the mPW1PW91/6-311+G(2d,p) calculations) of the calculated chemical shifts of the conformations.

Calculated ^{13}C chemical shifts for selected atoms in fragment B and its isomer

Carbon Number	^{13}C shift natural crenarchaeol (ppm)	^{13}C shift nominal synthetic crenarchaeol (ppm)	^{13}C shift Fragment B (ppm)	^{13}C shift natural calculated (ppm)	^{13}C shift nominal calculated (ppm)
**A10’**	45.81	45.75	45.73	48.33	48.42
**A11’**	39.38	39.07	39.01	41.89	40.99
**A12’**	32.27	31.81	31.80	32.66	32.98
**A13’**	22.40	22.12	22.10	23.78	23.31
**A14’**	44.06	38.30	38.16	40.11	41.24
**A15’**	33.20	32.93	32.92	35.60	36.08
**A16**	30.12	30.51	30.50	31.26	27.20
**A16’**	37.80	33.46	33.51	45.32	34.44
**A19’**	44.13	43.85	43.90	42.03	39.29
**A20’**	22.55	30.13	30.12	19.79	28.57

References

(1) Jones Jr., J.; Kover, W. B. Synthesis of the Four Epimeric Tosylates of (5R)-2, 3-Epoxy-5-isopropenyl-cyclohexanol. *Synth. Commun.* **1995,** *25* (23), 3907.

(2) Kover, W. B.; Jones Jr., J. Syntheses of Chiral Intermediates. Hydrolysis of Terpene Epoxy-Tosylates. *J. Braz. Chem. Soc.* **1996,** *7*, 257.

(3) Kim, K. S.; Song, Y. H.; Lee, B. H.; Hahn, C. S. Efficient and Selective Cleavage of Acetals and Ketals Using Ferric Chloride Adsorbed on Silica Gel. *J. Org. Chem.* **1986,** *51*, 404.

(4) ter Horst, B.; van Wermeskerken, J.; Feringa, B. L.; Minnaard, A. J. Catalytic Asymmetric Synthesis of Mycolipenic and Mycolipanolic Acid. *Eur. J. Org. Chem.* **2010,** *2010*, 38.

(5) Ostermeier, M.; Brunner, B.; Korff, C.; Helmchen, G. Highly Enantioselective Rhodium-Catalyzed Hydrogenation of 2-(2-Methoxy-2-oxoethyl)acrylic Acid− A Convenient Access of Enantiomerically Pure Isoprenoid Building Blocks. *Eur. J. Org. Chem.* **2003,** *2003* (17), 3453.

(6) Ueoka, R.; Bortfeld-Miller, M.; Morinaka, B. I.; Vorholt, J. A.; Piel, J. Toblerols: Cyclopropanol-Containing Polyketide Modulators of Antibiosis in Methylobacteria. *Angew. Chem. Int. Ed.* **2018,** *57* (4), 977.

(7) Roberts, S. W.; Rainier, J. D. Synthesis of an A−E Gambieric Acid Subunit with Use of a C-Glycoside Centered Strategy. *Org. Lett.* **2007,** *9*, 2227.

(8) Fürstner, A.; Kattnig, E.; Lepage, O. Total Syntheses of Amphidinolide X and Y. *J. Am. Chem. Soc.* **2006,** *128*, 9194.

(9) Tokairin, Y.; Konno, H. Preparation of (2R , 3R , 4R)-3-hydroxy-2,4,6-trimethylheptanoic acid via enzymatic desymmertization. *Tetrahedron* **2017,** *73* (1), 39.

(10) Tae, H. S.; Hines, J.; Schneekloth, A. R.; Crews, C. M. Total Synthesis and Biological Evaluation of Tyroscherin. *Org. Lett.* **2010,** *12*, 4308.

(11) Hu, H.; Faraldos, J. A.; Coates, R. M. Scope and Mechanism of Intramolecular Aziridination of Cyclopent-3-enyl-methylamines to 1-Azatricyclo[2.2.1.02,6]heptanes with Lead Tetraacetate. *J. Am. Chem. Soc.* **2009,** *131*, 11998.

(12) Deardorff, D. R.; Windham, C. Q.; Craney, C. L. ENANTIOSELECTIVE HYDROLYSIS OF cis-3,5-DIACETOXYCYCLOPENTENE: (1R,4S)-(+)-4-HYDROXY-2-CYCLOPENTENYL ACETATE. *Org. Synth.* **1996,** *73*, 25.

(13) Tietze, L.; Stadler, C.; Böhnke, N.; Brasche, G.; Grube, A. Synthesis of Enantiomerically Pure Cyclopentene Building Blocks. *Synlett* **2007,** *2007* (3), 485.

(14) Lennon, I. C.; Fox, M. E.; Gerlach, A.; Meek, G.; Praquin, C. A Convenient and Scaleable Synthesis of 11,12-Diamino-9,10-dihydro-9,10-ethanoanthracene and Its Enantiomers. *Synthesis* **2005,** (19), 3196.

(15) Trost, B. M.; Van Vranken, D. L.; Bingel, C. A Modular Approach for Ligand Design for Asymmetric Allylic Alkylations via Enantioselective Palladium-Catalyzed Ionizations. *J. Am. Chem. Soc.* **1992,** *114*, 9327.

(16) Krout, M. R.; Mohr, J. T.; Stoltz, B. M. PREPARATION OF (S)-tert-ButylPHOX (Oxazole, 4-(1,1-dimethylethyl)-2-[2-(diphenylphosphino)phenyl]-4,5-dihydro-(4S)-). *Org. Synth.* **2009,** *86*, 181.

(17) Cernijenko, A.; Risgaard, R.; Baran, P. S. 11-Step Total Synthesis of (-)-Maoecrystal V. *J Am Chem Soc* **2016,** *138* (30), 9425.

(18) Mohr, J. T.; Krout, M. R.; Stoltz, B. M. Preparation of (S)-2-allyl-2-methylcyclohexanone. *Org. Synth.* **2009,** *86*, 194.

(19) Brard, M.; Lainé, C.; Réthoré, R.; Laurent, I.; Neveu, C.; Lemiègre, L.; Benvegnu, T. Synthesis of Archaeal Bipolar Lipid Analogues: A Way to Versatile Drug/Gene Delivery Systems. *J. Org. Chem.* **2007,** *72*, 8267.

(20) Sinninghe Damsté, J. S.; Schouten, S.; Hopmans, E. C.; van Duin, A. C. T.; Geenevasen, J. A. J. Crenarchaeol: the characteristic core glycerol dibiphytanyl glycerol tetraether membrane lipid of cosmopolitan pelagic crenarchaeota. *J. Lipid. Res.* **2002,** *43* (10), 1641.

(21) Schouten, S.; Hoefs, M. J. L.; Koopmans, M. P.; Bosch, H.-J.; Sinnighe Damsté, J. S. Structural characterization, occurrence and fate of archaeal ether-bound acyclyc and cyclic biphytanes and corresponding diols in sediments. *Org. Geochem.* **1998,** *29*, 1305.

(22) Sinninghe Damsté, J. S.; Rijpstra, W. I. C.; Hopmans, E. C.; den Uijl, M. J.; Weijers, J. W. H.; Schouten, S. The enigmatic structure of the crenarchaeol isomer. *Org. Geochem.* **2018,** *124*, 22.

(23) Van Der Spoel, D.; Lindahl, E.; Hess, B.; Groenhof, G.; Mark, A. E.; Berendsen, H. J. GROMACS: Fast, Flexible, and Free. *J. Comput. Chem.* **2005,** *26* (16), 1701.

(24) Jorgensen, W. L.; Maxwell, D. S.; Tirado-Rives, J. Development and Testing of the OPLS All-Atom Force Field on Conformational Energetics and Properties of Organic Liquids. *J. Am. Chem. Soc.* **1996,** *118*, 11225.

(25) Bussi, G.; Donadio, D.; Parrinello, M. Canonical sampling through velocity rescaling. *J. Chem. Phys.* **2007,** *126*, 014101.

(26) Parrinello, M.; Rahman, A. Polymorphic transitions in single crystals: A new molecular dynamics method. *J. Appl. Phys.* **1981,** *52*, 7182.

(27) Caleman, C.; van Maaren, P. J.; Hong, M.; Hub, J. S.; Costa, L. T.; van der Spoel, D. Force Field Benchmark of Organic Liquids: Density, Enthalpy of Vaporization, Heat Capacities, Surface Tension, Isothermal Compressibility, Volumetric Expansion Coefficient, and Dielectric Constant. *J. Chem. Theory Comput.* **2012,** *8* (1), 61.

(28) Hess, B.; Bekker, H.; Berendsen, H. J. C.; Fraaije, J. G. E. M. LINCS: A Linear Constraint Solver for Molecular Simulations. *J. Comput. Chem.* **1997,** *18*, 1463.

(29) Gaus, M.; Goez, A.; Elstner, M. Parametrization and Benchmark of DFTB3 for Organic Molecules. *J. Chem. Theory Comput.* **2013,** *9*, 338.

(30) Fonseca Guerra, C.; Snijders, J. G.; te Velde, G.; Baerends, E. J. Towards an order-N DFT method. *Theor. Chem. Acc.* **1998,** *99*, 391.

(31) te Velde, G.; Bickelhaupt, F. M.; Baerends, E. J.; Fonseca Guerra, C.; Van Gisbergen, S. J. A.; Snijders, J. G.; Ziegler, T. Chemistry with ADF. *J. Comput. Chem.* **2001,** *22*, 931.

(32) Baerends, E. J.; ADF2020.01, SCM; Theoretical Chemistry, Vrije Universiteit Amsterdam, The Netherlands, 2020, www.scm.com.

(33) Frisch, M. J.; Trucks, G. W.; Schlegel, H. B.; Scuseria, G. E.; Robb, M. A.; Cheeseman, J. R.; Scalmani, G.; Barone, V.; Petersson, G. A.; Nakatsuji, H.et al. Wallingford, CT, 2016.

(34) Pierens, G. K. 1H and 13C NMR scaling factors for the calculation of chemical shifts in commonly used solvents using density functional theory. *J. Comput. Chem.* **2014,** *35*, 1388.
